# Bass detection model based on improved YOLOv5 in circulating water system

**DOI:** 10.1371/journal.pone.0283671

**Published:** 2023-03-27

**Authors:** Longqin Xu, Hao Deng, Yingying Cao, Wenjun Liu, Guohuang He, Wenting Fan, Tangliang Wei, Liang Cao, Tonglai Liu, Shuangyin Liu

**Affiliations:** 1 College of Information Science and Technology, Zhongkai University of Agriculture and Engineering, Guangzhou, China; 2 Academy of Intelligent Agricultural Engineering Innovations, Zhongkai University of Agriculture and Engineering, Guangzhou, China; 3 Intelligent Agriculture Engineering Technology Research Center of Guangdong Higher Education Institues, Zhongkai University of Agriculture and Engineering, Guangzhou, China; 4 Guangzhou Key Laboratory of Agricultural Products Quality & Safety Traceability Information Technology, Zhongkai University of Agriculture and Engineering, Guangzhou, China; 5 College of Computer and Information Engineering, Tianjin Agricultural University, Tianjin, China; TU Wien: Technische Universitat Wien, AUSTRIA

## Abstract

The feeding amount of bass farming is closely related to the number of bass. It is of great significance to master the number of bass to achieve accurate feeding and improve the economic benefits of the farm. In view of the interference caused by the problems of multiple targets and target occlusion in bass data for bass detection, this paper proposes a bass target detection model based on improved YOLOV5 in circulating water system. Firstly, acquiring by HD cameras, Mosaic-8, a data augmentation method, is utilized to expand datasets and improve the generalization ability of the model. And K-means clustering algorithm is applied to generate suitable coordinates of prior boxes to improve training efficiency. Secondly, Coordinate Attention mechanism (CA) is introduced into backbone feature extraction network and neck feature fusion network to enhance attention to targets of interest. Finally, Soft-NMS algorithm replaces Non-Maximum Suppression algorithm (NMS) to re-screen prediction boxes and keep targets with higher overlap, which effectively solves the problems of missed detection and false detection. The experiments show that the proposed model can reach 98.09% in detection accuracy and detection speed reaches 13.4ms. The proposed model can help bass farmers under the circulating water system to accurately grasp the number of bass, which has important application value to realize accurate feeding and water conservation.

## Introduction

Bass is an edible fish with high nutritional value. The amount of bait feeding in bass culture is closely related to the number of bass [1]. If the feeding amount is too small, it will easily lead to mutual injury of bass due to starvation and reduce survival rates. If fed too much, it will reduce the quality of water and waste resources. Therefore, monitoring the bass population is beneficial to scientific feeding of bass and control of culture density [2], which is important to achieve efficient bass culture. The traditional methods of bass counting mainly relies on manual estimation, which is time-consuming and error-prone. With the application of computer vision technology in aquaculture, target detection algorithms are adopted to solve the problem of accurate counting by acquiring biological image data through HD cameras and other devices. Traditional target detection algorithms use image processing methods to extract features [3–6]. Zhang [7] applied image processing methods such as binarization, expansion and erosion to extract fry images and employed connected area algorithm and refinement algorithm to count bass in the images. Comparing the two algorithms, the refinement algorithm has higher accuracy for images with high overlap, but it requires high quality image acquisition. In practical applications, most of the acquired images are not suitable for direct counting. After designing a tank with a stable flow rate, Wang [8] used local threshold segmentation to extract targets and calculated the total number of bass from non-overlapping regions in images. In addition, four-neighborhood labeling [9], grayscale image analysis [10], image noise reduction and segmentation [11] are also employed to biological counting. Convolutional neural networks can greatly improve accuracy and generalization ability [12–15], which are widely used in the field of fish target detection [16–21]. An improved YOLOv3 model was proposed by Cui [22] to implement a counting system of puffer fish. The improved YOLOv3 model effectively reduced the occurrence of missed and false detection in overlapping areas, and the counting accuracy can reach 92.5%. Using local image training and migration learning, Lu [23] proposed a lightweight YOLOv4-based shrimp automatic counting model with 92.12% counting accuracy. For high-resolution images, Chen [24] designed an adaptive cropping preprocessing algorithm to augment datasets and proposed a YOLOv5-based model, which achieved 92.55% accuracy. Li [25] improved VGG-19 (a convolutional neural network) to achieve 99.79% accuracy in evaluating iced pomfret freshness. The fish recognition method based on convolutional neural networks have high recognition accuracy, but the inference speed is slow, which cannot meet the demand of rapid multi-target detection. Zhao [26] proposed a fish detection method combining visual attention mechanism SKNet and YOLOv5, it had recognition accuracy of 98.86% and recall rate of 96.64%, 2.14% higher and 2.29% higher compared with YOLOv5. Li [27] proposed a novel method of abnormal behavior detection based on image fusion, the BCS-YOLOv5 based image fusion achieved the best accuracy with an average accuracy of 96.69%. Zhao [28] proposed a high-precision and lightweight end-to-end target detection model based on deformable convolution and improved YOLOv4, the proposed model has an accuracy of 95.47% while the parameter amount is reduced by 10 times and the FPS is doubled. To address the above problems, this paper proposes an improved YOLOv5 model for bass detection under recirculating water aquaculture system, aiming to improve the rapid localization ability and counting accuracy. Taking bass as the research object, the proposed model introduces Coordinate Attention mechanism [29] based on YOLOv5 model to accurately locate and identify the targets of interest, and utilizes the Soft-NMS [30] algorithm instead of the original NMS [31] algorithm for selecting more suitable target boxes.

## Materials and methods

### Experimental steps of proposed work

**Fig 1:**
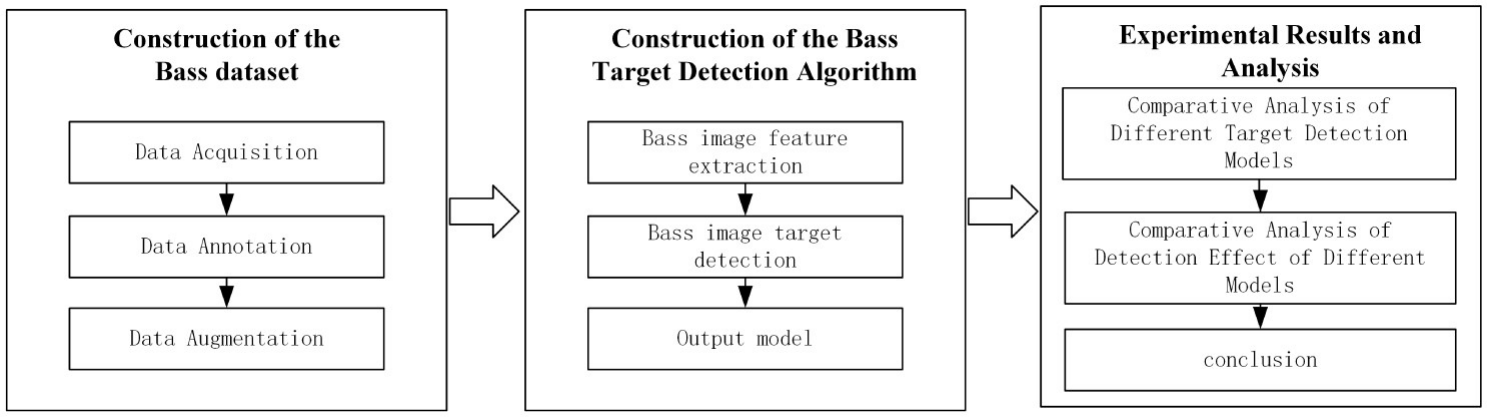
Experimental steps of the proposed work.

In this study, The Experimental steps of the proposed work are shown in Fig 1.

### Data acquisition

**Fig 2:**
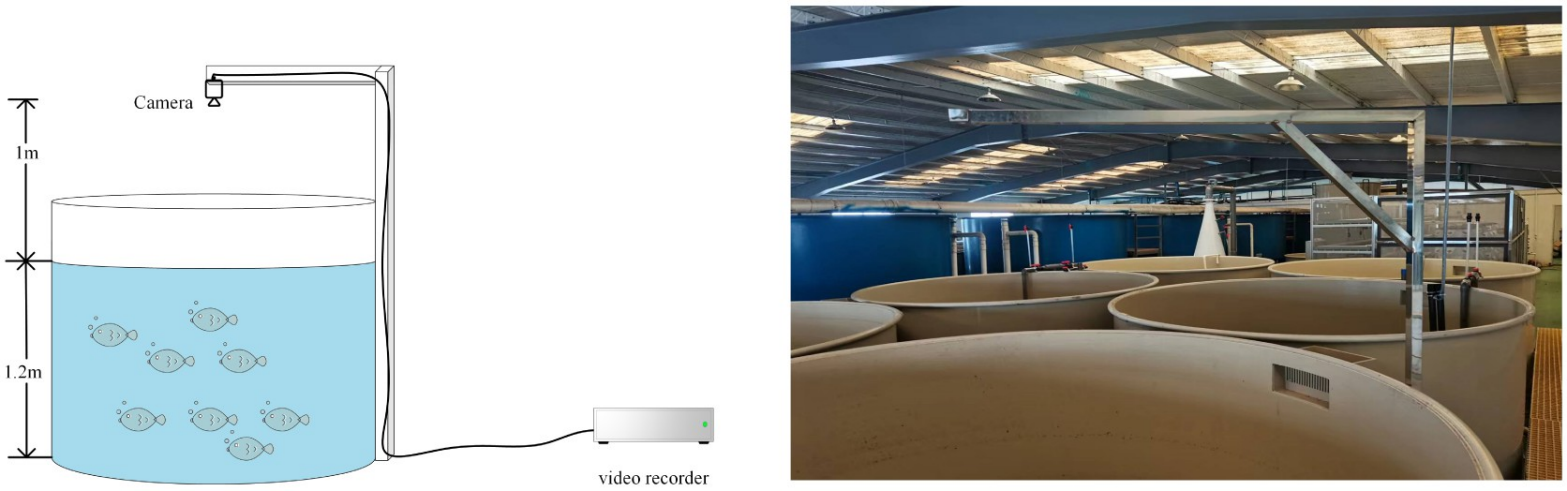
Bass video data acquisition platform.

**Fig 3:**
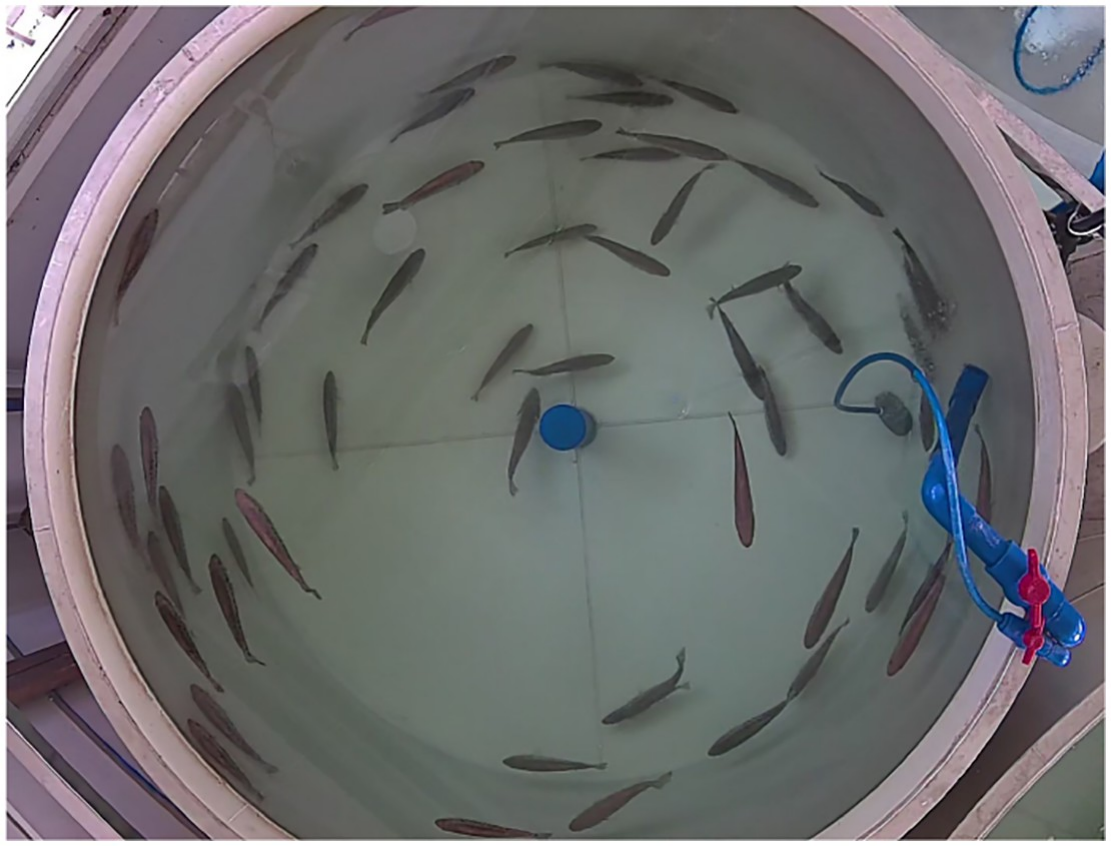
Video capture image.

In the circulating water bass breeding workshop of Guangdong Provincial General Fisheries Technology Promotion Station in Nansha District, Guangzhou, a bass video data collection platform is built, mainly including a circular fish pond made of polypropylene plates, HD cameras and video recorders. As shown in Fig 2, the diameter of the fish pond is 2.7 meters. The datasets are collected from 7:00 am to 18:00 pm. In order to obtain complete images of fish ponds from the top viewing angle, cameras are directly installed above the center of fish pond, with a height of 1 meter from the water surface. The cameras have 1080P resolution, 2.8mm focal length lens, 130 degree wide angle lens, and 30FPS MP4 video capture format. The acquired videos are intercepted as JPG format images, and 1066 photos are intercepted at different time periods and light intensities, as shown in Fig 3.

### Data annotation

**Fig 4:**
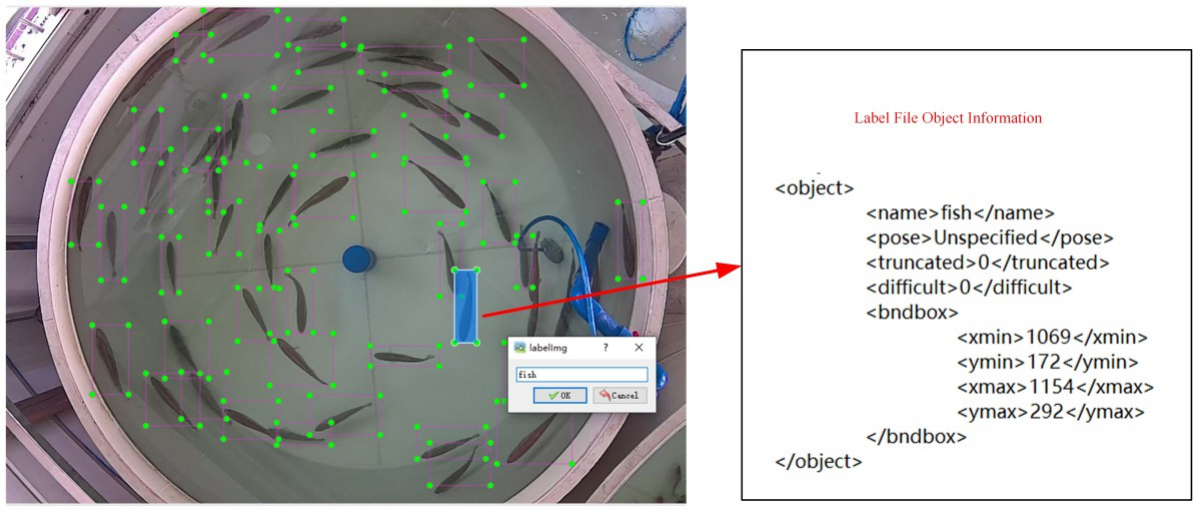
Image annotation.

In this paper, the datasets are manually labeled with the labeling tool *LabelImg* in VOC dataset format, naming label target as *fish*, as shown in Fig 4. The annotation information is stored in an XML file, which corresponds to the image file. Each line stores one target information, in order: target category, X and Y axis coordinates of the center point of the detection boxes, and target width and height.

### Data augmentation

**Fig 5:**
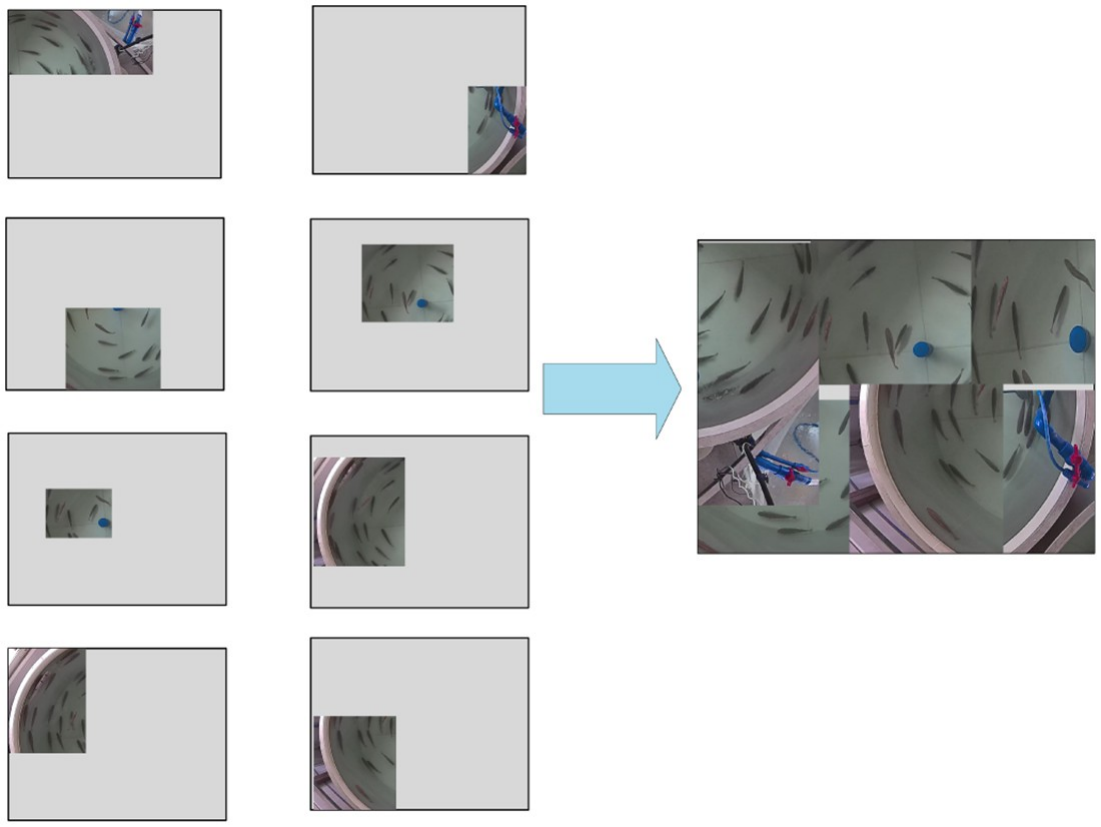
Mosaic-8 data enhancement process.

YOLOv5 model applied the data augmentation method *Mosaic*, which randomly scales and crops four images and then concatenates them to form a single image. This method calculates 4 images at a time during the normalization operation, which can reduce the requirement for memory and speed up training. Therefore, an improved data augmentation method *Mosaic-8* is adopted in this paper. *Mosaic-8* increases the number of images processed from 4 to 8 [32], while random noise and random brightness are introduced to improve robustness and generalization, as shown in Fig 5.

### Improved YOLOv5 bass target detection algorithm

#### Principle of YOLOv5s algorithm

In this paper, lightweight detection model YOLOv5s is applied, which has characteristics of fast detection, high performance, flexibility and rapid deployment. YOLOv5s consists of a backbone feature extraction network (Backbone), a neck feature fusion network (Neck), and a prediction layer (Prediction).

The backbone feature extraction network employs a CSPDarknet network structure that mainly consists of a residual convolution layer (Bottleneck) and a CSPnet network structure, which can increase the depth of networks and enhance the learning ability of convolutional neural networks. Secondly, the backbone feature extraction network introduces a Focus network structure to slice and stack the input images, expand the number of channels, decrease parameters and reduce the memory usage during training. Moreover, the backbone feature extraction network also introduces a SPPF network structure, which employs maximum pooling with different kernel sizes to increase the perceptual field.

Inspired by feature pyramids, the neck feature fusion network consists of a FPN [33] layer with a PAN [34] layer. The FPN layer performs a down-sampling operation to deliver strong semantic information from the top down, while the PAN layer performs an up-sampling operation to deliver strong localization information from the bottom down. They aggregate features from different backbone feature layers to different prediction layers.

**Fig 6:**
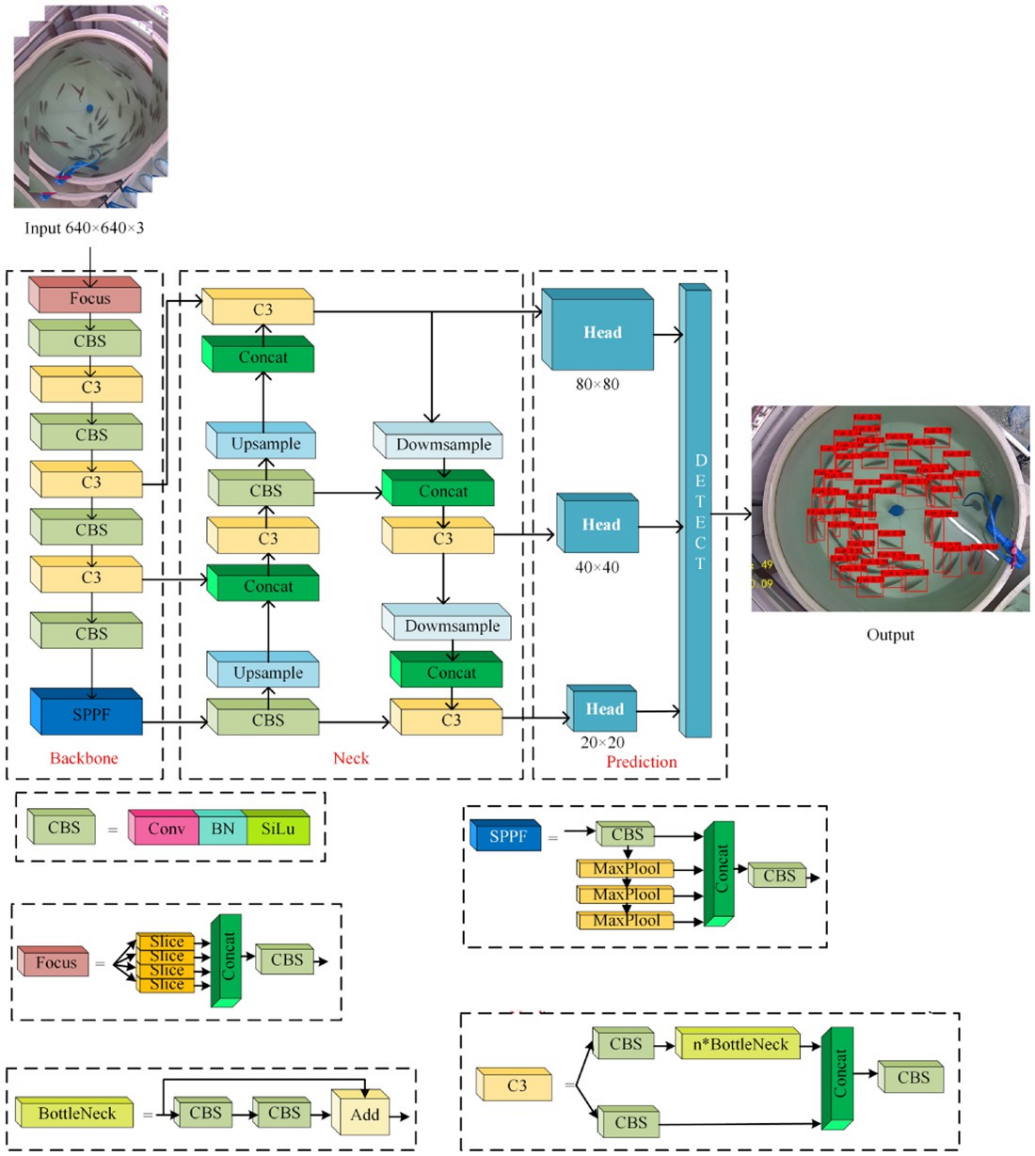
YOLOv5s network structure.

The prediction layer consists of three prediction feature layers, which aim to detect targets of different sizes. The detection results output from the prediction layer are filtered by non-maximal suppression (NMS) to obtain the highest scoring detection boxes. The structure of the YOLOv5s network is shown in Fig 6.

#### Coordinate attention

Since environmental constraints and fixed camera shooting angles, fish overlap and occlusion problems often occur, resulting in low model detection accuracy. Therefore, in this paper, Coordinate Attention mechanism (CA) that combines location information with channel information, is added to capture not only cross-channel information but also direction-aware and location-sensitive information. The method helps to locate and identify the target of interestand improve accuracy in the case of overlap and occlusion.

**Fig 7:**
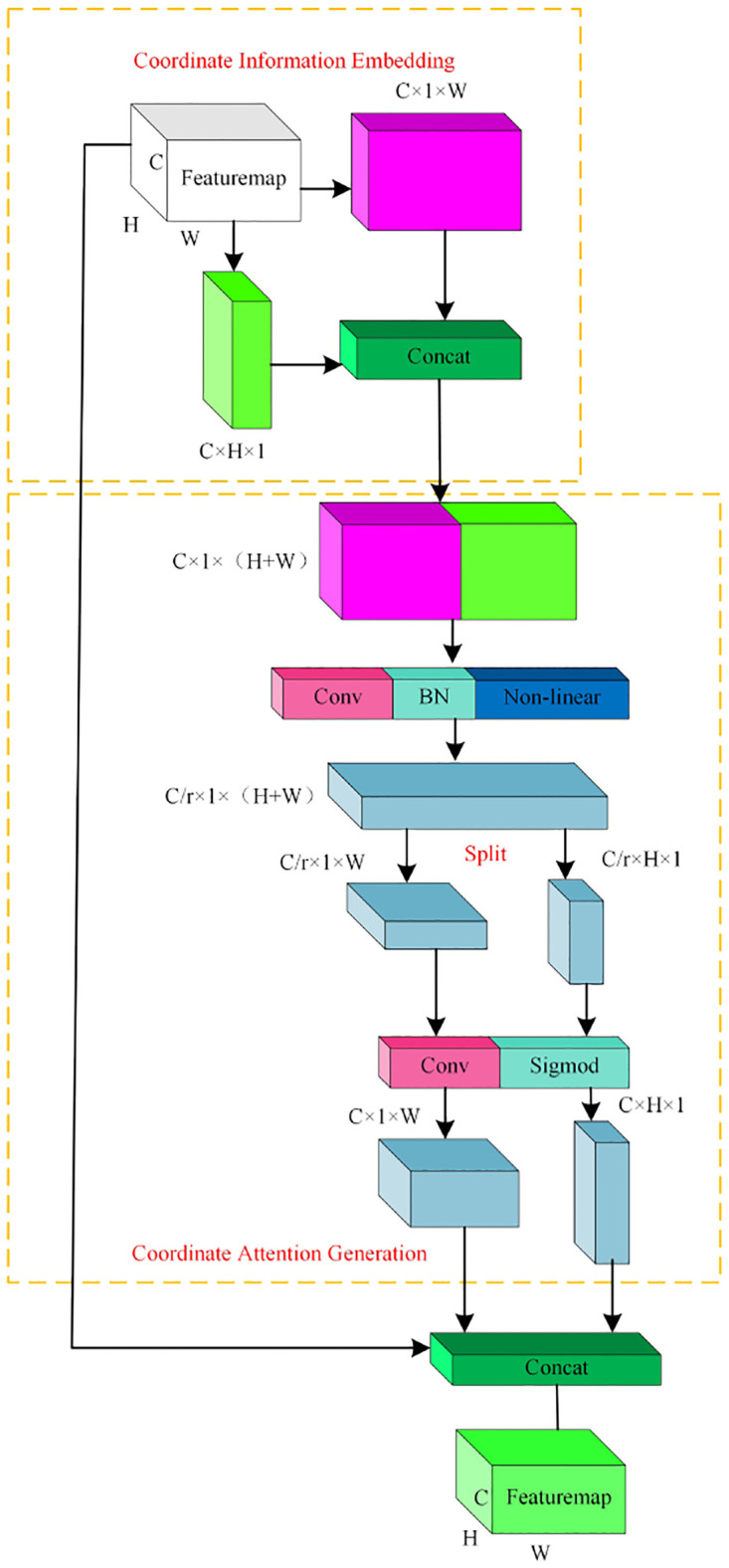
Coordinate attention mechanism.

As shown in Fig 7, CA mechanism is divided into two main steps, namely coordinate information embedding and coordinate attention generation, aim to embed location information into channel attention.

#### Improved backbone feature extraction network

**Fig 8:**
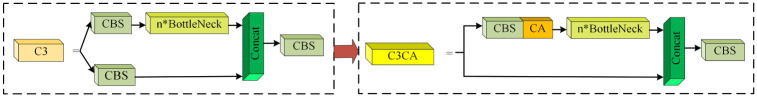
C3 module replaced by C3CA module.

To extract different scale features in the backbone feature extraction network, CA mechanism is inserted into the C3 module, and the CBS module on another branch is removed. As shown in Fig 8, the C3CA module is replaced by the C3 module, which can effectively reduce parameters and make the model more lightweight.

#### Improved neck feature fusion network

Similarly, the neck feature fusion network incorporates CA mechanism to obtain multi-scale feature information, which can help to locate targets of interest.

**Fig 9:**
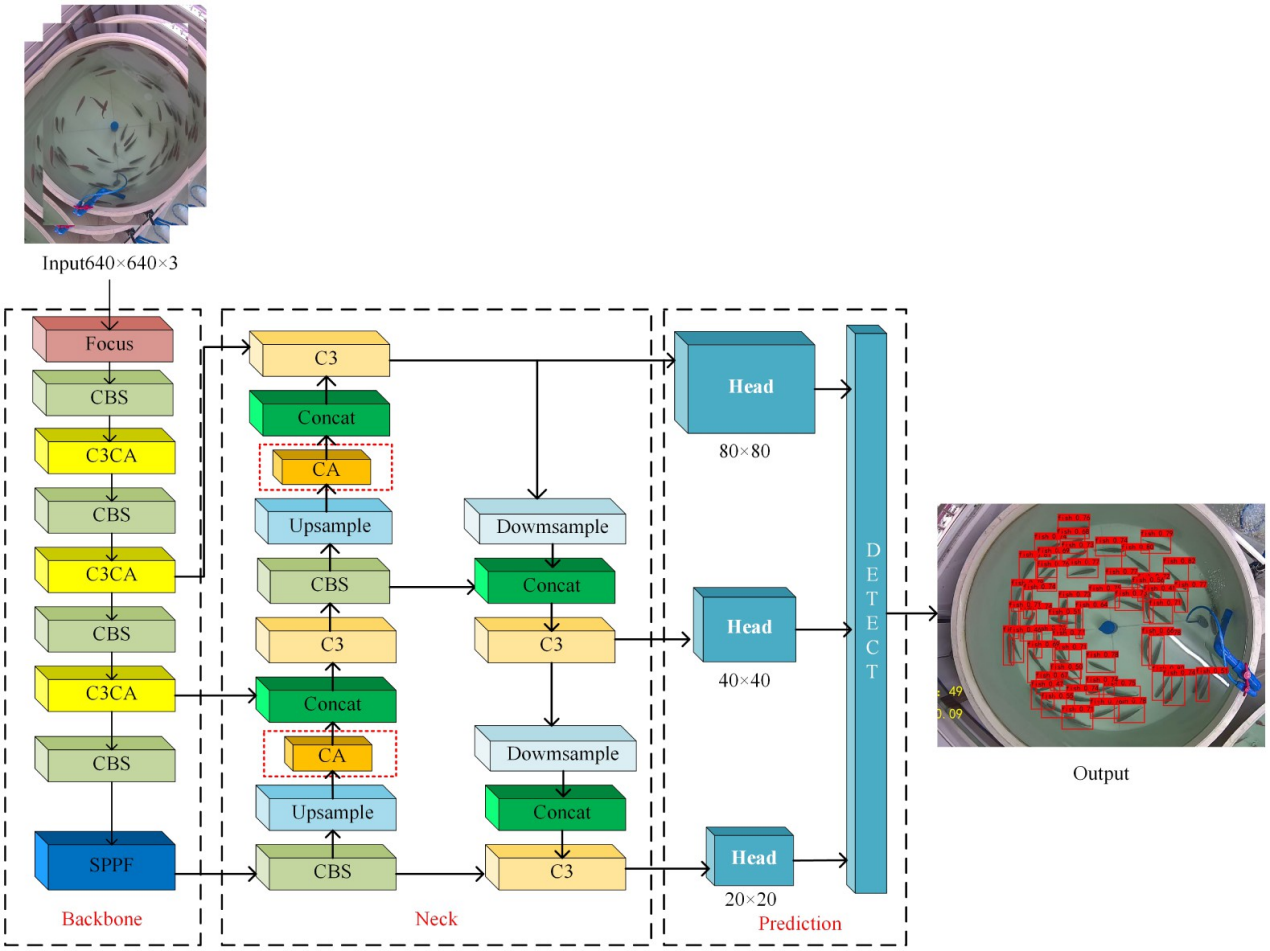
Improved YOLOv5 network structure.

The framework diagram of the improved YOLOv5 perch detection model is shown in Fig 9.

#### Loss function

The loss function of the improved YOLOv5 target detection model is shown in Eq 1, which consists of a bounding box regression loss *L*_*bbox*_, a confidence loss *L*_*cfd*_ and a object class loss *L*_*cls*_.

$$\begin{array}{c} Loss=L_{bbox}+L_{cfd}+L_{cls} \end{array}$$ (1)

The bounding box regression loss *L*_*bbox*_ employs CIOU that takes into account overlap area, central point distance and aspect ratio between ground-turth boxes and predicted boxes, making the detection accuracy higher, as shown in Eqs 3, 4, 5 and 6.

$$\begin{array}{c} L_{bbox}=1-CIOU \end{array}$$ (2)

$$\begin{array}{c} CIOU=IOU-\frac{\rho (b,b^{gt})}{c^{2}}-\alpha v \end{array}$$ (3)

$$\begin{array}{c} IOU=\frac{A\cap B}{A\cup B} \end{array}$$ (4)

$$\begin{array}{c} \alpha =\frac{v}{(1-IOU)+v} \end{array}$$ (5)

$$\begin{array}{c} v=\frac{4}{\pi^{2}}{\left(\text{arctan}\frac{w^{gt}}{h^{gt}}-\text{arctan}\frac{w}{h}\right)}^{2} \end{array}$$ (6)

where IoU denotes the ratio of the intersection of the prediction box A and the ground-turth box B to their concatenation. *ρ* denotes Euclidean distance between the center point *b* of the prediction box and the center point *b*^*gt*^ of ground-turth boxes, and *c* presents the diagonal length of the minimum enclosing box. *α* is a weight parameter, and *v* is a aspect ratio coefficient of the ground-turth box to the predicted box. *h* and *w* denote the height and width of the prediction box, and *h*^*gt*^ and *w*^*gt*^ denote the height and width of ground-turth boxes.

The confidence loss *L*_*cfd*_ is shown as Eq 7.

$$\begin{array}{c} \begin{array}{cc} L_{cfd} & =-\sum_{i=0}^{K\times K}\sum_{j=0}^{M}I_{ij}^{obj}\left[{\hat{C}}_{i}\text{log}C_{i}+\left(1-{\hat{C}}_{i}\right)\text{log}\left(1-{\hat{C}}_{i}\right)\right]\\ & \;-\lambda_{noobj}\sum_{i=0}^{K\times K}\sum_{j=0}^{M}I_{ij}^{noobj}\left[{\hat{C}}_{i}\text{log}C_{i}+\left(1-{\hat{C}}_{i}\right)\text{log}\left(1-{\hat{C}}_{i}\right)\right] \end{array} \end{array}$$ (7)

where *K* × *K* denotes the grid parameters of the output layer, *M* denotes the number of prediction boxes corresponding to each output grid, *C* is the total number of categories, and λ_*noobj*_ is a penalty weight.

The object class loss *L*_*cls*_ is shown as Eq 8.

$$\begin{array}{c} L_{cls}=-\sum_{i=0}^{K\times K}I_{ij}^{obj}\sum_{c\in cls}^{}\left[{\hat{P}}_{i}\left(c\right)\text{log}P_{i}\left(c\right)+\left(1-{\hat{P}}_{i}\left(c\right)\right)\text{log}\left(1-{\hat{P}}_{i}\left(c\right)\right)\right] \end{array}$$ (8)

where *P* is the classification probability, *P*_*i*_(*c*) is the probability that the *i*-th sample is of class *c*, and ${\hat{P}}_{i}\left(c\right)$ is the probability that the *i*-th sample is predicted to be of class *c*.

#### Soft-NMS

In the post-processing stage of YOLOv5, Non-Maximum Suppression (NMS) is used to remove most of duplicate predictors and reduce the incidence of false detection. Prediction boxes are sorted first followed by filtering according to the pre-set threshold, and maximum value prediction boxes are output, as shown in Eq 9.

$$\begin{array}{c} s_{k}=\{\begin{array}{cc} s_{k},\; & A_{IOU}\left(M,b_{k}\right)\geq N_{j}\\ 0,\; & A_{IOU}\left(M,b_{k}\right)<N_{j} \end{array} \end{array}$$ (9)

where *s*_*k*_ is a score of the *k*-th pre-set box, *N*_*j*_ is the preset threshold, *M* is the current highest scoring predictor box, *b*_*k*_ is a *k*-th predictor box to be filtered, and *A*_*IoU*_ is the overlap region between *M* and *b*_*k*_.

When two fish are close to each other in a densely populated image, NMS only keeps the highest scoring prediction boxes and delete low-scoring prediction boxes, resulting in a low recall and missed detection. Therefore, Soft-NMS method is utilized to replace the traditional NMS in this paper, as shown in Eq 10.

$$\begin{array}{c} s_{k}=\{\begin{array}{cc} s_{k},\; & A_{IOU}\left(M,b_{k}\right)\geq N_{j}\\ s_{k}\left(1-A_{IOU}\left(M,b_{k}\right)\right),\; & A_{IOU}\left(M,b_{k}\right)<N_{j} \end{array} \end{array}$$ (10)

Soft-NMS resets scores based on the overlap of similar detection boxes and retains the prediction boxes for next screening, solving the problem that traditional NMS directly removes low-scoring prediction boxes that lead to missed detection.

## Experimental results and analysis

### Experimental platform

All experiments are conducted in Ubuntu 16.04 and Pytorch 1.7.1, where GPU is GeForce GTX 3090 with 32 GB of memory, CPU is Intel(R) Xeon(R) Gold 6146 @2.40 GHz.

**Fig 10:**
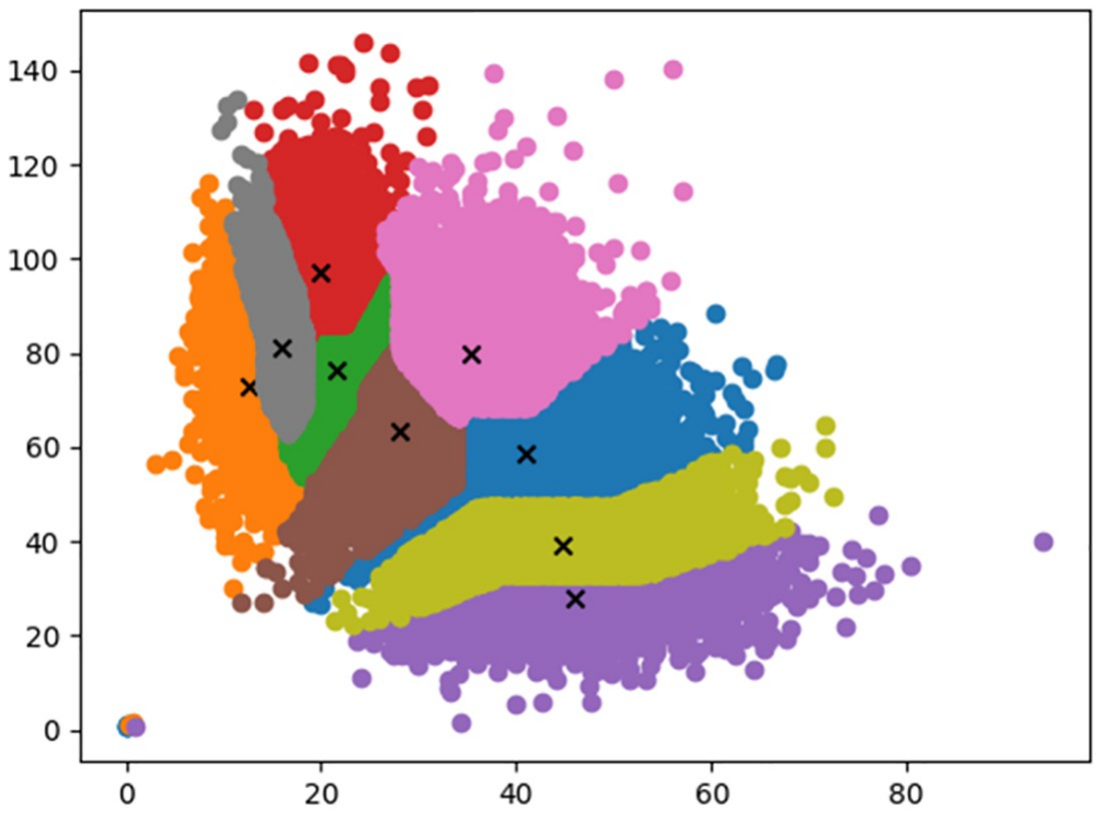
Cluster center distribution diagram.

The original YOLOv5s model introduces a prior boxes to speed up training. In this experiment, K-means algorithm is applied to cluster different sizes of bounding boxes, and 9 clustering centers are regenerated as prior boxes. The coordinates of clustering centers are (12,72), (46,27), (16,81), (21,76), (44,39), (28,63), (20,97), (41,58), and (35,79), respectively, as shown in Fig 10.

### Evaluation metrics

The average precision (AP) and F1 are employed as evaluation metrics, where AP is the area under the Precision-Recall curve, as shown in Eqs 11, 12 and 13.

$$\begin{array}{c} P=\frac{TP}{TP+FP} \end{array}$$ (11)

$$\begin{array}{c} R=\frac{TP}{TP+FN} \end{array}$$ (12)

$$\begin{array}{c} AP=\int_{0}^{1}P\left(R\right)dR \end{array}$$ (13)

where P denotes the probability of correct prediction, R denotes the probability of incorrect prediction, TP is the number of successful predictions, FP is the number of incorrect predictions, and FN is the number of unpredicted predictions.

F1 is the average of precision and recall, as shown in Eq 14.

$$\begin{array}{c} F1=2\cdot \frac{P\cdot R}{P+R} \end{array}$$ (14)

In addition, experiments also analyze the model performance in terms of detection time, number of parameters, and model sizes.

#### Model training and hyperparameter setting

**Fig 11:**
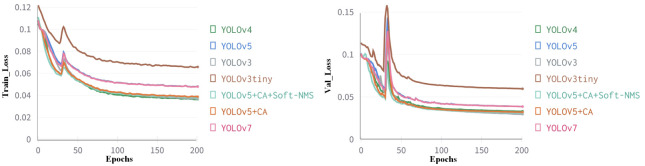
Loss value change curve.

To verify the performance of the proposed model, it is compared with target detection models YOLOv3tiny, YOLOv3, YOLOv4, YOLOv7 and YOLOv5s. The total number of iterations for experiments are 200, the iteration batch size is 32, the optimizer is Adam, and the default parameters are adopted. The learning rate is set to 0.01, and the cosine annealing learning rate adjustment strategy is introduced. The training loss and validation loss of each iteration are recorded and plotted, and the model with the lowest loss in validation set is saved as the training result. The losses for training and validation of different models are shown in Fig 11.

### Comparative analysis of different target detection models

As shown in Table 1, compared with YOLOv5, YOLOv5-CA-Soft-NMS shows 2.91% increase in AP and 8.03% decrease in the amount of parameters, and 1.1ms decreases in detection time. This shows that with the introduction of C3CA module, the backbone feature extraction network can effectively extract location features and channel features to focus on the region of interest. Compared with YOLOv5-CA, YOLOv5-CA-Soft-NMS has an increased detection time by 1.6ms, but it can also meet the requirements of fast detection with higher accuracy. This phenomenon indicates that although Soft-NMS has more computational overhead than NMS, it is more effective in detecting dense overlapping perch targets.

**Table 1:** Comparative experimental results.

Model	AP_0.5	Recall	Precision	F1	Parameters	Model size	Time
YOLOv3tiny	94.7%	88.71%	92.01%	90.32%	8666692	16.61MB	17ms
YOLOv3	98.68%	95.33%	97.34%	96.32%	62546518	193.89MB	46ms
YOLOv4	97.66%	93.71%	95.14%	94.42%	52569318	119.83MB	35ms
YOLOv7	97.71%	93.63%	95.32%	94.26%	37196556	71.32MB	18ms
YOLOv5	95.18%	91.26%	93.52%	92.37%	7012822	13.7MB	14.5ms
YOLOv5-CA	97.64%	92.59%	95.31%	93.93%	6413870	12.59MB	11.8ms
YOLOv5-CA-Soft-NMS	98.09%	93.7%	95.78%	94.72%	6449758	12.67MB	13.4ms

In terms of accuracy, YOLOv5-CA-Soft-NMS improves AP by 3.21% and 0.43% compared with YOLOv3-tiny and YOLOv4, respectively. However, compared with YOLOv3, AP is reduced by 0.59%. It is possible that YOLOv3 classifies incorrect targets as correct predictions, resulting in higher AP.

In terms of performance, compared with YOLOv5-CA-Soft-NMS, YOLOv3-tiny, YOLOv3, YOLOV4 and YOLOv7 has 1.3 times, 9.69 times, 8.15 times and 5.76 times higher number of parameters, 1.31 times, 15.3 times, 9.45 times and 5.63 times higher model size, and 1.26 times, 3.4 times, 2.6 times and 1.34 times higher detection time, respectively. As shown in Table 1, YOLOv5-CA-Soft-NMS has a lower number of parameters, smaller model weights and better detection speed.

### Comparative analysis of detection effect of different models

**Fig 12:**
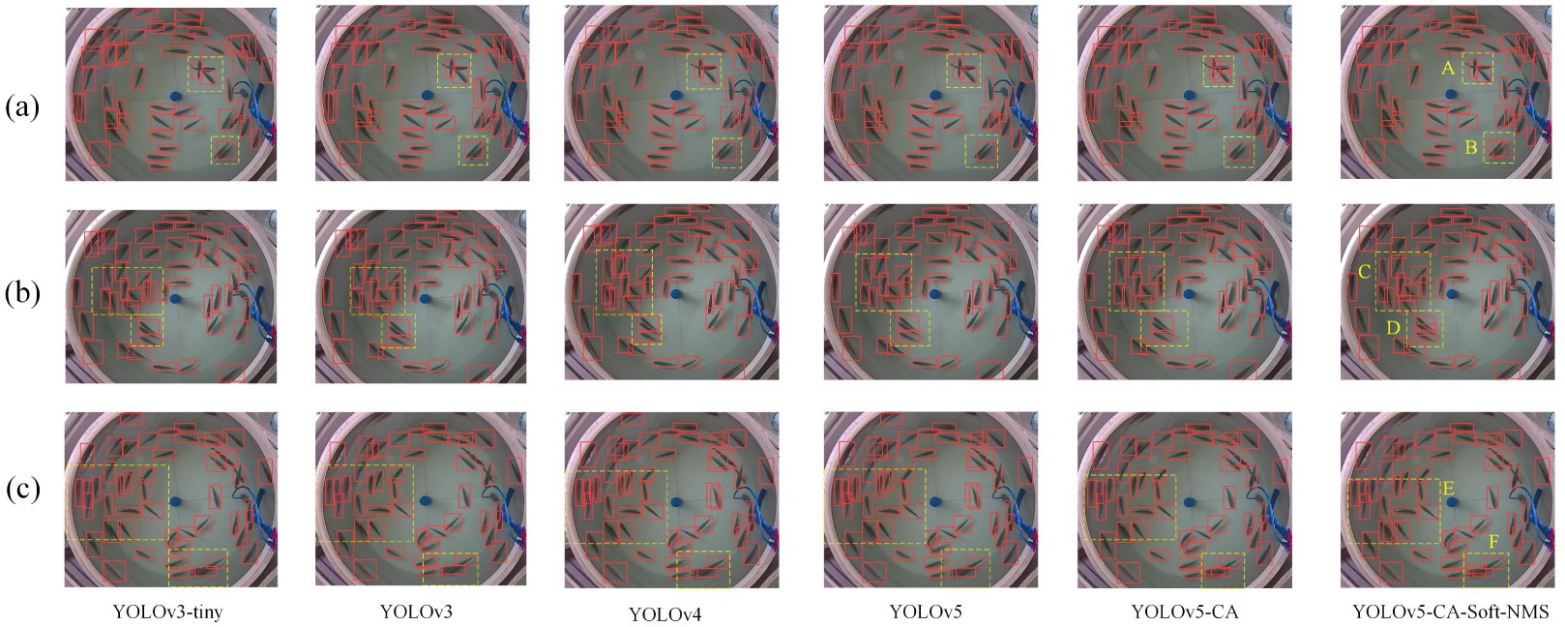
Comparison of detection effects of different models.

To verify the effectiveness of detection, YOLOv5-CA-Soft-NMS and other models are compared on the test set, as shown in Fig 12.

In region A of Fig 12(a), YOLOv5-CA successfully detects all targets that cross-obscure each other, and all other models have missed detection. In region B of Fig 12(a), YOLOv5-CA-Soft-NMS successfully detects all parallel-obscured targets, while all other models have missed detection phenomena.

In region C of Fig 12(b), YOLOv5-CA successfully detects all densely overlapping targets, and all other models suffer from false detection.

In region E of Fig 12(c), there are partially overlapping targets and a large number of independent targets. YOLOv5-CA-Soft-NMS successfully detects all targets, while other models have missed or false detection. There are targets with a high degree of overlap in region F of Fig 11(c), and YOLOv5-CA-Soft-NMS and YOLOv5-CA successfully detect all targets, while other models have missed detection.

In summary, YOLOv5-CA-Soft-NMS detects targets better than other models and effectively improves accuracy in the case of target occlusion.

## Conclusions

For the problems of multiple target occlusion and overlap in fish dense farming scenarios, this paper proposes a bass target detection model based on improved YOLOv5.

To effectively detect bass targets, CA mechanism is inserted to improve the backbone feature extraction network and neck feature fusion network of YOLOv5 to capture cross-channel information, orientation awareness and location sensitive information. The average mean accuracy of model detection is improved by 2.46% compared with YOLOv5s model.

To effectively retain targets with high overlap degree and improve the recognition accuracy, Soft-NMS replaces NMS to re-screen prediction boxes. The detection accuracy is improved by 2.91% compared with YOLOv5s.

In this paper, we propose a bass detection model, YOLOv5-CA-Soft-NMS, which has a detection speed of 13.4ms and a detection accuracy of 98.09%. Compared with YOLOv3-tiny, YOLOv3, YOLOv4, YOLOv5 and YOLOv7, it has higher detection accuracy and good detection speed. The proposed model is more suitable for rapid detection of bass population, so that farmers can feed bait scientifically, control cost and save resources, which has high practical application value.

## Supporting information

S1 Dataset (ZIP)
