# Supplementary figures and images for: Bass detection model based on improved YOLOv5 in circulating water system (part 2 of 2)
Source: PLoS One. 2023 Mar 27;18(3):e0283671. doi: 10.1371/journal.pone.0283671 (PMC10042332; doi:10.1371/journal.pone.0283671)

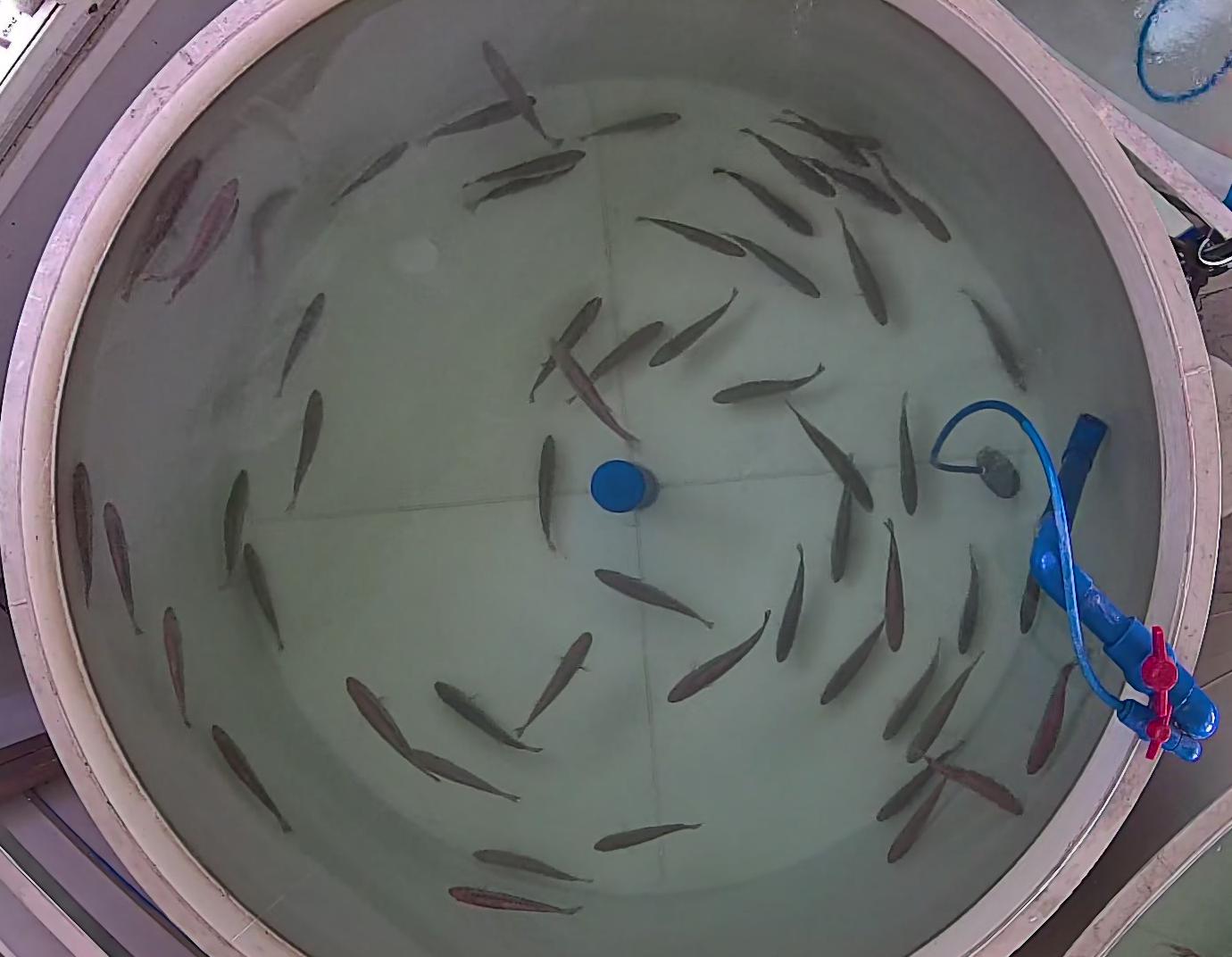

Supplement: S1 Dataset — (ZIP) [file pone.0283671.s001.zip › datasets/00101.jpg]

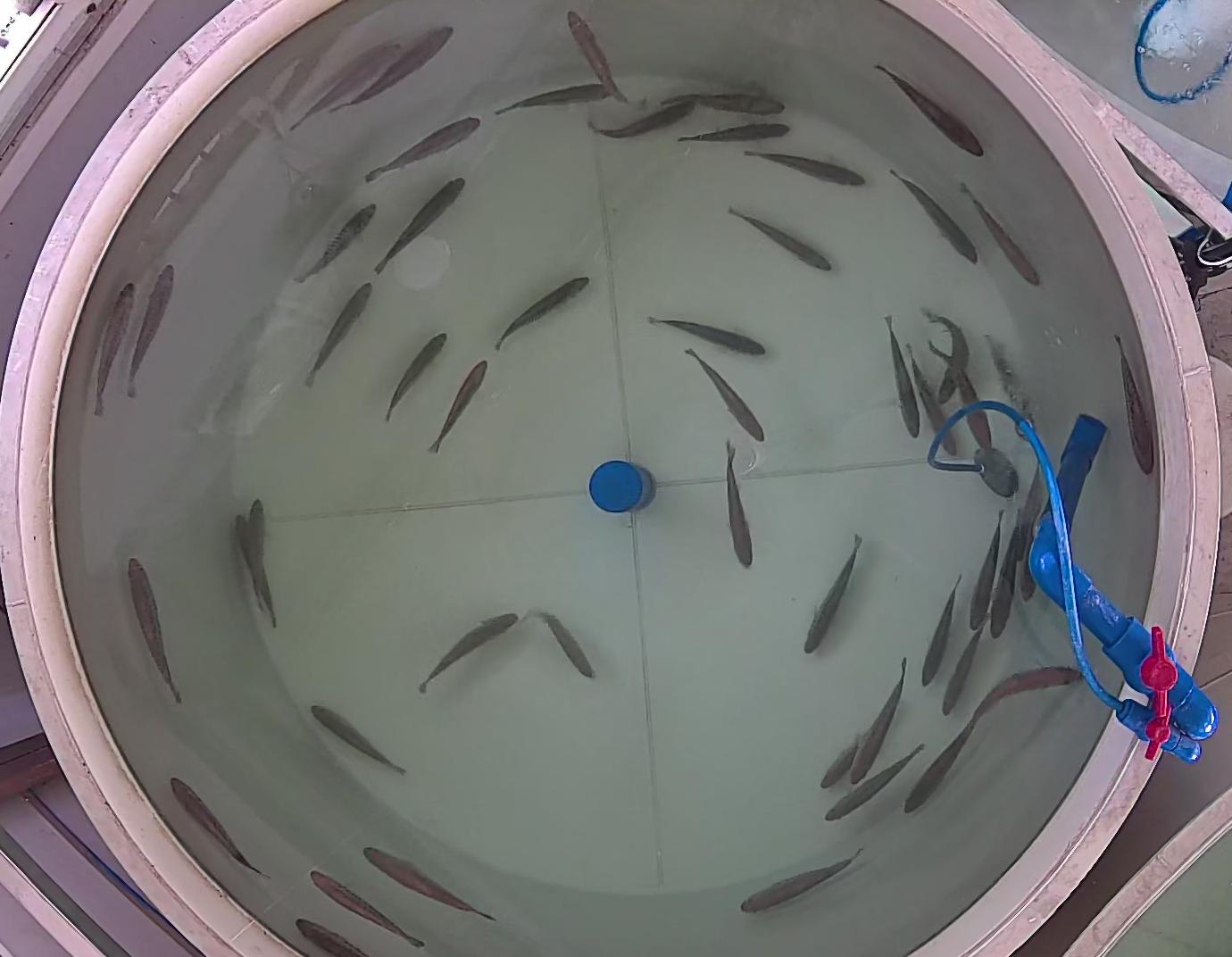

Supplement: S1 Dataset — (ZIP) [file pone.0283671.s001.zip › datasets/00102.jpg]

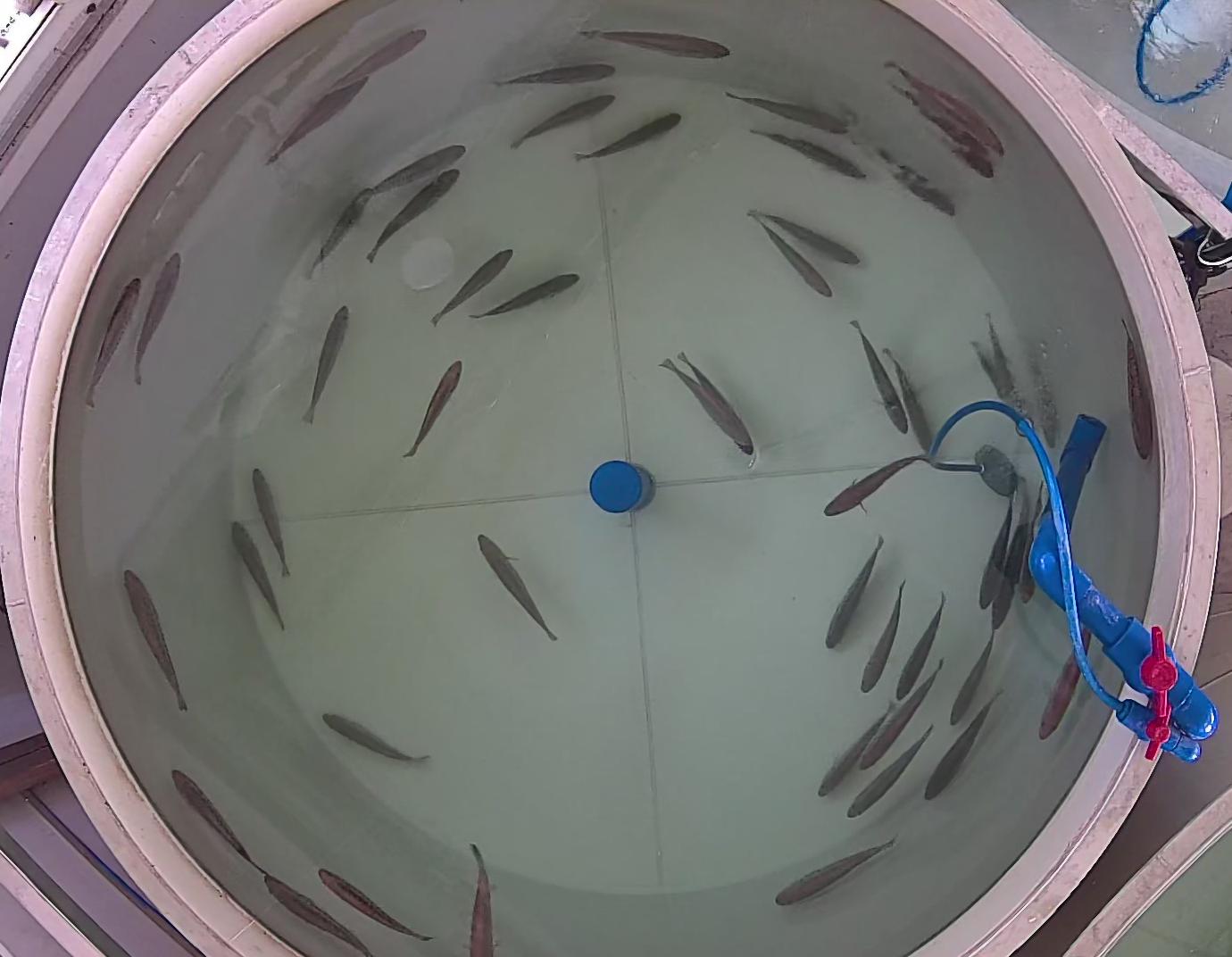

Supplement: S1 Dataset — (ZIP) [file pone.0283671.s001.zip › datasets/00103.jpg]

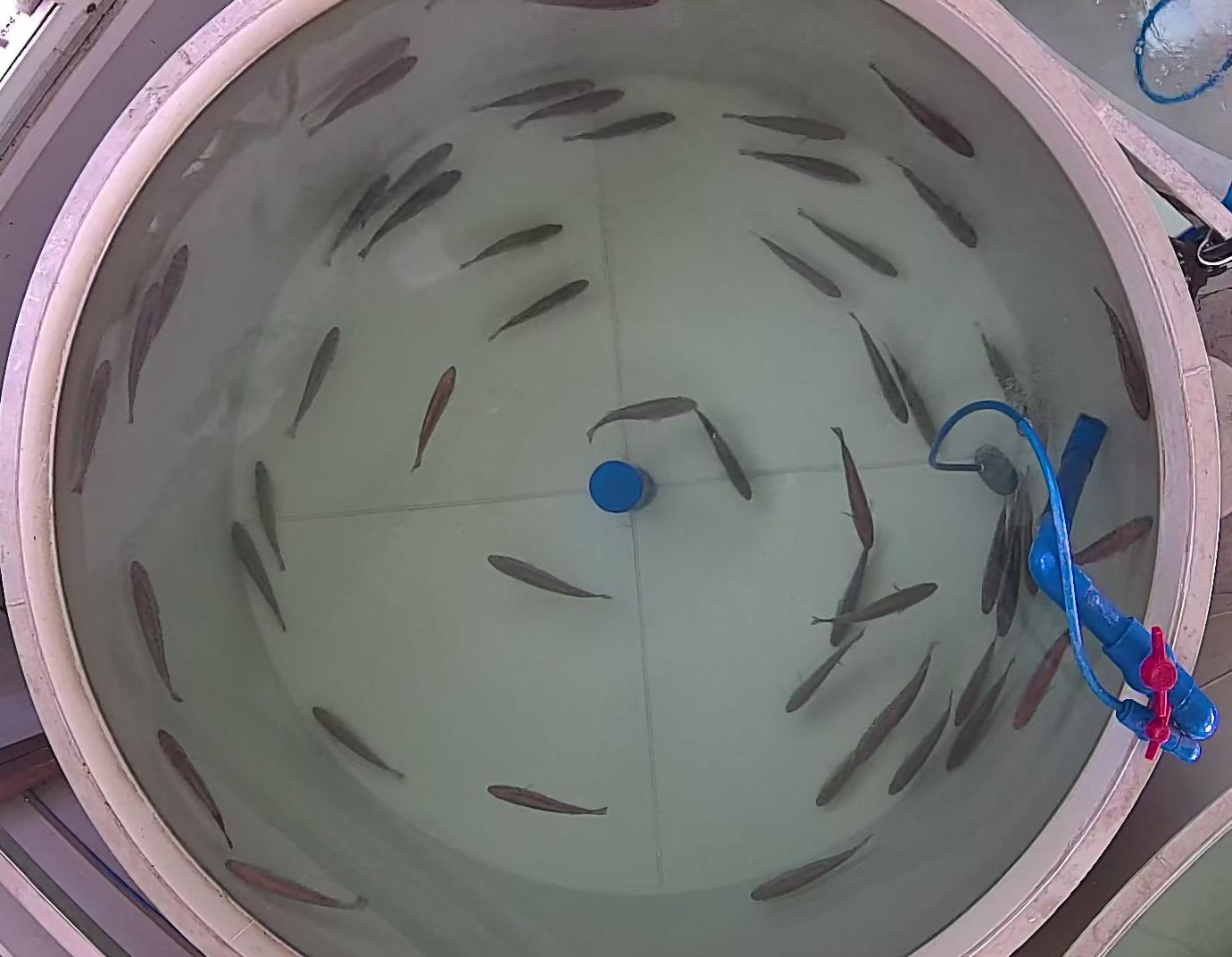

Supplement: S1 Dataset — (ZIP) [file pone.0283671.s001.zip › datasets/00104.jpg]

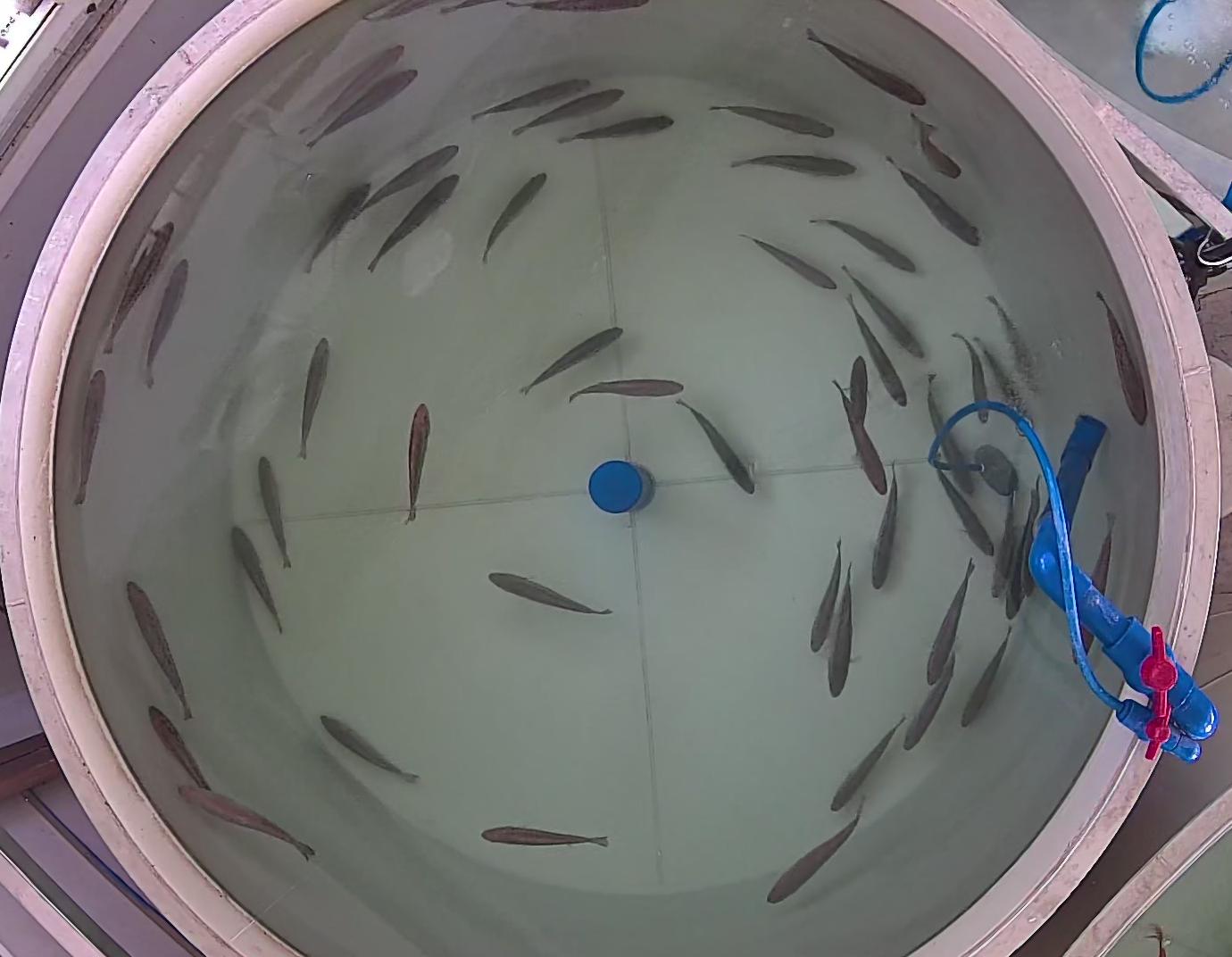

Supplement: S1 Dataset — (ZIP) [file pone.0283671.s001.zip › datasets/00105.jpg]

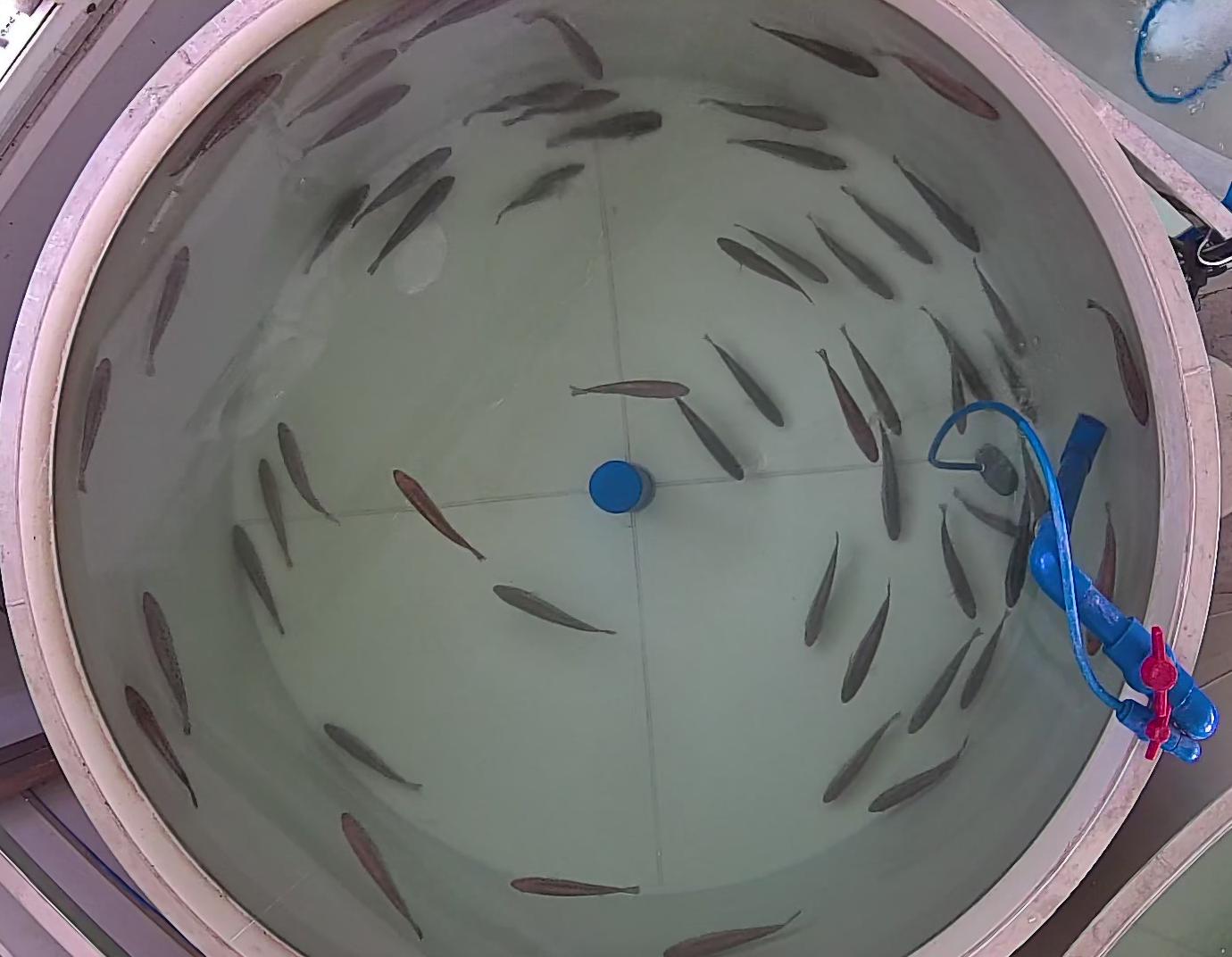

Supplement: S1 Dataset — (ZIP) [file pone.0283671.s001.zip › datasets/00106.jpg]

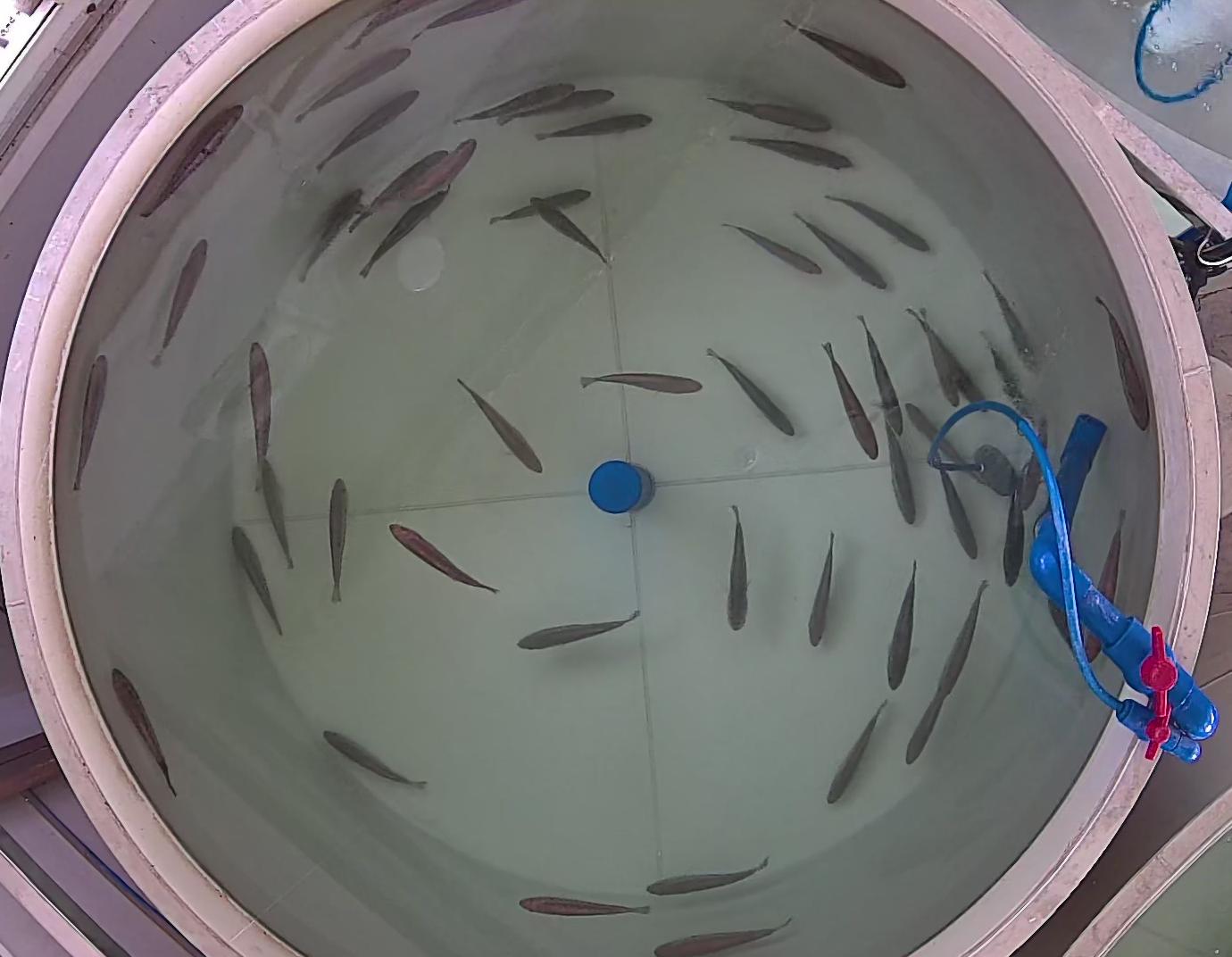

Supplement: S1 Dataset — (ZIP) [file pone.0283671.s001.zip › datasets/00107.jpg]

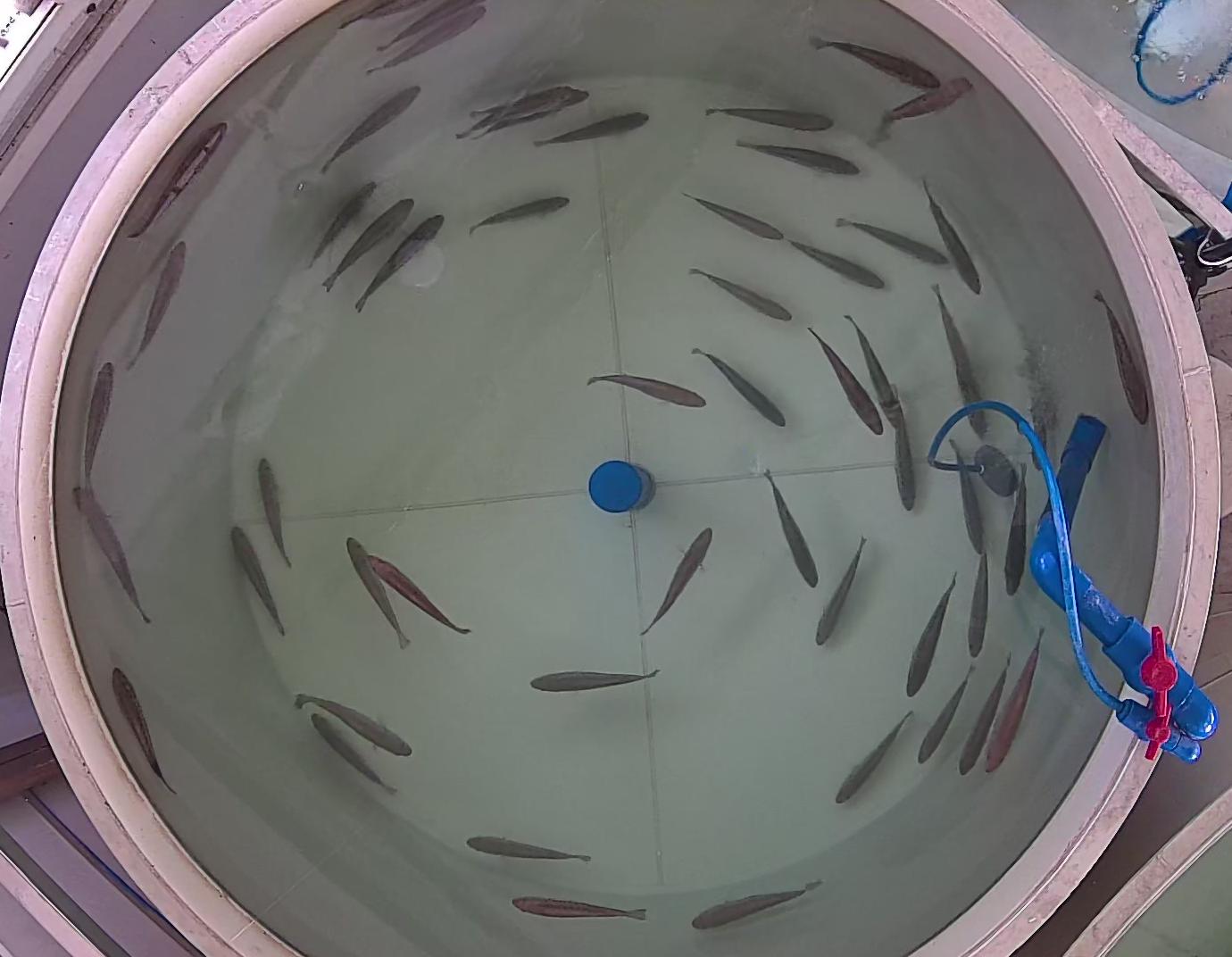

Supplement: S1 Dataset — (ZIP) [file pone.0283671.s001.zip › datasets/00108.jpg]

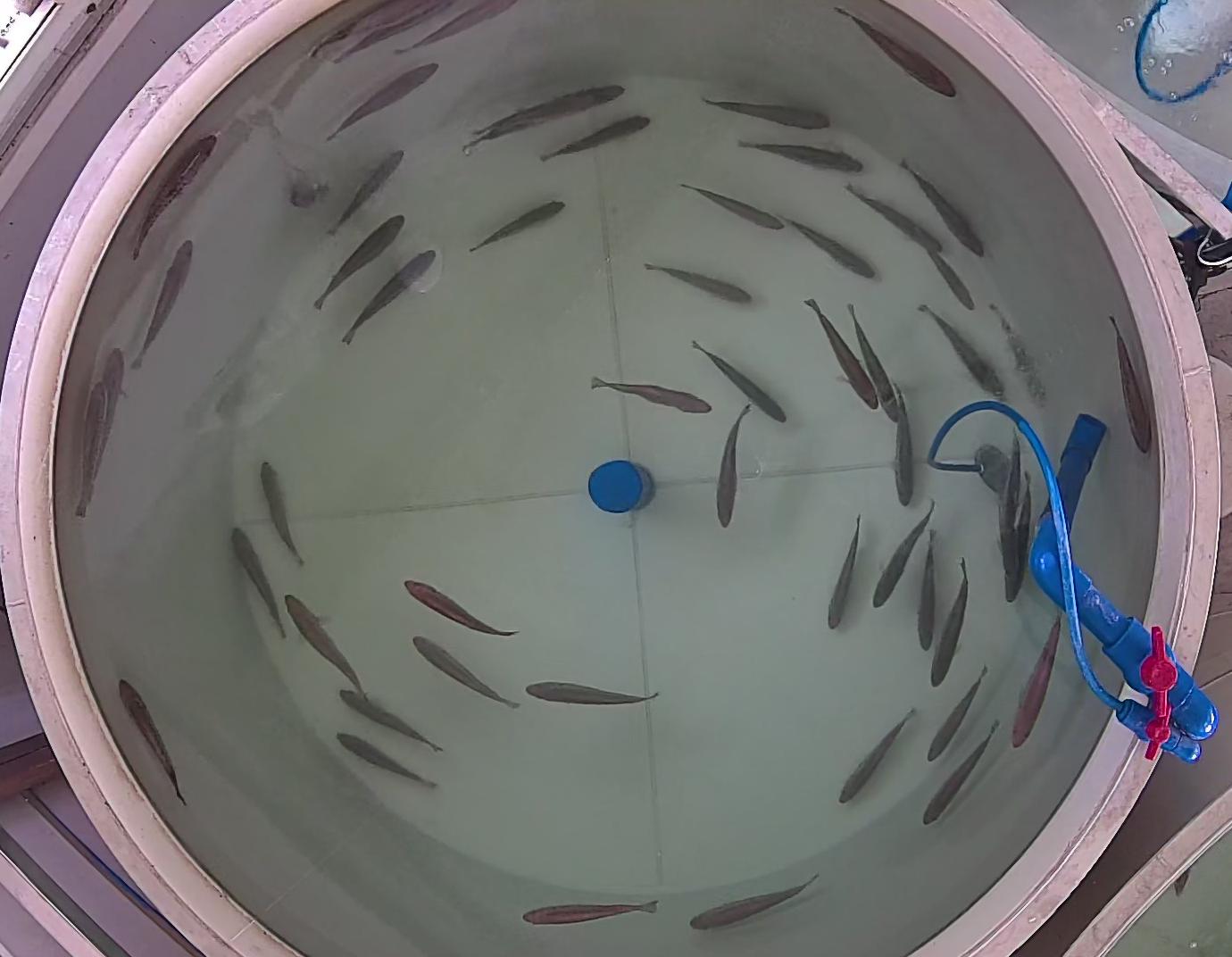

Supplement: S1 Dataset — (ZIP) [file pone.0283671.s001.zip › datasets/00109.jpg]

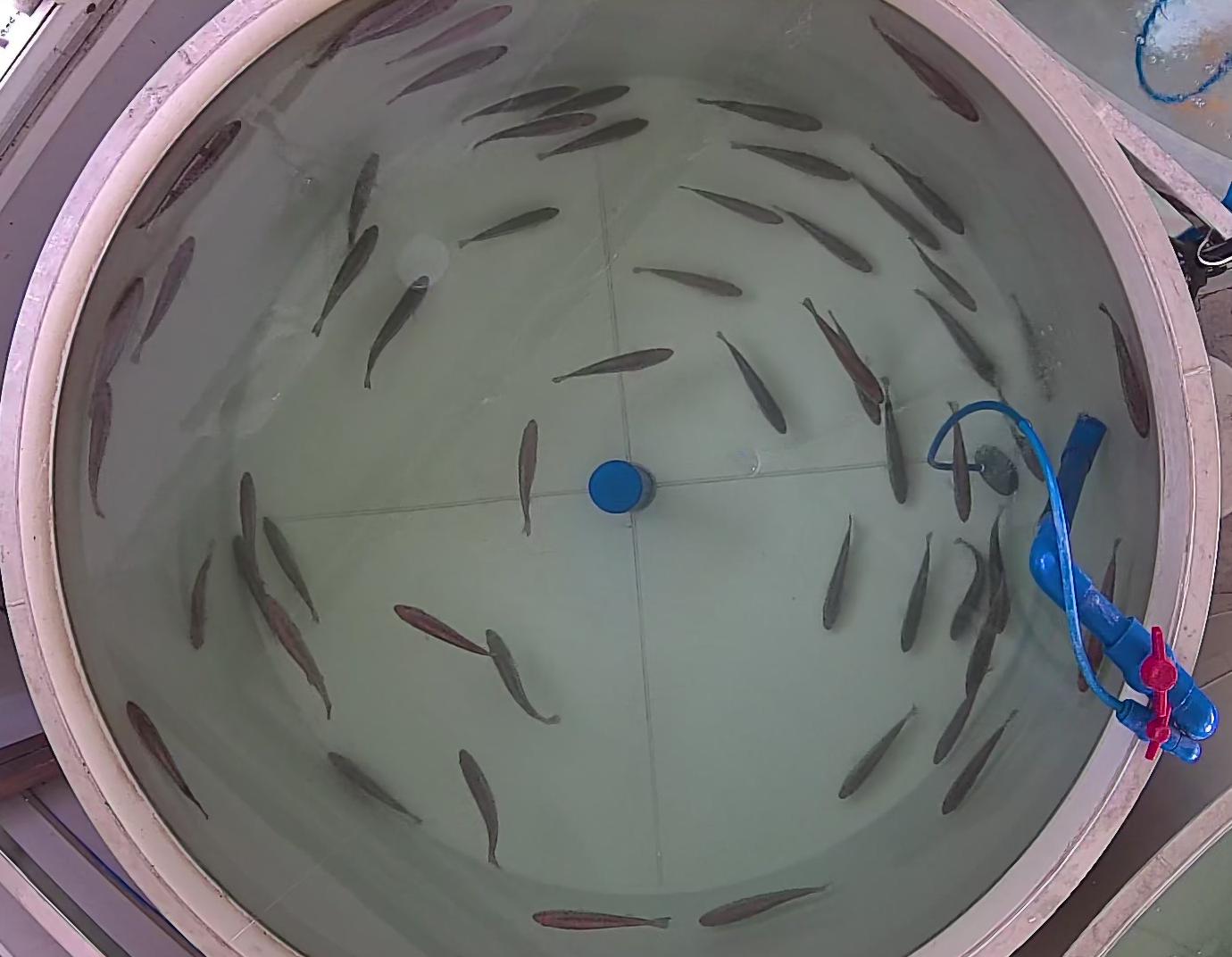

Supplement: S1 Dataset — (ZIP) [file pone.0283671.s001.zip › datasets/00110.jpg]

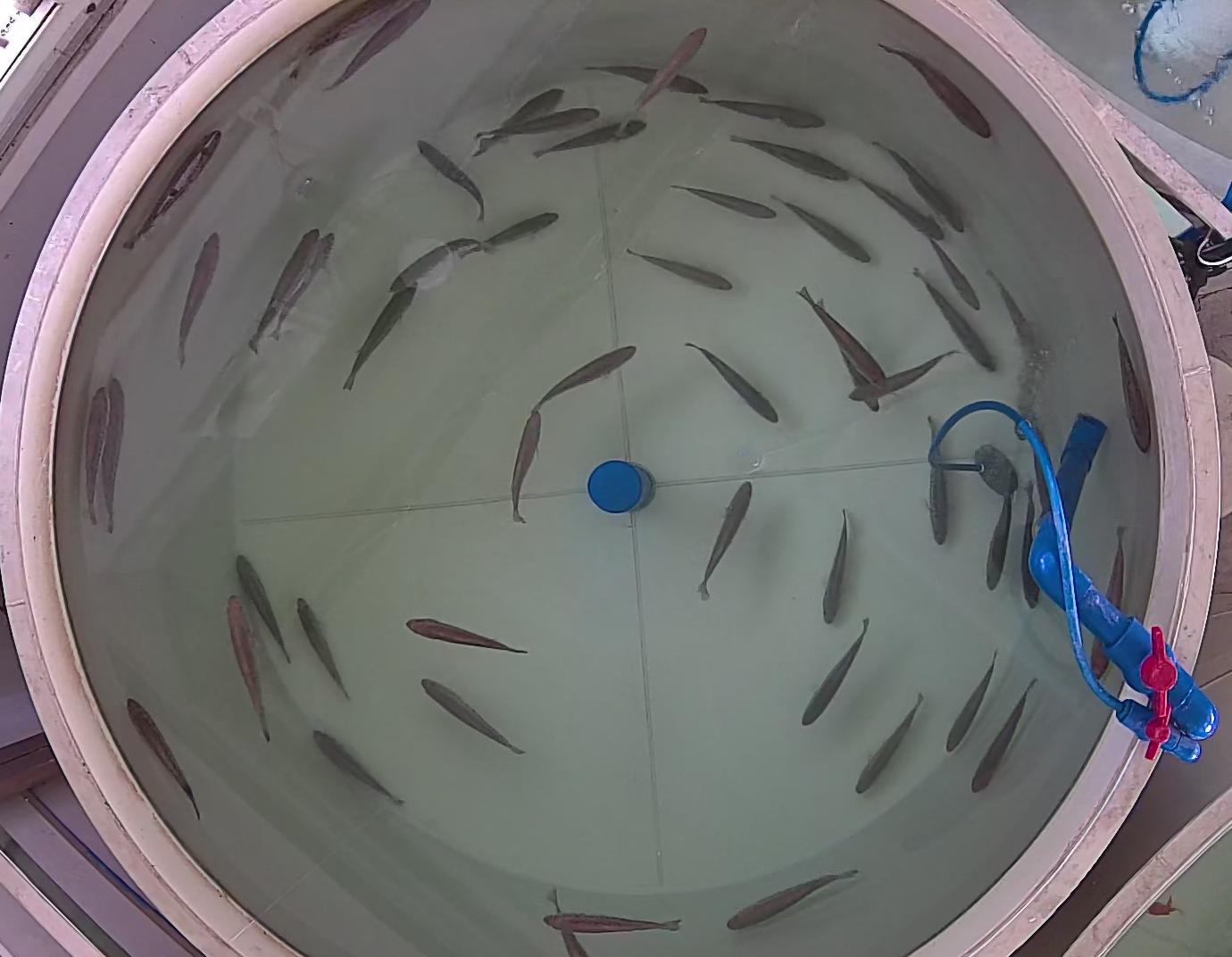

Supplement: S1 Dataset — (ZIP) [file pone.0283671.s001.zip › datasets/00111.jpg]

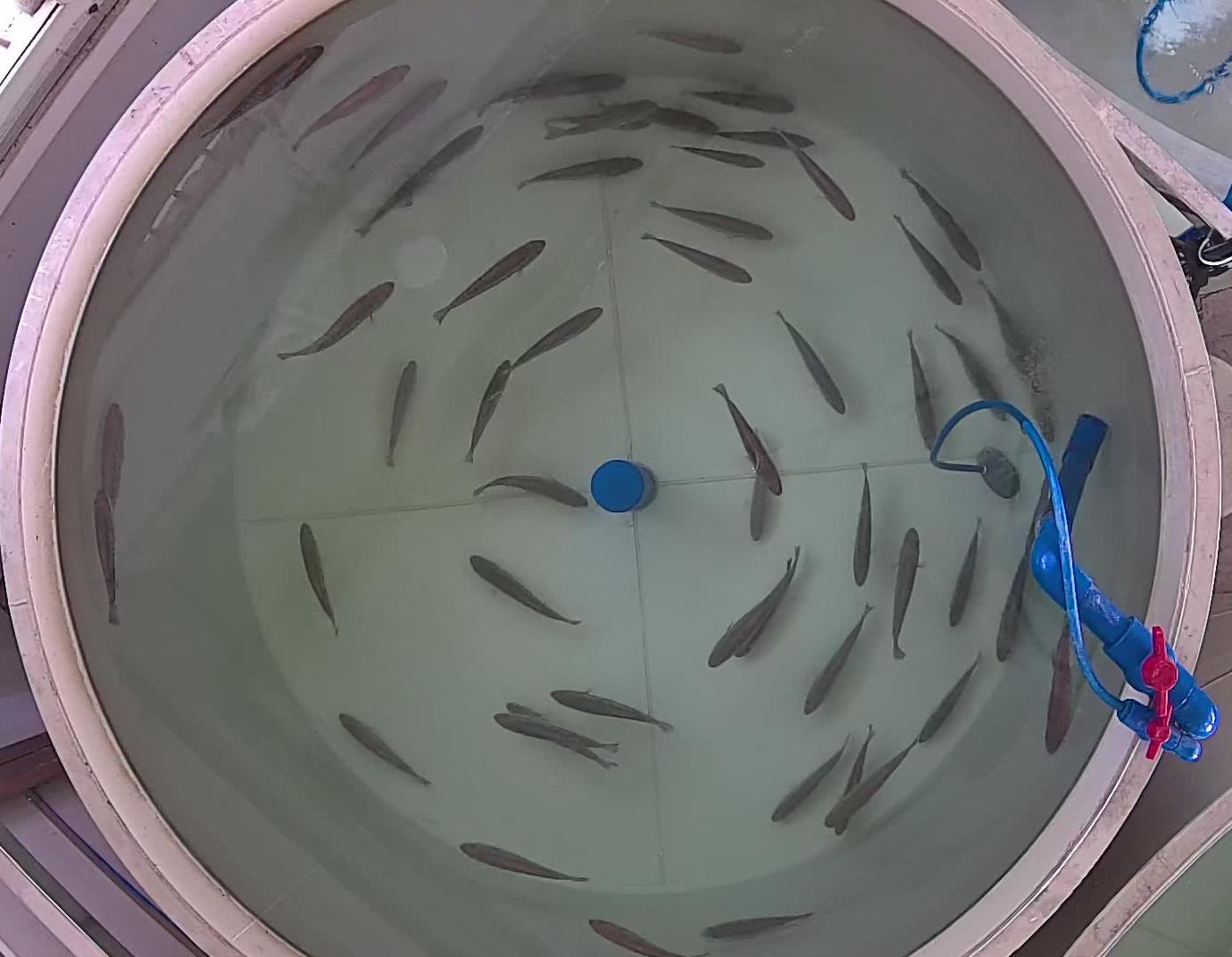

Supplement: S1 Dataset — (ZIP) [file pone.0283671.s001.zip › datasets/00112.jpg]

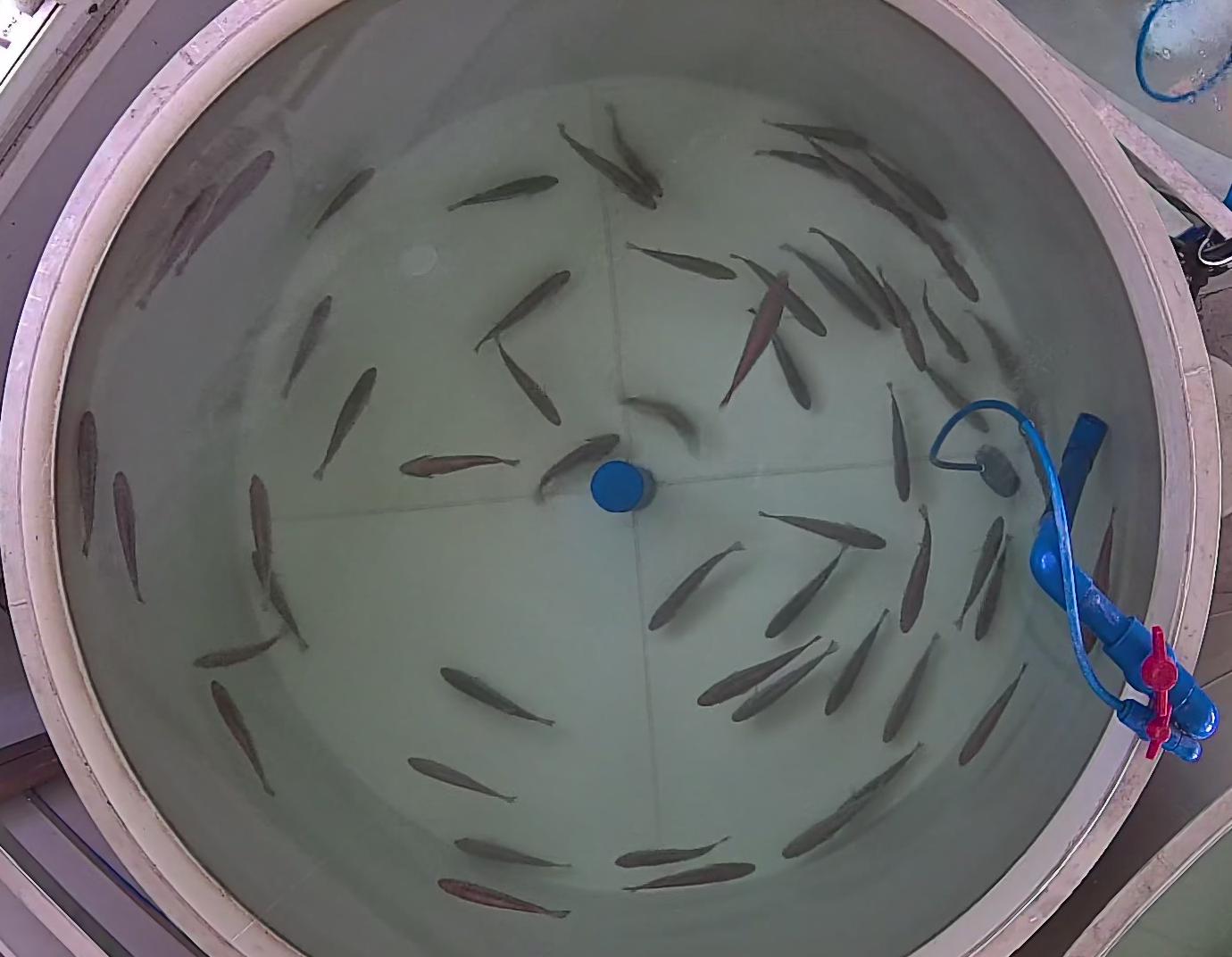

Supplement: S1 Dataset — (ZIP) [file pone.0283671.s001.zip › datasets/00113.jpg]

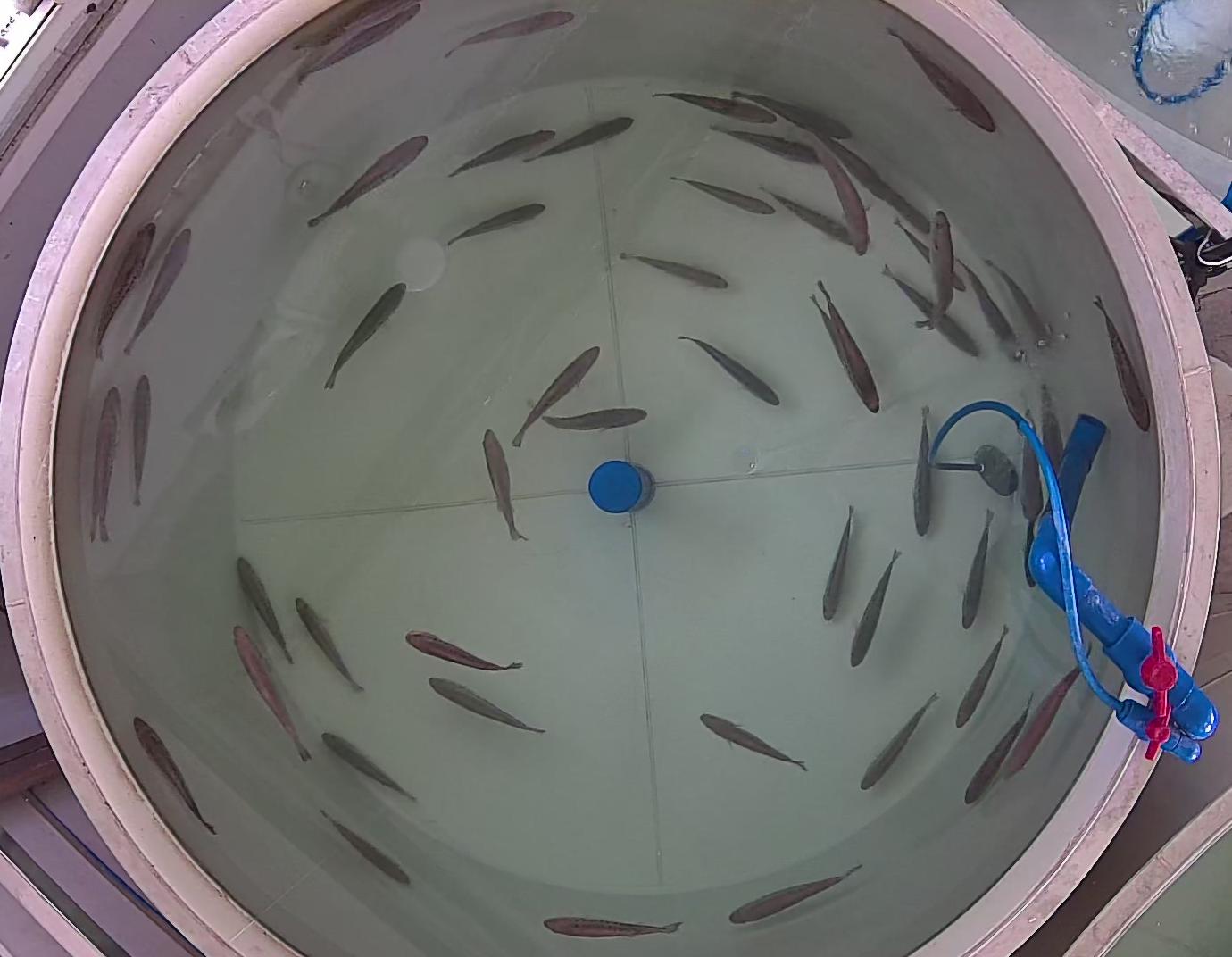

Supplement: S1 Dataset — (ZIP) [file pone.0283671.s001.zip › datasets/00114.jpg]

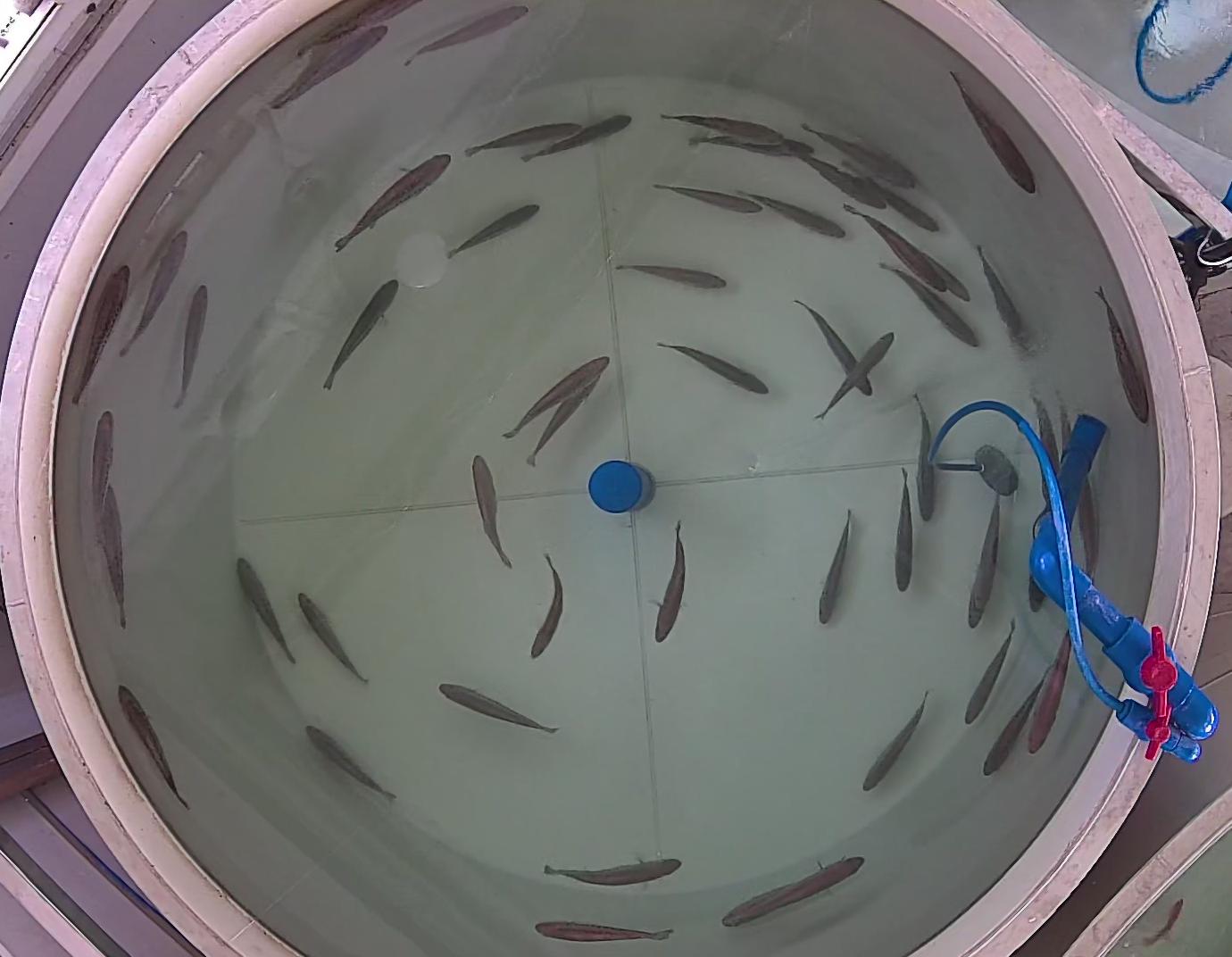

Supplement: S1 Dataset — (ZIP) [file pone.0283671.s001.zip › datasets/00115.jpg]

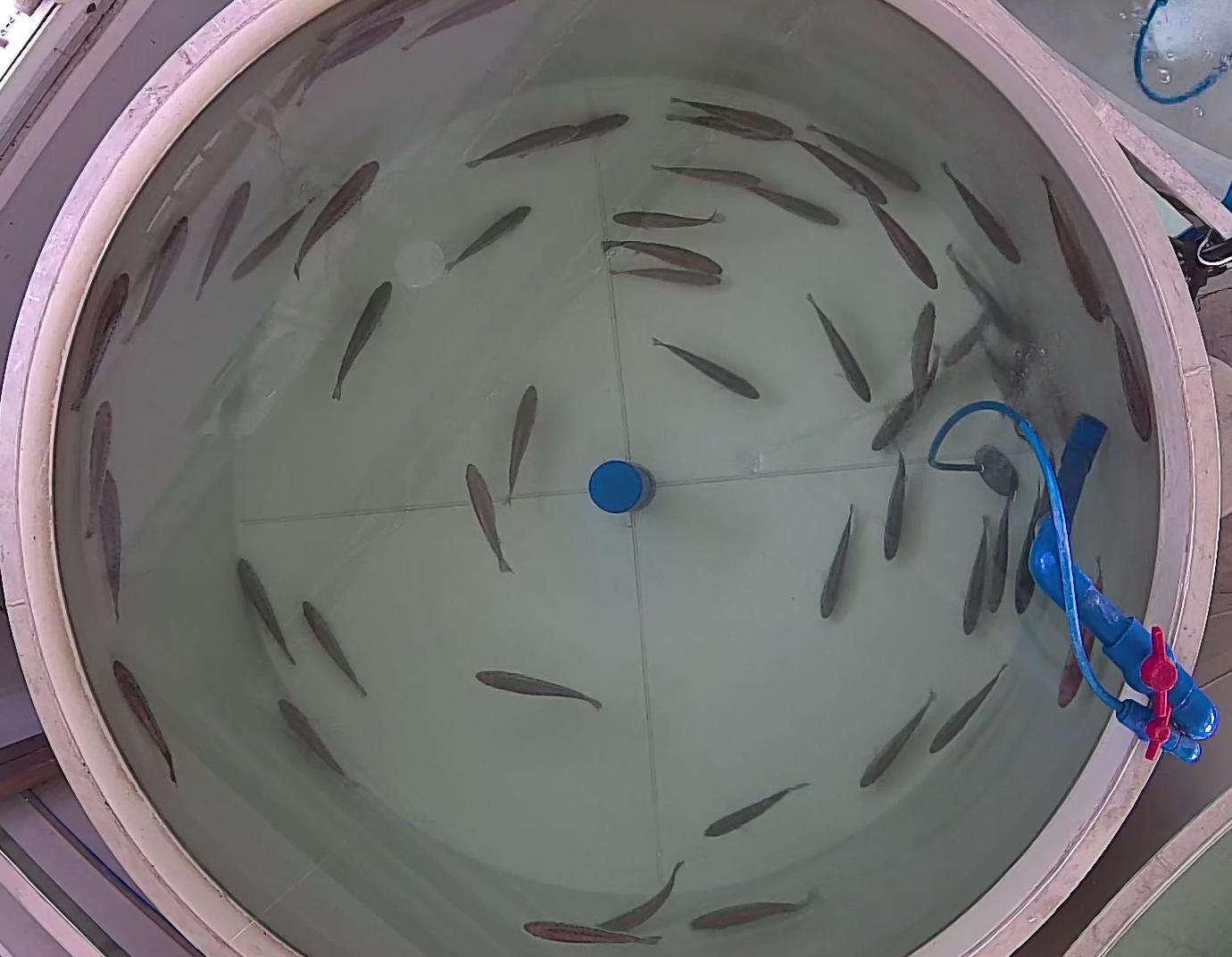

Supplement: S1 Dataset — (ZIP) [file pone.0283671.s001.zip › datasets/00116.jpg]

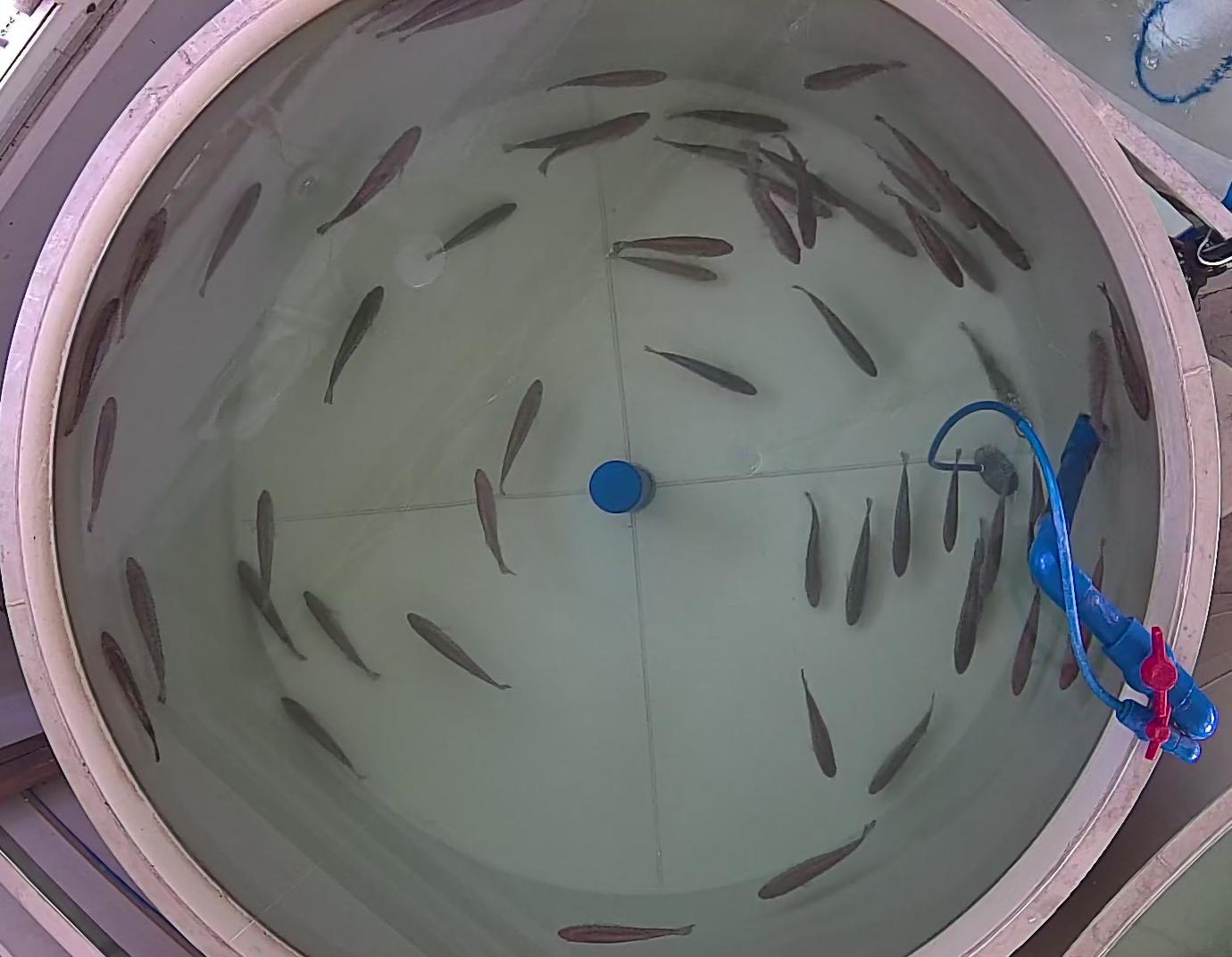

Supplement: S1 Dataset — (ZIP) [file pone.0283671.s001.zip › datasets/00117.jpg]

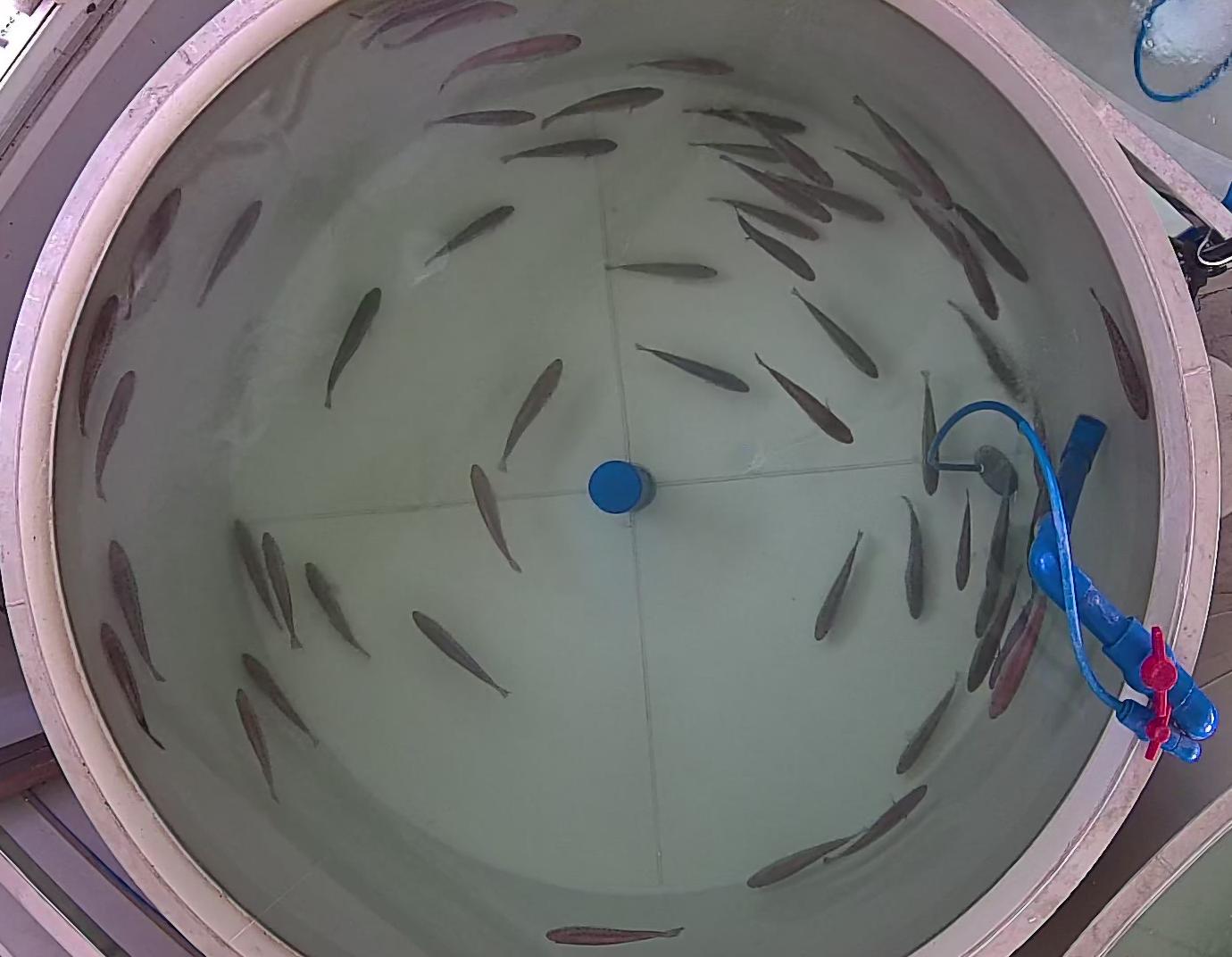

Supplement: S1 Dataset — (ZIP) [file pone.0283671.s001.zip › datasets/00118.jpg]

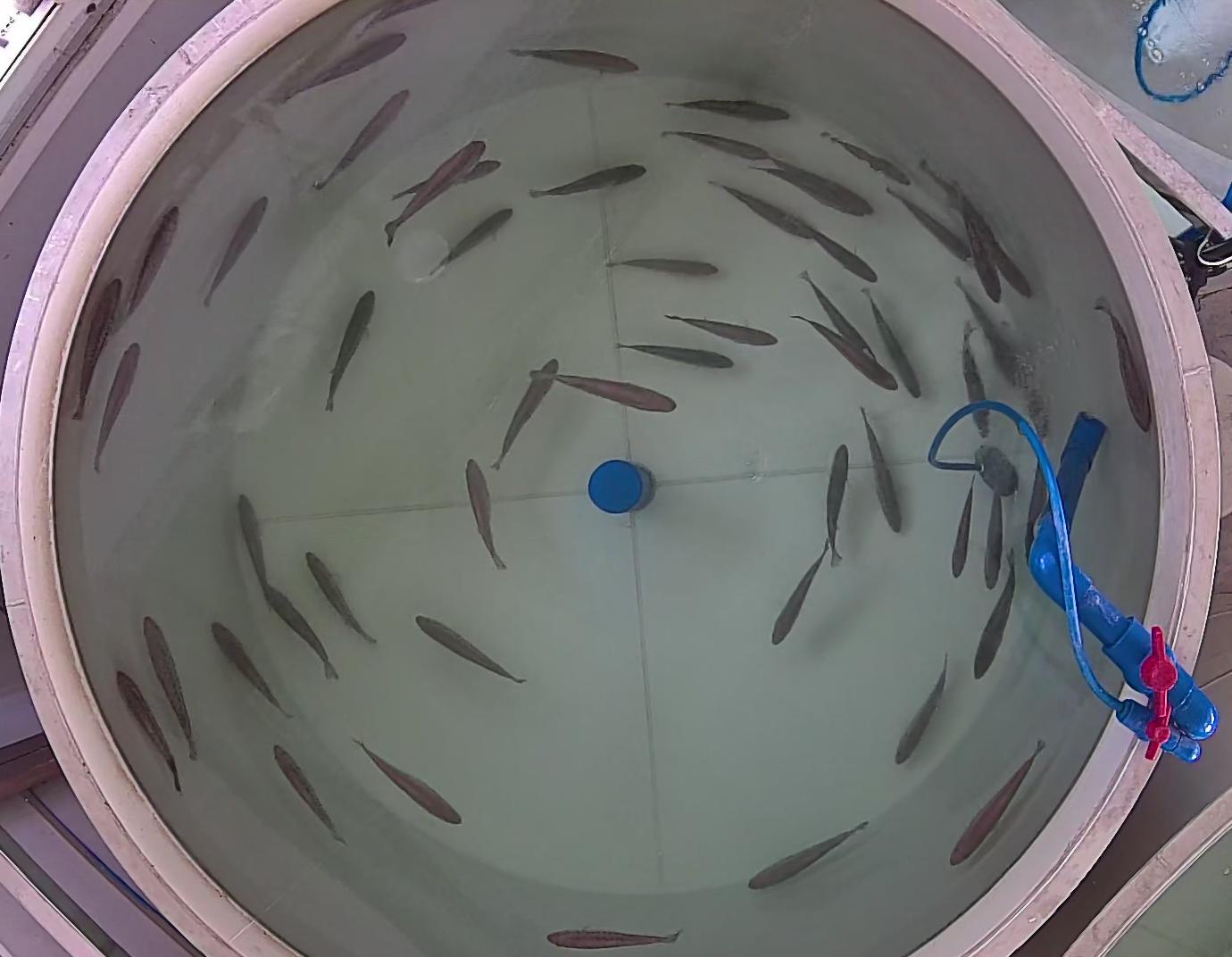

Supplement: S1 Dataset — (ZIP) [file pone.0283671.s001.zip › datasets/00119.jpg]

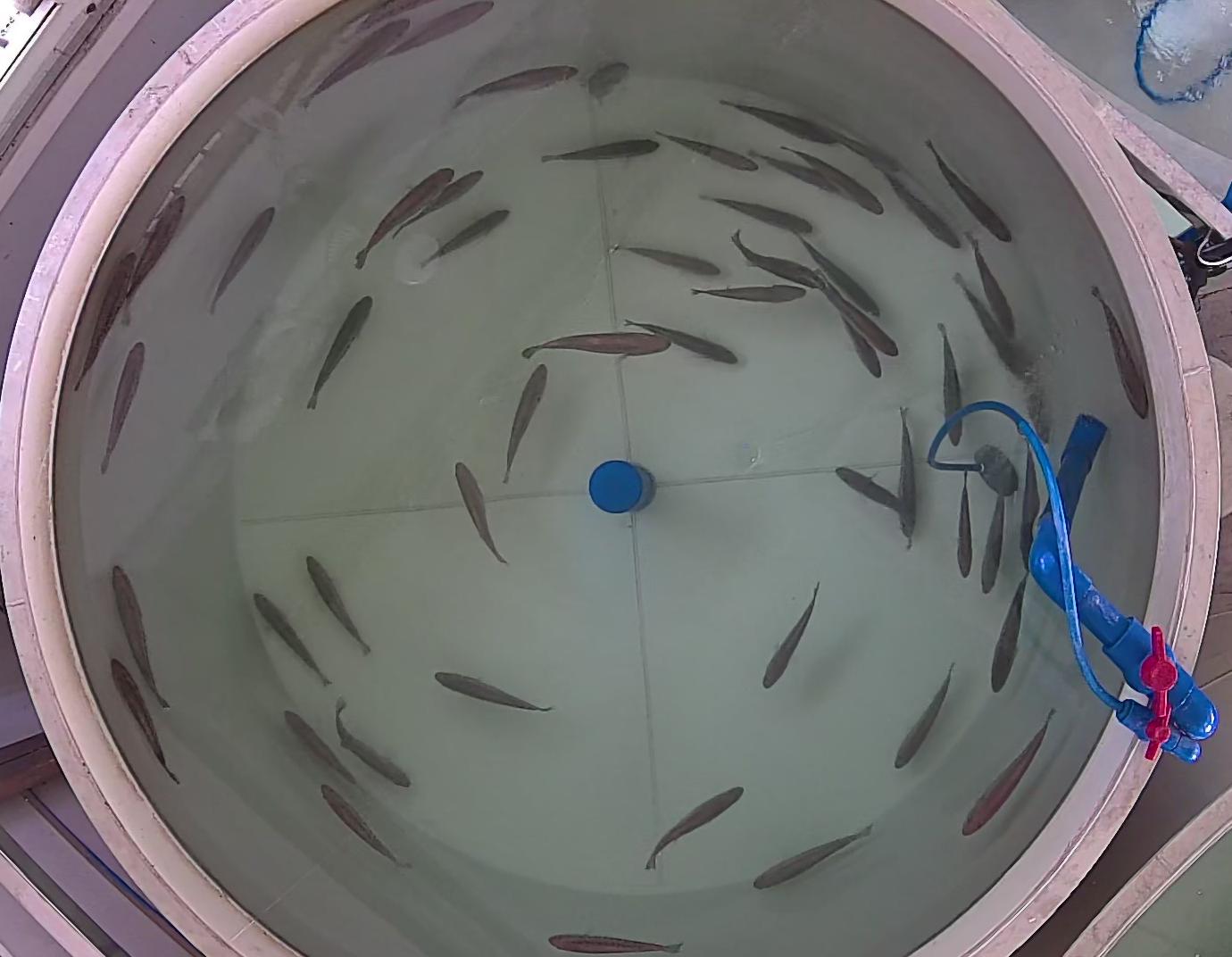

Supplement: S1 Dataset — (ZIP) [file pone.0283671.s001.zip › datasets/00120.jpg]
