# Supplementary figures and images for: Bass detection model based on improved YOLOv5 in circulating water system (part 1 of 2)
Source: PLoS One. 2023 Mar 27;18(3):e0283671. doi: 10.1371/journal.pone.0283671 (PMC10042332; doi:10.1371/journal.pone.0283671)

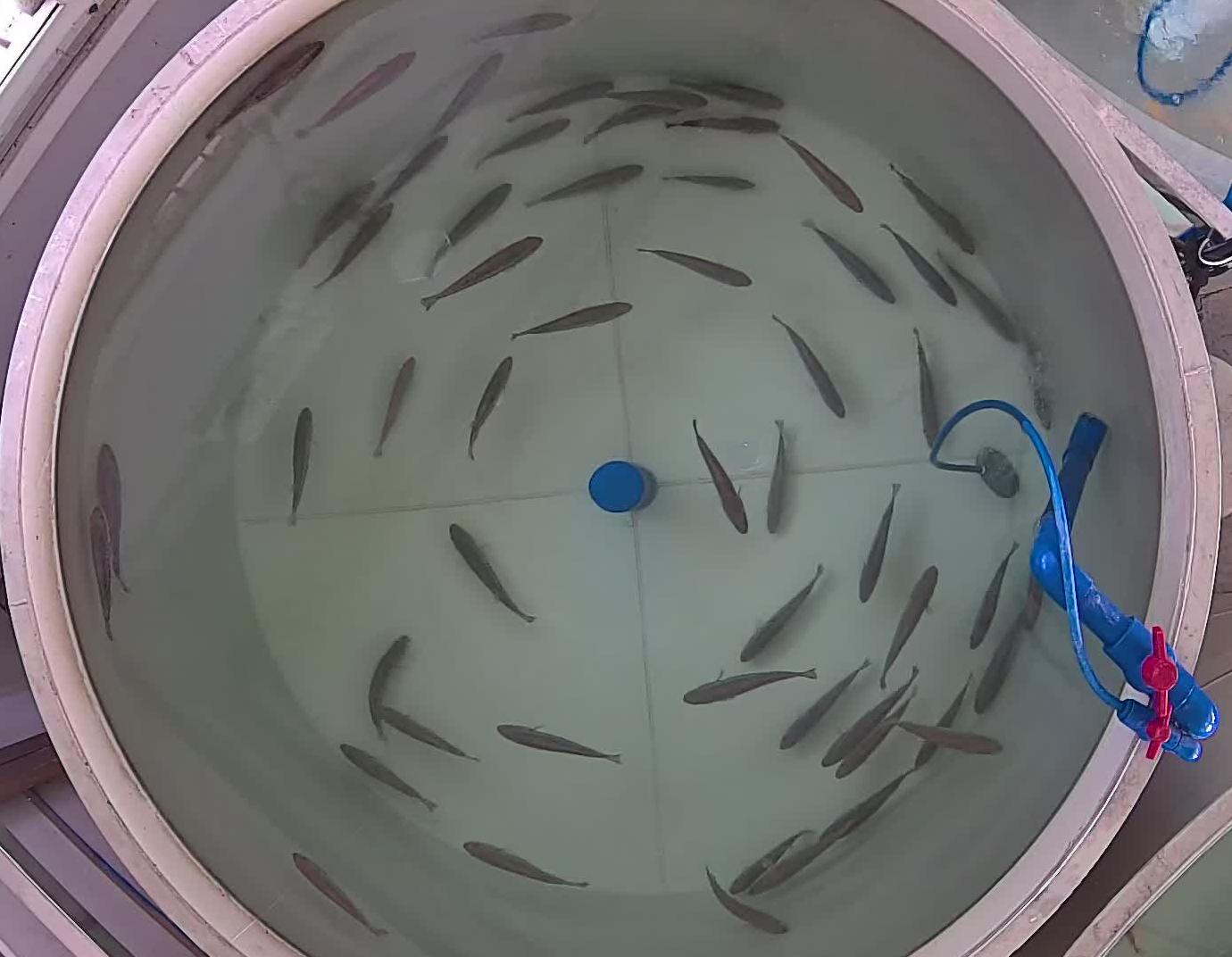

Supplement: S1 Dataset — (ZIP) [file pone.0283671.s001.zip › datasets/00001.jpg]

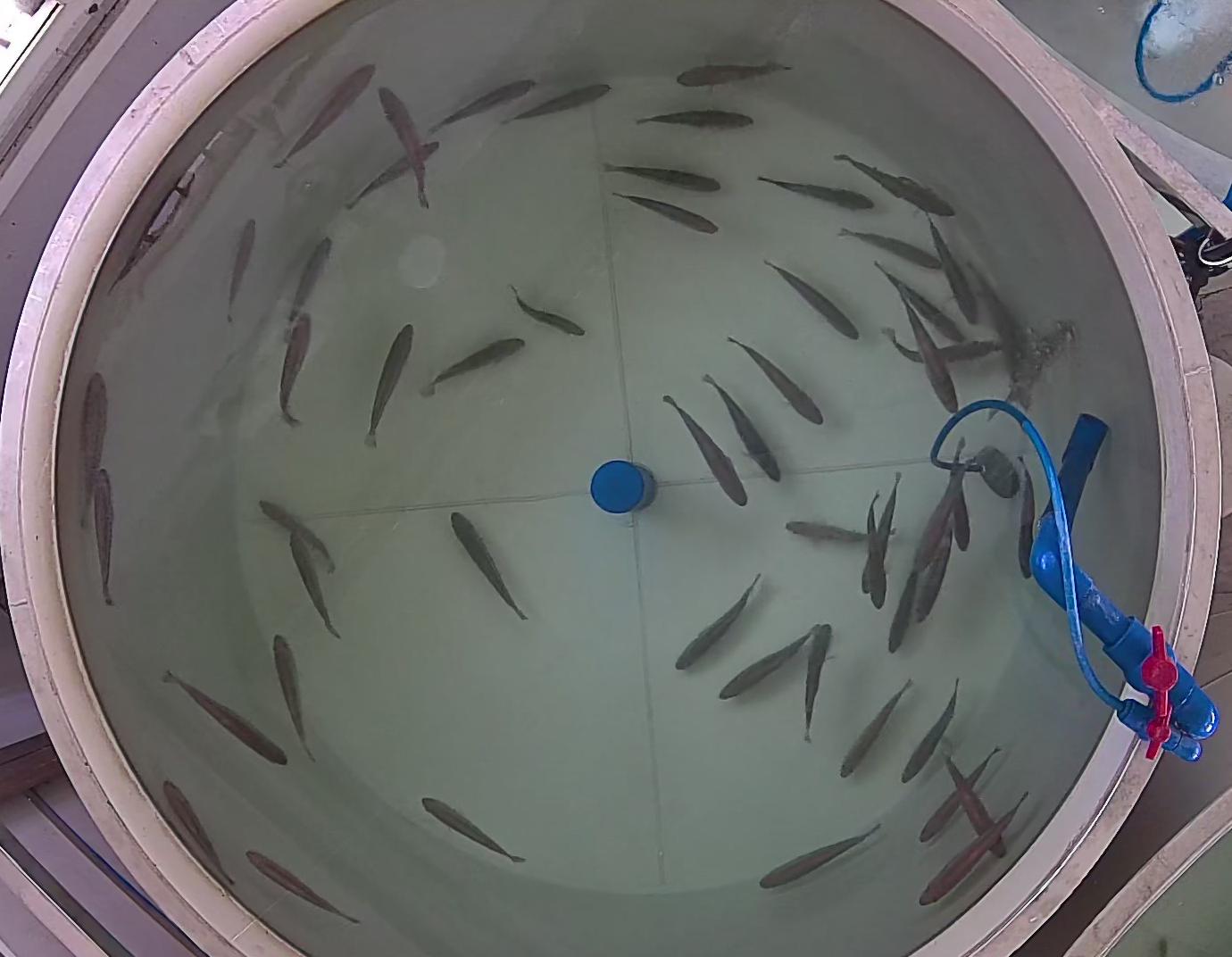

Supplement: S1 Dataset — (ZIP) [file pone.0283671.s001.zip › datasets/00002.jpg]

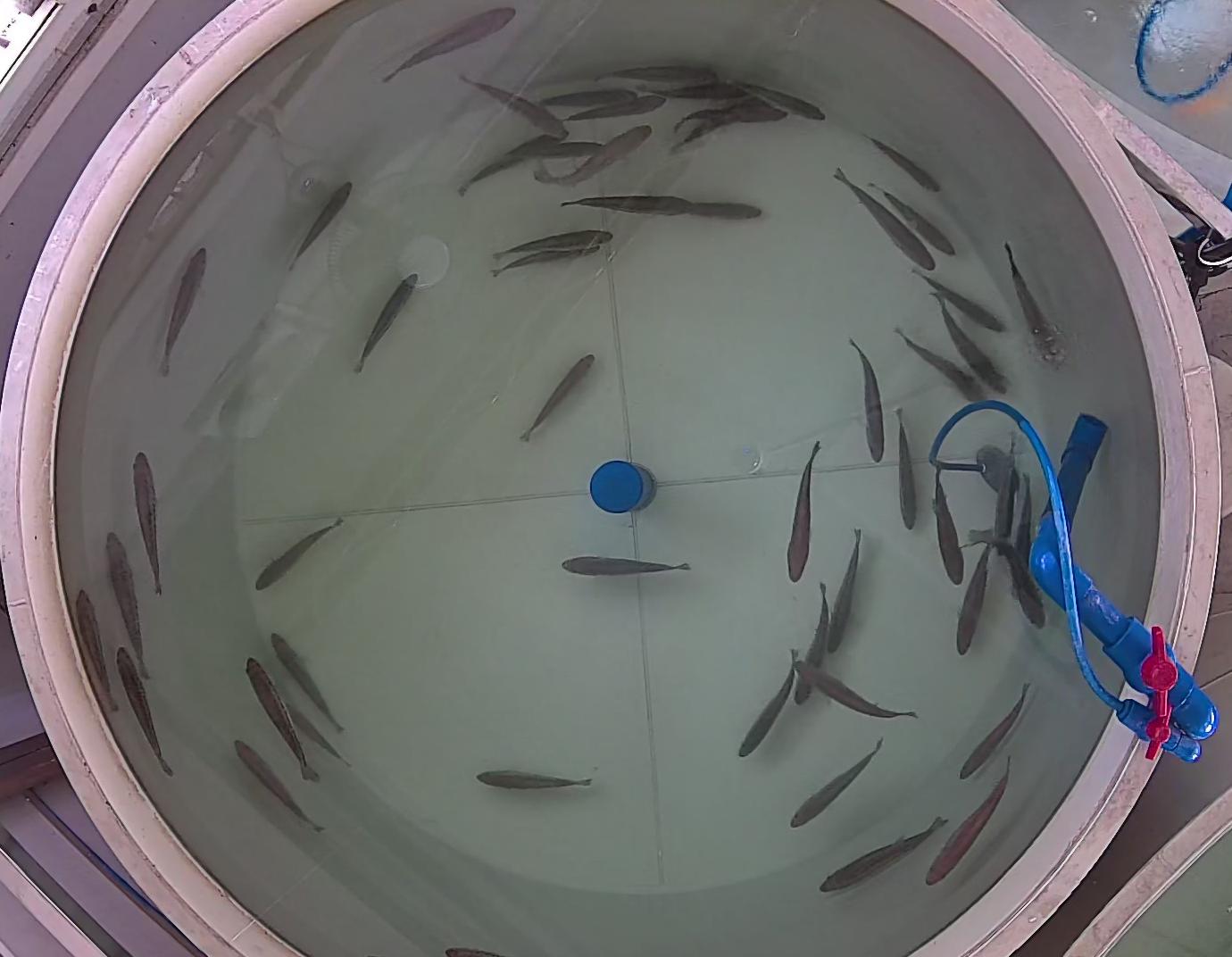

Supplement: S1 Dataset — (ZIP) [file pone.0283671.s001.zip › datasets/00003.jpg]

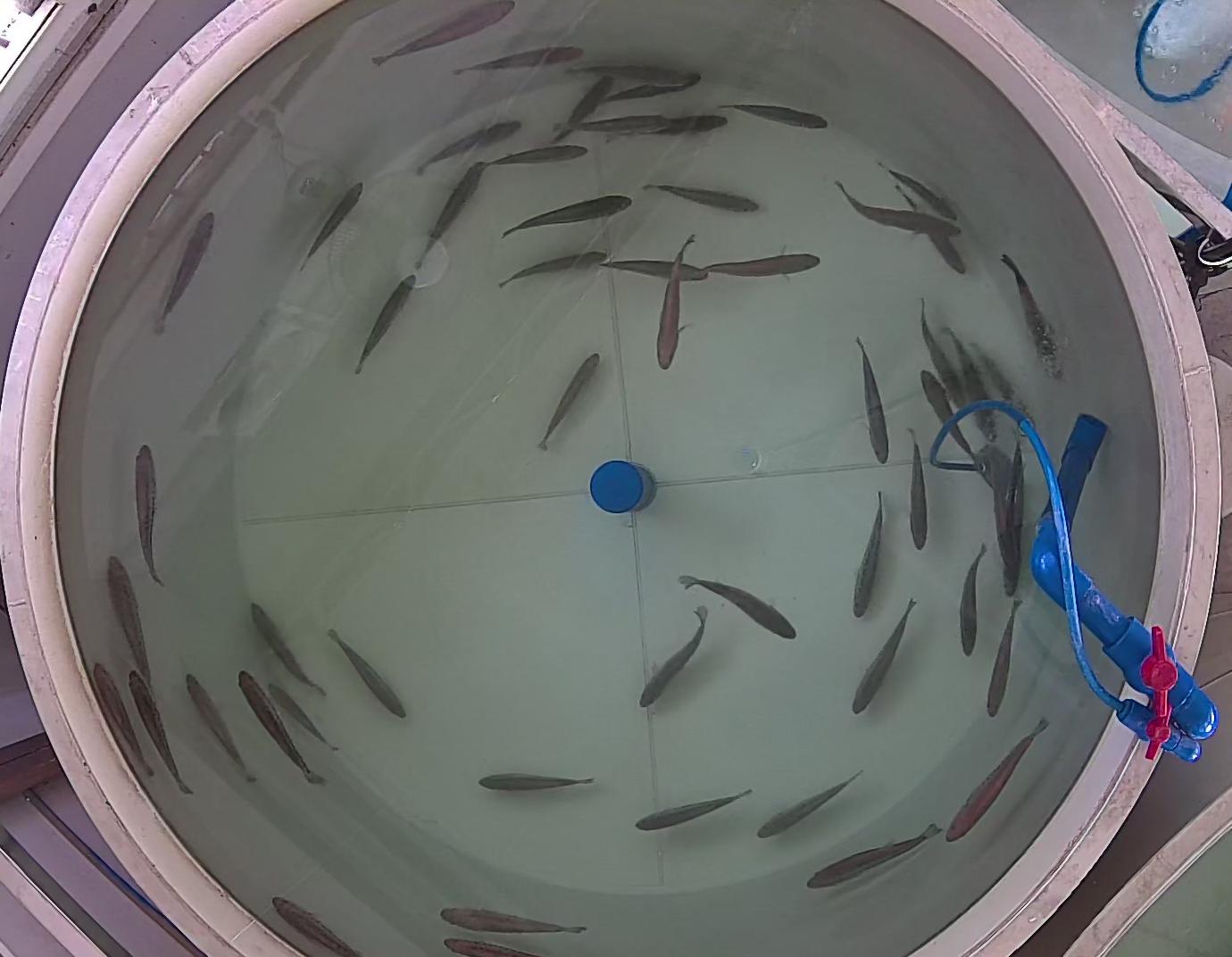

Supplement: S1 Dataset — (ZIP) [file pone.0283671.s001.zip › datasets/00004.jpg]

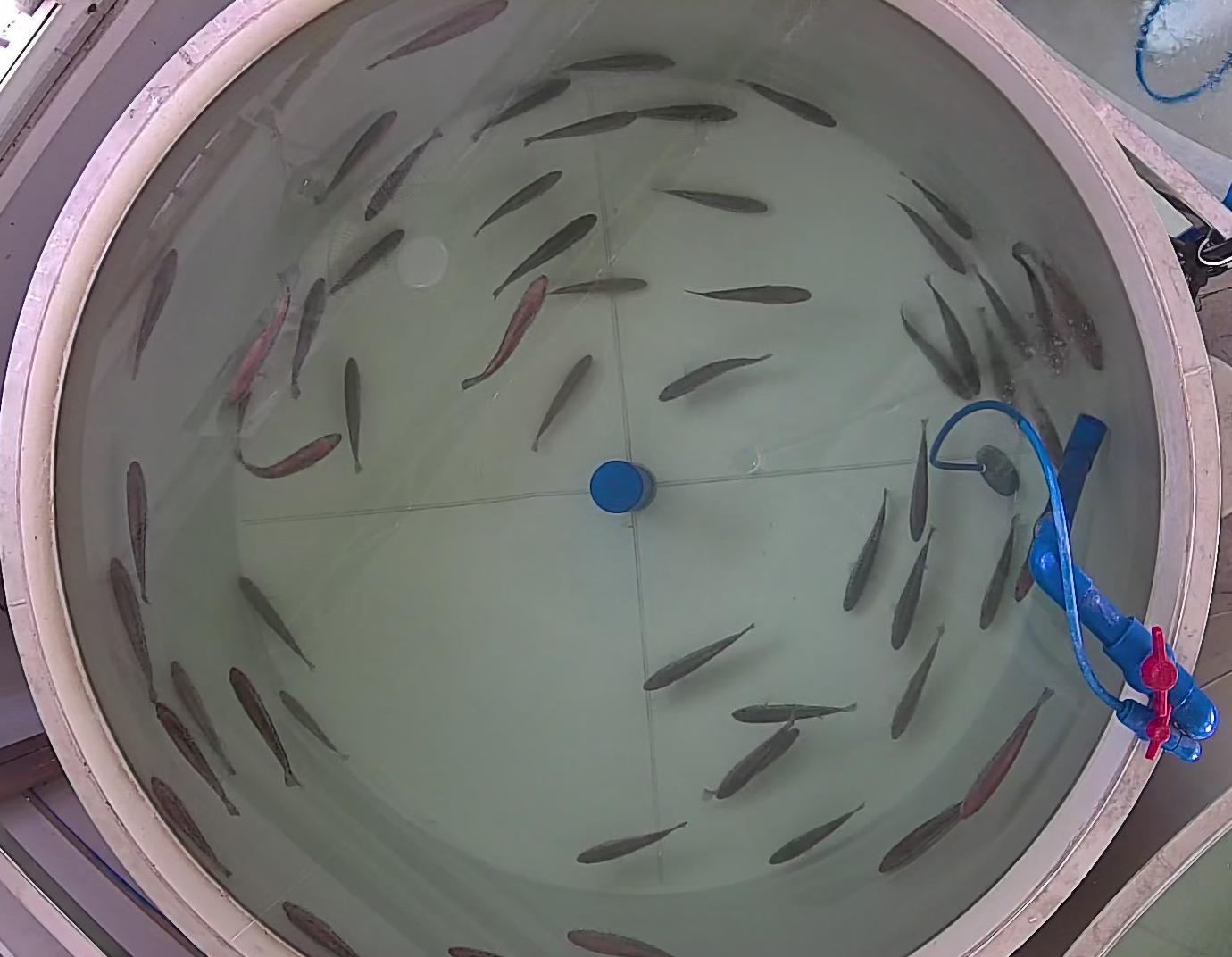

Supplement: S1 Dataset — (ZIP) [file pone.0283671.s001.zip › datasets/00005.jpg]

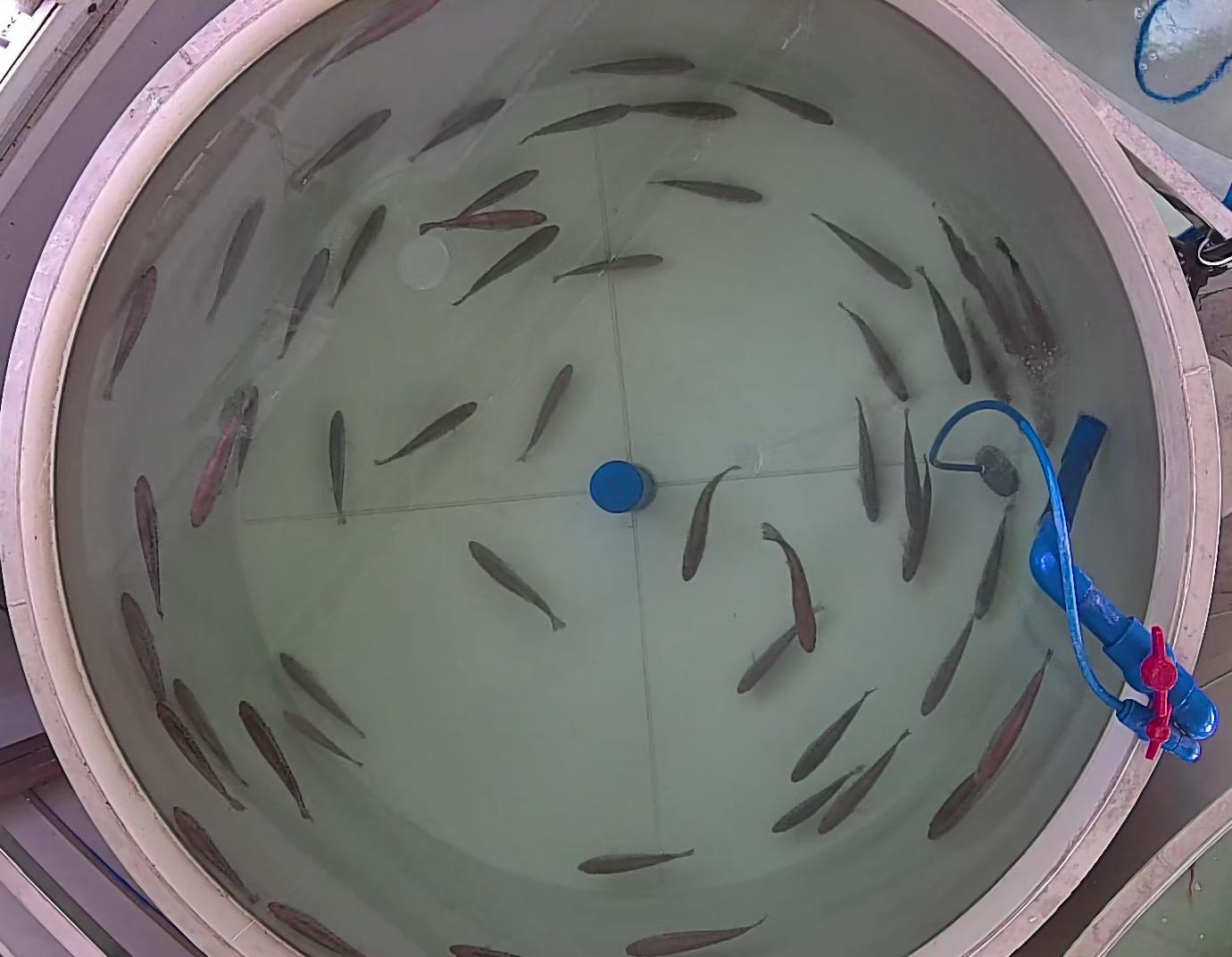

Supplement: S1 Dataset — (ZIP) [file pone.0283671.s001.zip › datasets/00006.jpg]

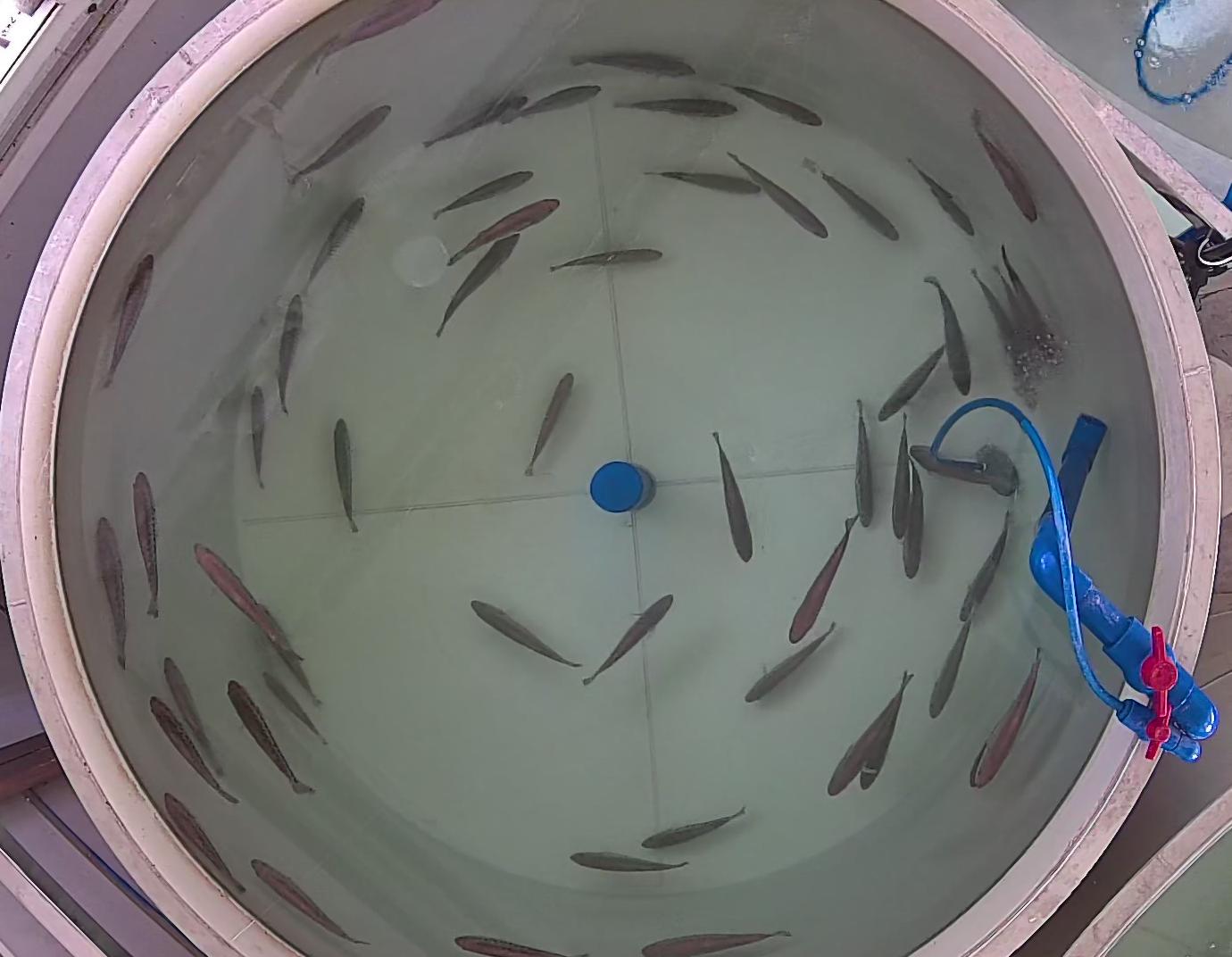

Supplement: S1 Dataset — (ZIP) [file pone.0283671.s001.zip › datasets/00007.jpg]

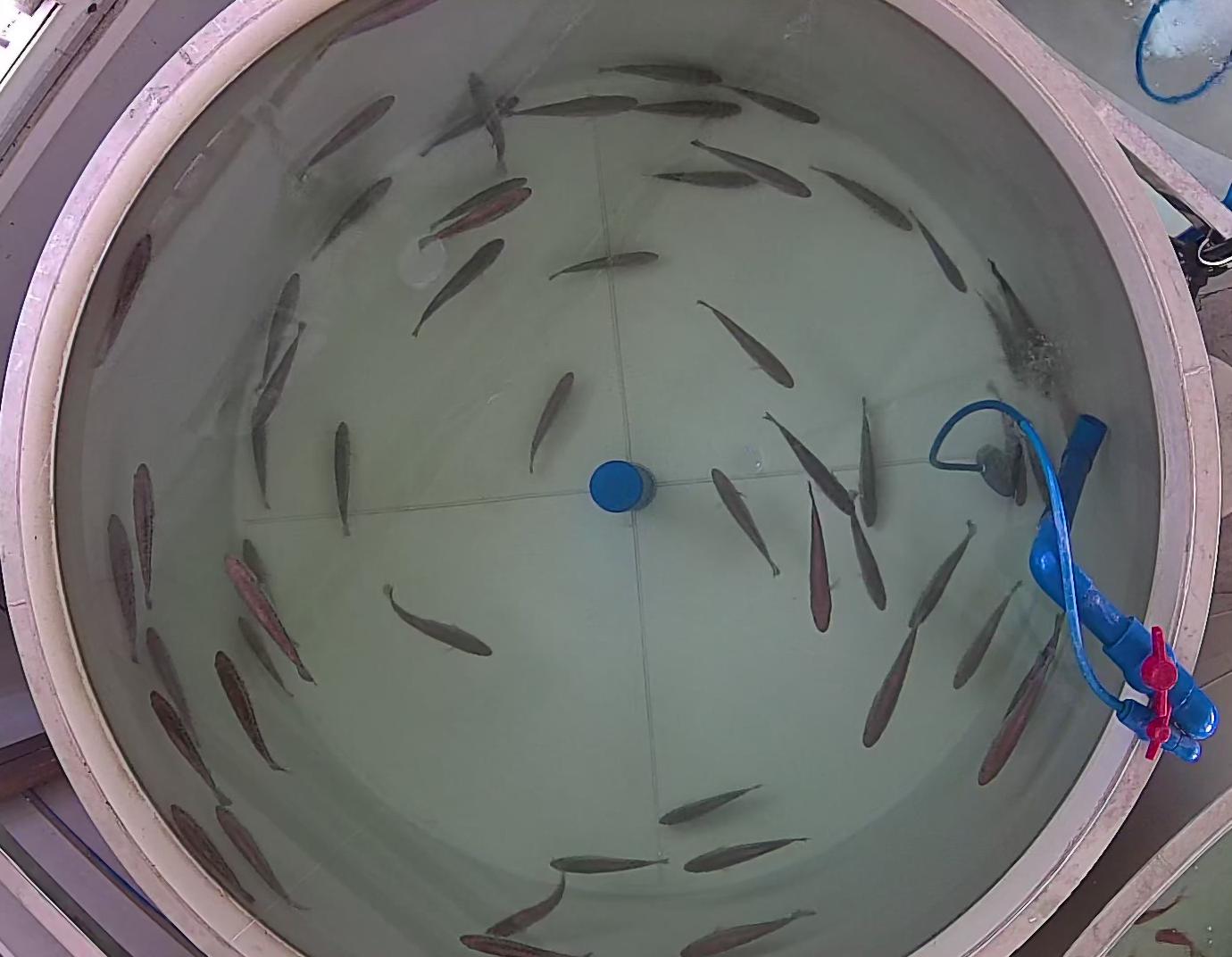

Supplement: S1 Dataset — (ZIP) [file pone.0283671.s001.zip › datasets/00008.jpg]

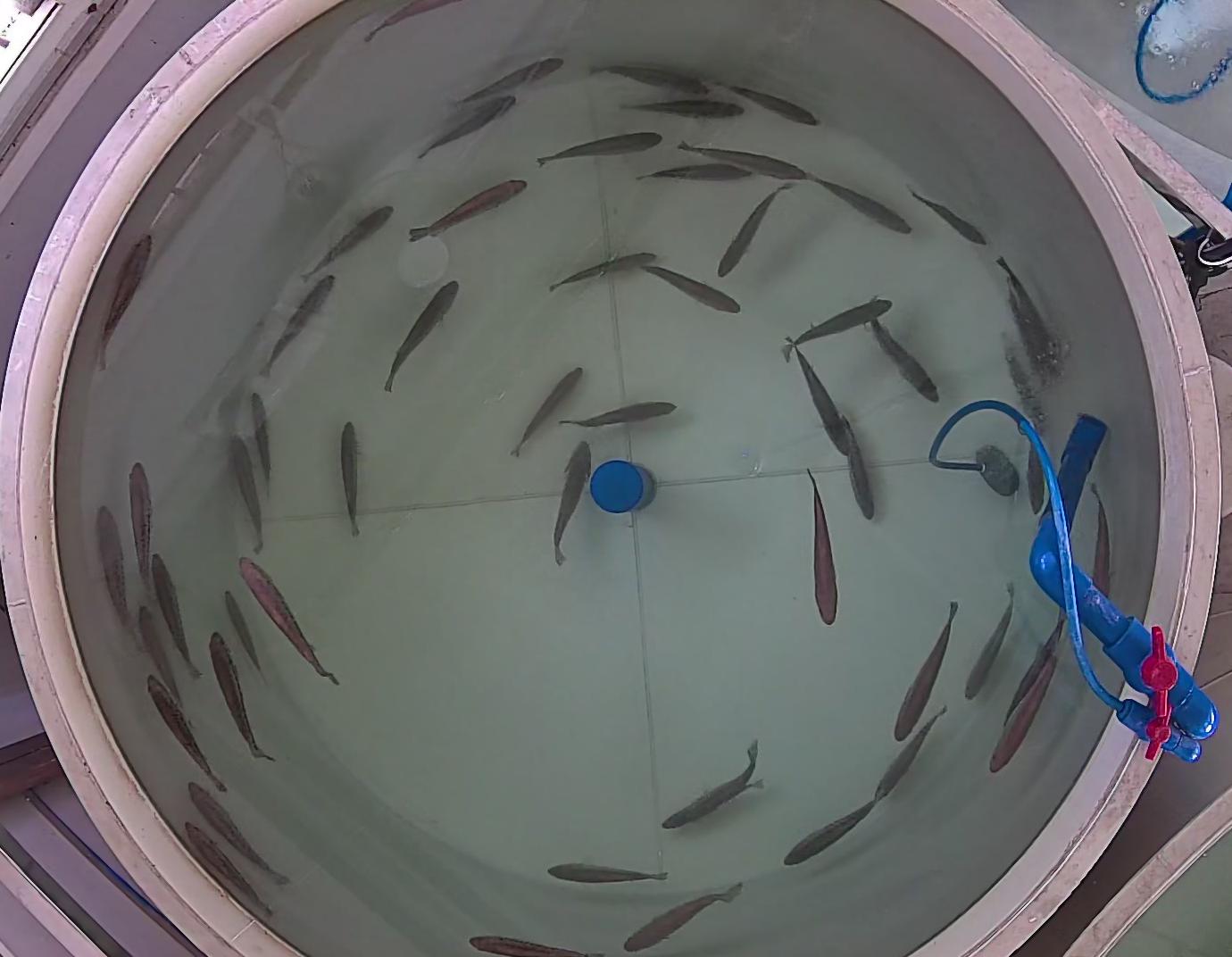

Supplement: S1 Dataset — (ZIP) [file pone.0283671.s001.zip › datasets/00009.jpg]

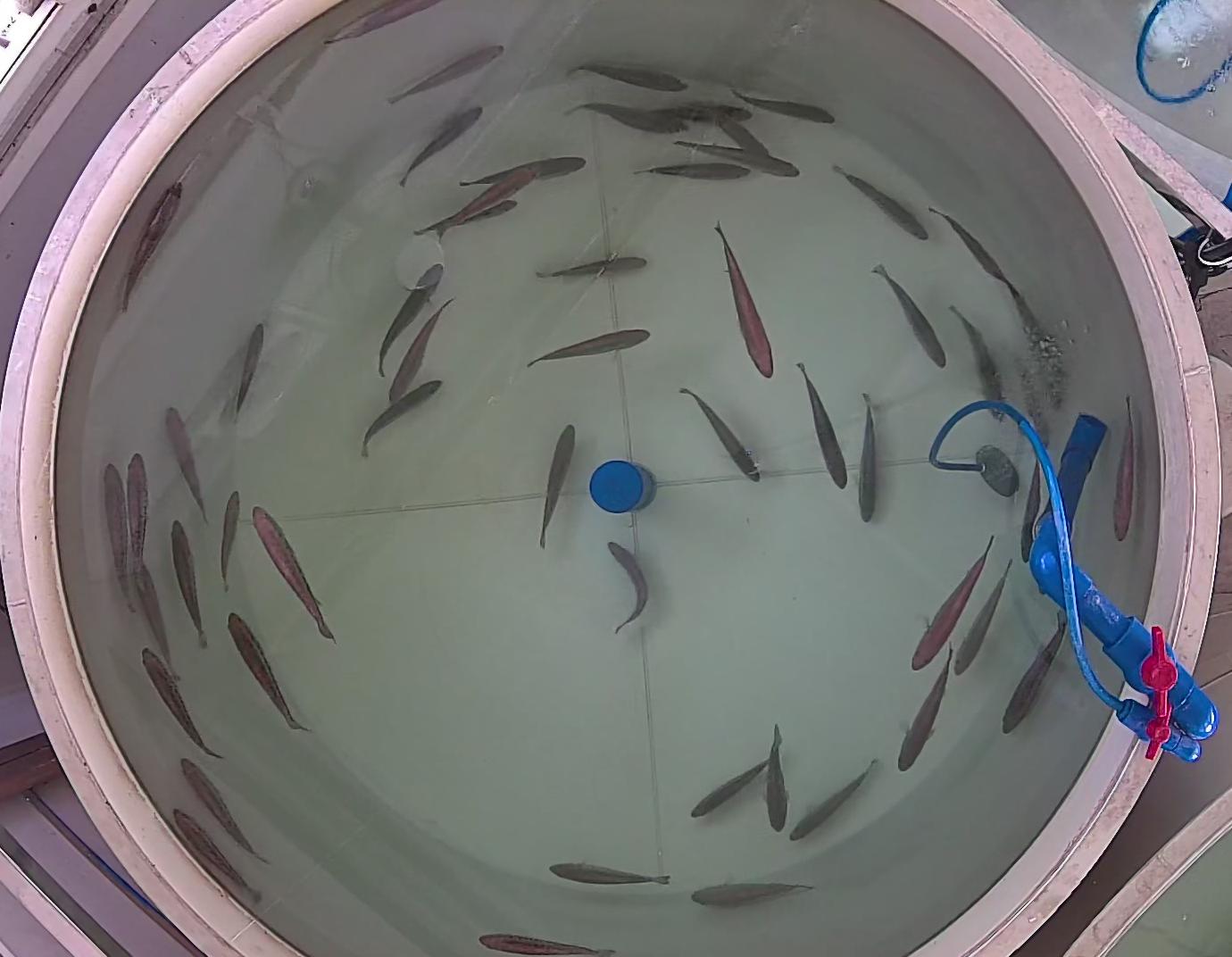

Supplement: S1 Dataset — (ZIP) [file pone.0283671.s001.zip › datasets/00010.jpg]

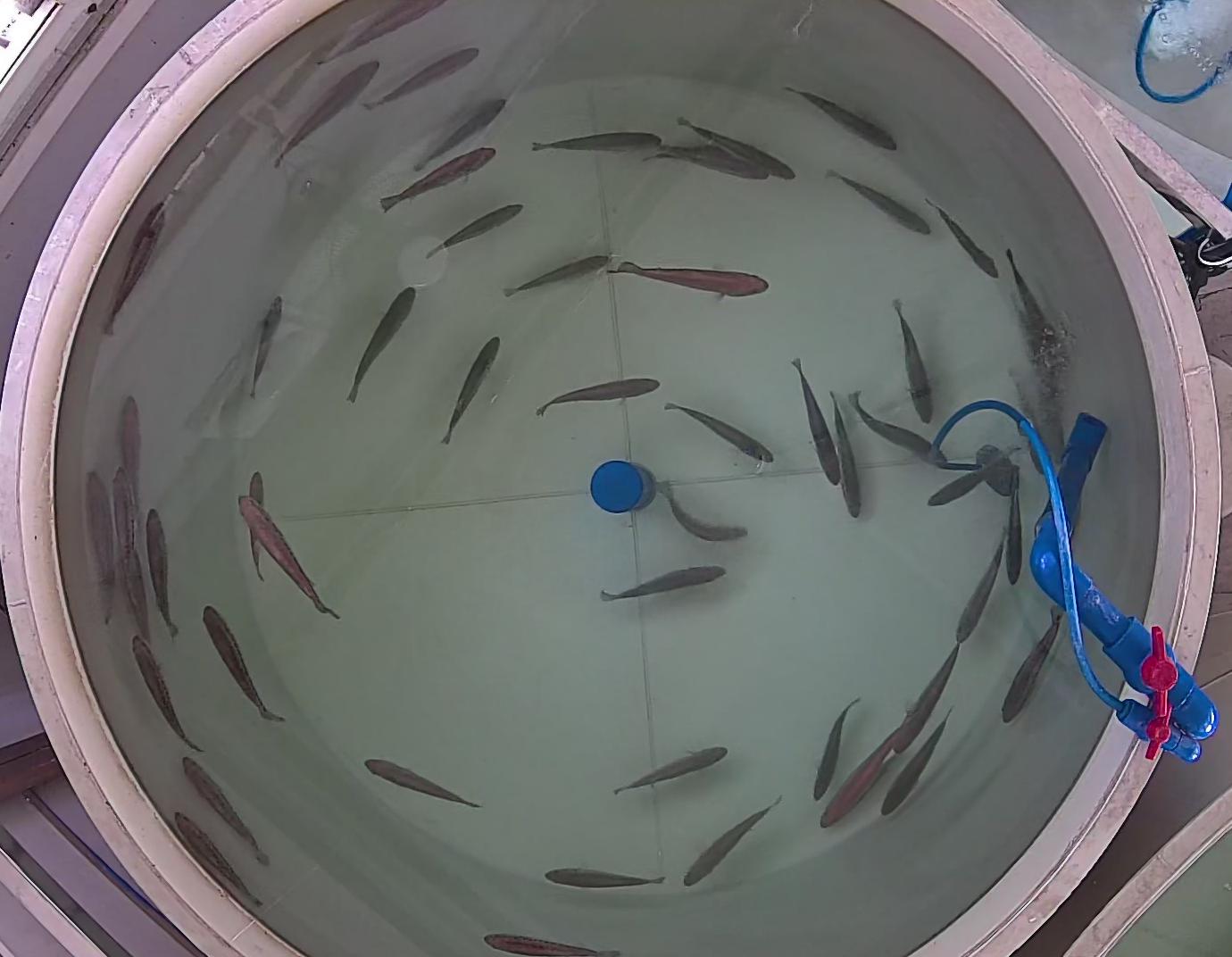

Supplement: S1 Dataset — (ZIP) [file pone.0283671.s001.zip › datasets/00011.jpg]

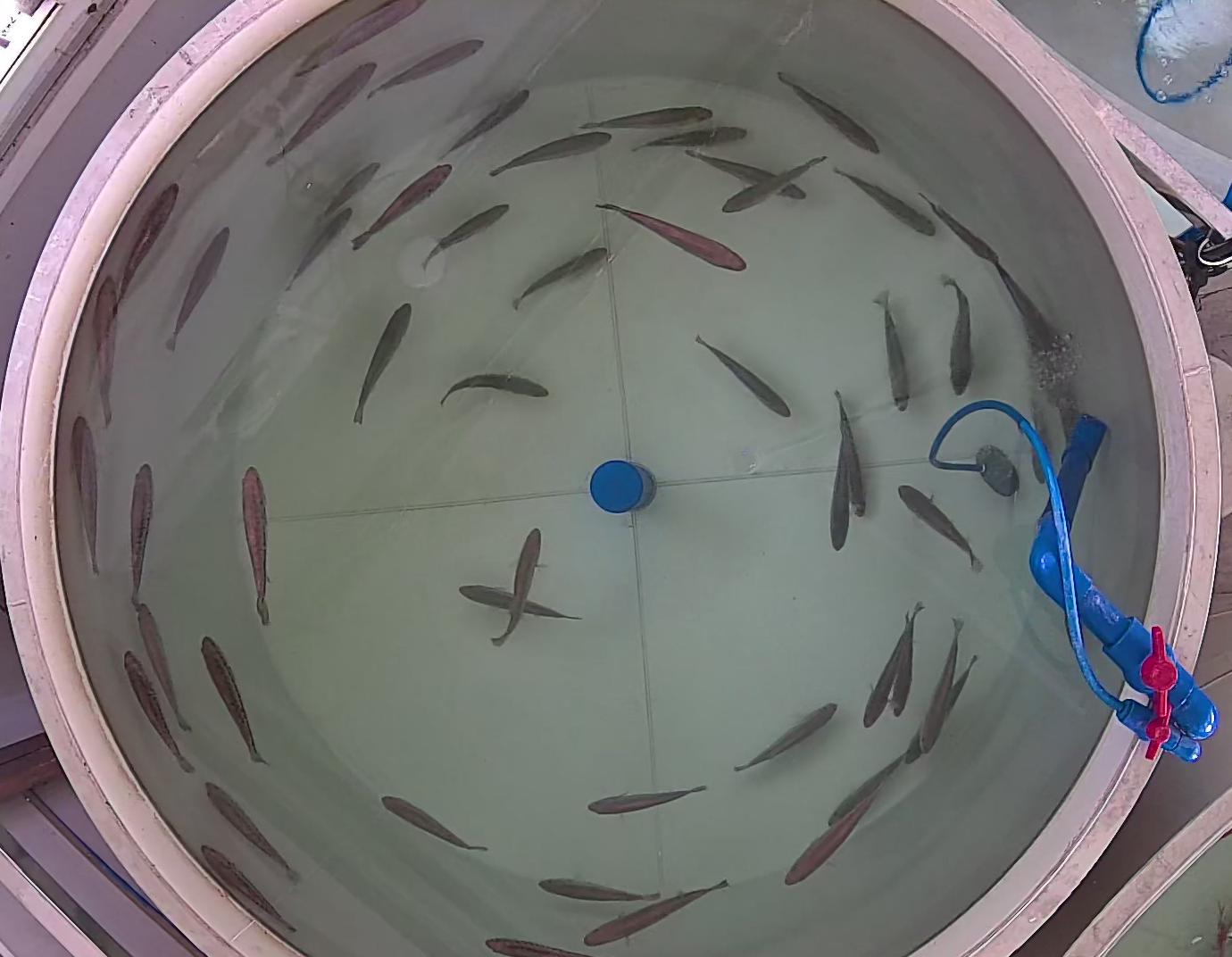

Supplement: S1 Dataset — (ZIP) [file pone.0283671.s001.zip › datasets/00012.jpg]

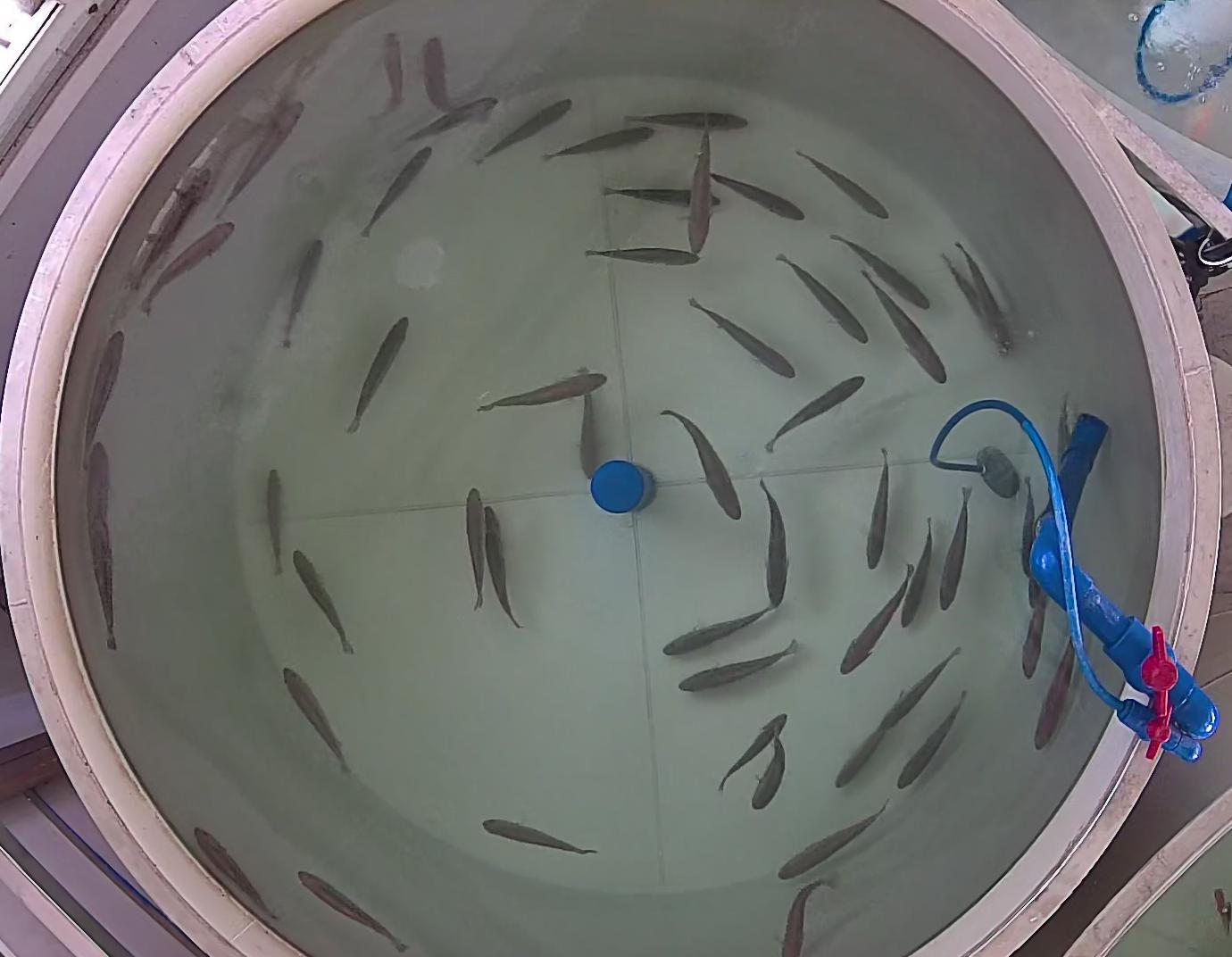

Supplement: S1 Dataset — (ZIP) [file pone.0283671.s001.zip › datasets/00013.jpg]

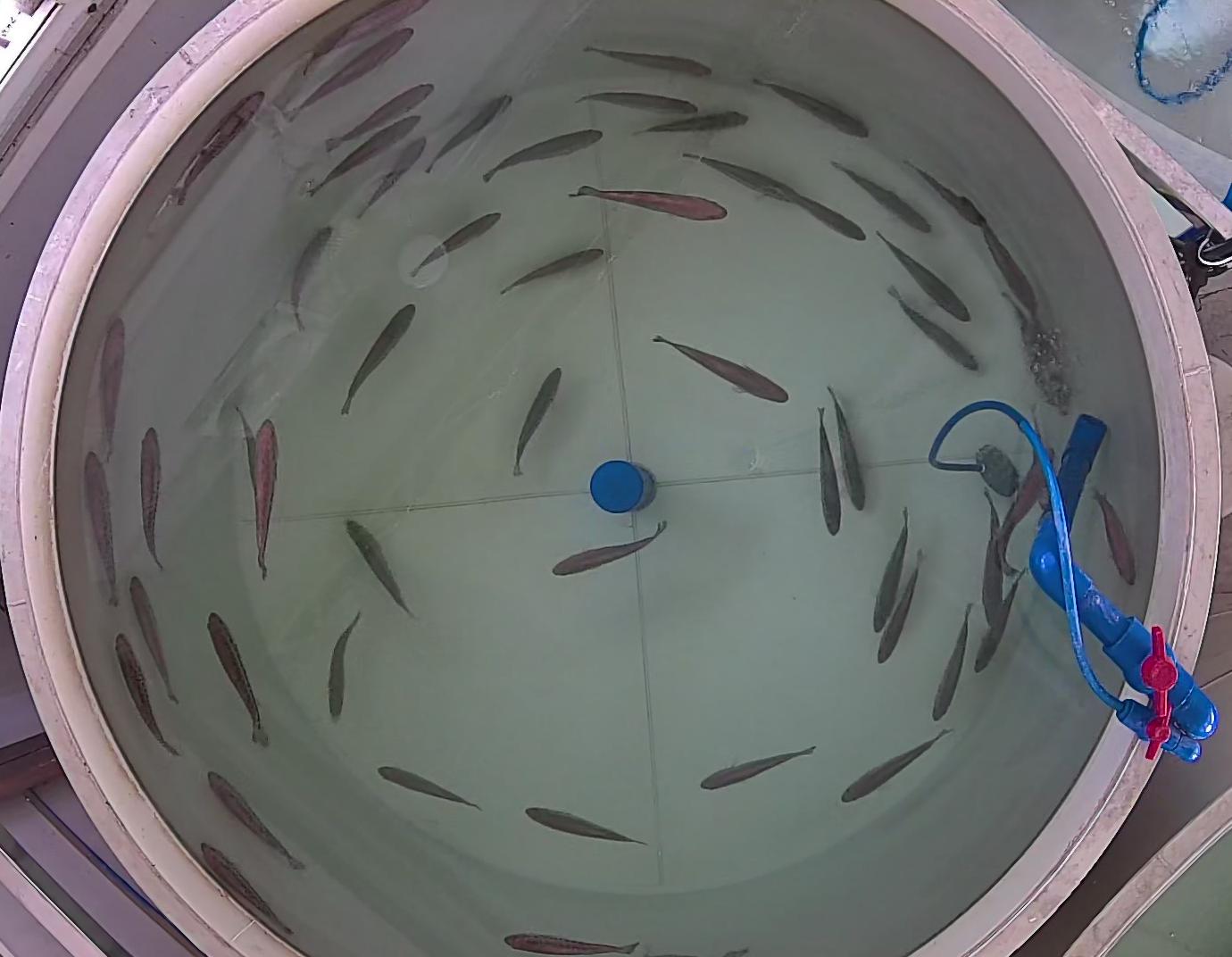

Supplement: S1 Dataset — (ZIP) [file pone.0283671.s001.zip › datasets/00014.jpg]

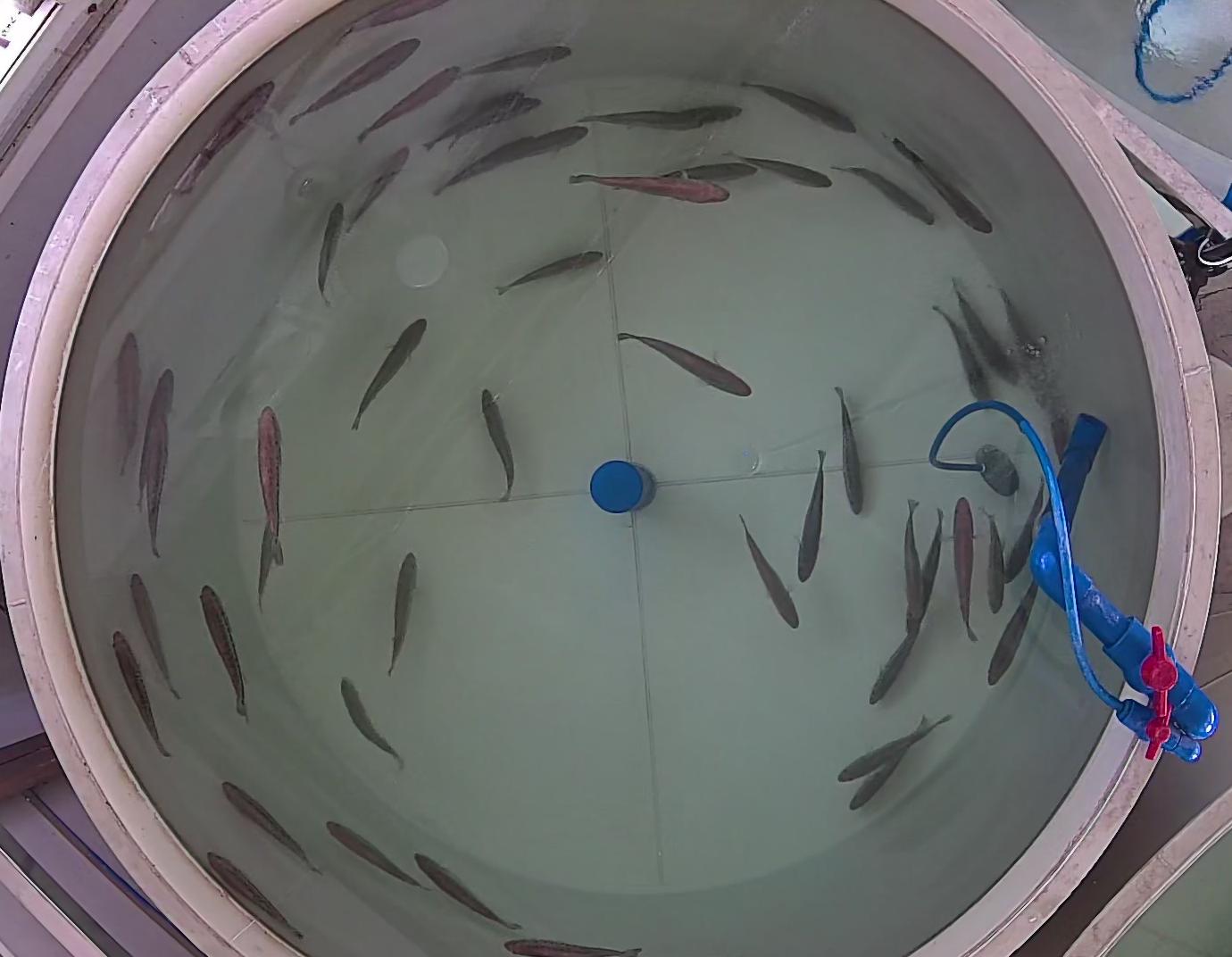

Supplement: S1 Dataset — (ZIP) [file pone.0283671.s001.zip › datasets/00015.jpg]

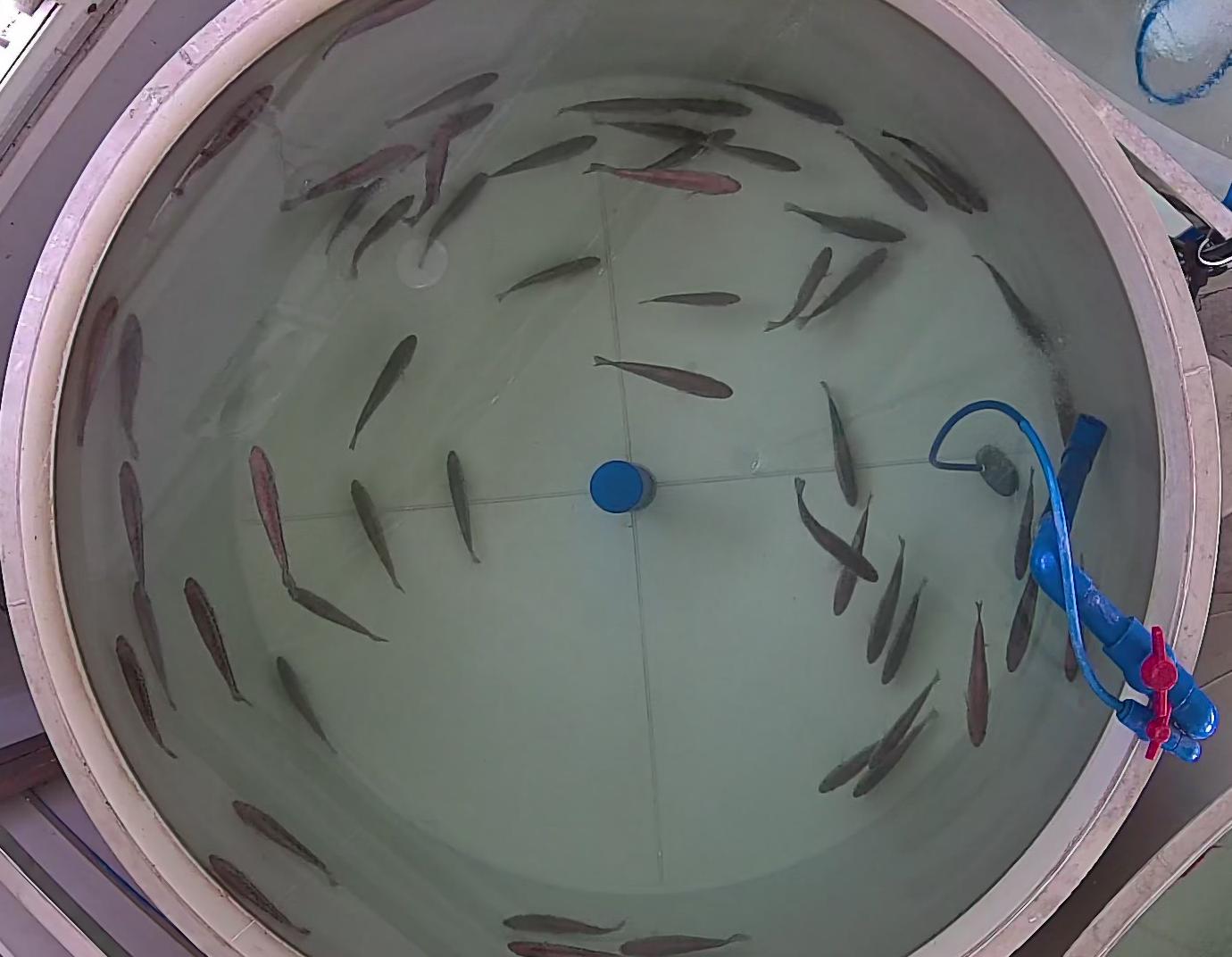

Supplement: S1 Dataset — (ZIP) [file pone.0283671.s001.zip › datasets/00016.jpg]

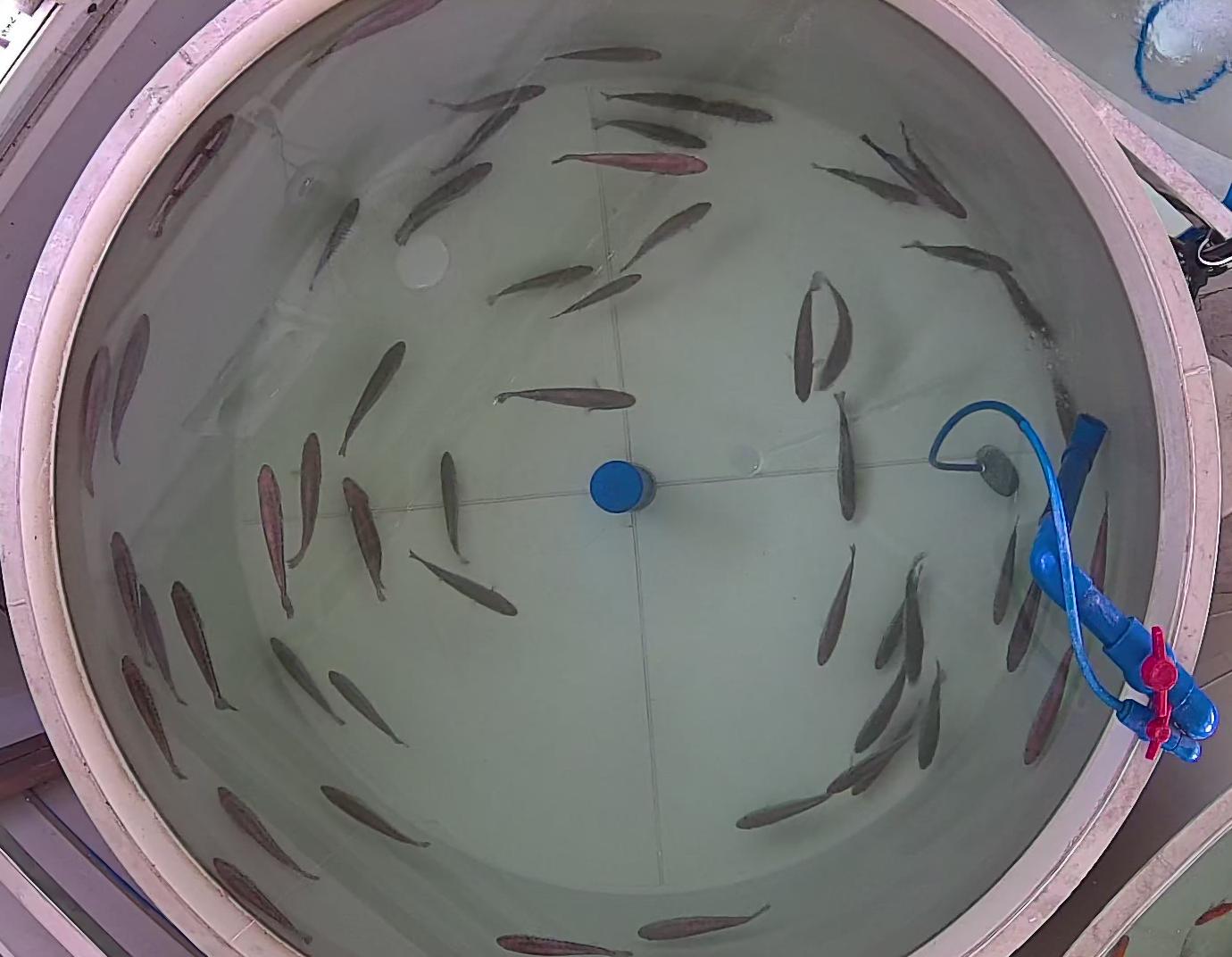

Supplement: S1 Dataset — (ZIP) [file pone.0283671.s001.zip › datasets/00017.jpg]

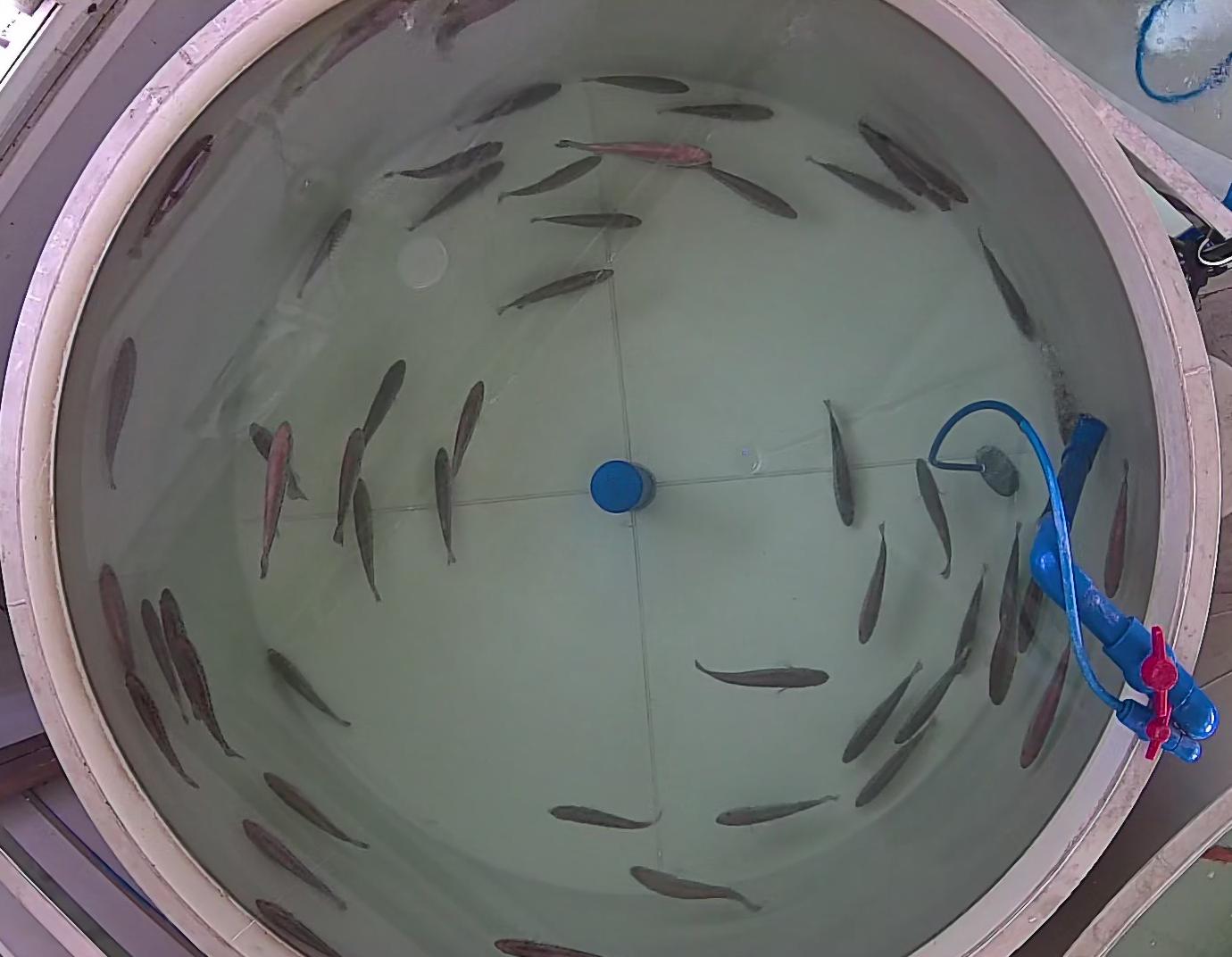

Supplement: S1 Dataset — (ZIP) [file pone.0283671.s001.zip › datasets/00018.jpg]

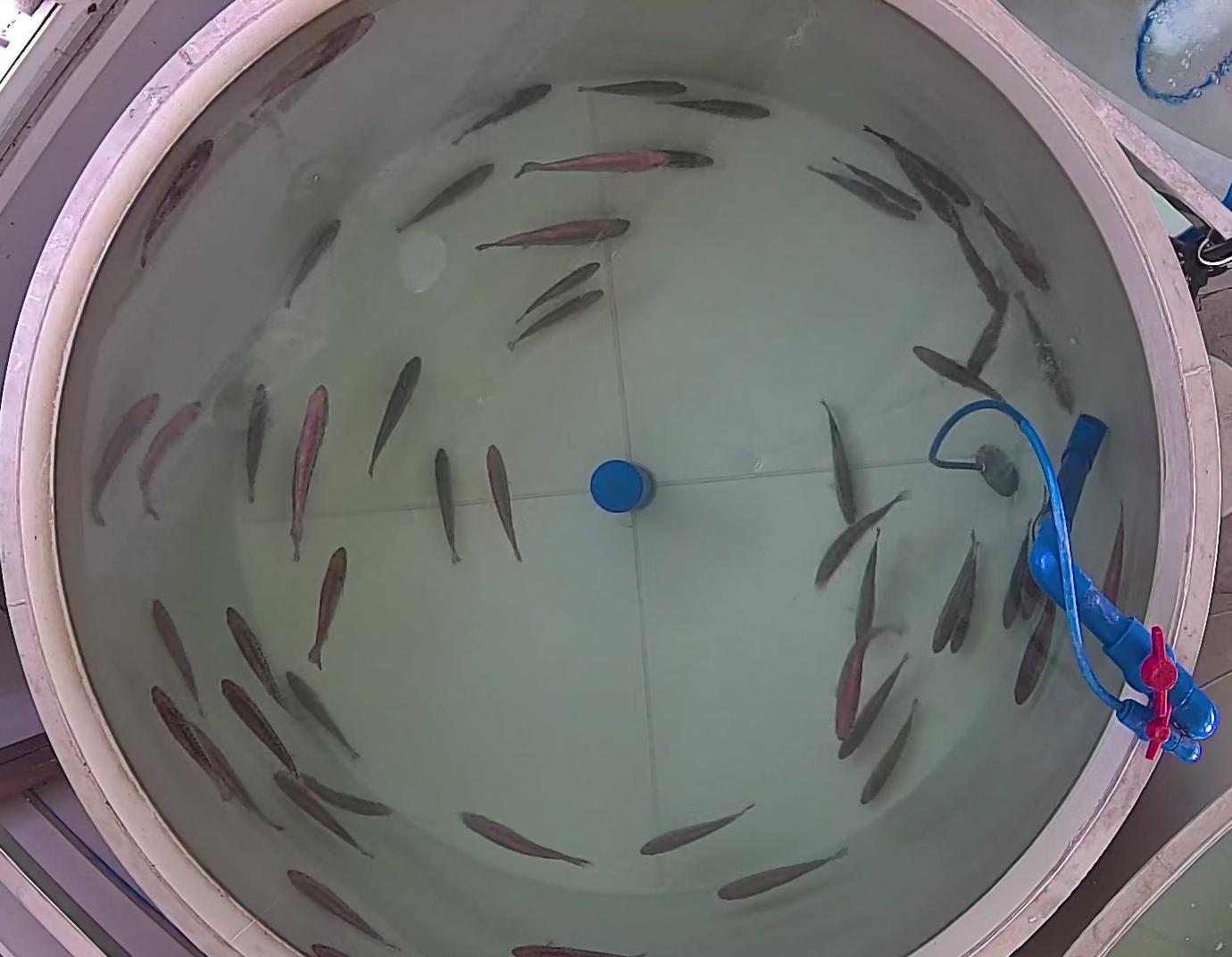

Supplement: S1 Dataset — (ZIP) [file pone.0283671.s001.zip › datasets/00019.jpg]

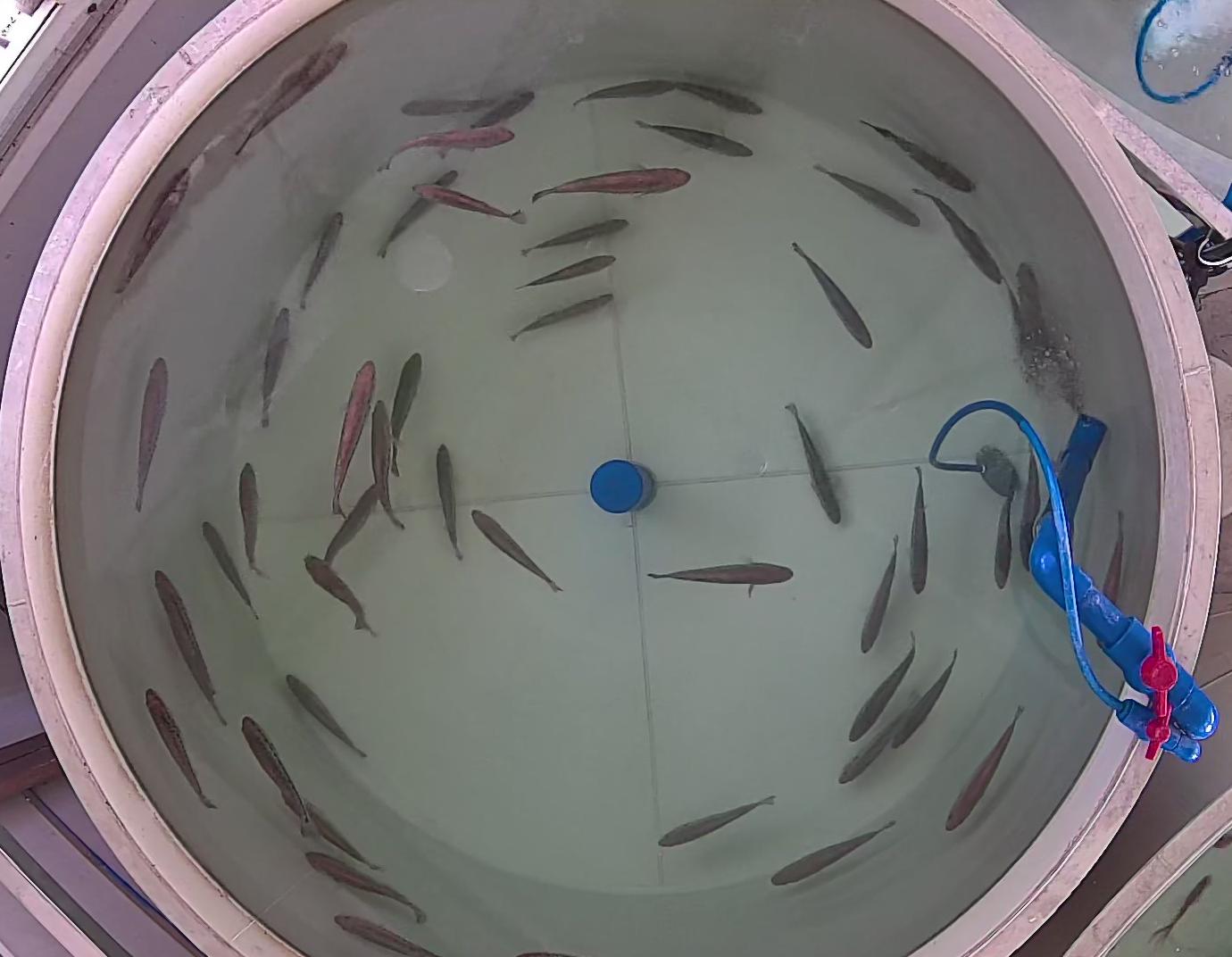

Supplement: S1 Dataset — (ZIP) [file pone.0283671.s001.zip › datasets/00020.jpg]

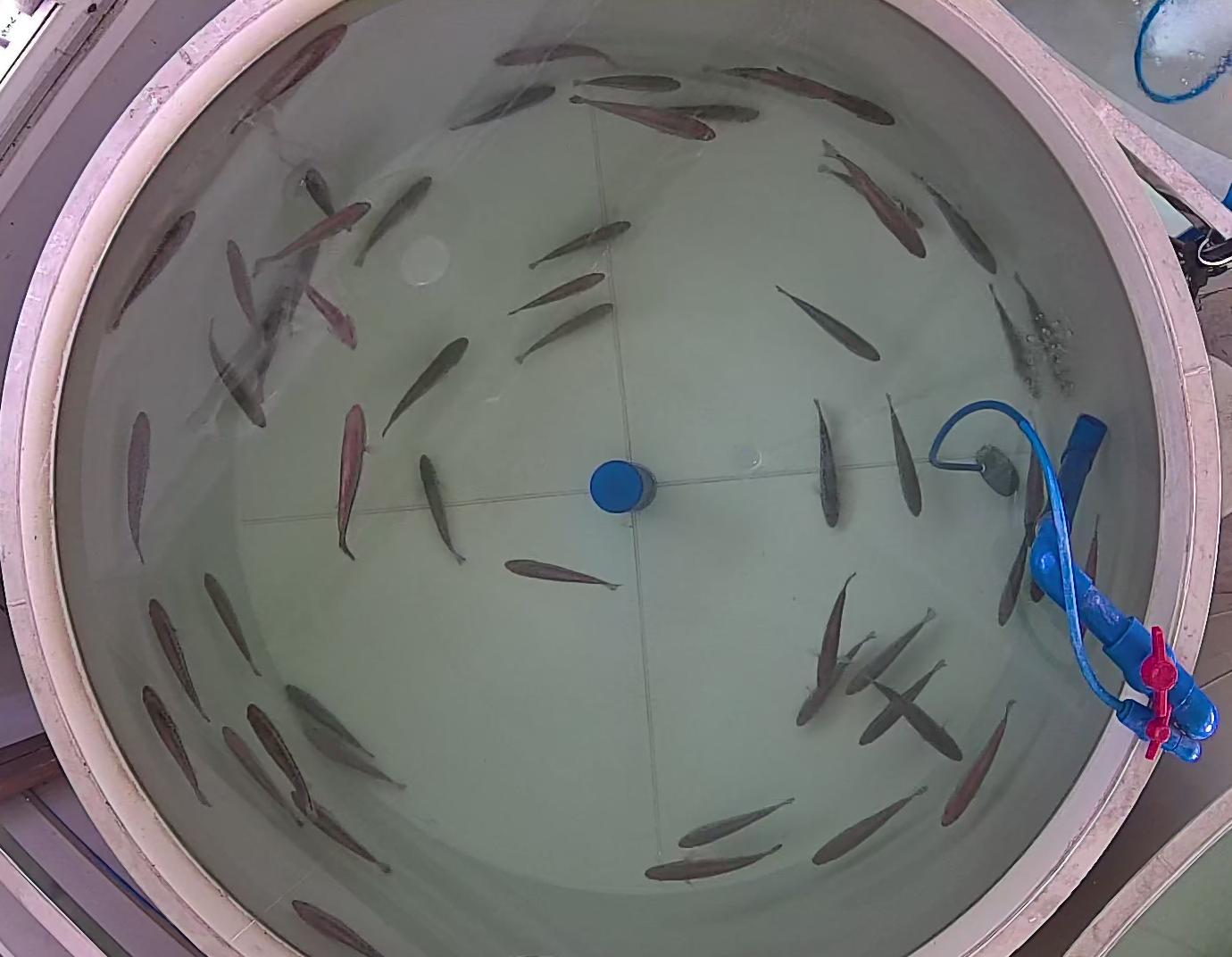

Supplement: S1 Dataset — (ZIP) [file pone.0283671.s001.zip › datasets/00021.jpg]

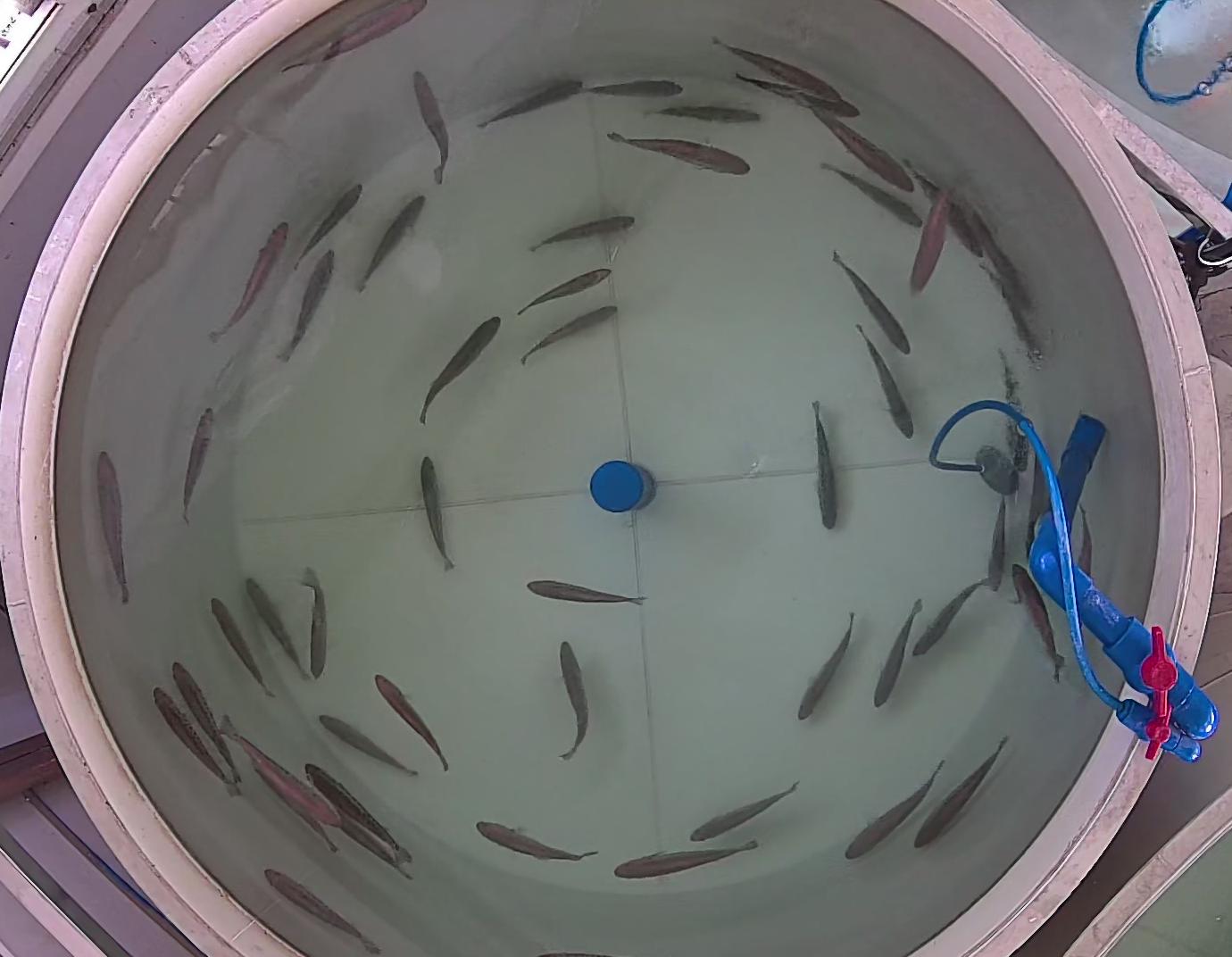

Supplement: S1 Dataset — (ZIP) [file pone.0283671.s001.zip › datasets/00022.jpg]

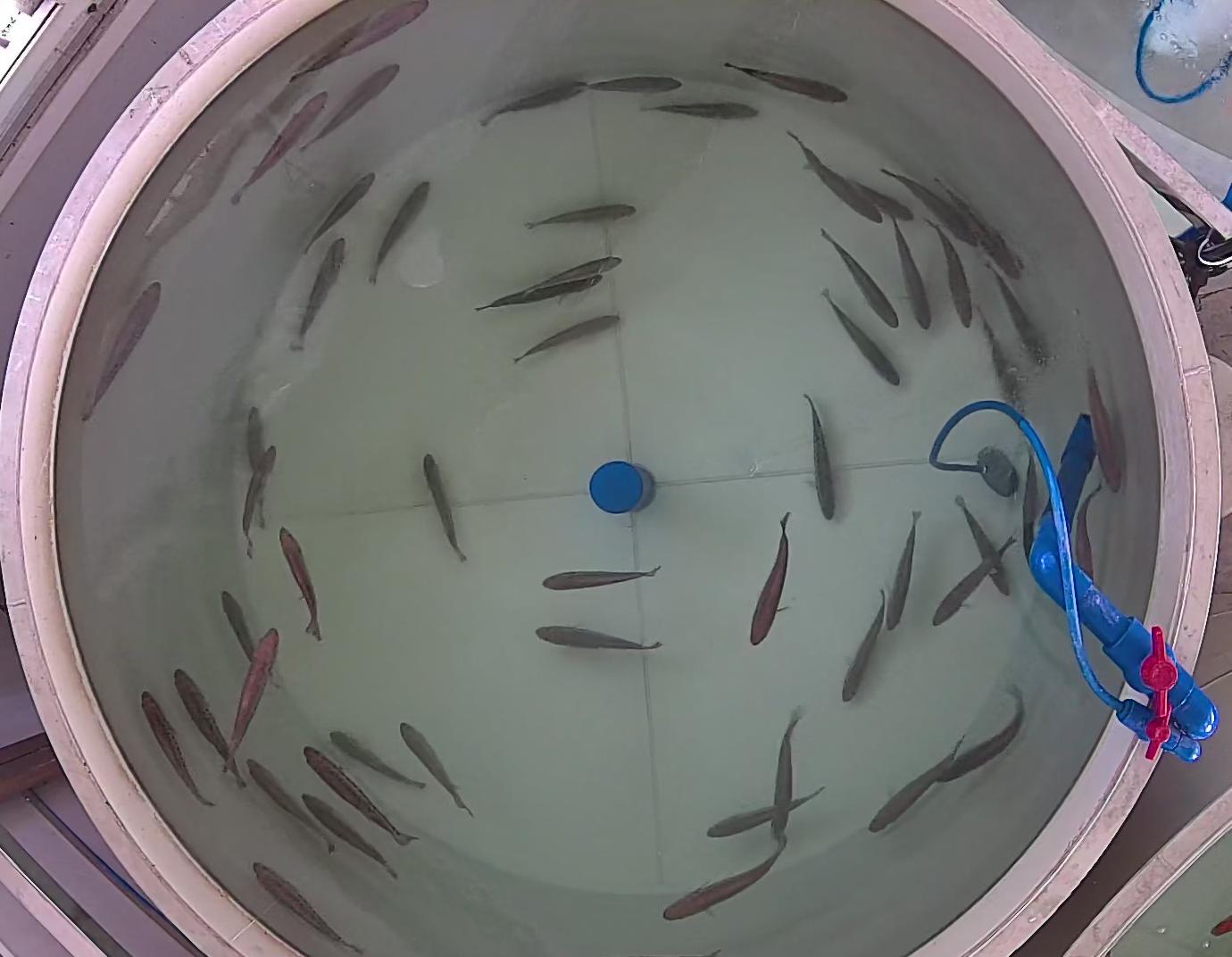

Supplement: S1 Dataset — (ZIP) [file pone.0283671.s001.zip › datasets/00023.jpg]

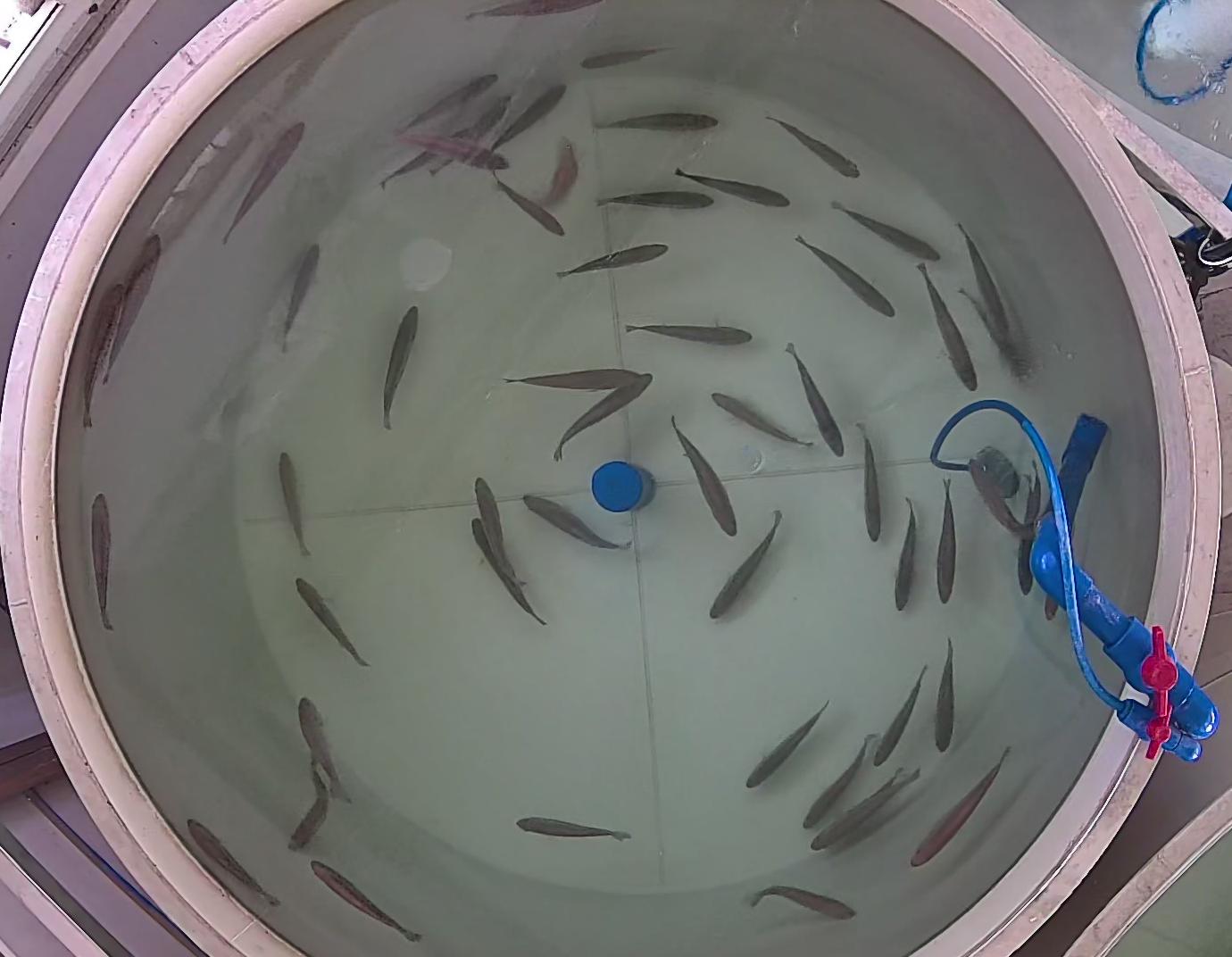

Supplement: S1 Dataset — (ZIP) [file pone.0283671.s001.zip › datasets/00024.jpg]

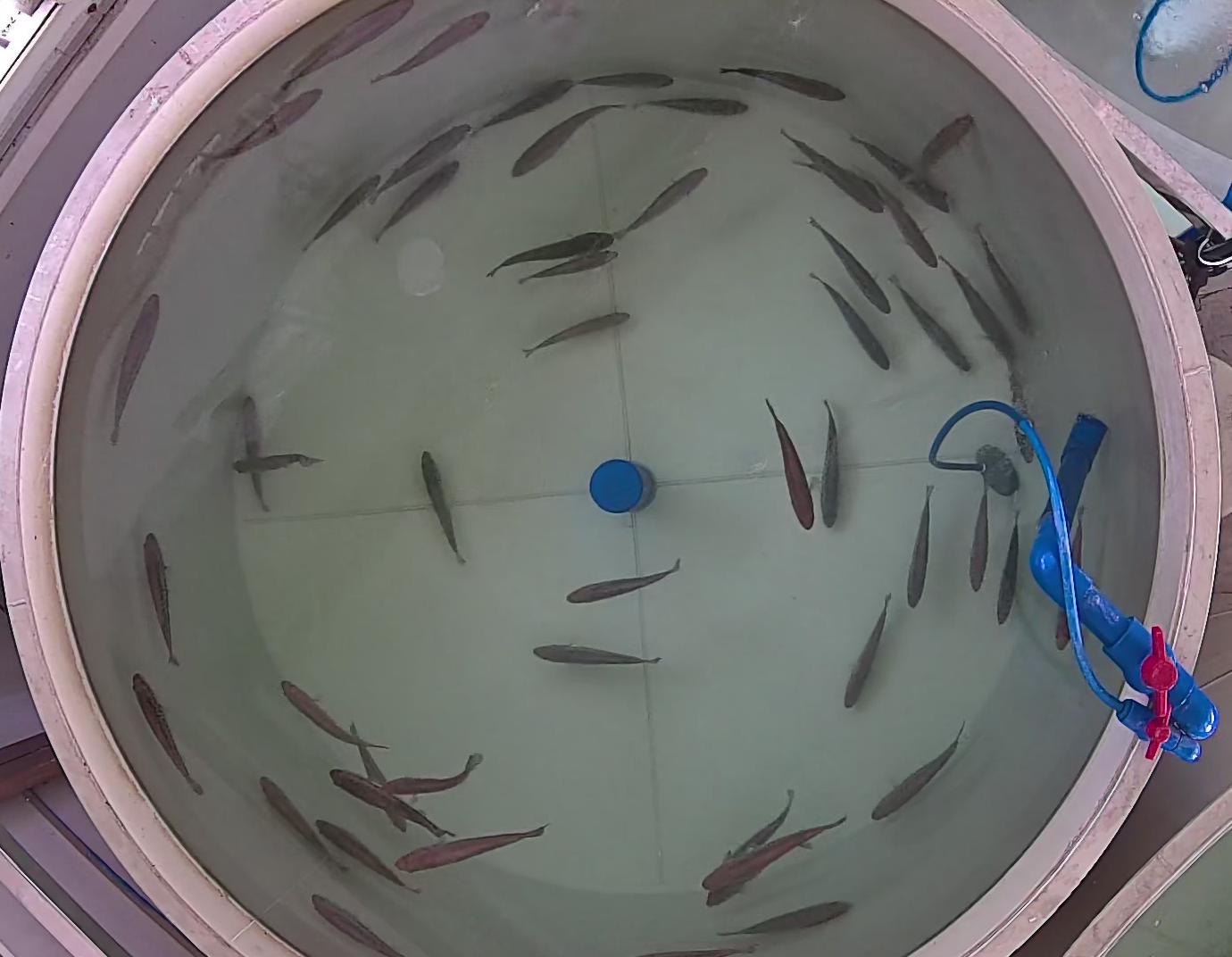

Supplement: S1 Dataset — (ZIP) [file pone.0283671.s001.zip › datasets/00025.jpg]

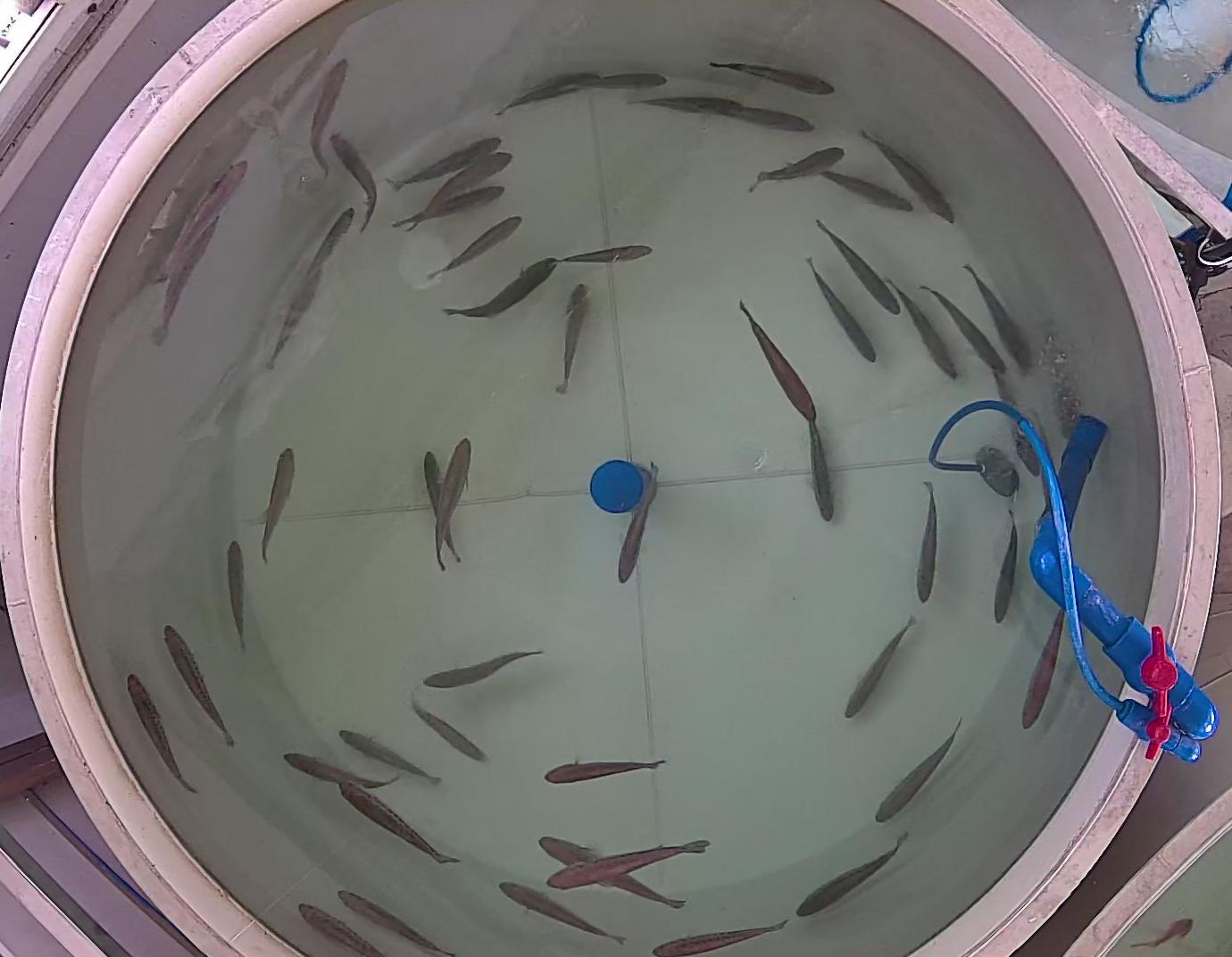

Supplement: S1 Dataset — (ZIP) [file pone.0283671.s001.zip › datasets/00026.jpg]

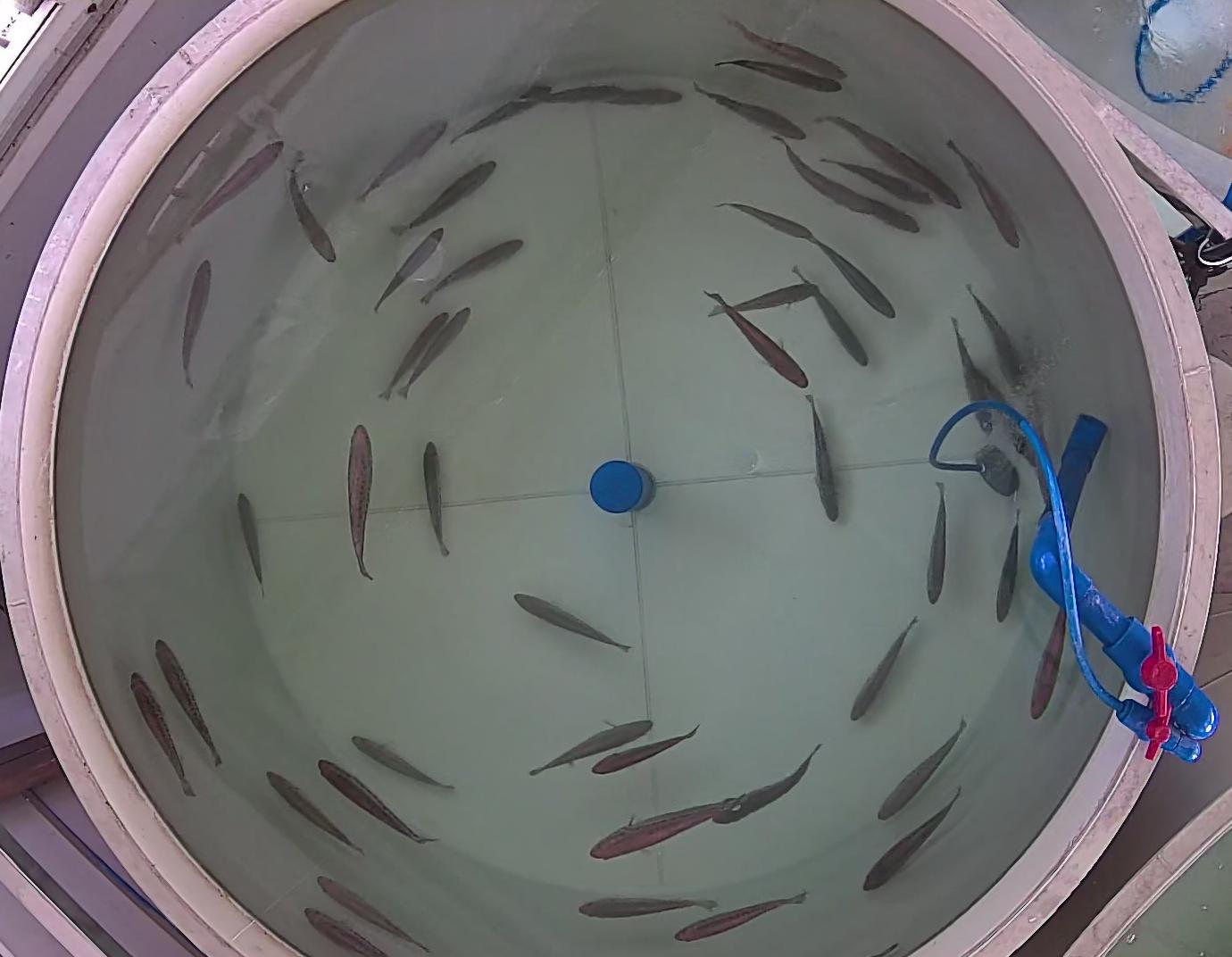

Supplement: S1 Dataset — (ZIP) [file pone.0283671.s001.zip › datasets/00027.jpg]

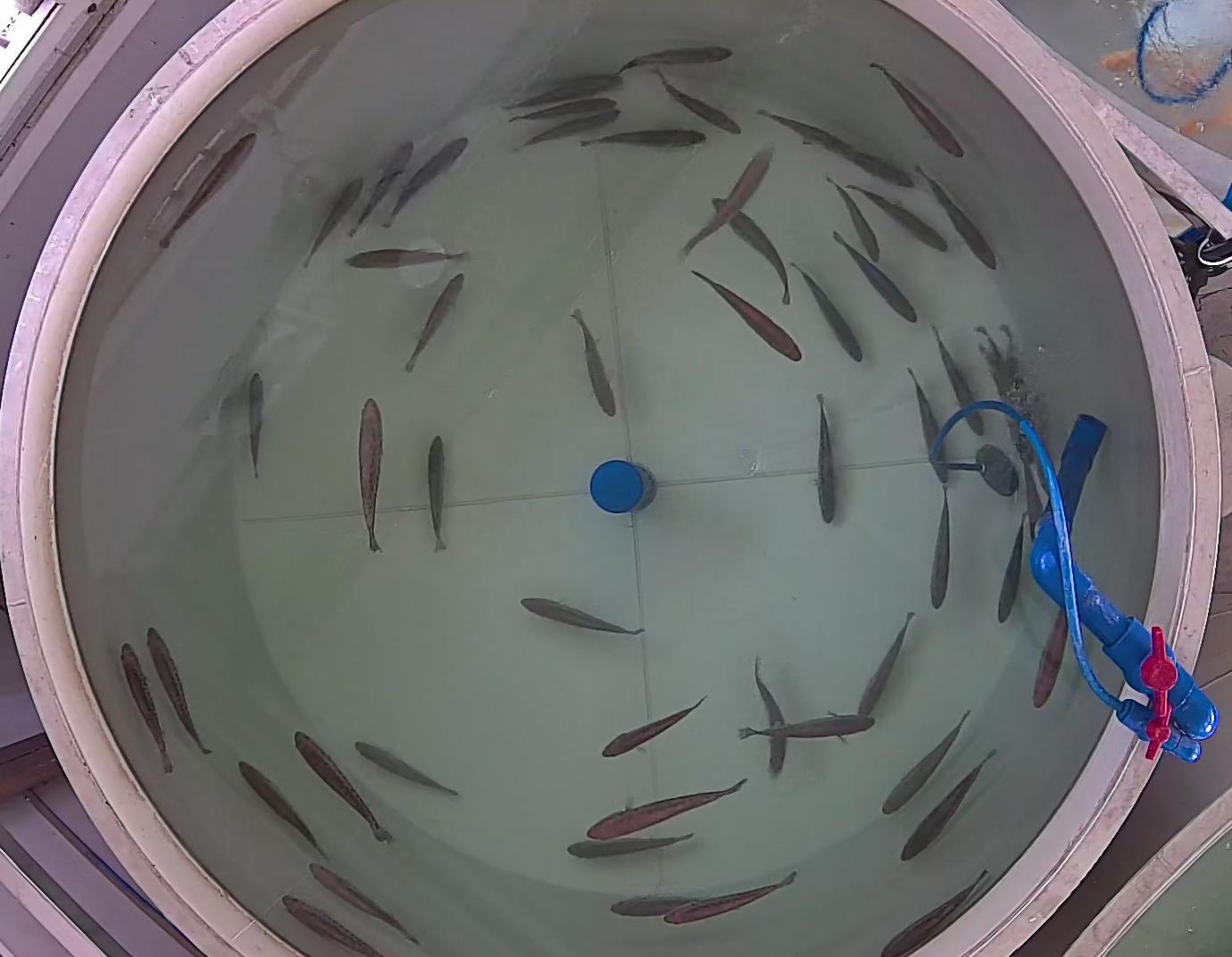

Supplement: S1 Dataset — (ZIP) [file pone.0283671.s001.zip › datasets/00028.jpg]

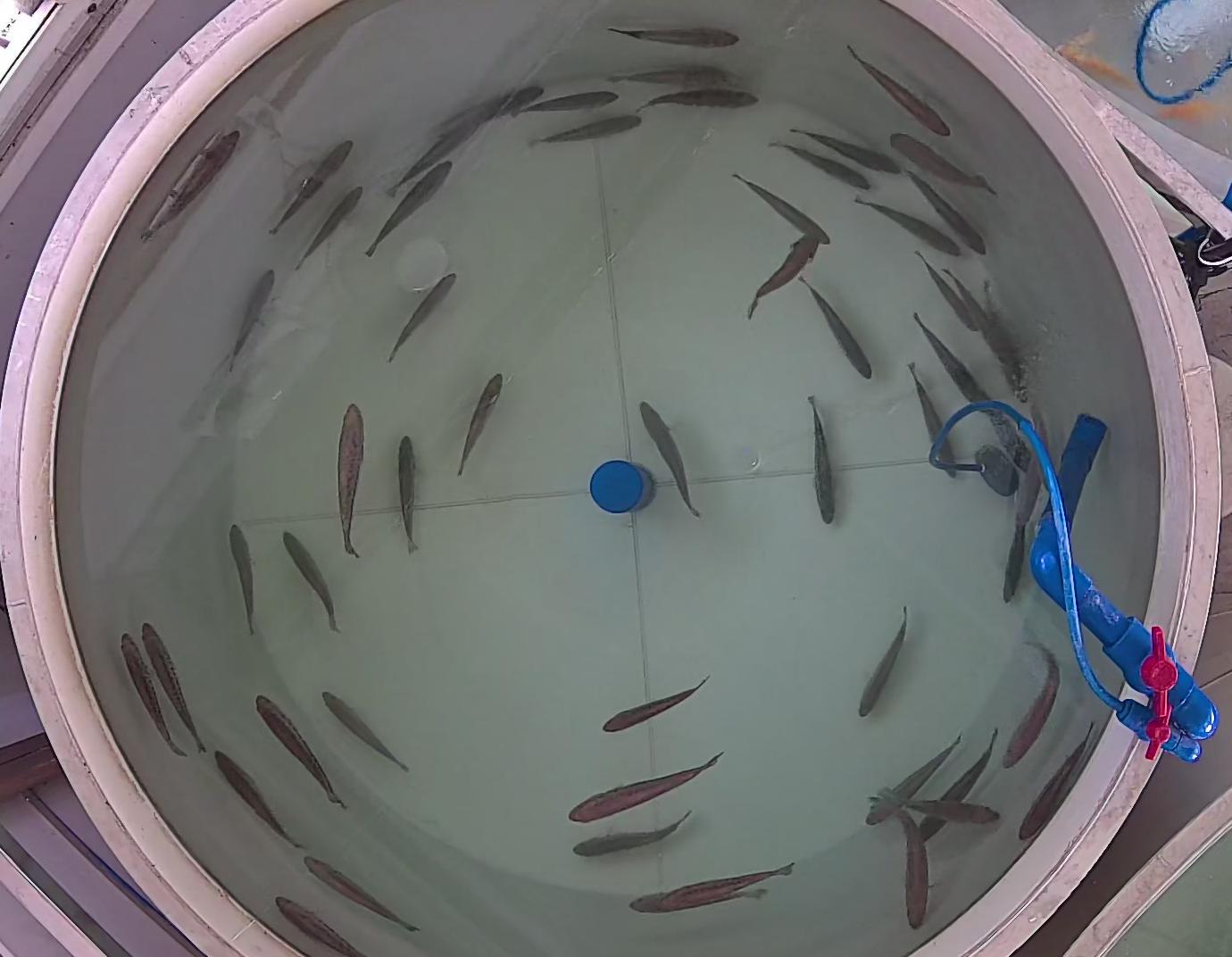

Supplement: S1 Dataset — (ZIP) [file pone.0283671.s001.zip › datasets/00029.jpg]

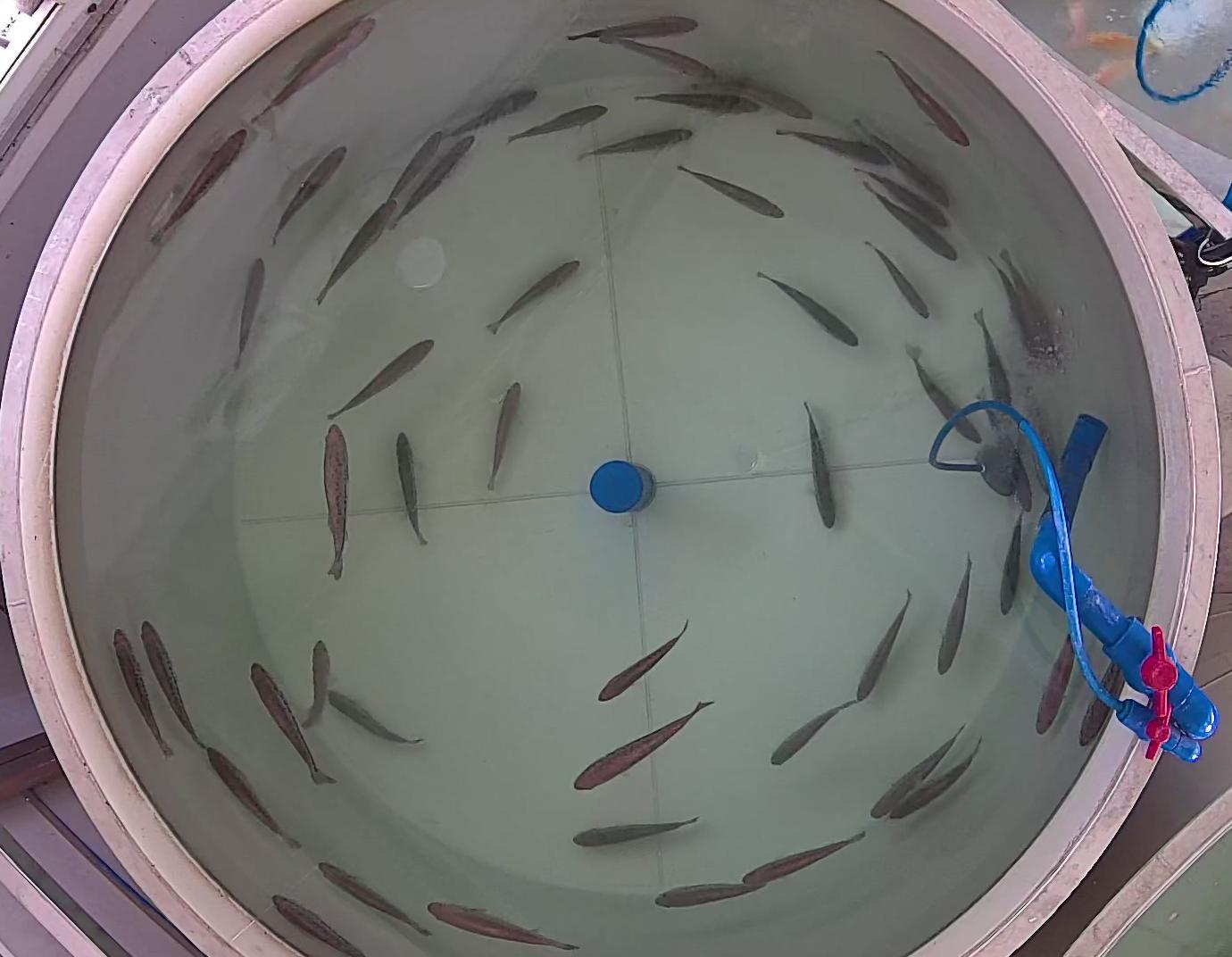

Supplement: S1 Dataset — (ZIP) [file pone.0283671.s001.zip › datasets/00030.jpg]

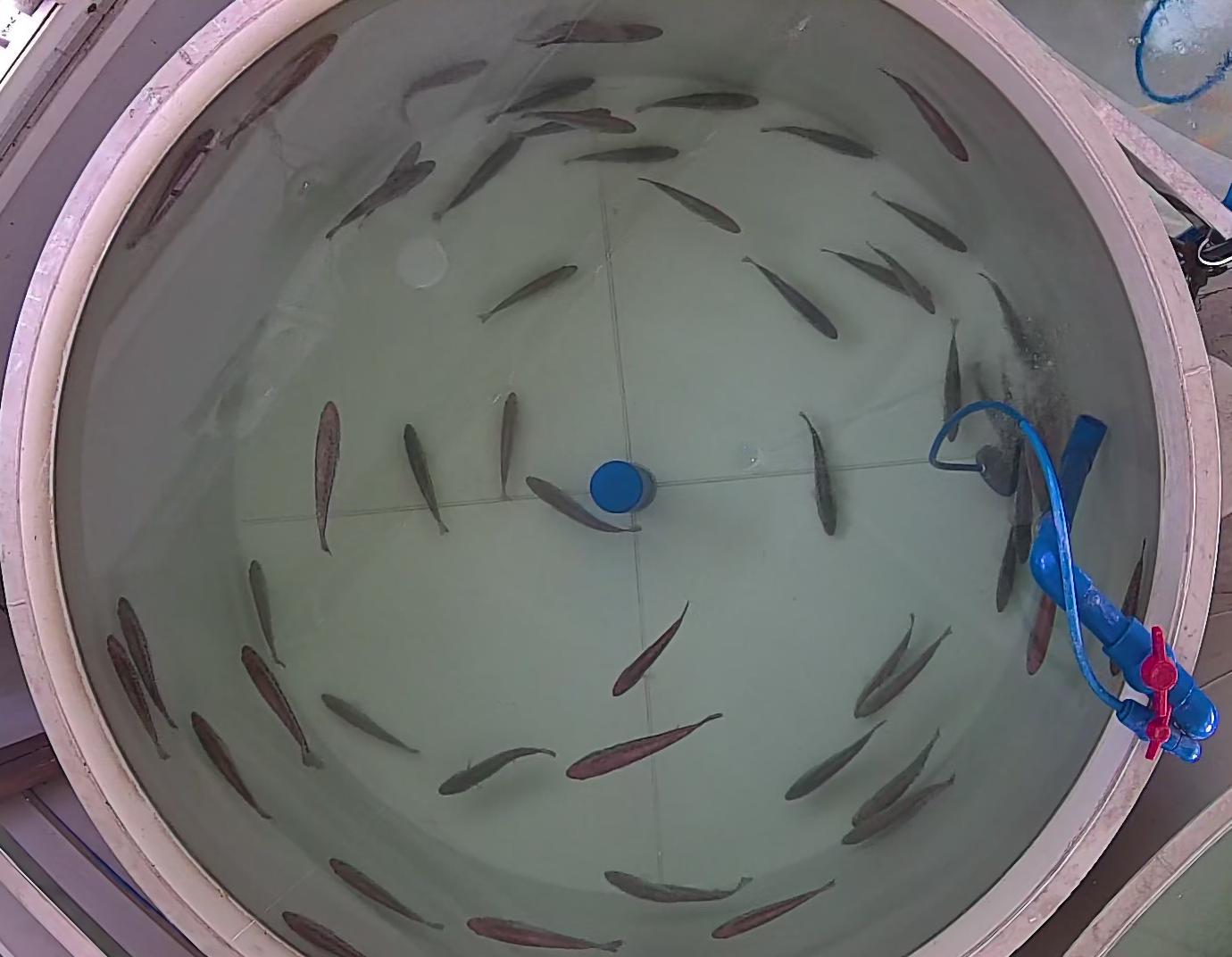

Supplement: S1 Dataset — (ZIP) [file pone.0283671.s001.zip › datasets/00031.jpg]

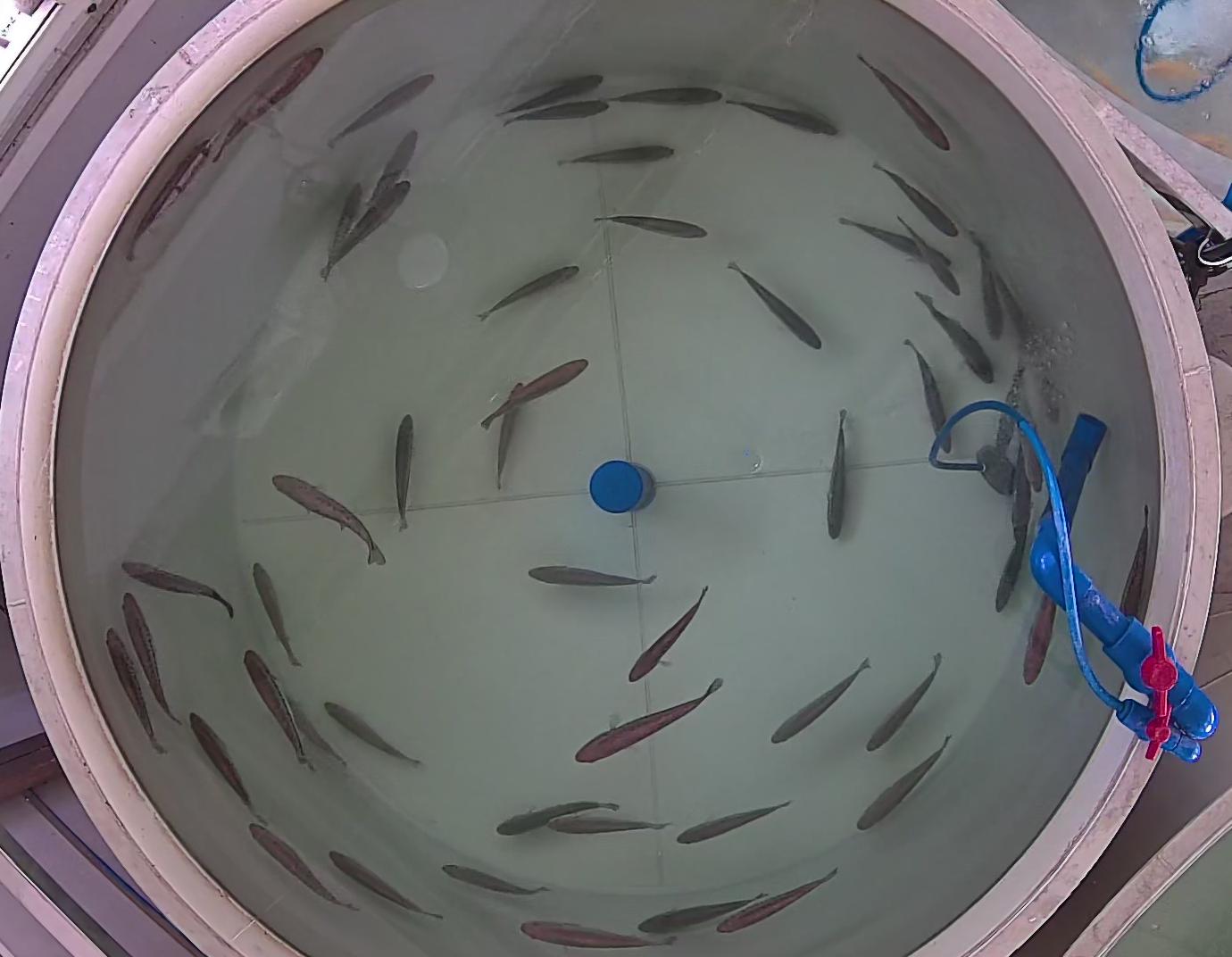

Supplement: S1 Dataset — (ZIP) [file pone.0283671.s001.zip › datasets/00032.jpg]

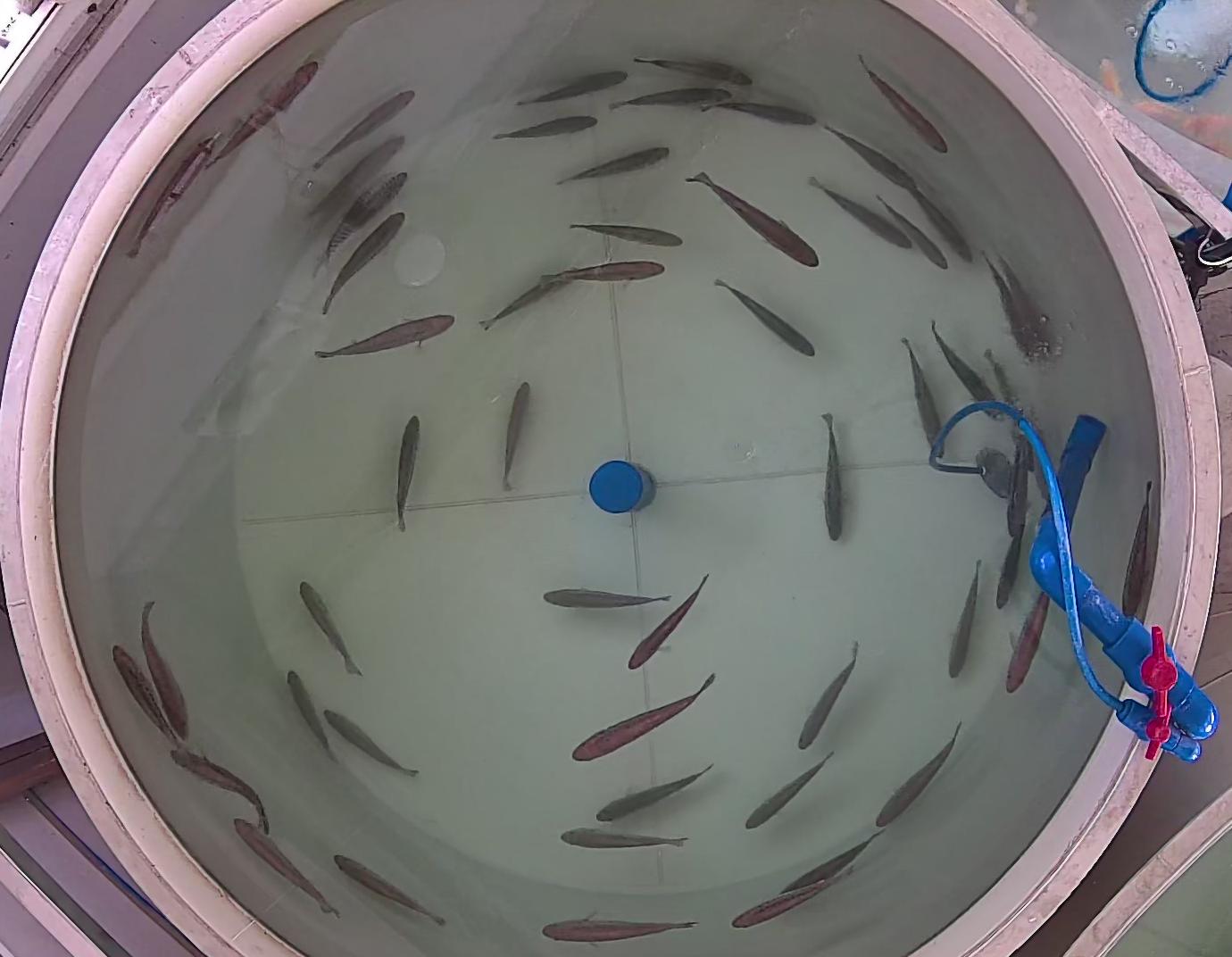

Supplement: S1 Dataset — (ZIP) [file pone.0283671.s001.zip › datasets/00033.jpg]

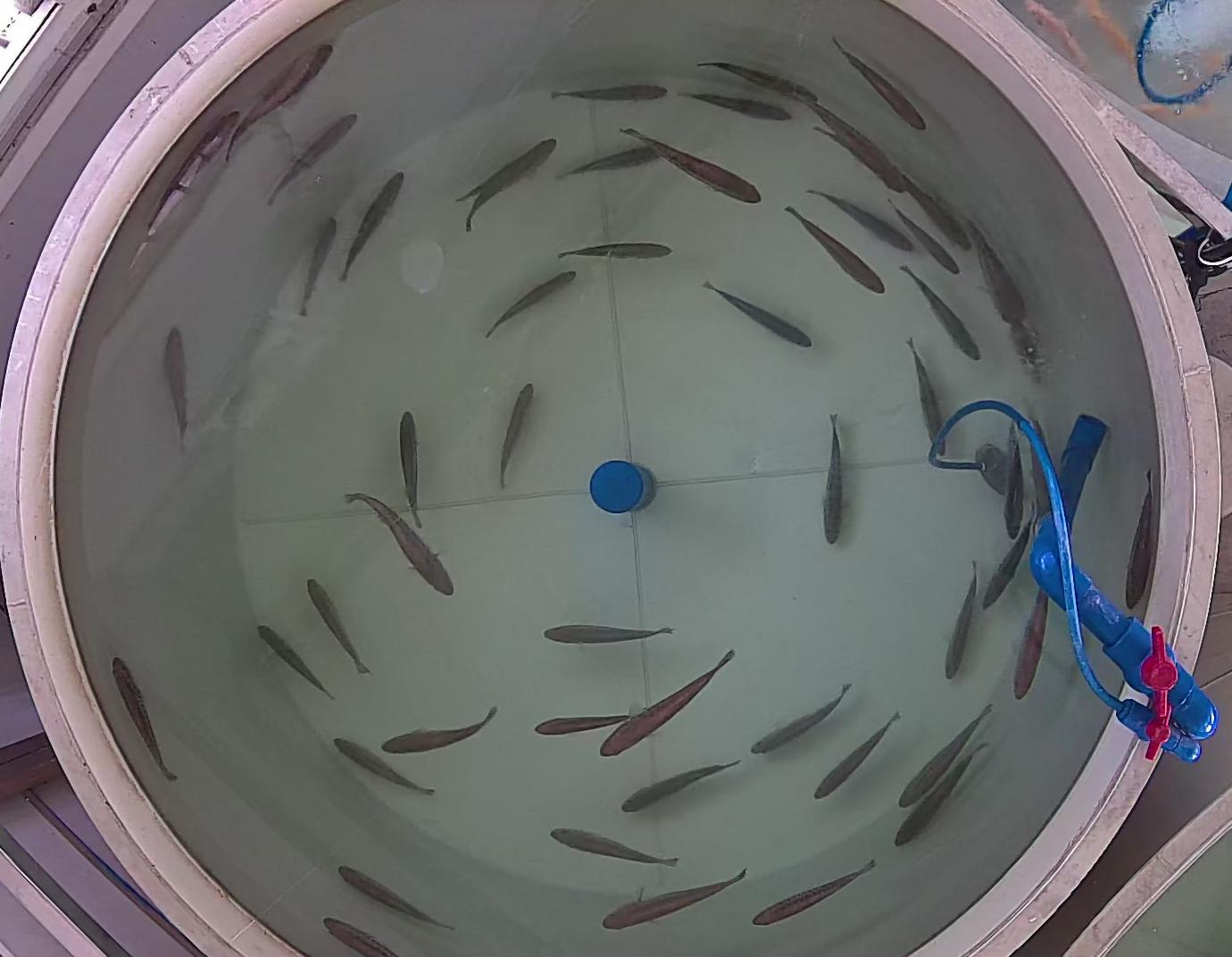

Supplement: S1 Dataset — (ZIP) [file pone.0283671.s001.zip › datasets/00034.jpg]

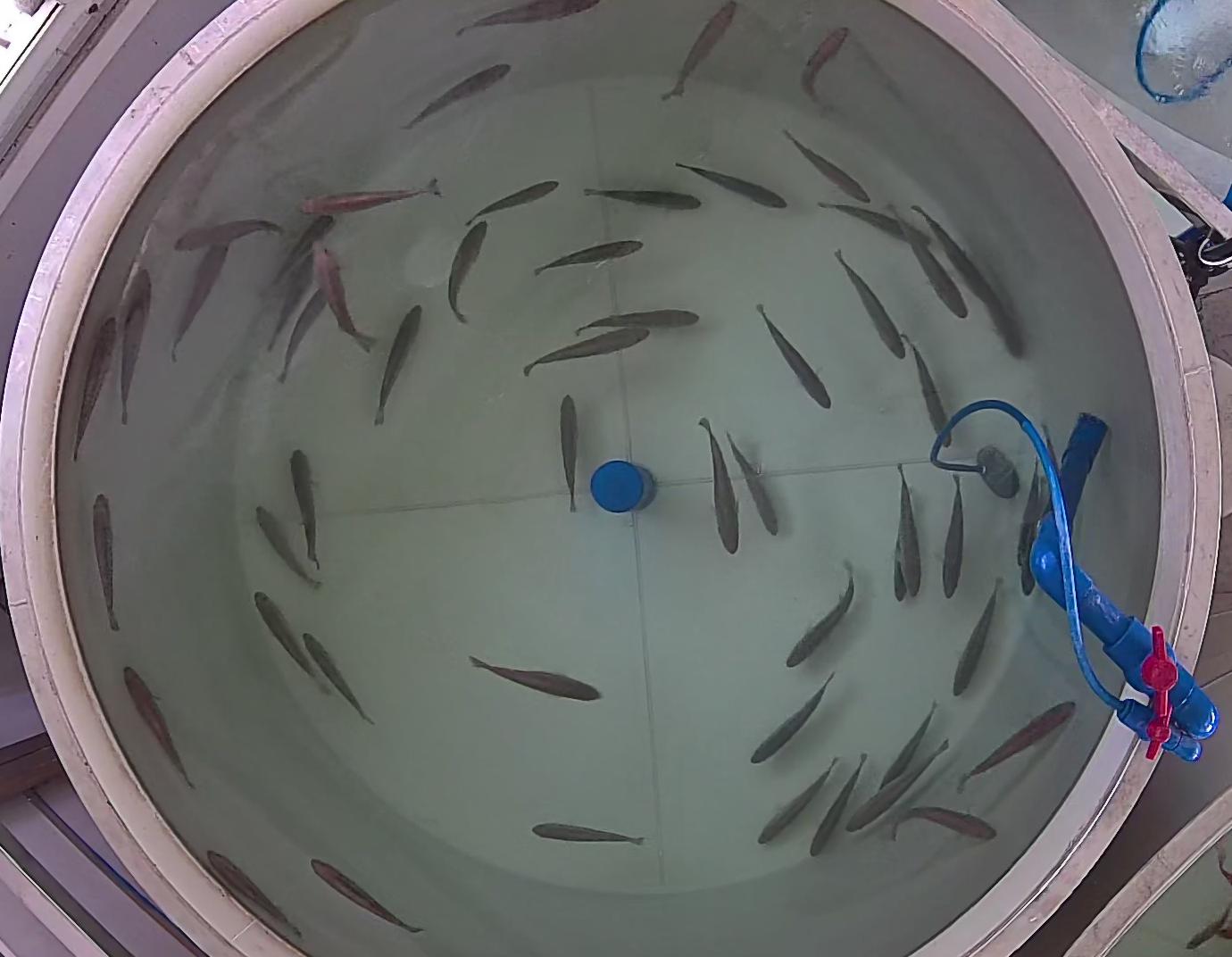

Supplement: S1 Dataset — (ZIP) [file pone.0283671.s001.zip › datasets/00035.jpg]

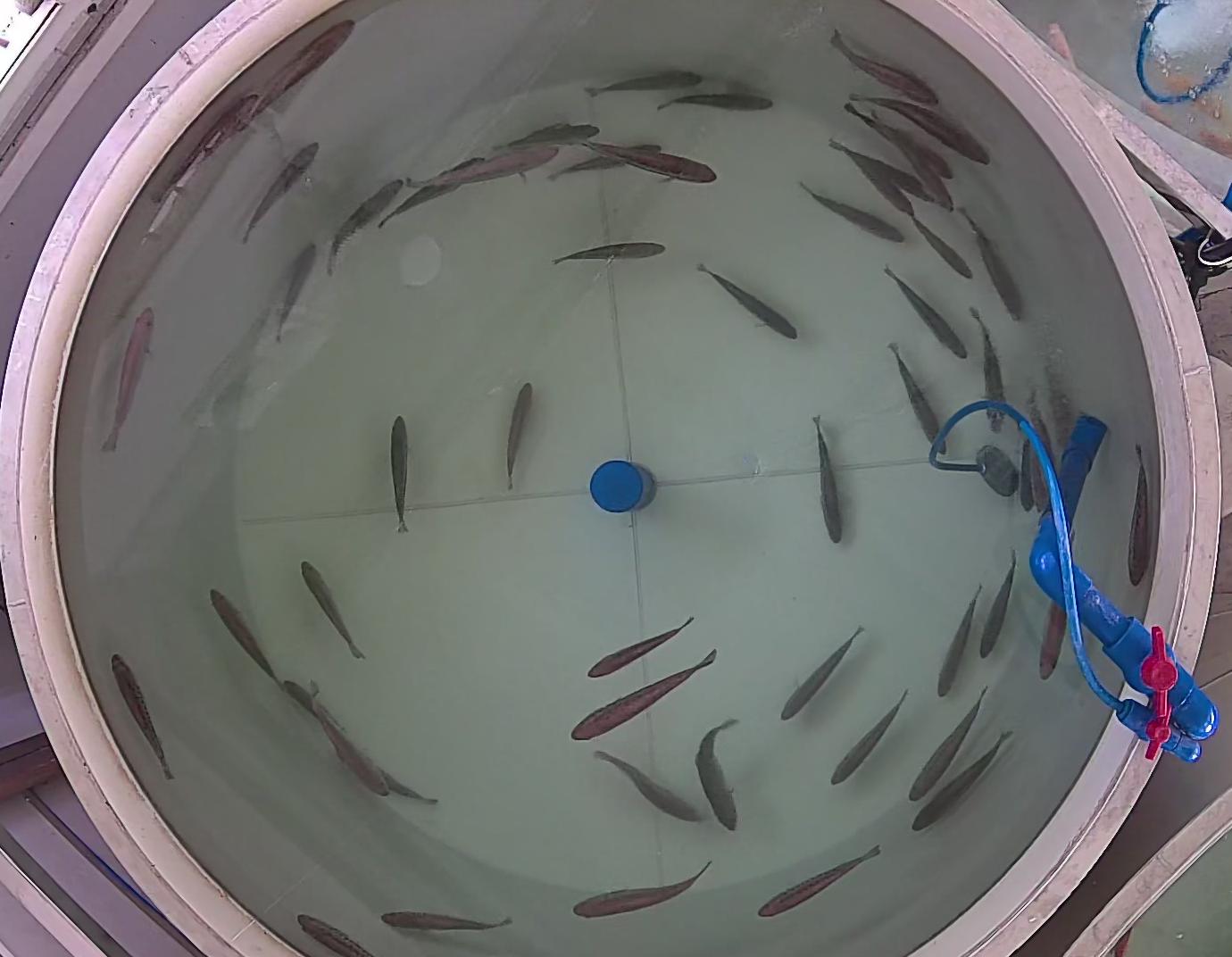

Supplement: S1 Dataset — (ZIP) [file pone.0283671.s001.zip › datasets/00036.jpg]

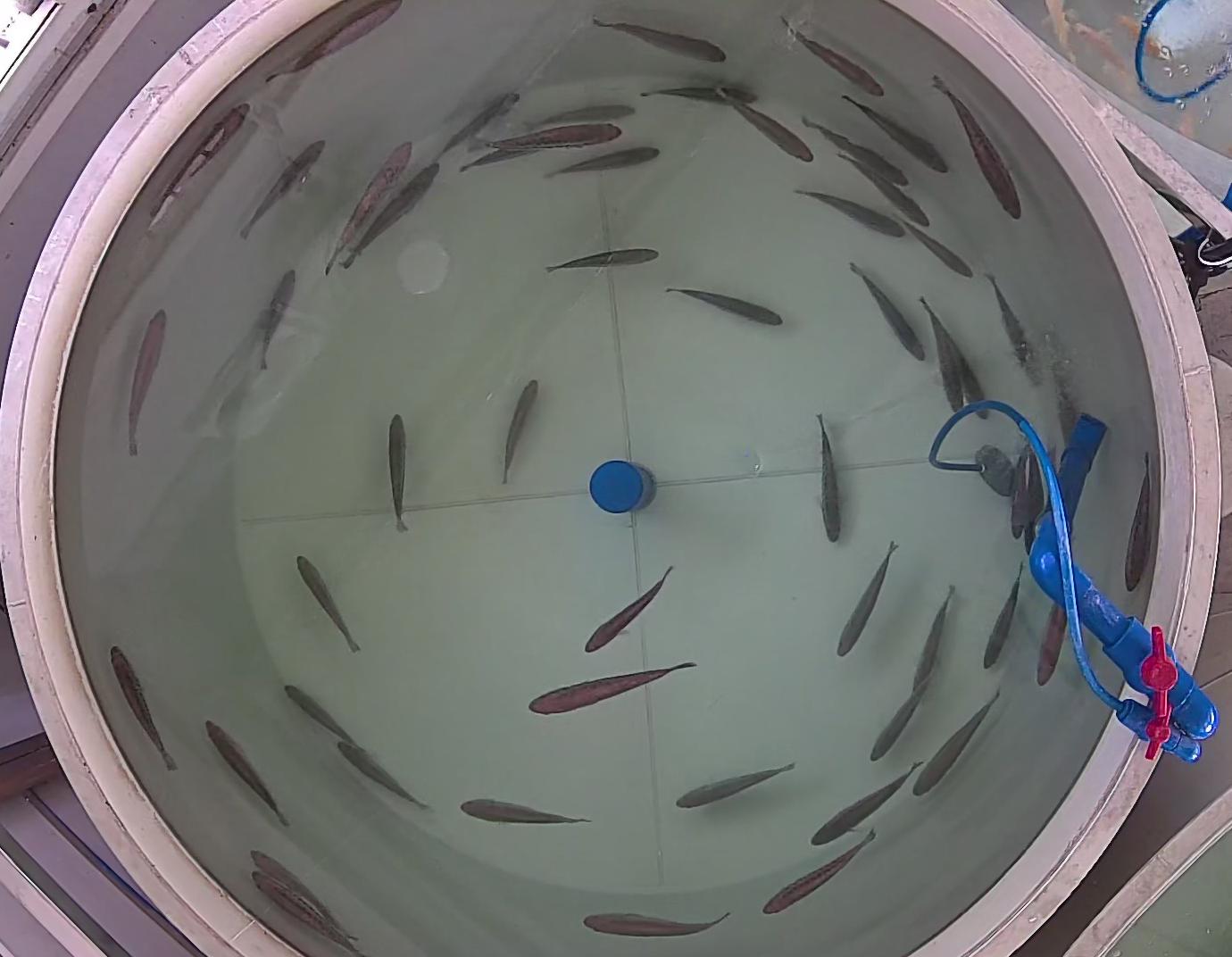

Supplement: S1 Dataset — (ZIP) [file pone.0283671.s001.zip › datasets/00037.jpg]

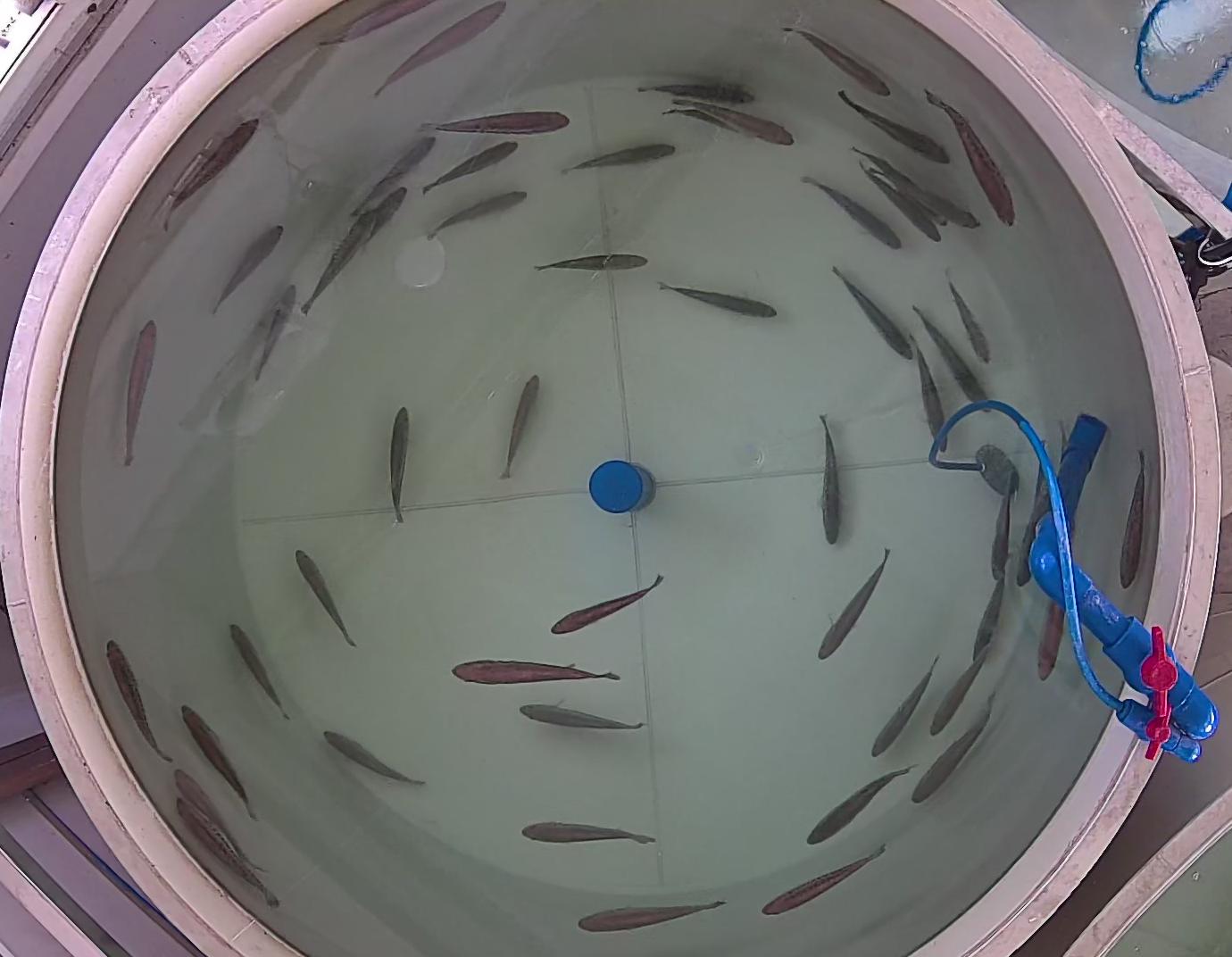

Supplement: S1 Dataset — (ZIP) [file pone.0283671.s001.zip › datasets/00038.jpg]

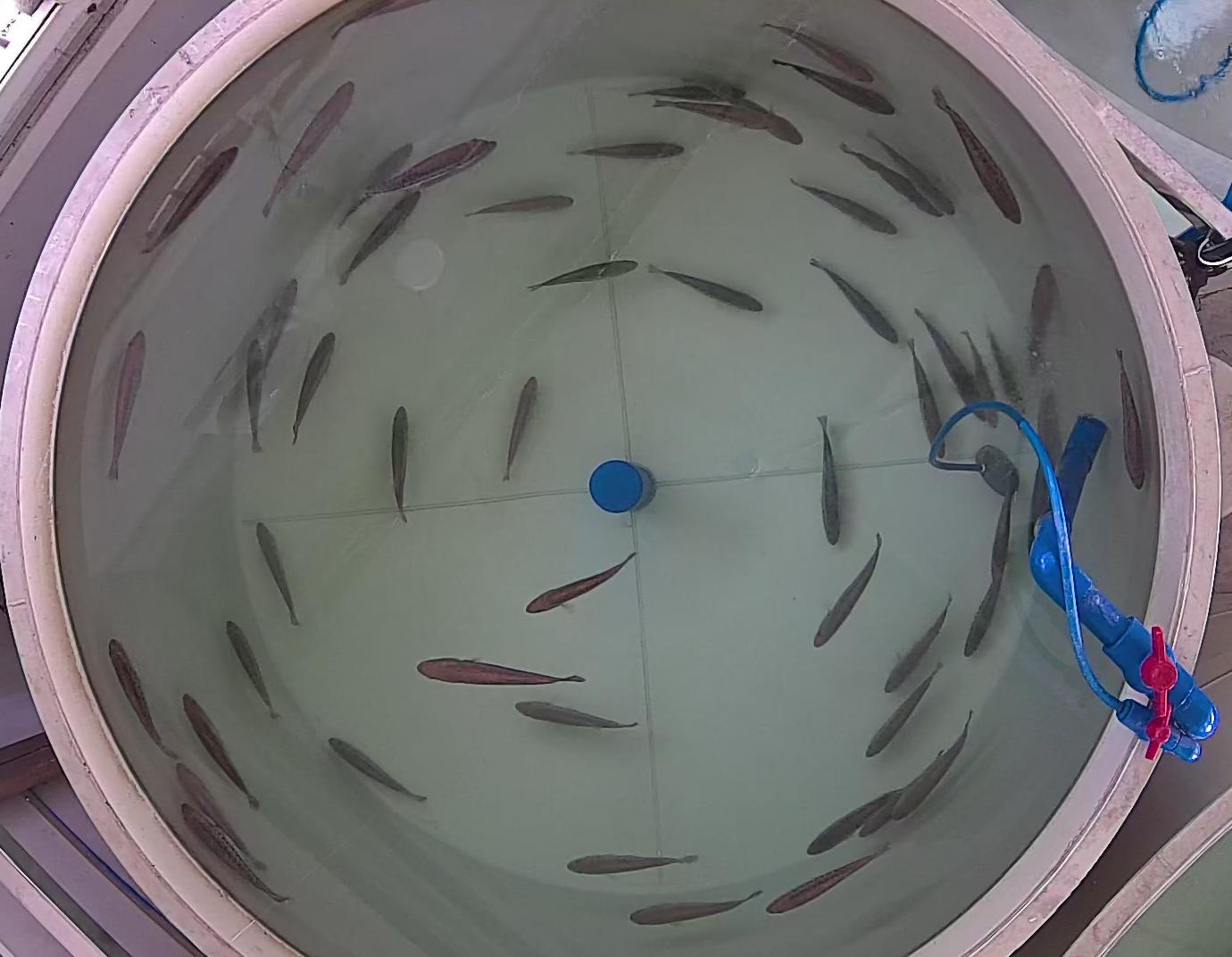

Supplement: S1 Dataset — (ZIP) [file pone.0283671.s001.zip › datasets/00039.jpg]

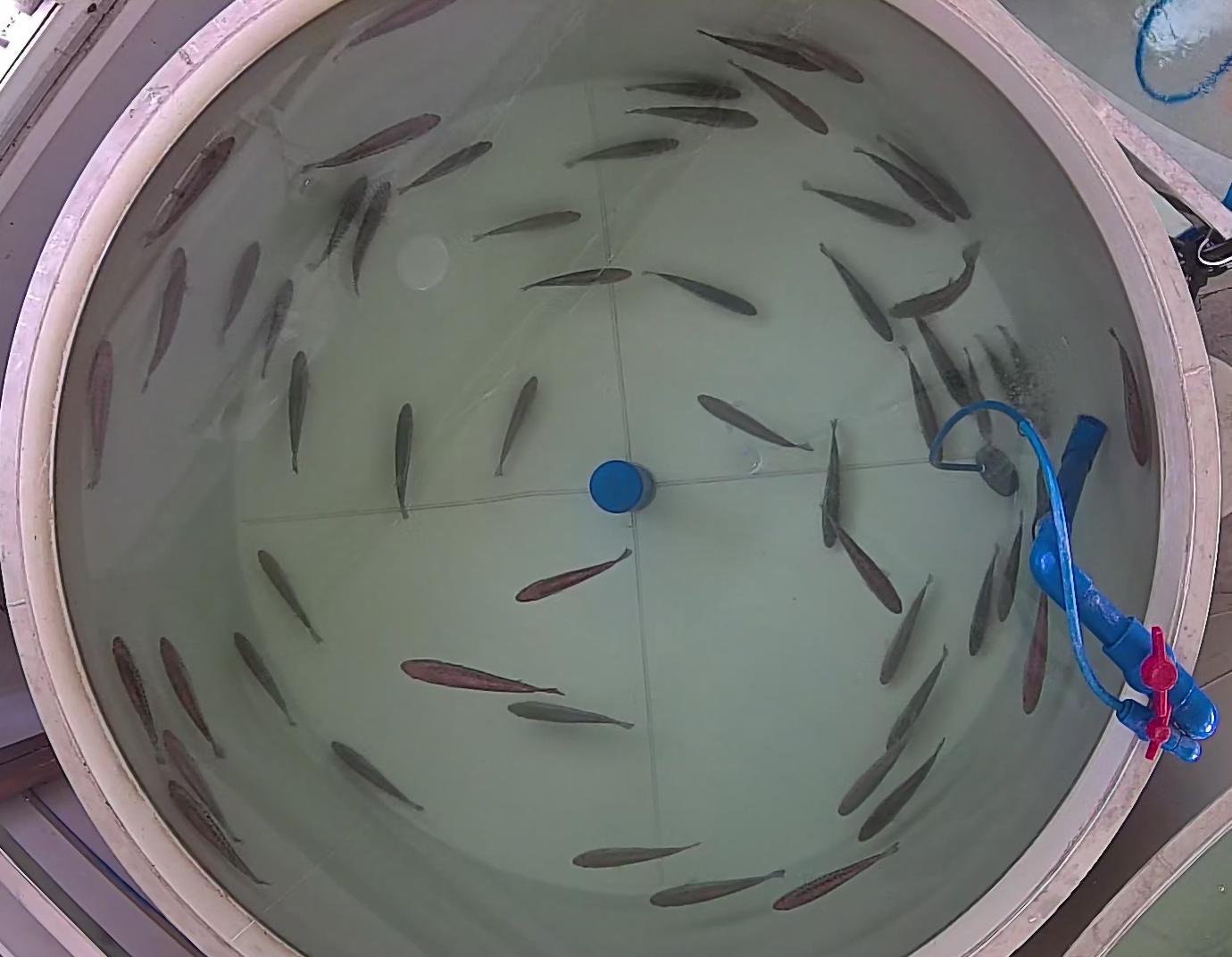

Supplement: S1 Dataset — (ZIP) [file pone.0283671.s001.zip › datasets/00040.jpg]

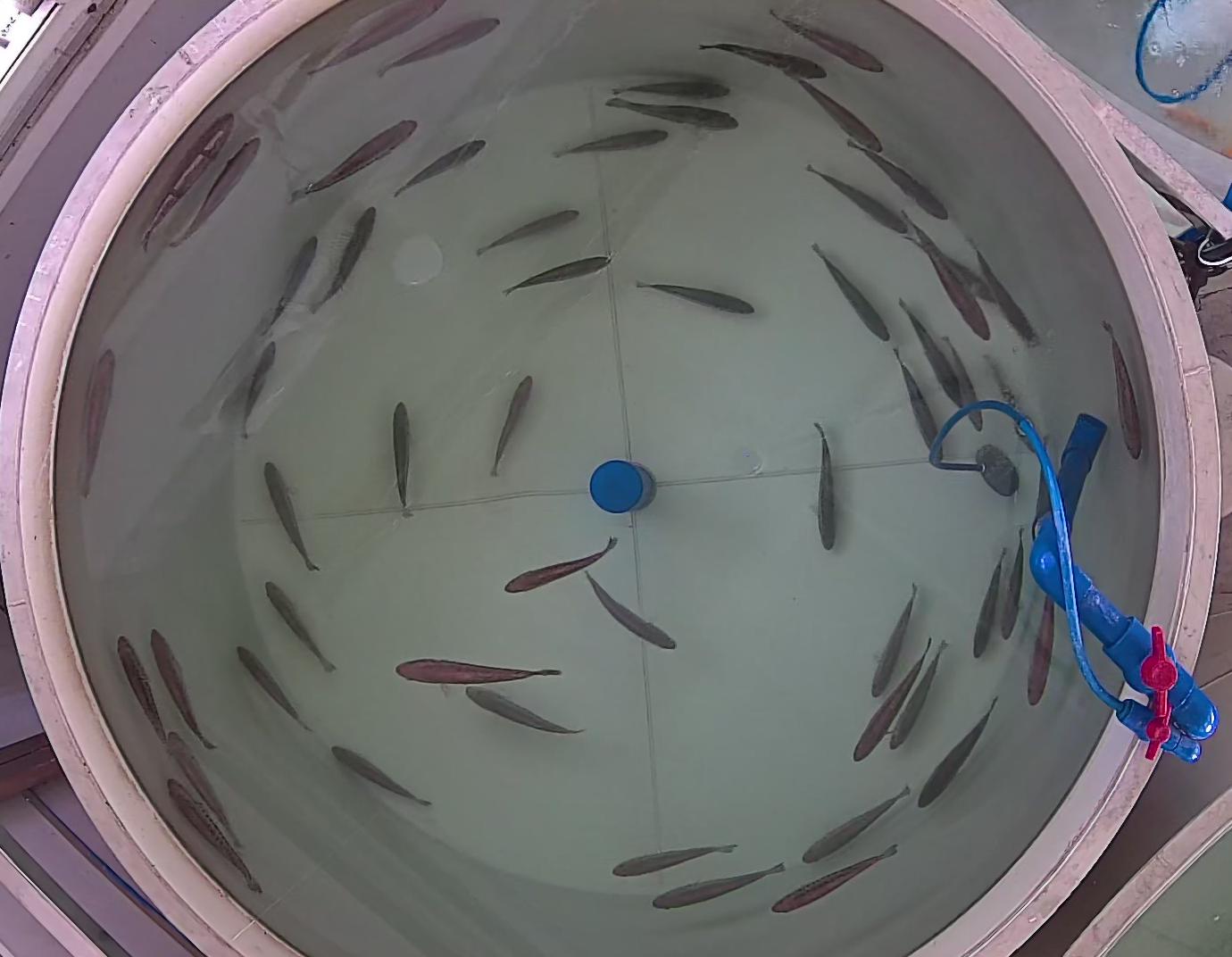

Supplement: S1 Dataset — (ZIP) [file pone.0283671.s001.zip › datasets/00041.jpg]

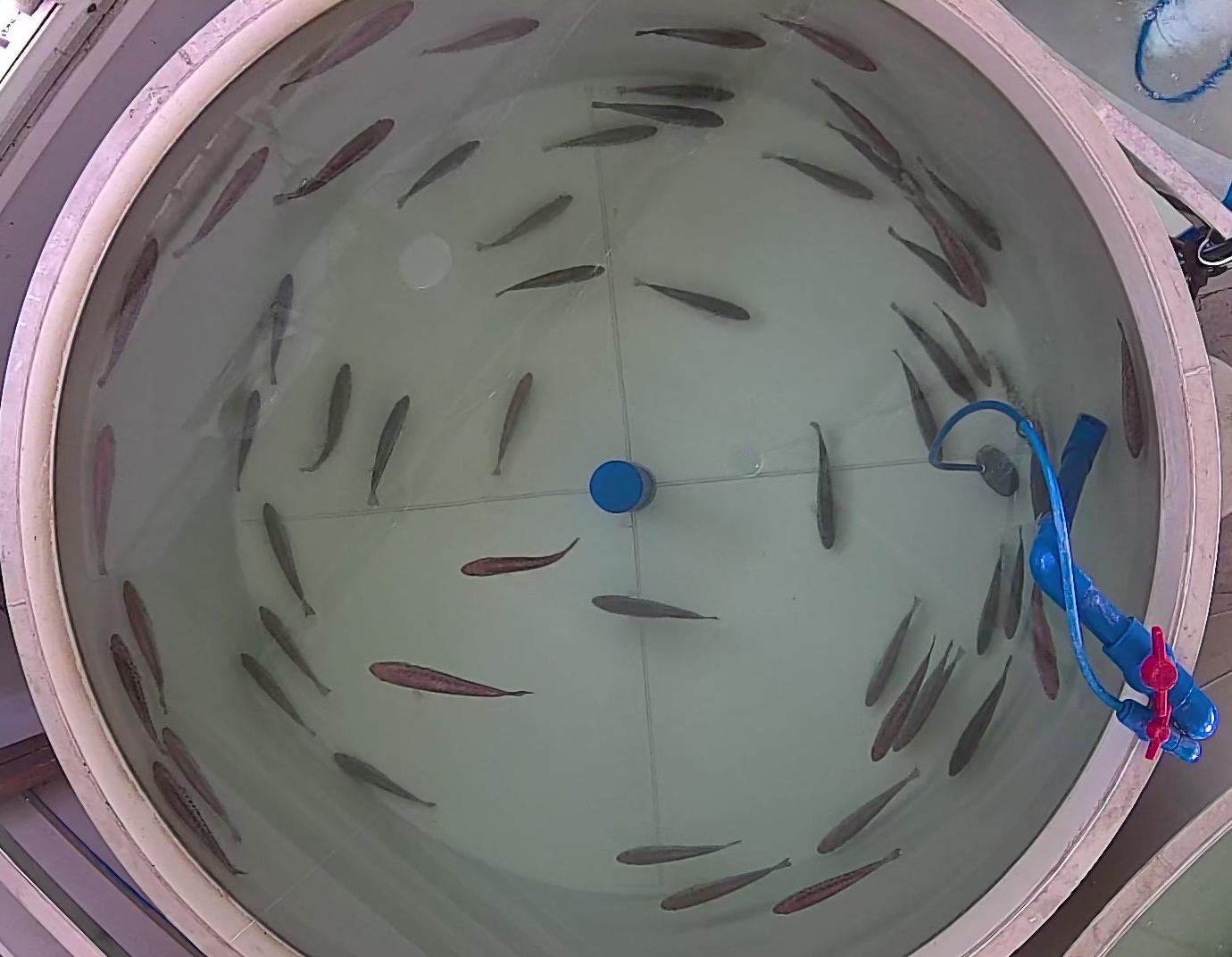

Supplement: S1 Dataset — (ZIP) [file pone.0283671.s001.zip › datasets/00042.jpg]

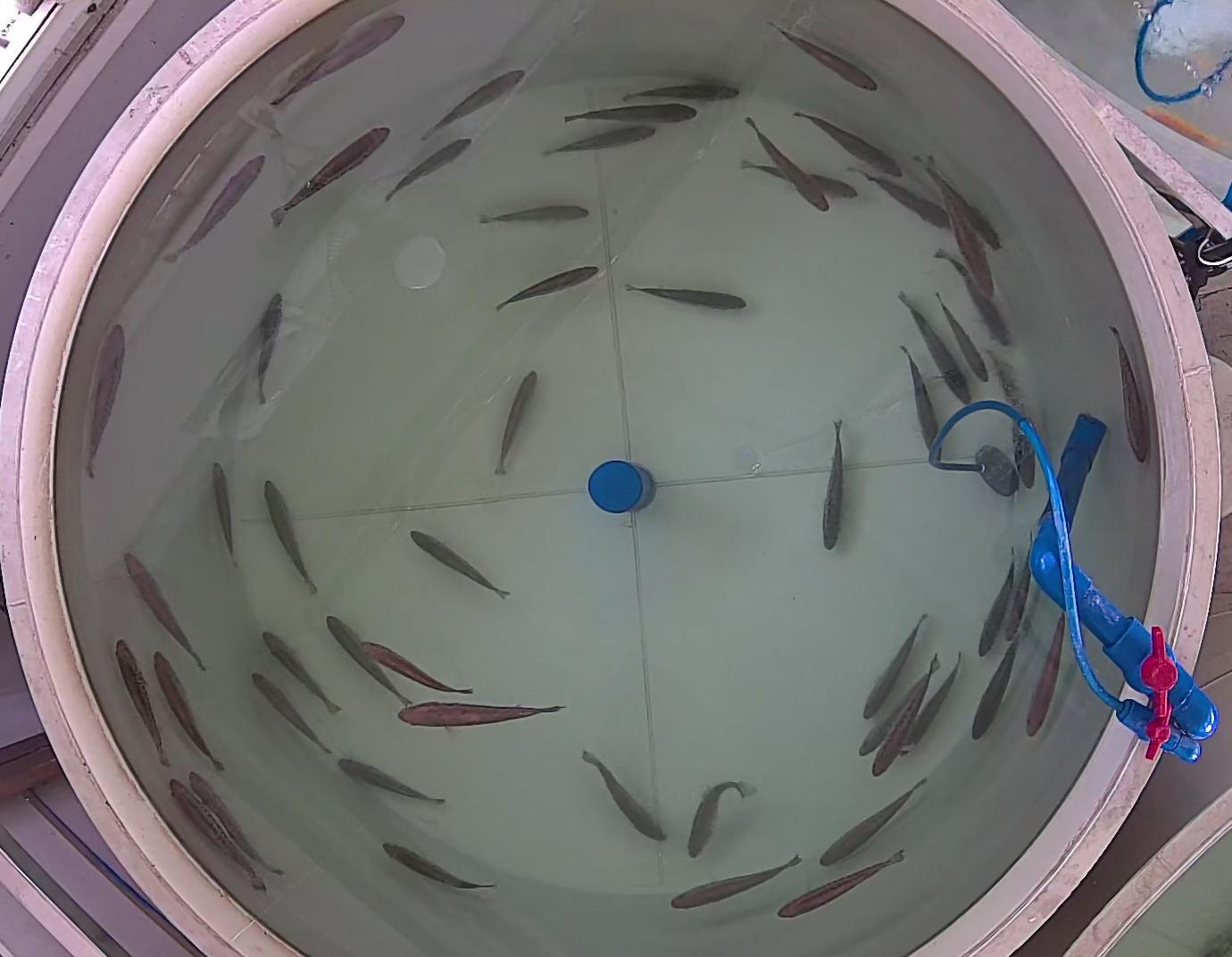

Supplement: S1 Dataset — (ZIP) [file pone.0283671.s001.zip › datasets/00043.jpg]

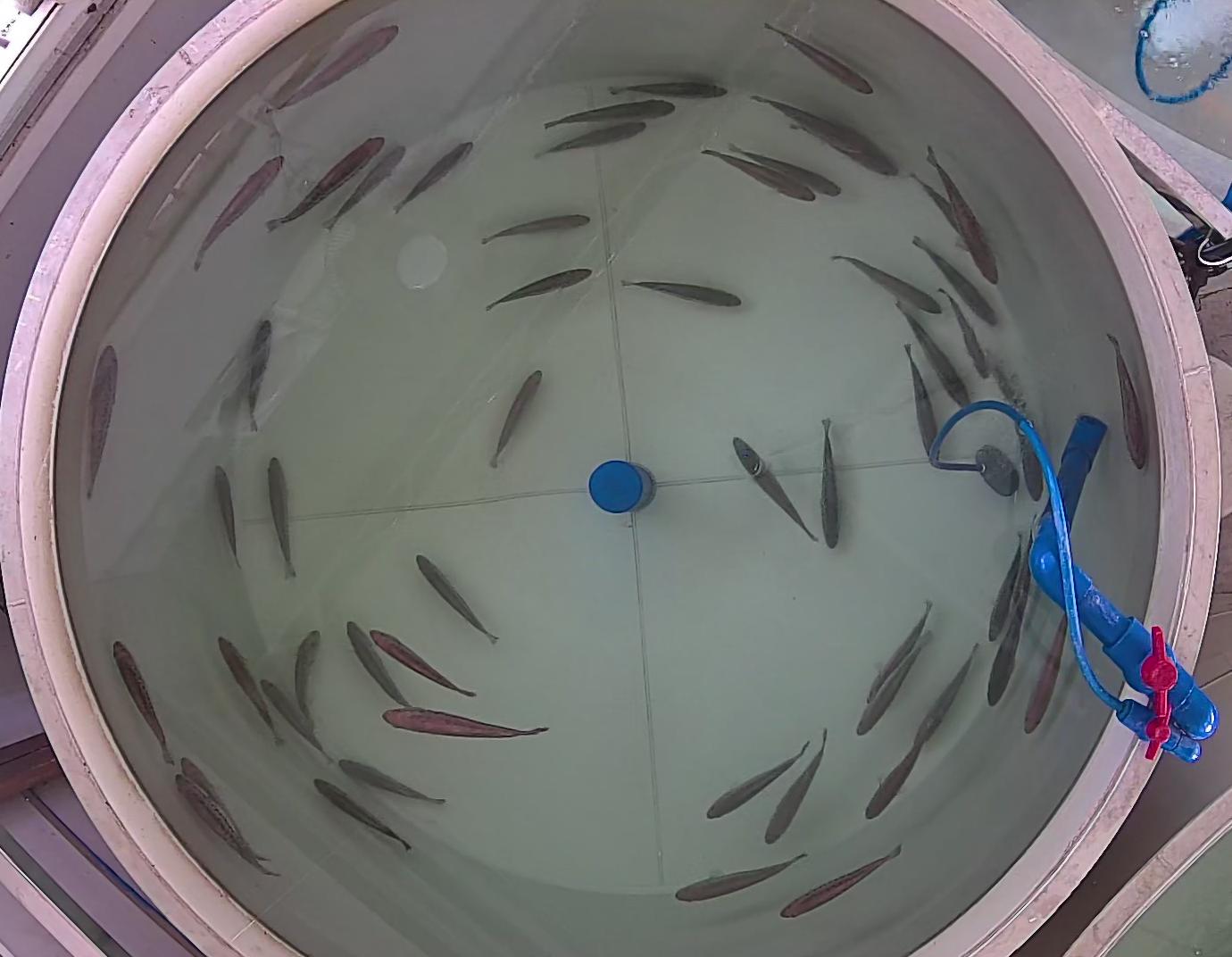

Supplement: S1 Dataset — (ZIP) [file pone.0283671.s001.zip › datasets/00044.jpg]

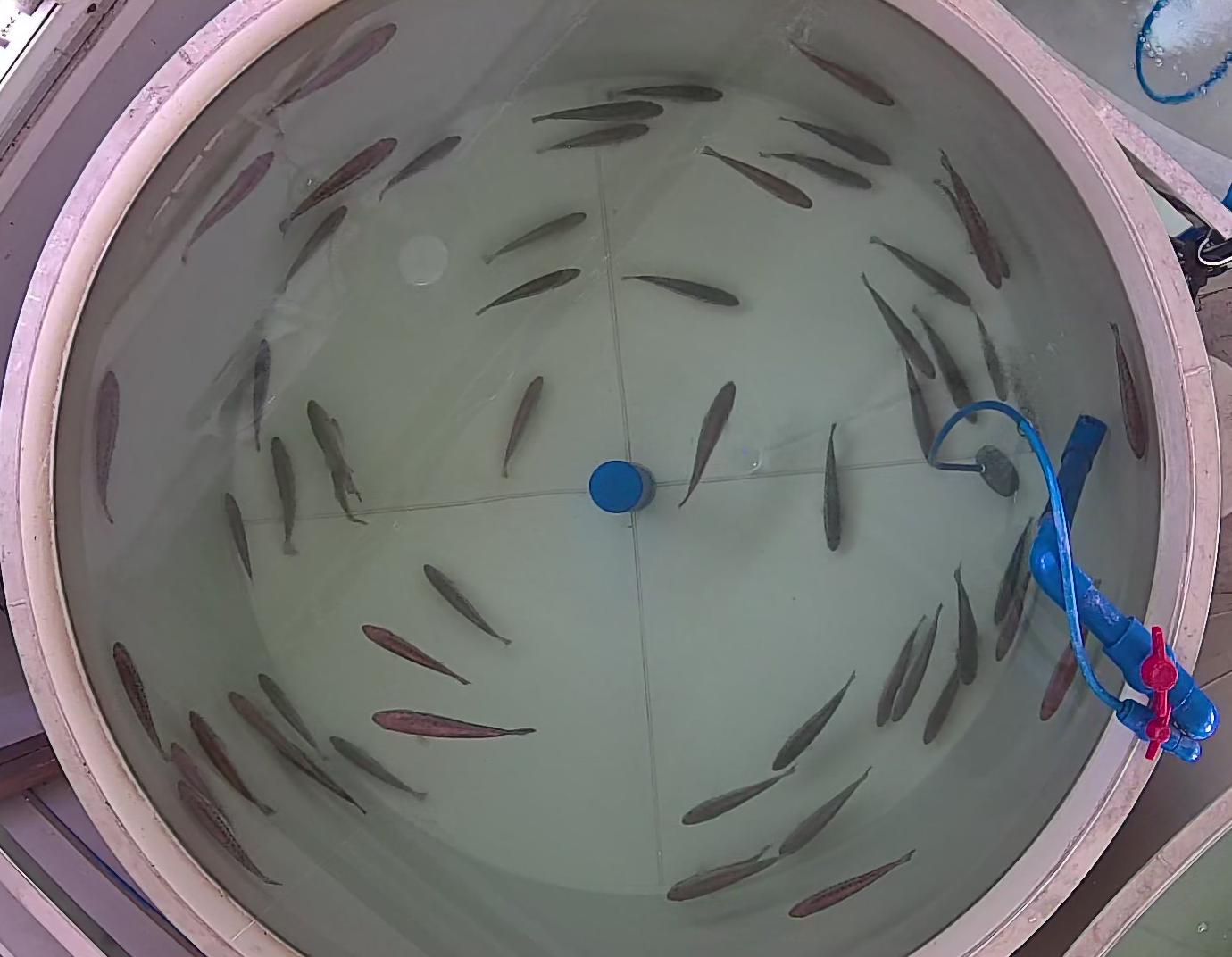

Supplement: S1 Dataset — (ZIP) [file pone.0283671.s001.zip › datasets/00045.jpg]

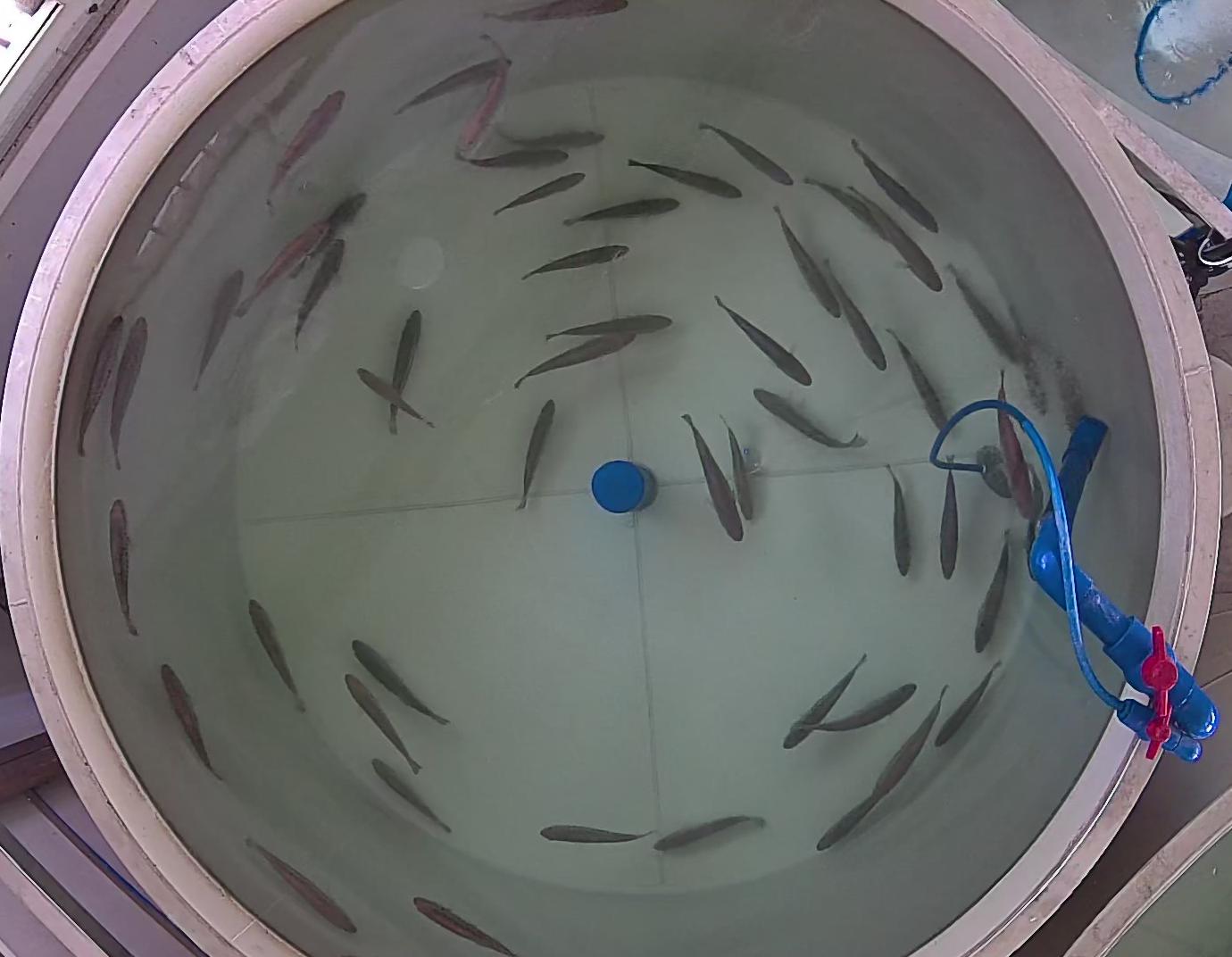

Supplement: S1 Dataset — (ZIP) [file pone.0283671.s001.zip › datasets/00046.jpg]

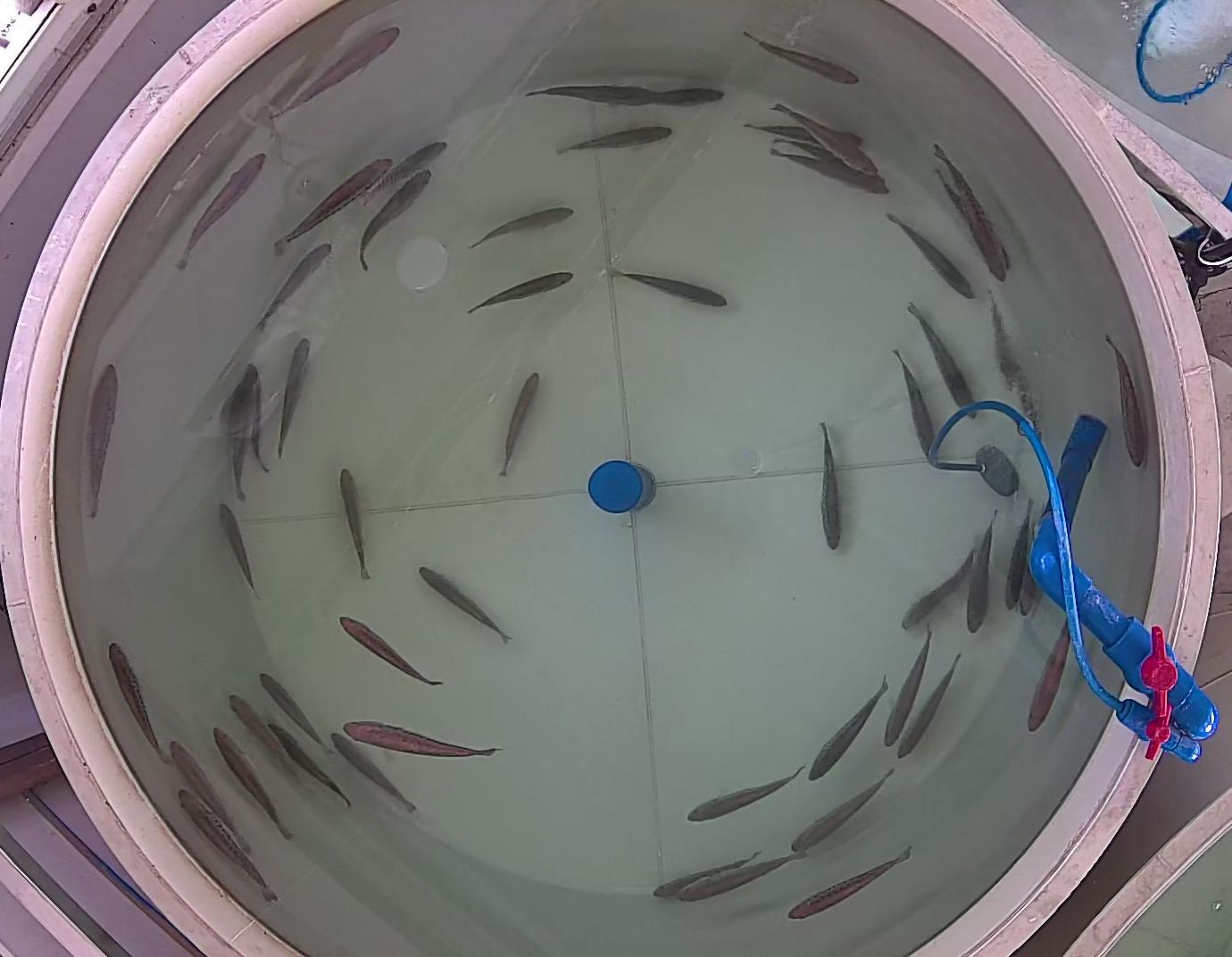

Supplement: S1 Dataset — (ZIP) [file pone.0283671.s001.zip › datasets/00047.jpg]

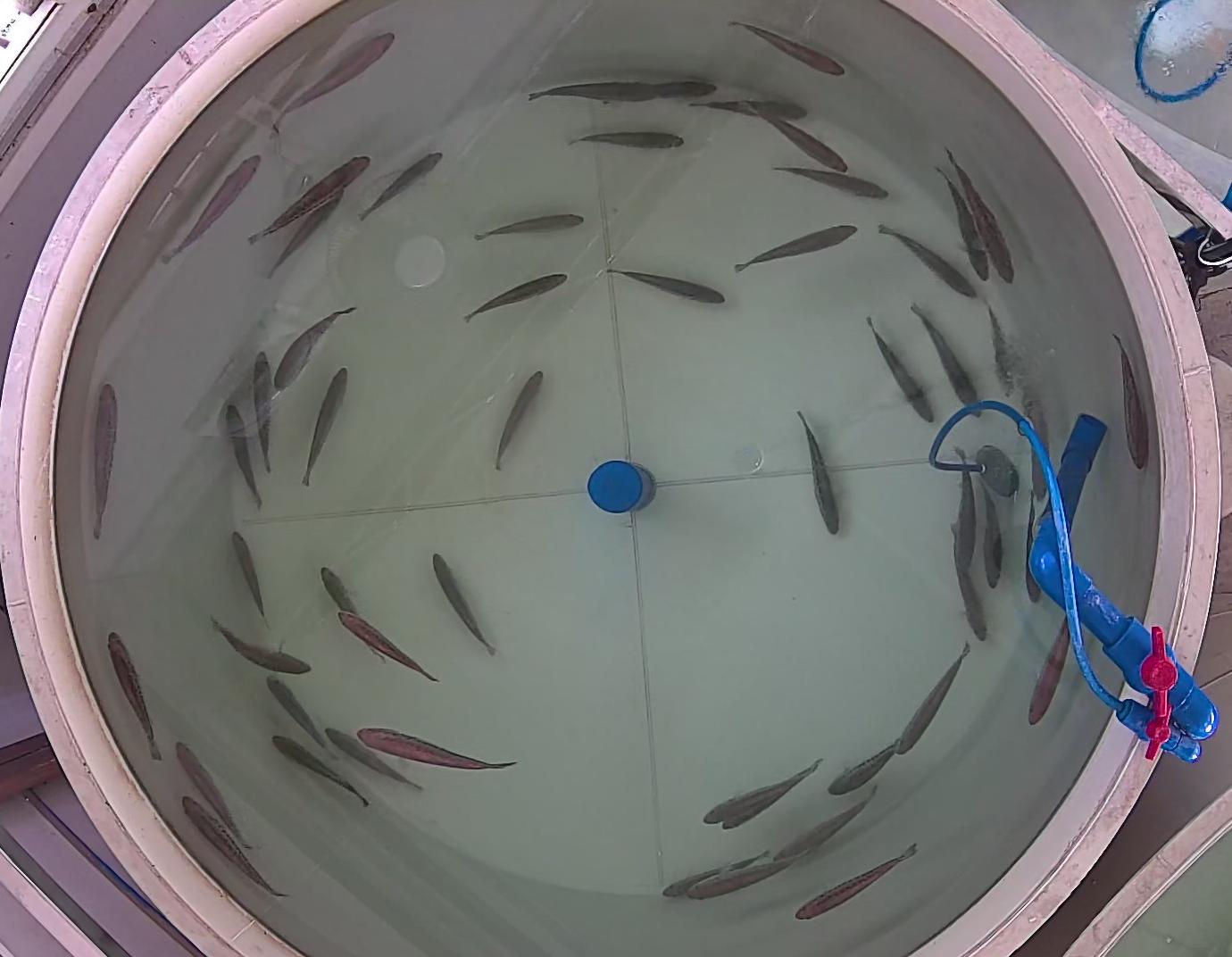

Supplement: S1 Dataset — (ZIP) [file pone.0283671.s001.zip › datasets/00048.jpg]

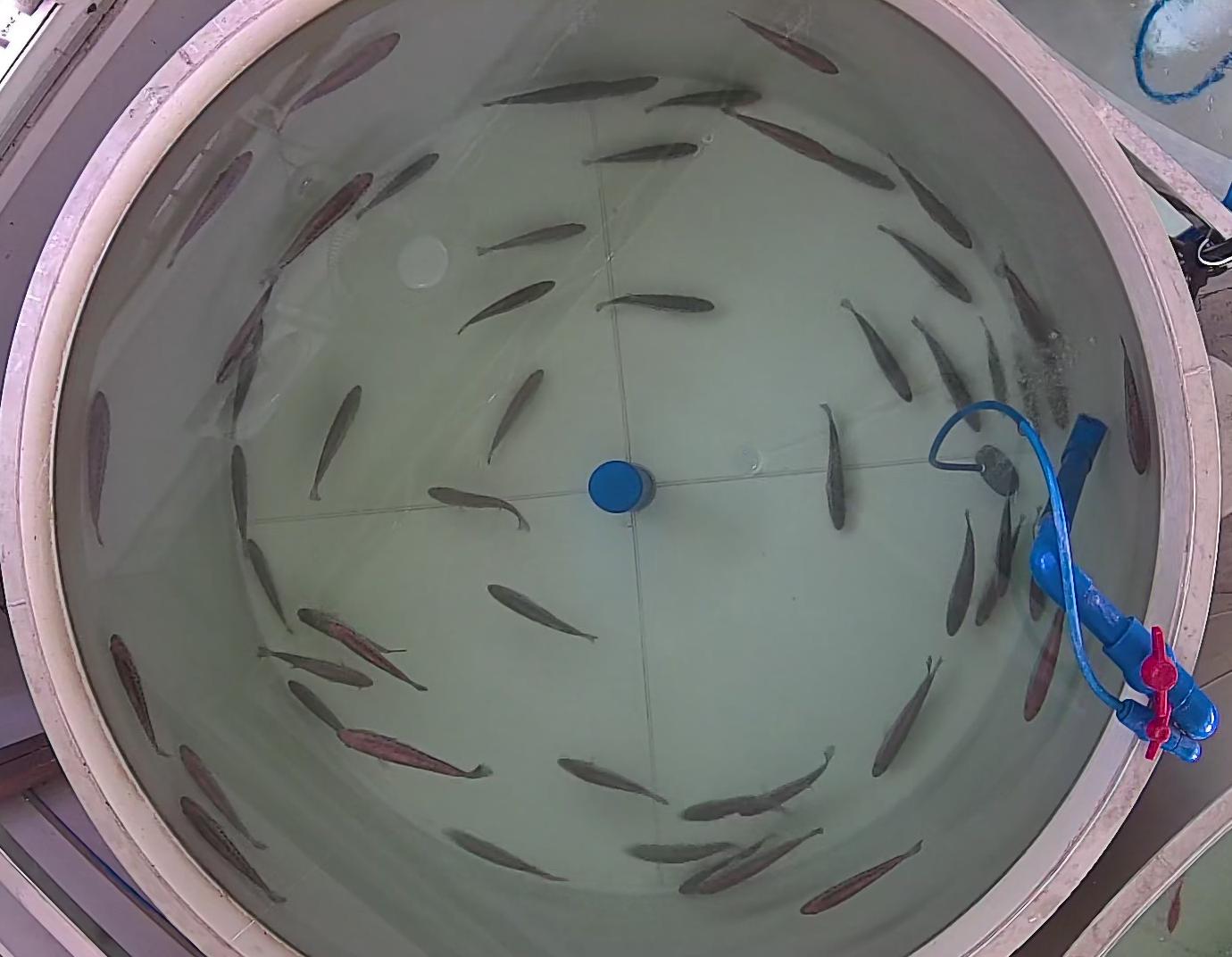

Supplement: S1 Dataset — (ZIP) [file pone.0283671.s001.zip › datasets/00049.jpg]

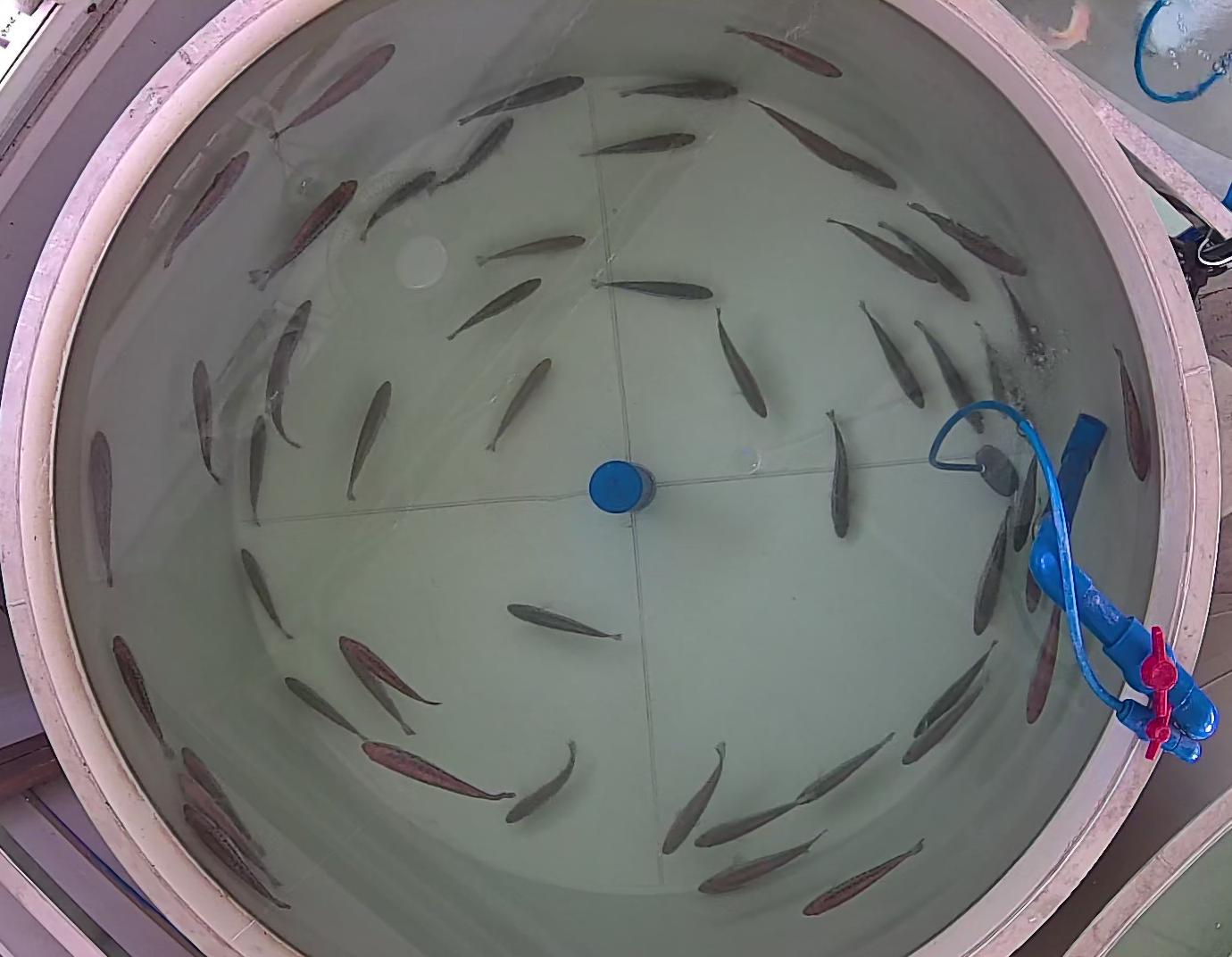

Supplement: S1 Dataset — (ZIP) [file pone.0283671.s001.zip › datasets/00050.jpg]

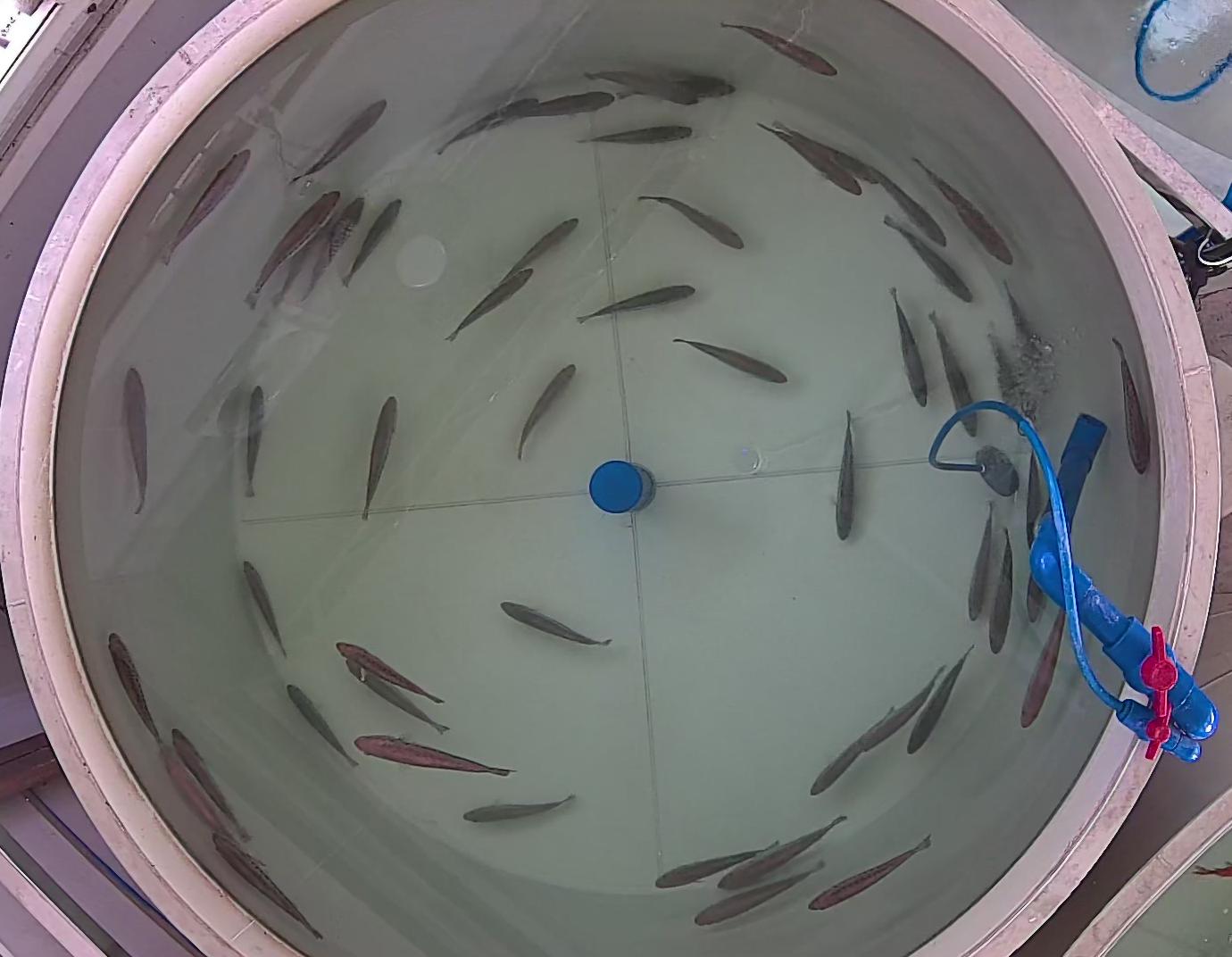

Supplement: S1 Dataset — (ZIP) [file pone.0283671.s001.zip › datasets/00051.jpg]

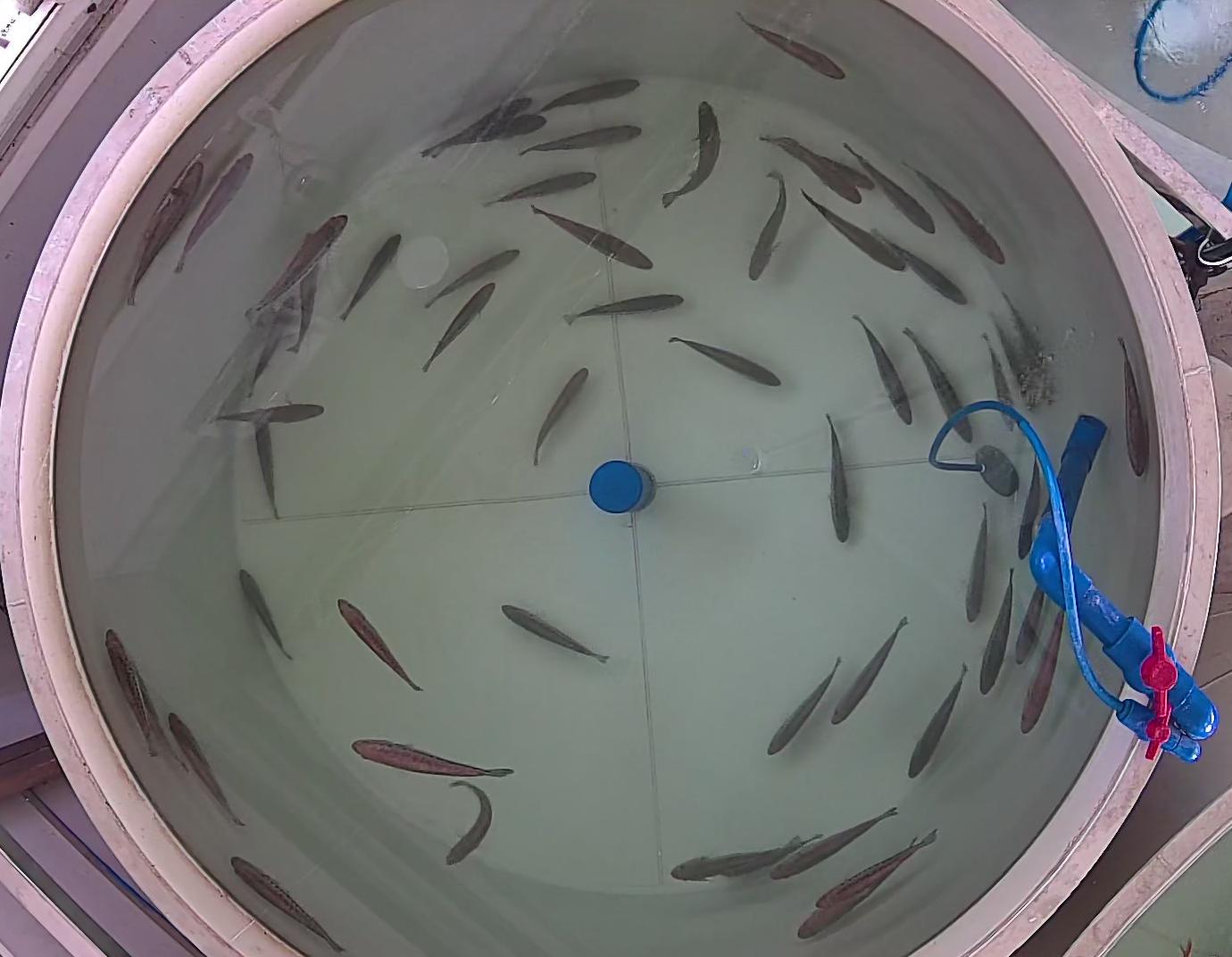

Supplement: S1 Dataset — (ZIP) [file pone.0283671.s001.zip › datasets/00052.jpg]

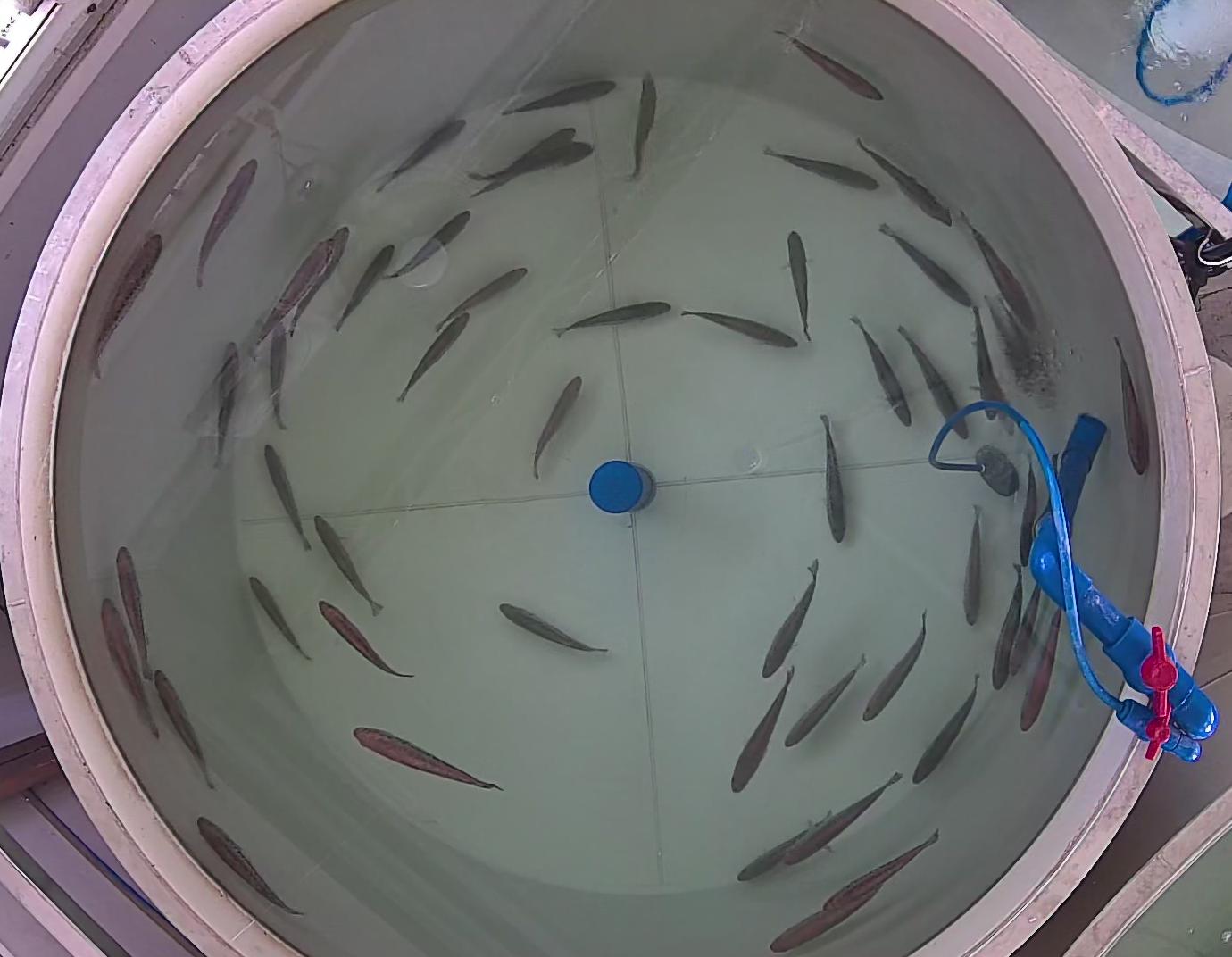

Supplement: S1 Dataset — (ZIP) [file pone.0283671.s001.zip › datasets/00053.jpg]

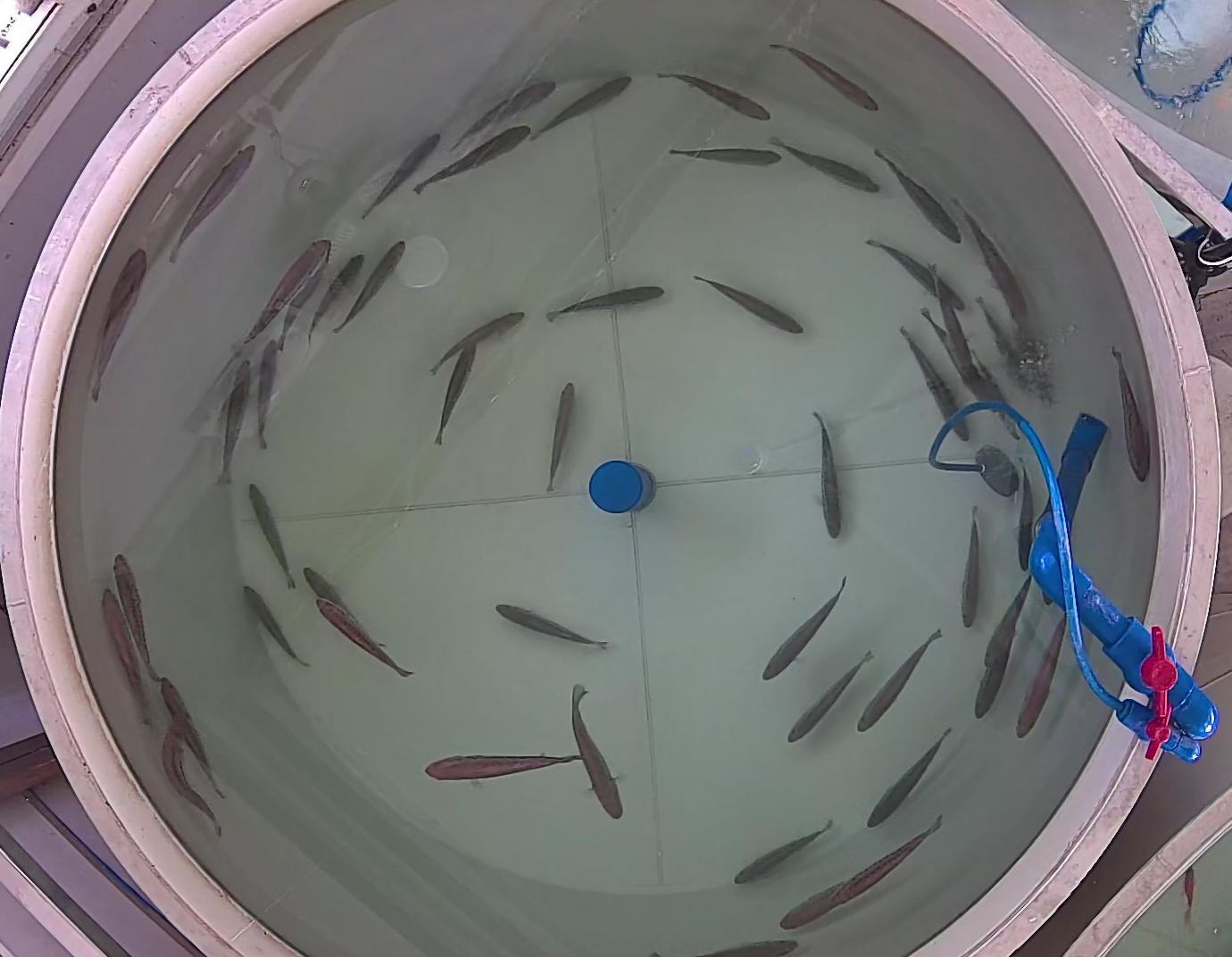

Supplement: S1 Dataset — (ZIP) [file pone.0283671.s001.zip › datasets/00054.jpg]

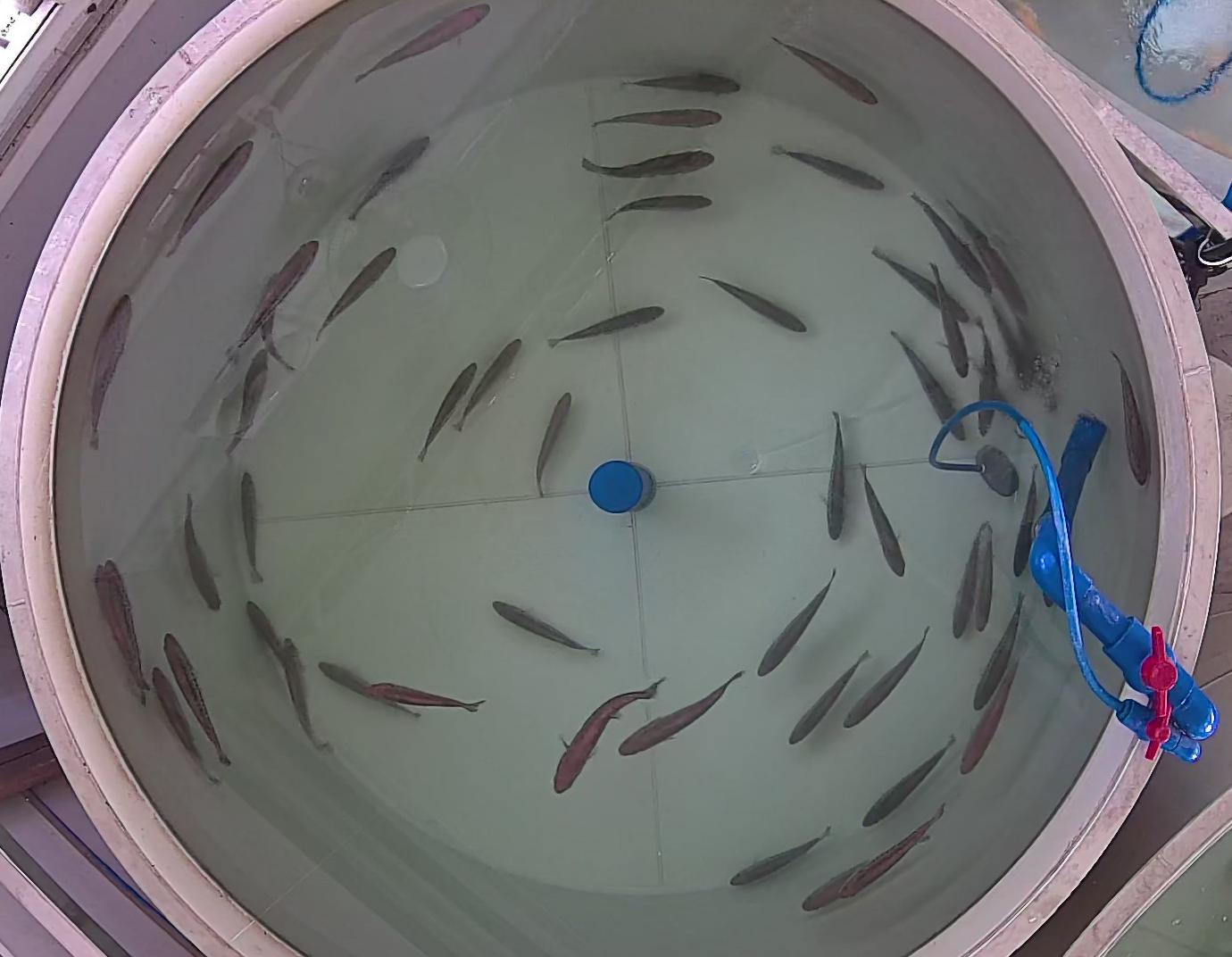

Supplement: S1 Dataset — (ZIP) [file pone.0283671.s001.zip › datasets/00055.jpg]

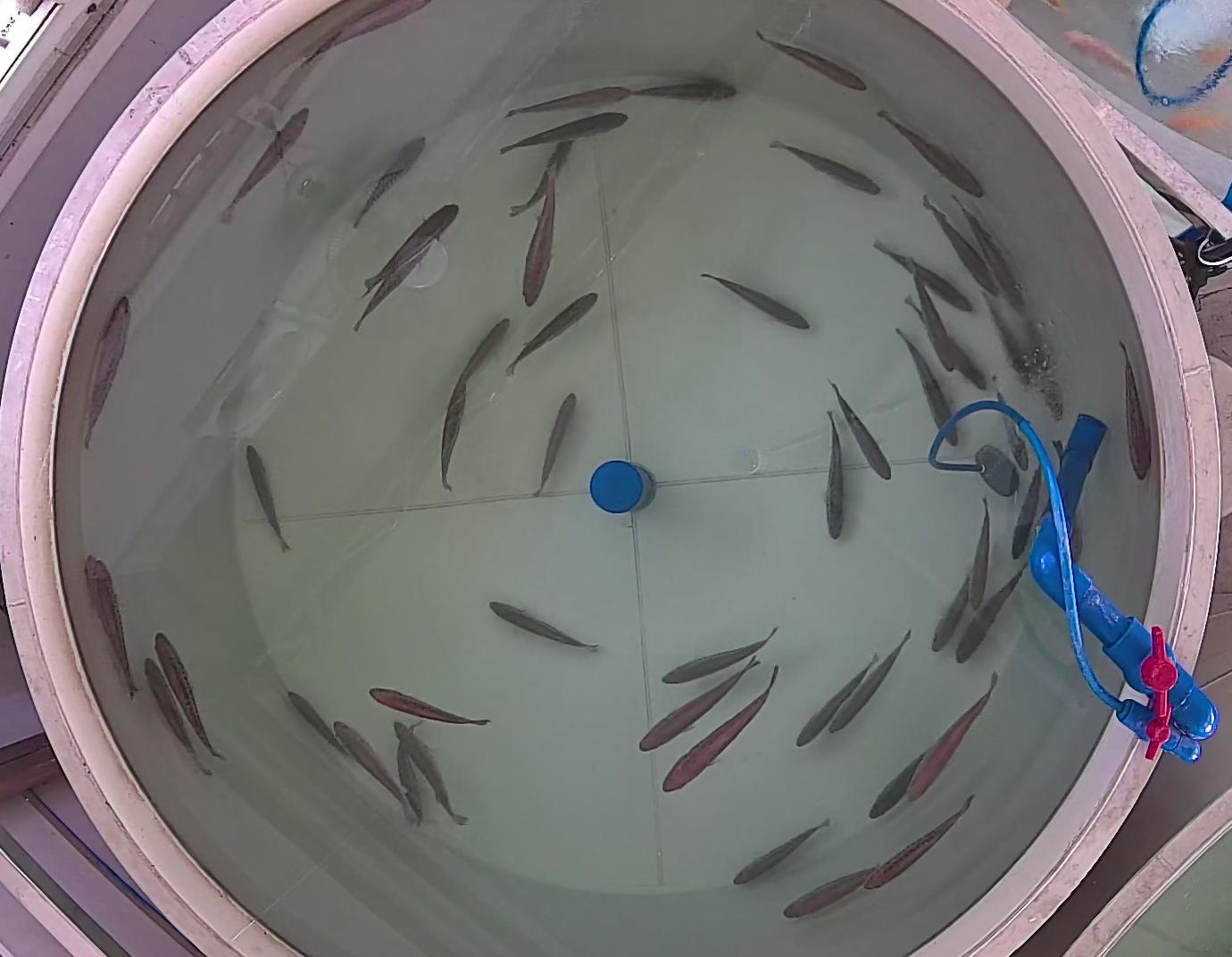

Supplement: S1 Dataset — (ZIP) [file pone.0283671.s001.zip › datasets/00056.jpg]

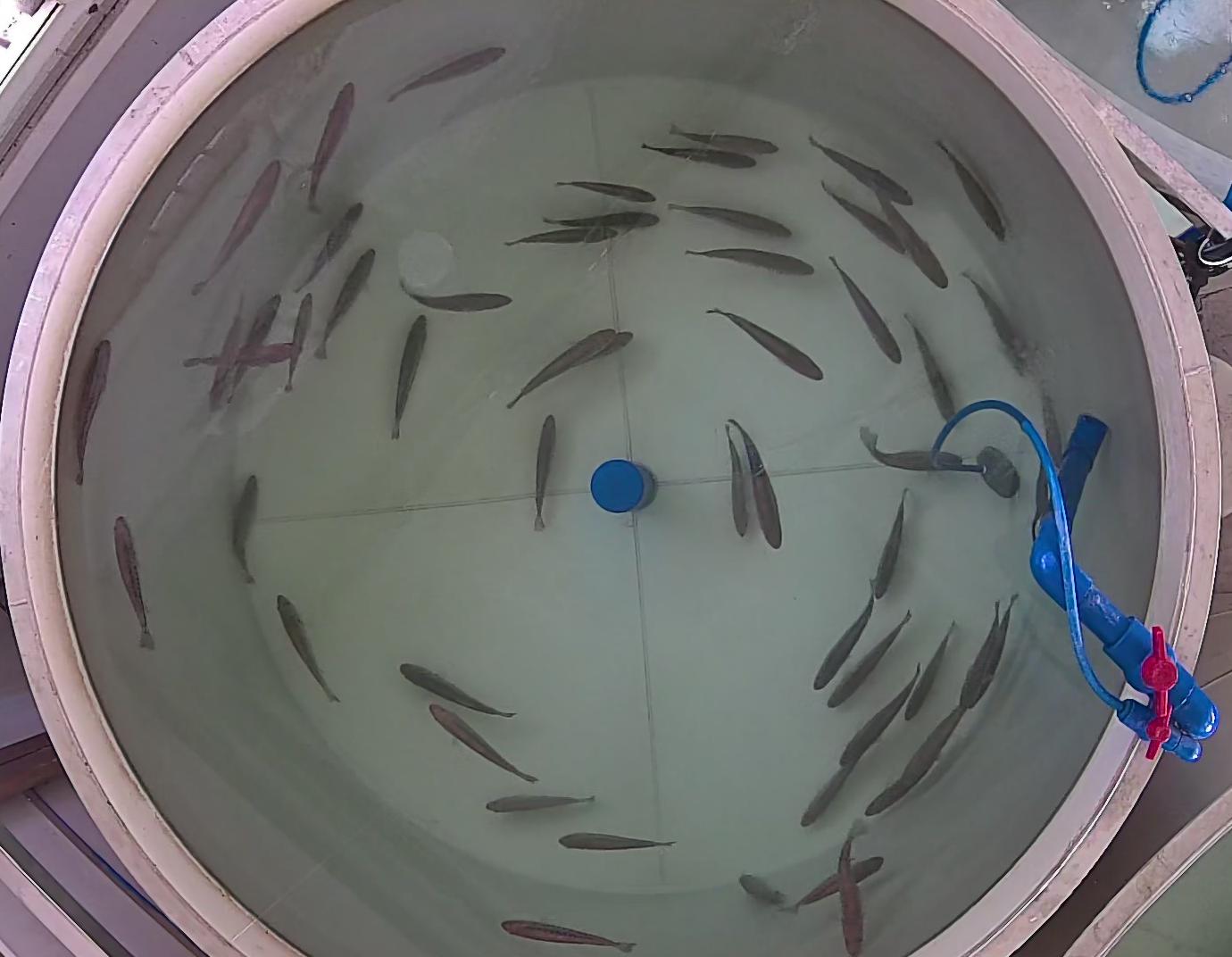

Supplement: S1 Dataset — (ZIP) [file pone.0283671.s001.zip › datasets/00057.jpg]

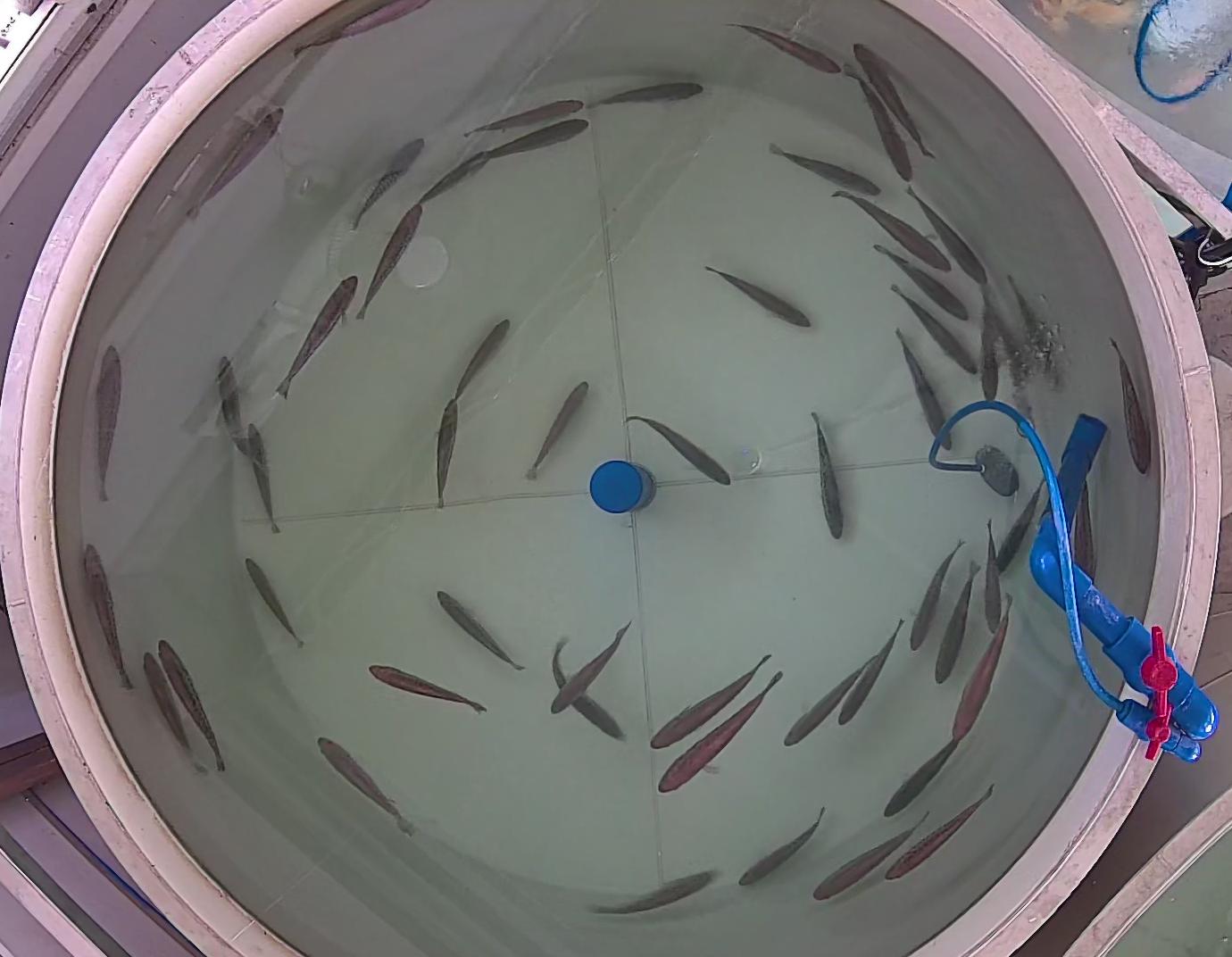

Supplement: S1 Dataset — (ZIP) [file pone.0283671.s001.zip › datasets/00058.jpg]

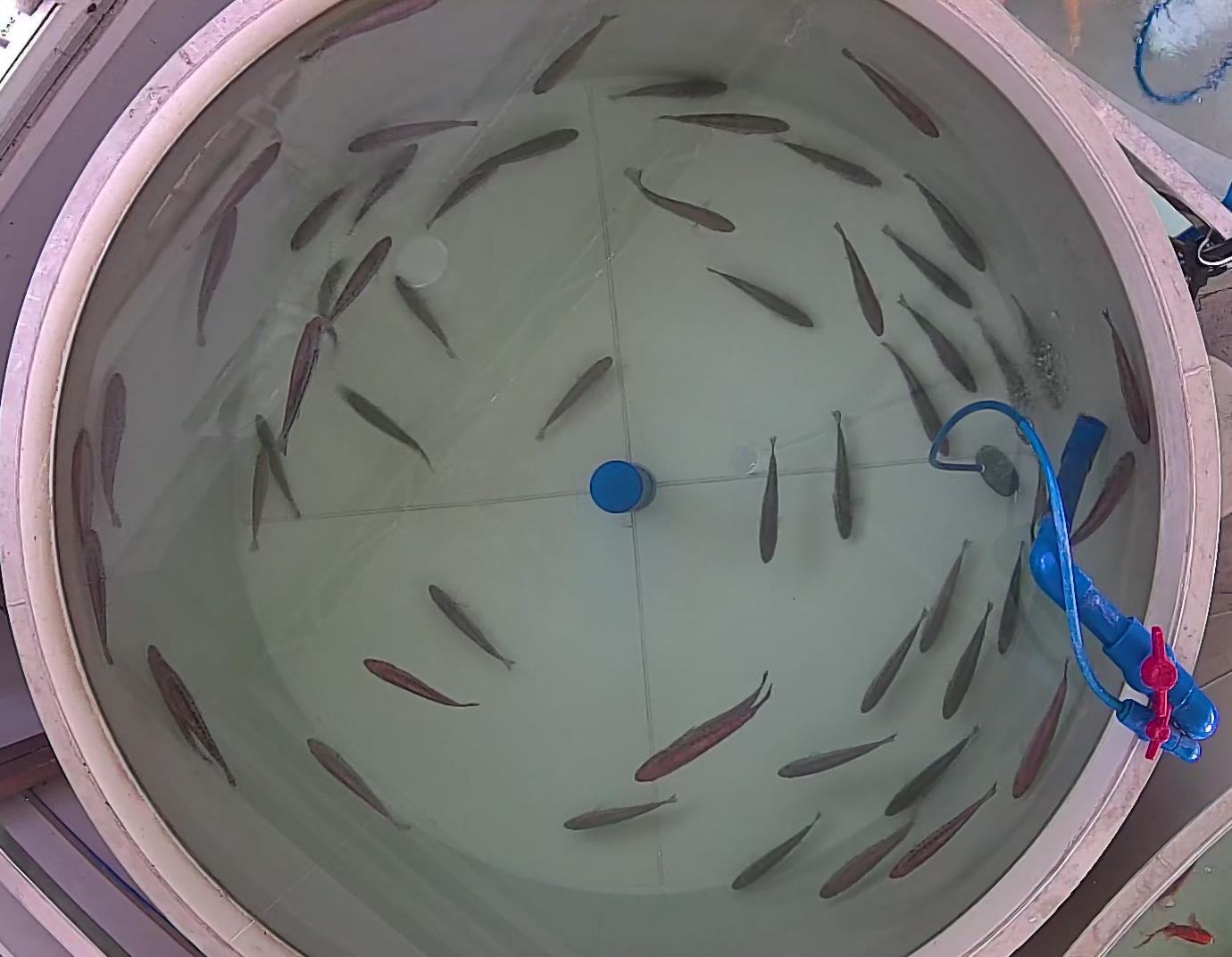

Supplement: S1 Dataset — (ZIP) [file pone.0283671.s001.zip › datasets/00059.jpg]

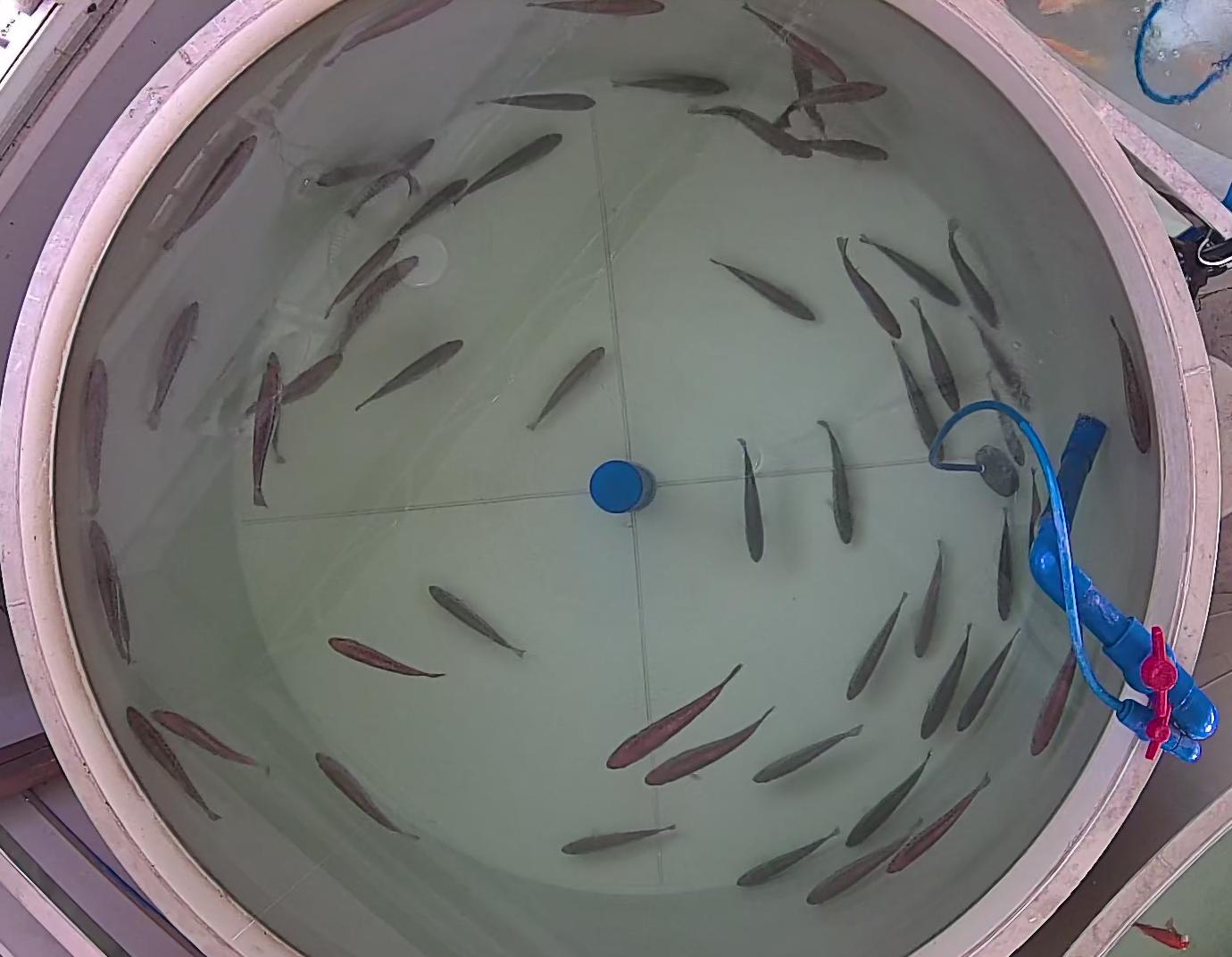

Supplement: S1 Dataset — (ZIP) [file pone.0283671.s001.zip › datasets/00060.jpg]

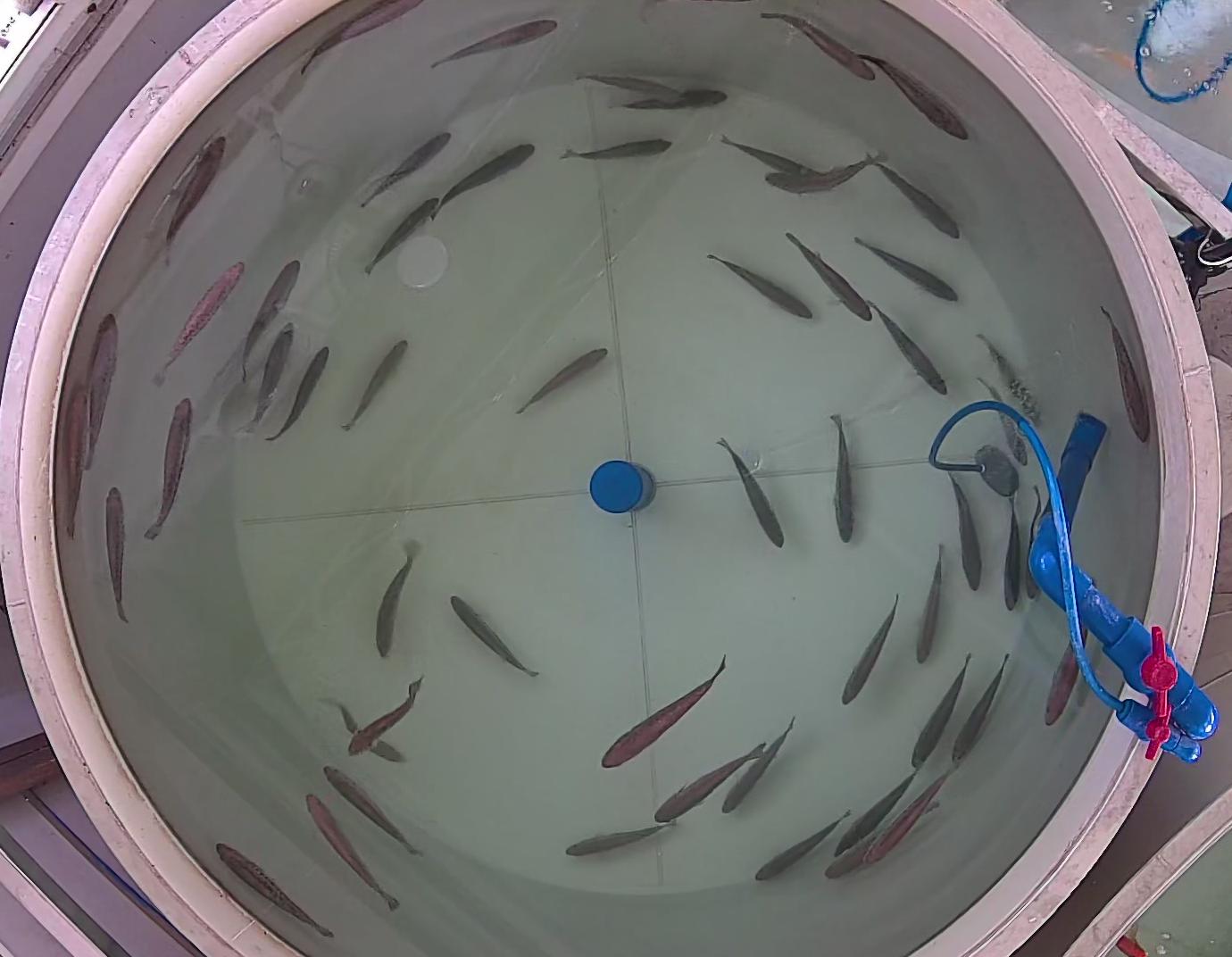

Supplement: S1 Dataset — (ZIP) [file pone.0283671.s001.zip › datasets/00061.jpg]

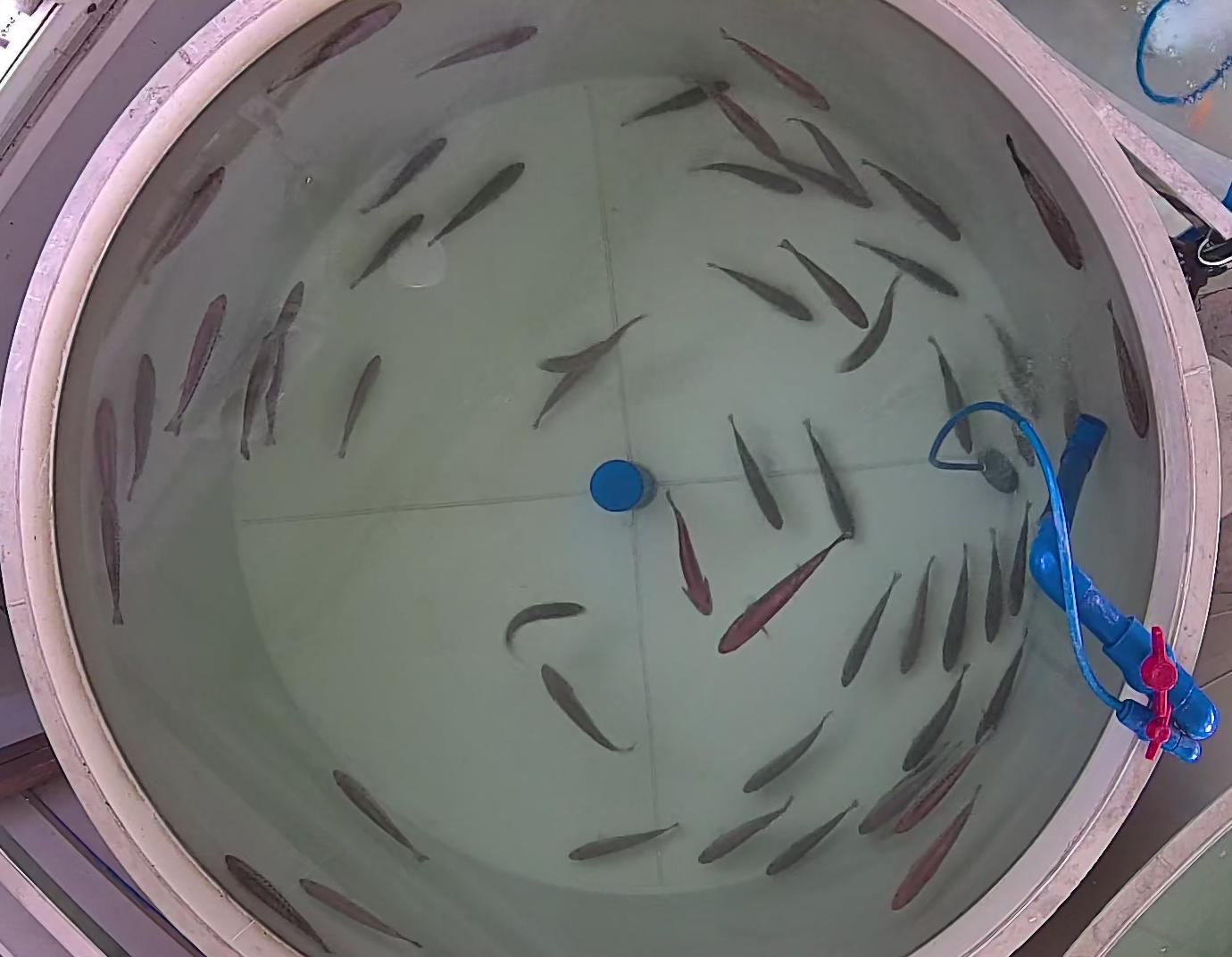

Supplement: S1 Dataset — (ZIP) [file pone.0283671.s001.zip › datasets/00062.jpg]

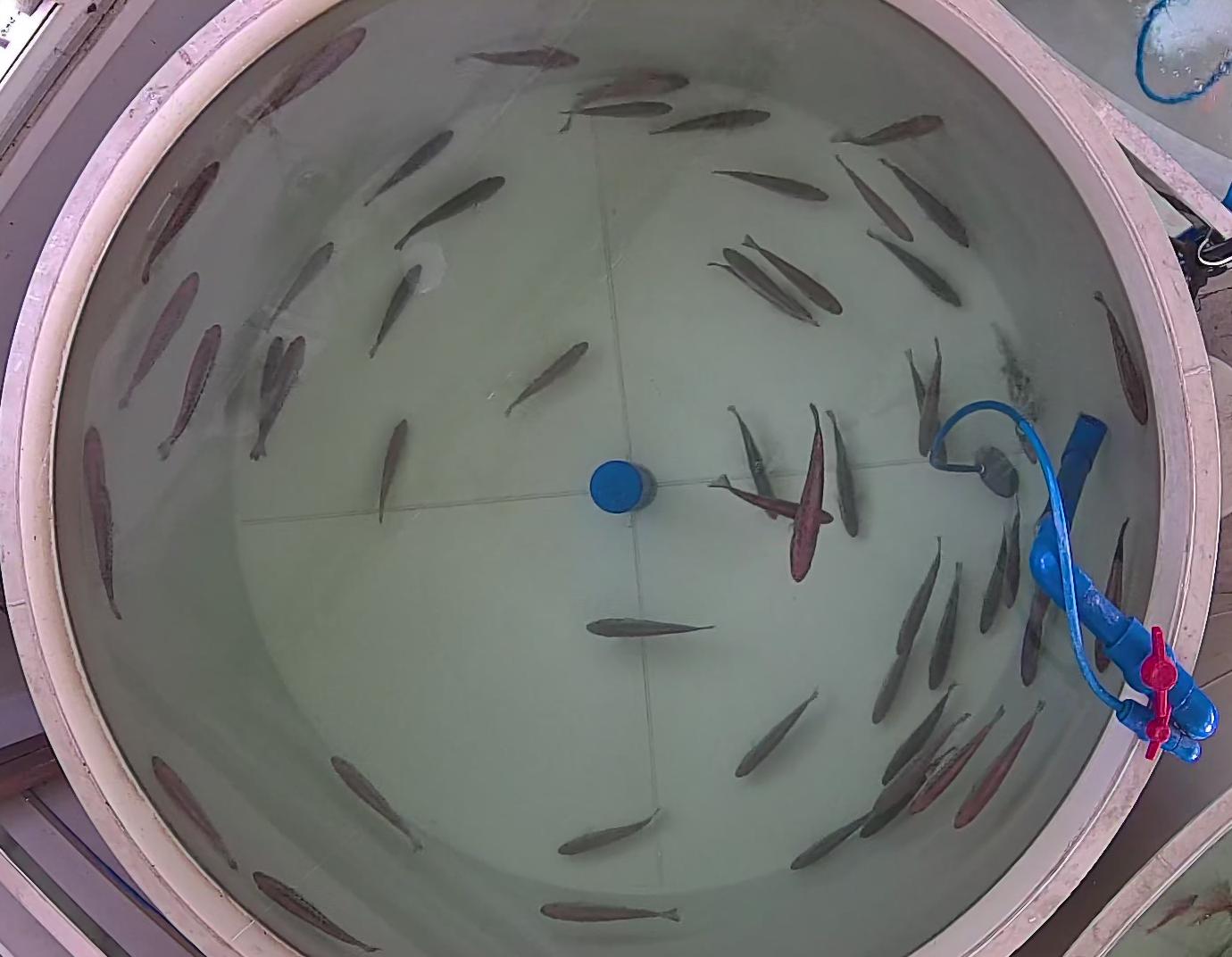

Supplement: S1 Dataset — (ZIP) [file pone.0283671.s001.zip › datasets/00063.jpg]

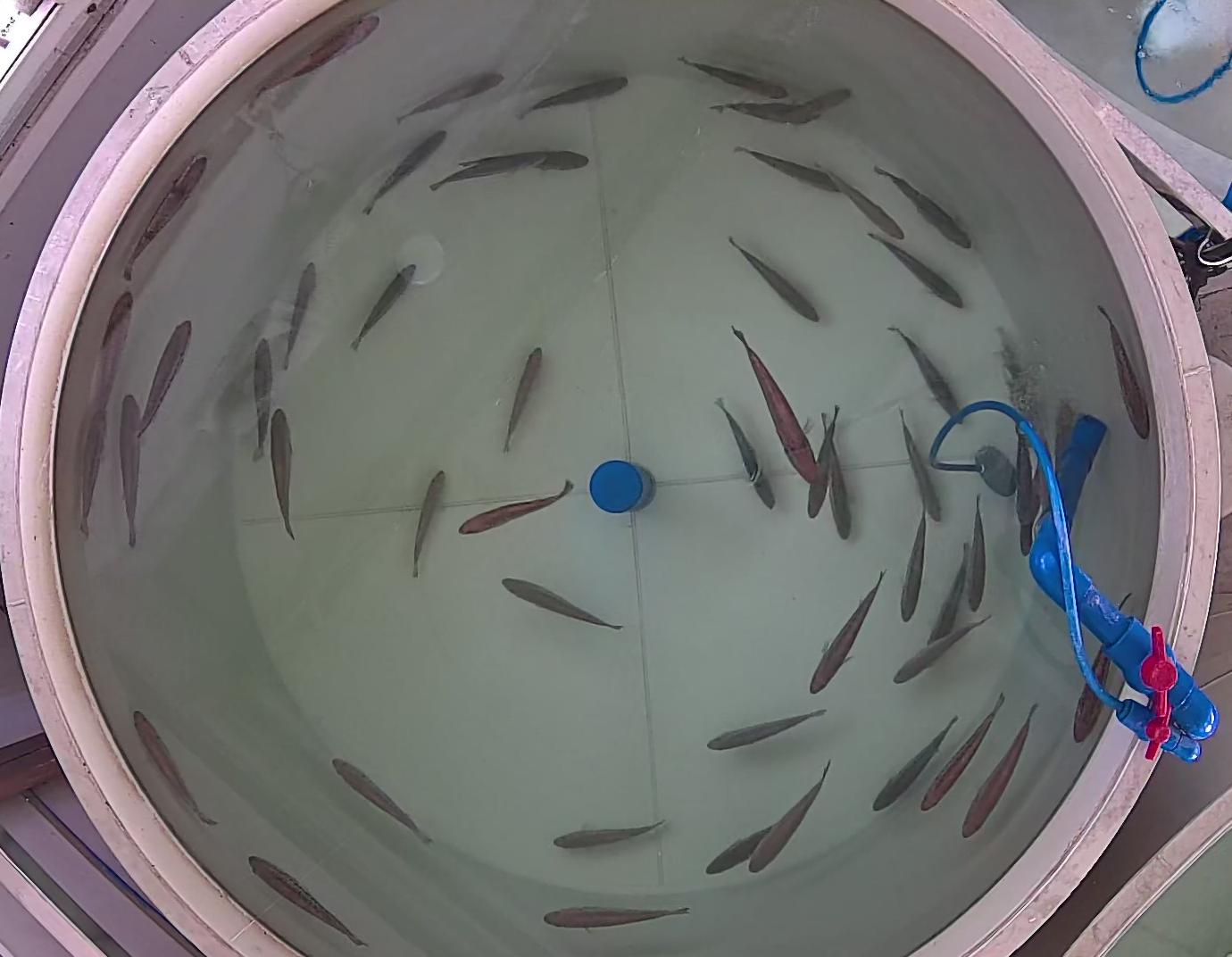

Supplement: S1 Dataset — (ZIP) [file pone.0283671.s001.zip › datasets/00064.jpg]

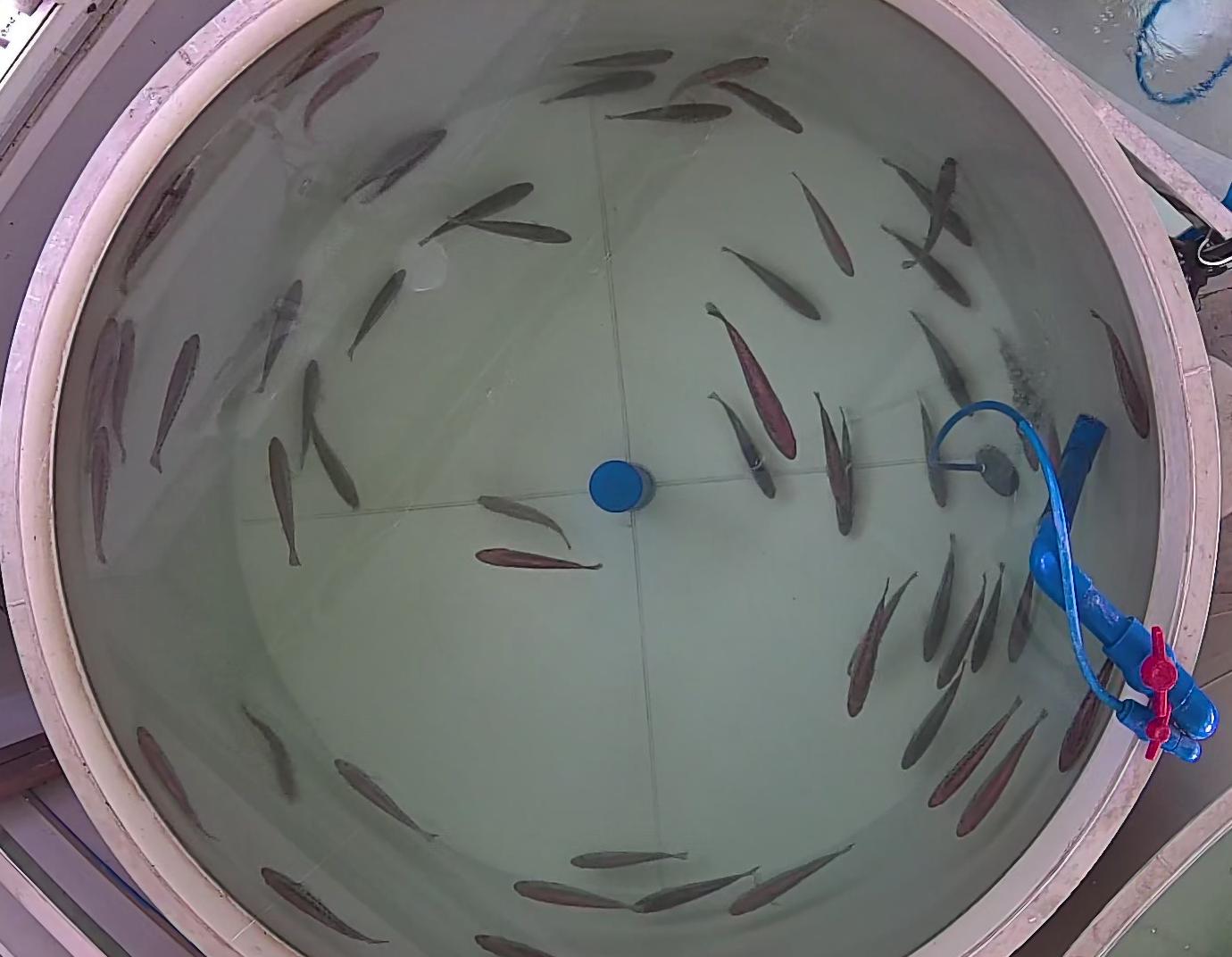

Supplement: S1 Dataset — (ZIP) [file pone.0283671.s001.zip › datasets/00065.jpg]

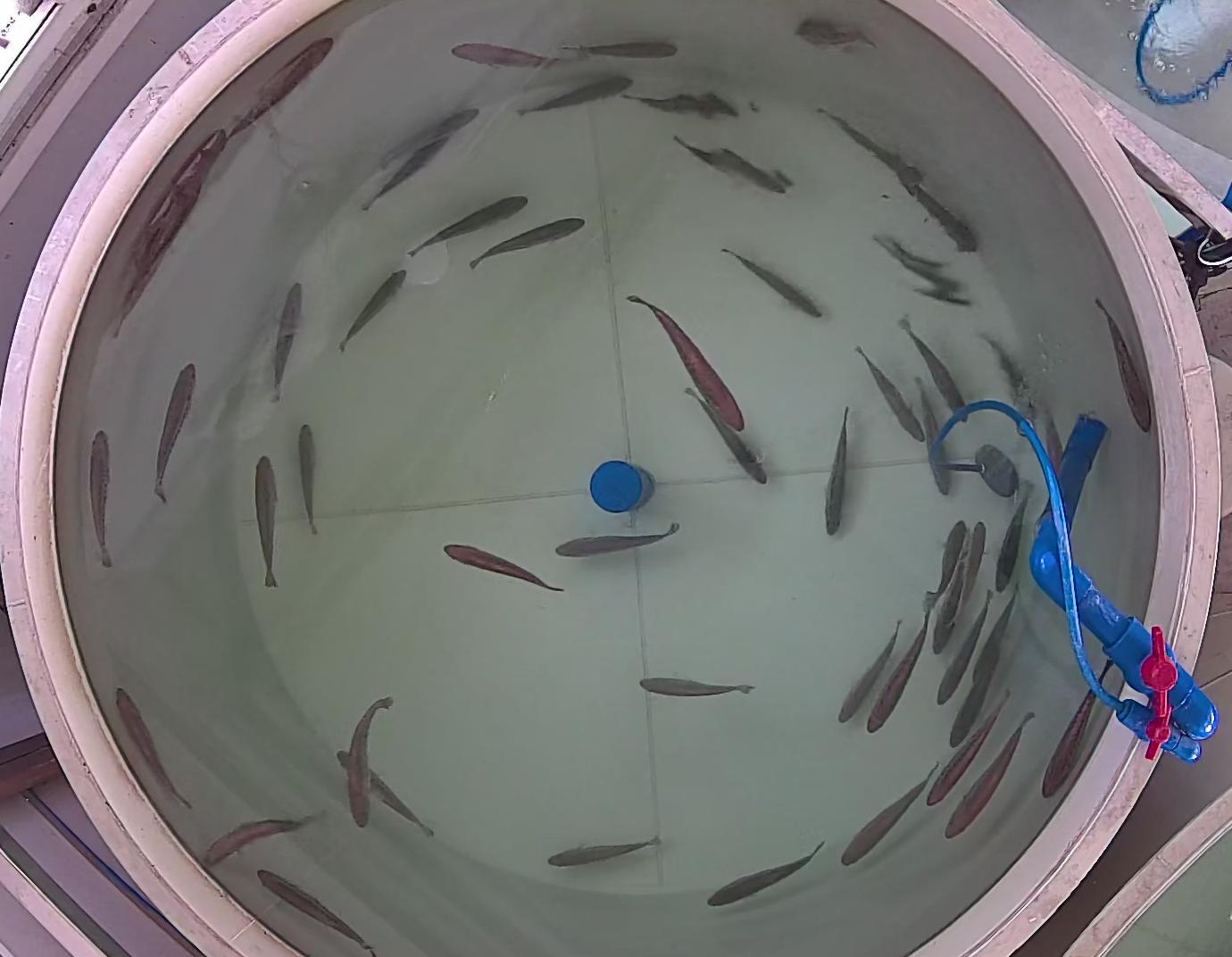

Supplement: S1 Dataset — (ZIP) [file pone.0283671.s001.zip › datasets/00066.jpg]

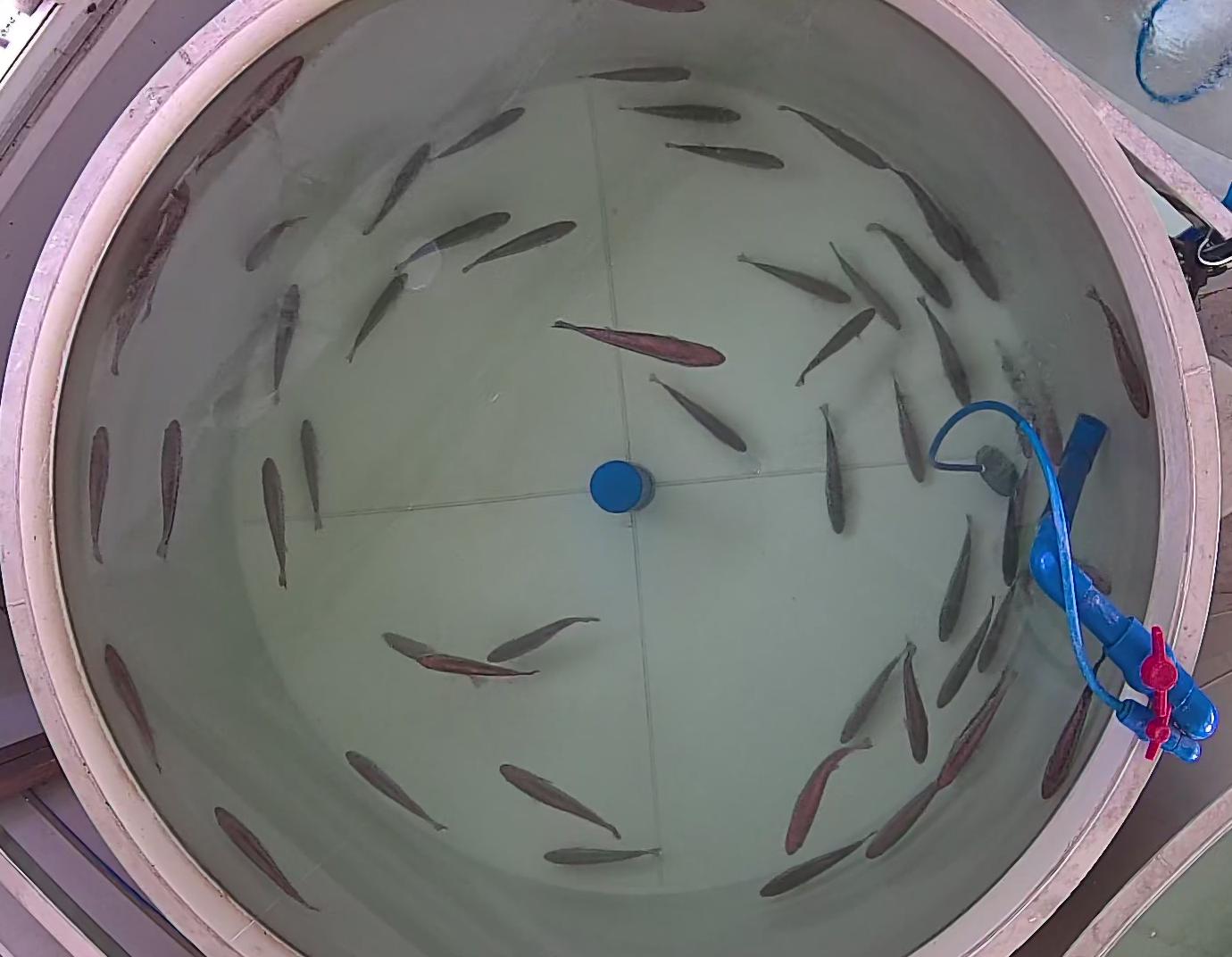

Supplement: S1 Dataset — (ZIP) [file pone.0283671.s001.zip › datasets/00067.jpg]

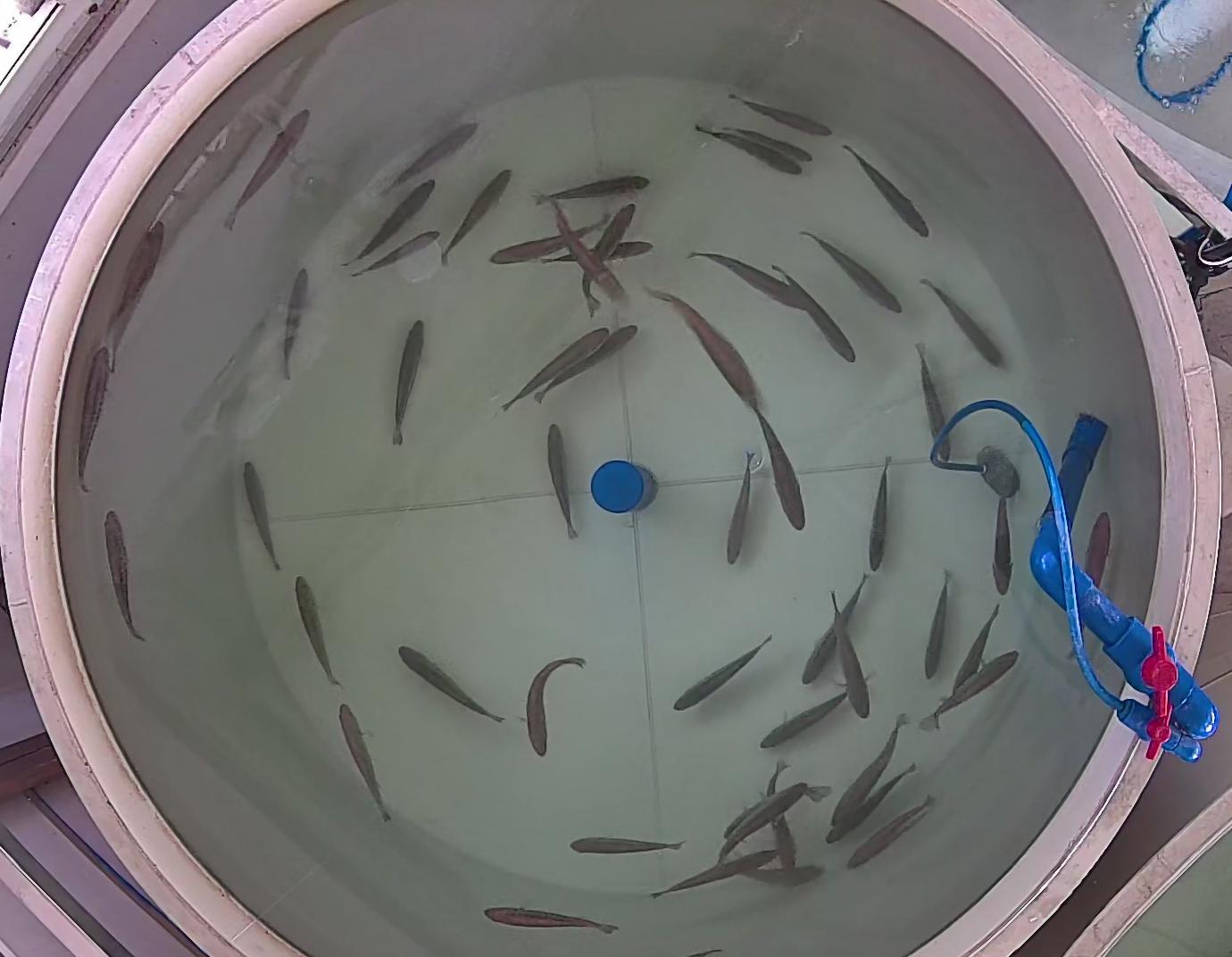

Supplement: S1 Dataset — (ZIP) [file pone.0283671.s001.zip › datasets/00068.jpg]

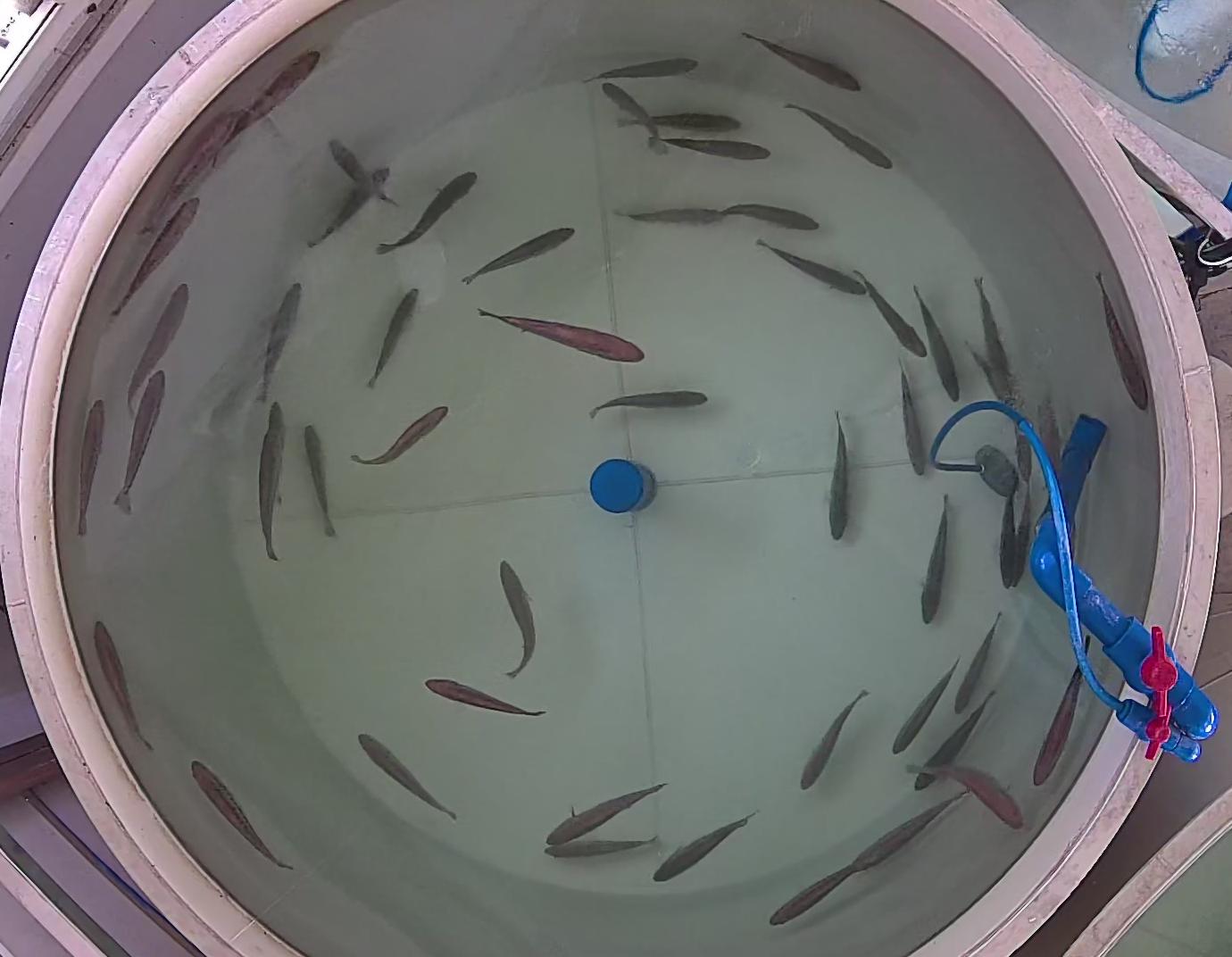

Supplement: S1 Dataset — (ZIP) [file pone.0283671.s001.zip › datasets/00069.jpg]

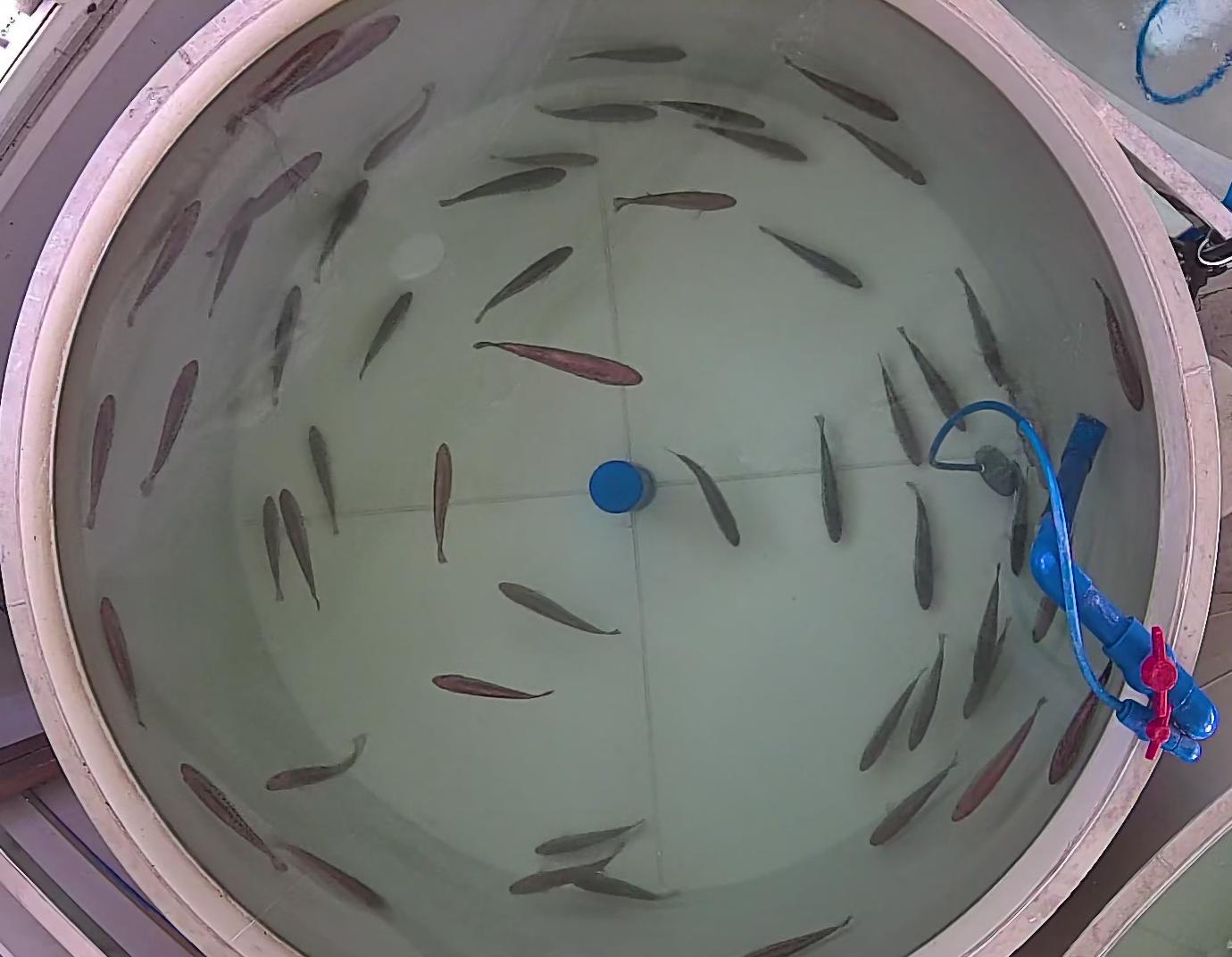

Supplement: S1 Dataset — (ZIP) [file pone.0283671.s001.zip › datasets/00070.jpg]

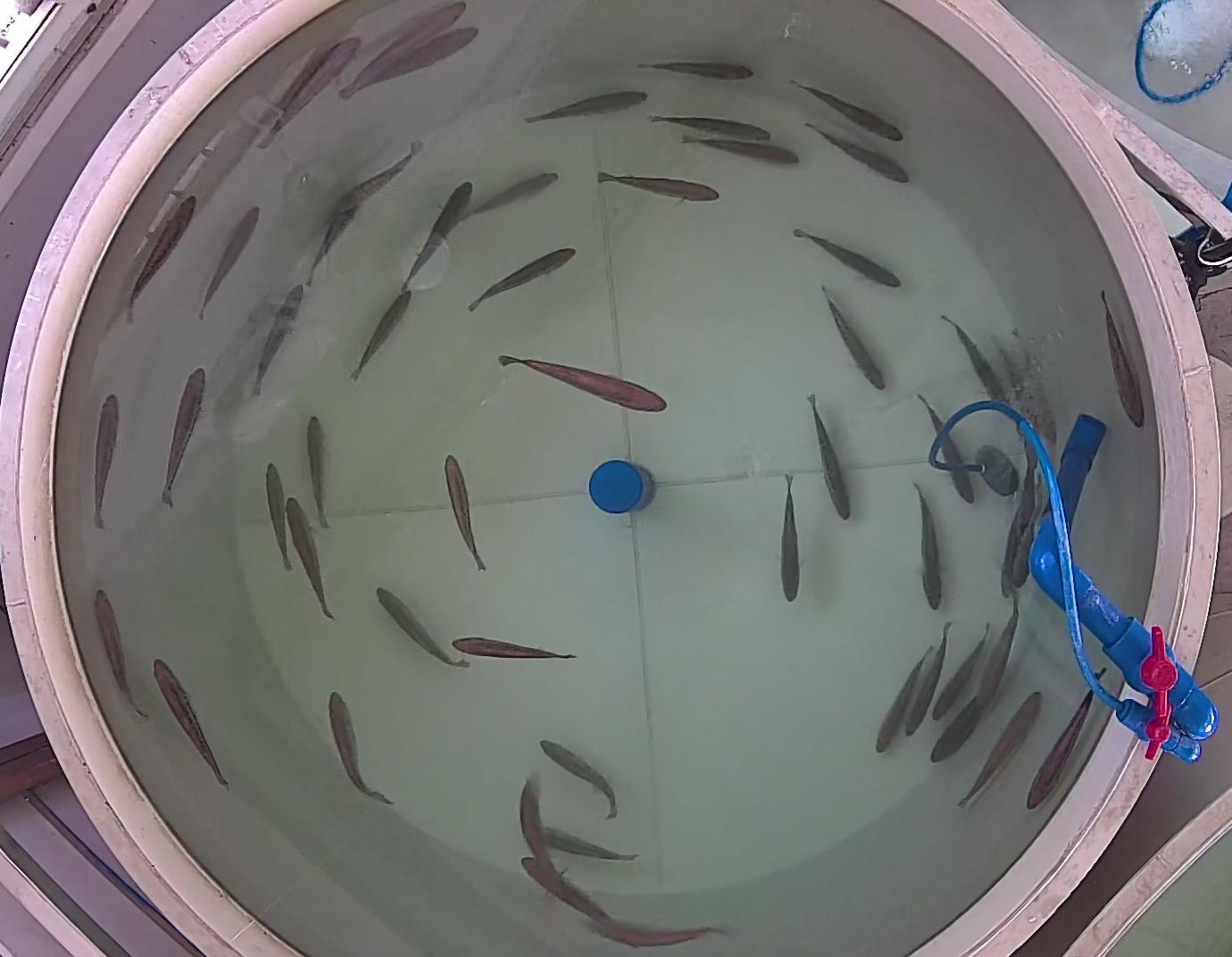

Supplement: S1 Dataset — (ZIP) [file pone.0283671.s001.zip › datasets/00071.jpg]

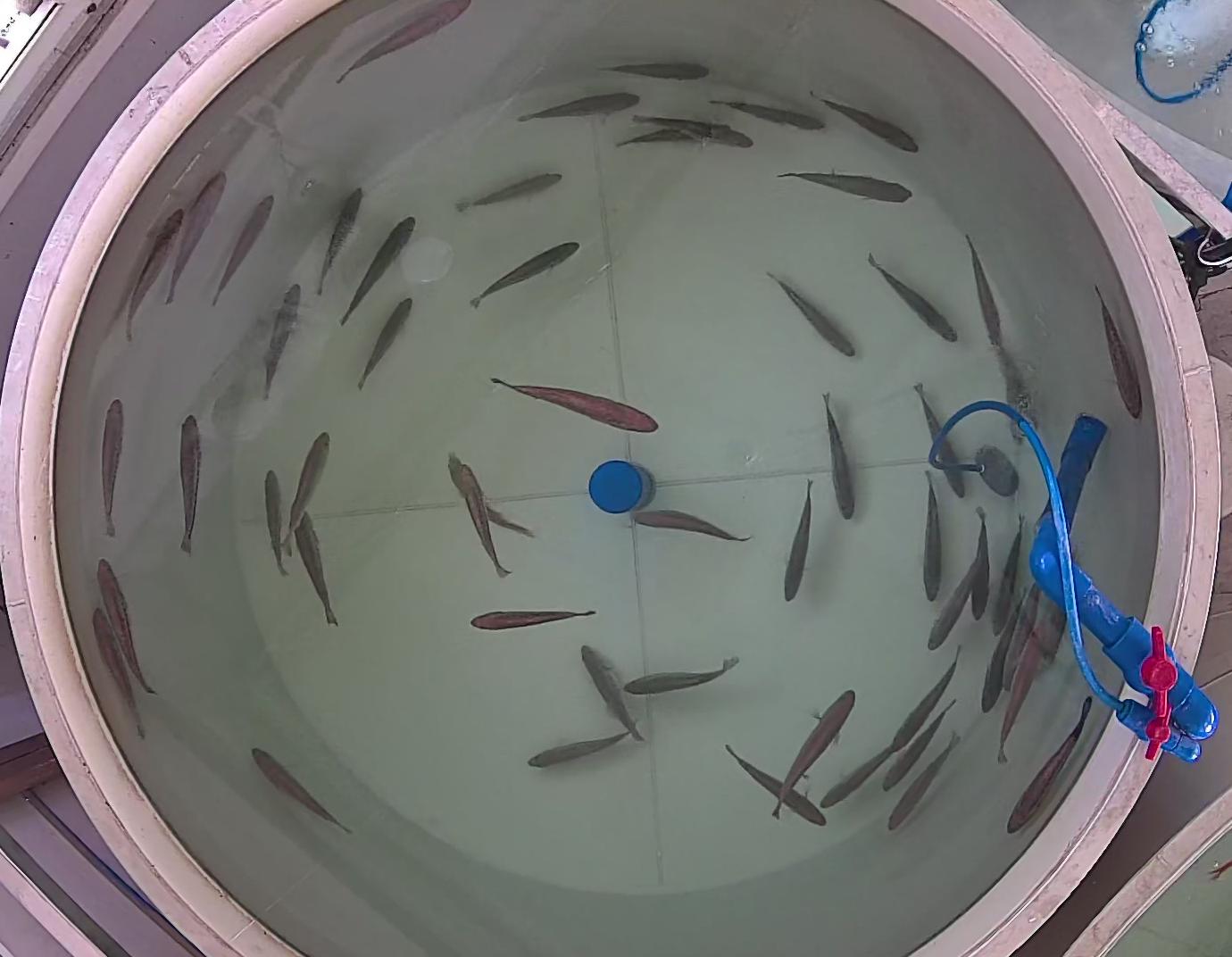

Supplement: S1 Dataset — (ZIP) [file pone.0283671.s001.zip › datasets/00072.jpg]

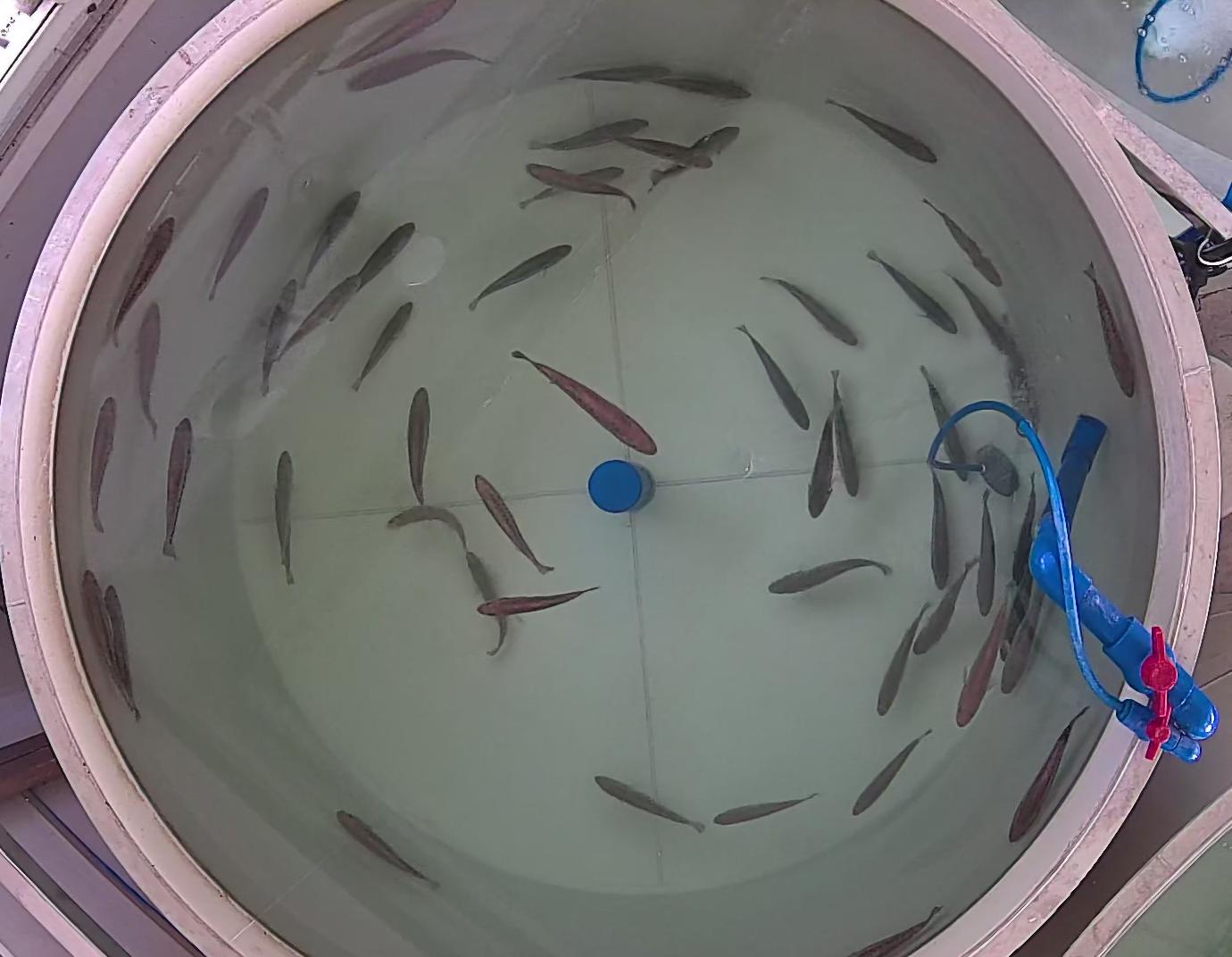

Supplement: S1 Dataset — (ZIP) [file pone.0283671.s001.zip › datasets/00073.jpg]

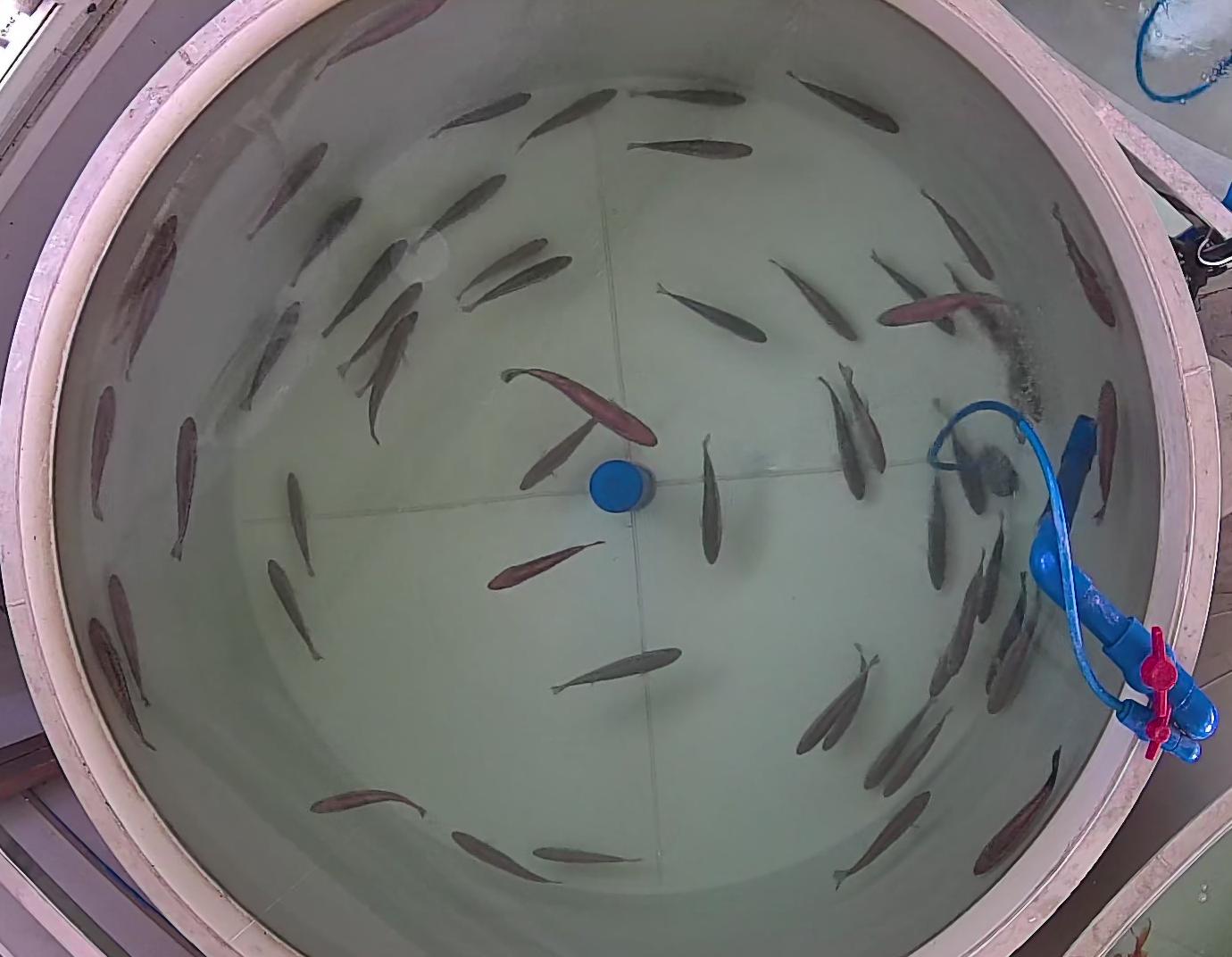

Supplement: S1 Dataset — (ZIP) [file pone.0283671.s001.zip › datasets/00074.jpg]

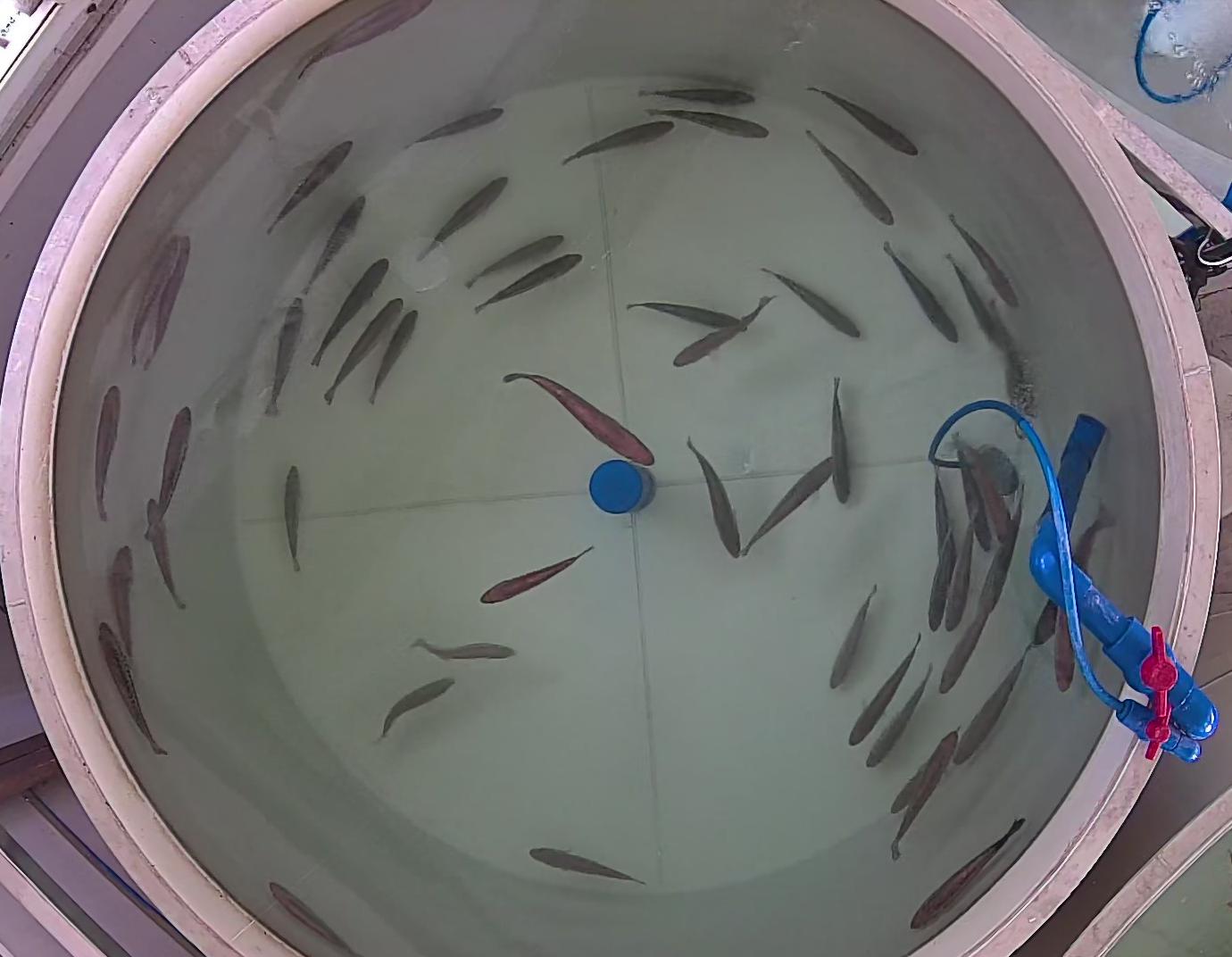

Supplement: S1 Dataset — (ZIP) [file pone.0283671.s001.zip › datasets/00075.jpg]

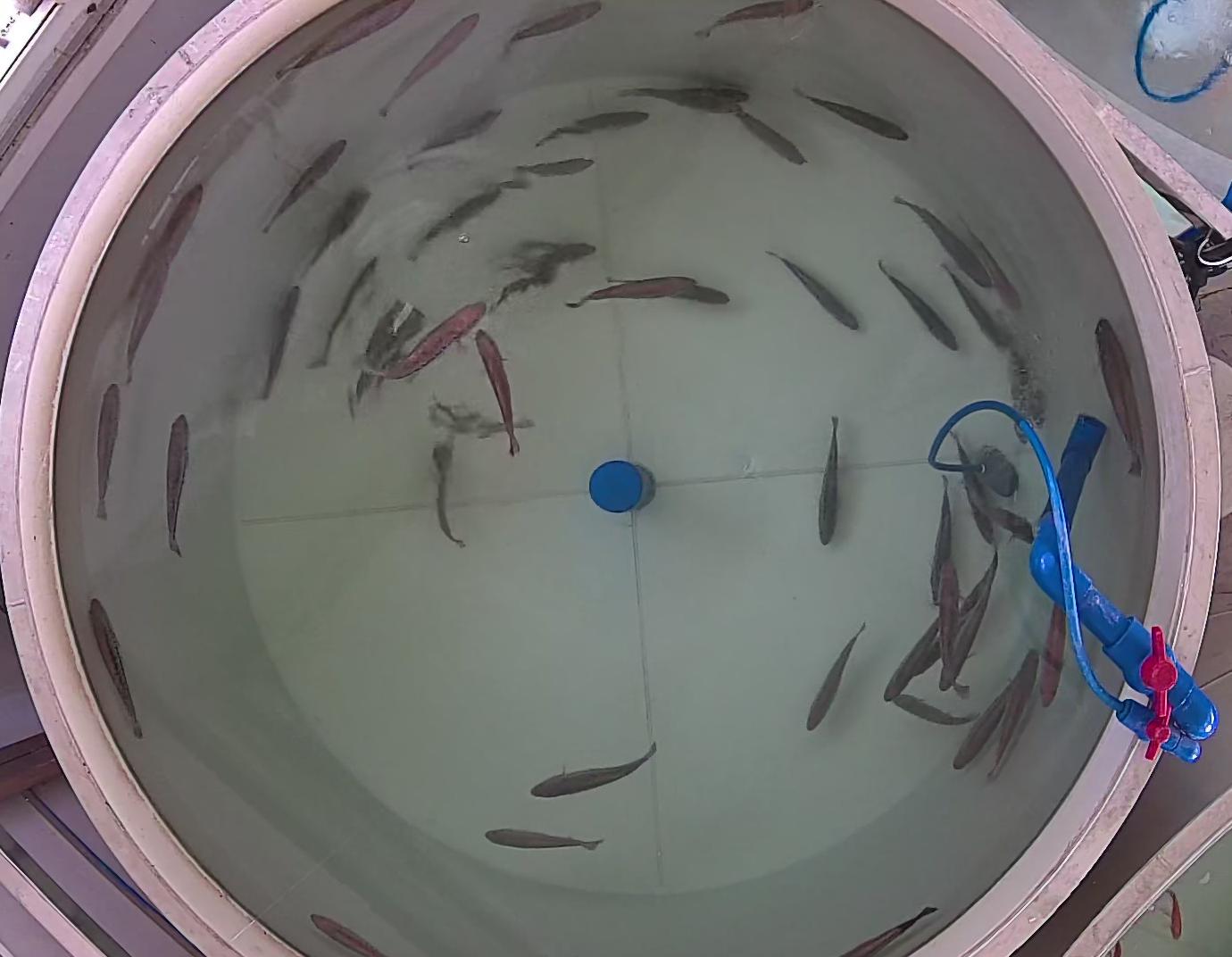

Supplement: S1 Dataset — (ZIP) [file pone.0283671.s001.zip › datasets/00076.jpg]

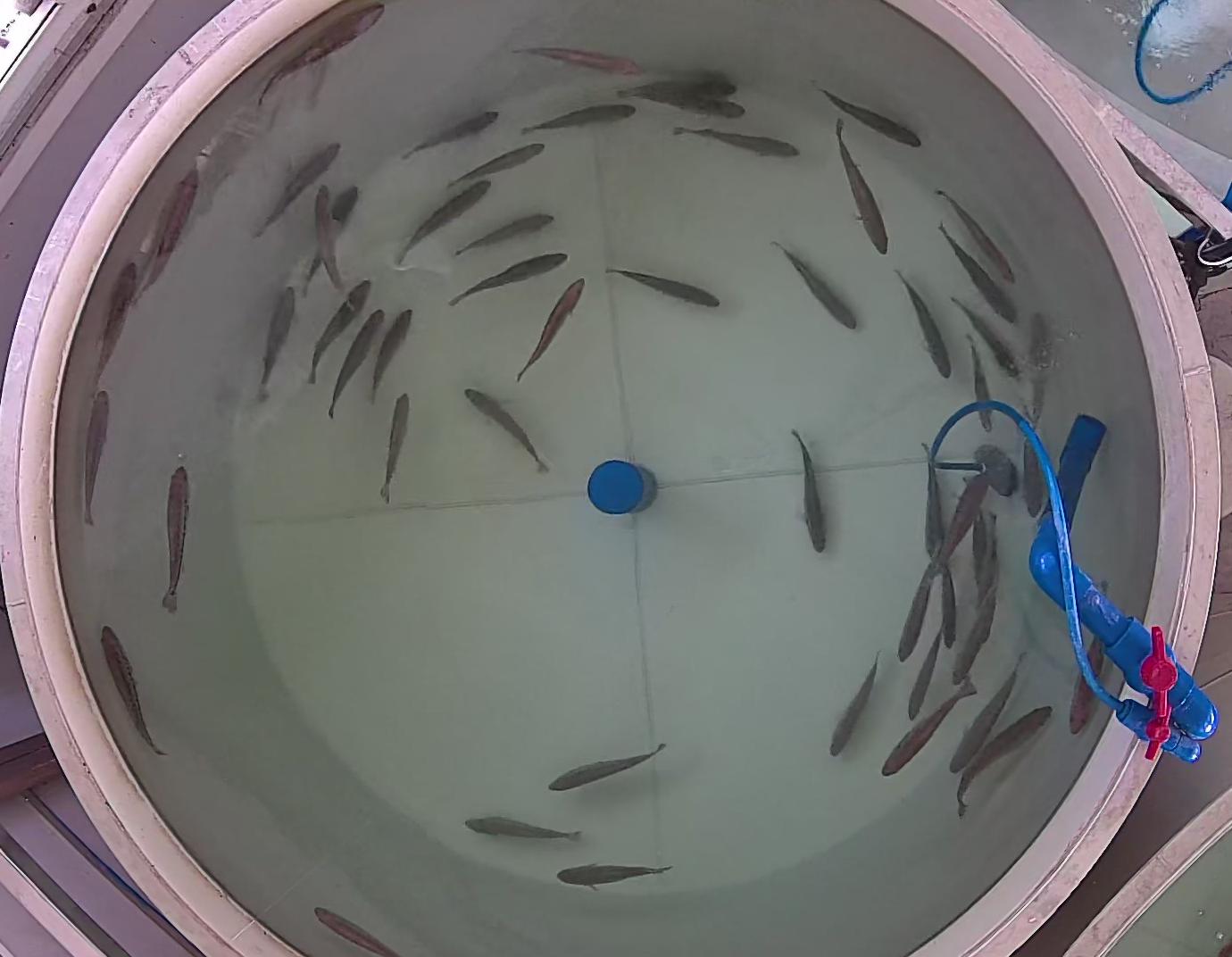

Supplement: S1 Dataset — (ZIP) [file pone.0283671.s001.zip › datasets/00077.jpg]

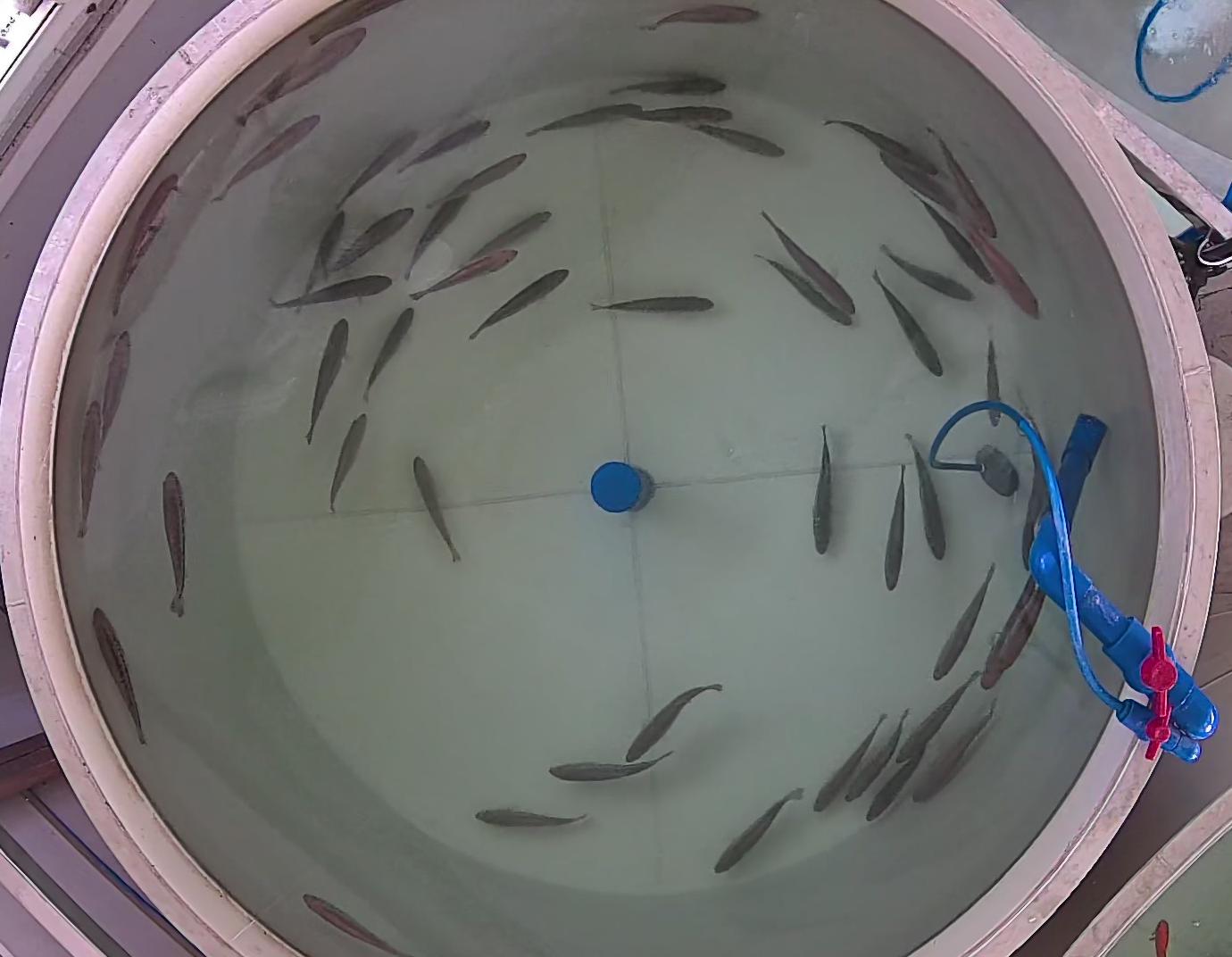

Supplement: S1 Dataset — (ZIP) [file pone.0283671.s001.zip › datasets/00078.jpg]

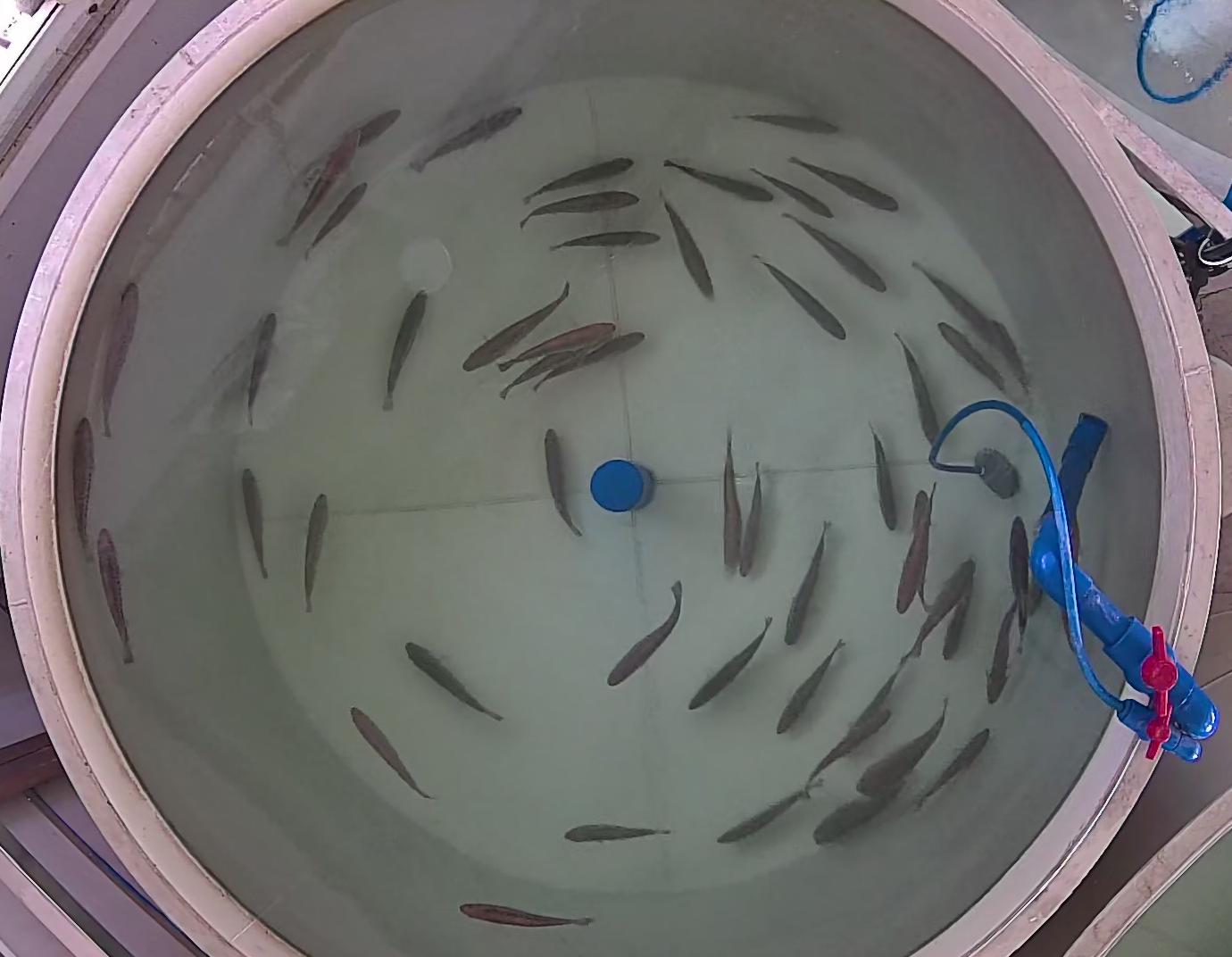

Supplement: S1 Dataset — (ZIP) [file pone.0283671.s001.zip › datasets/00079.jpg]

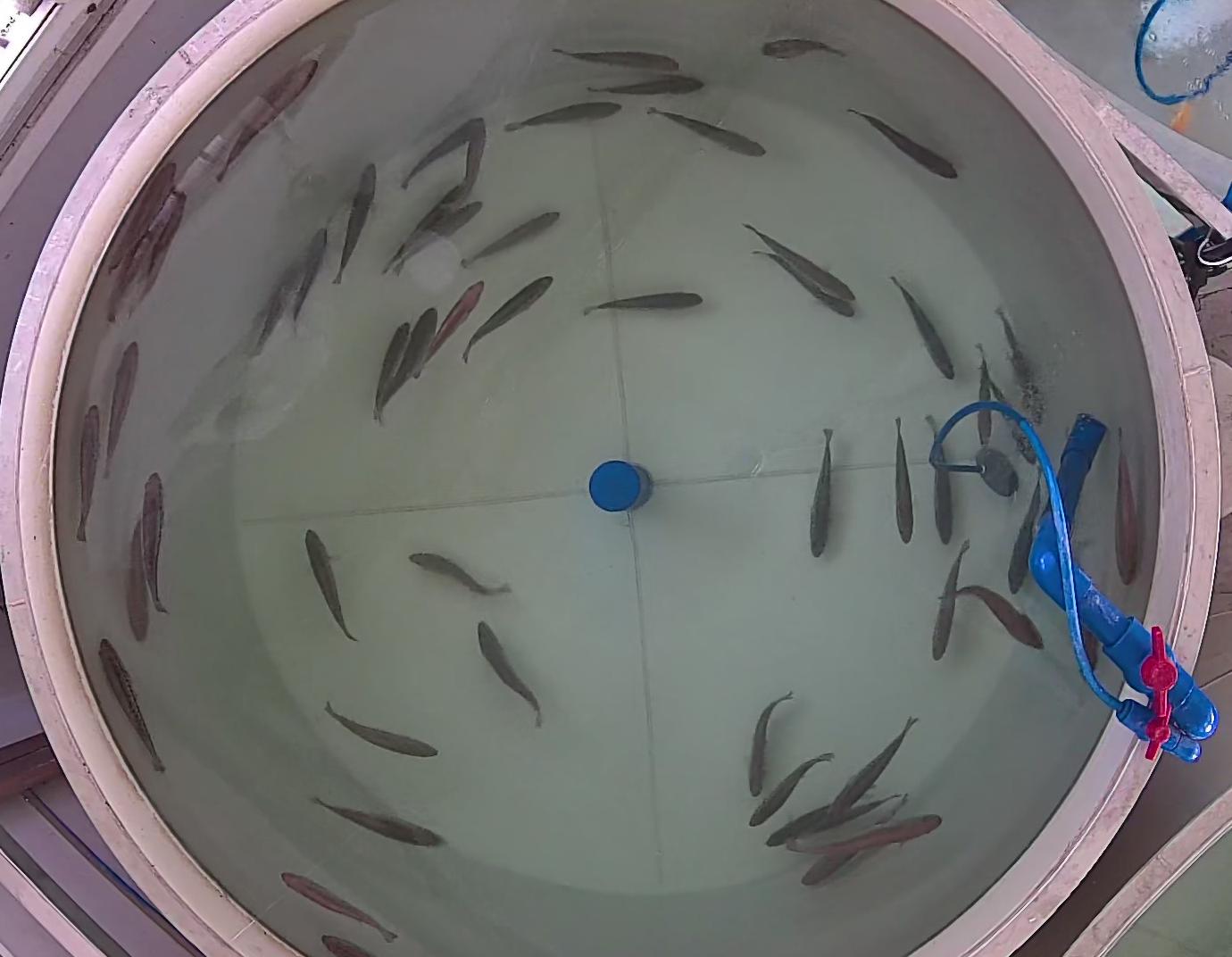

Supplement: S1 Dataset — (ZIP) [file pone.0283671.s001.zip › datasets/00080.jpg]

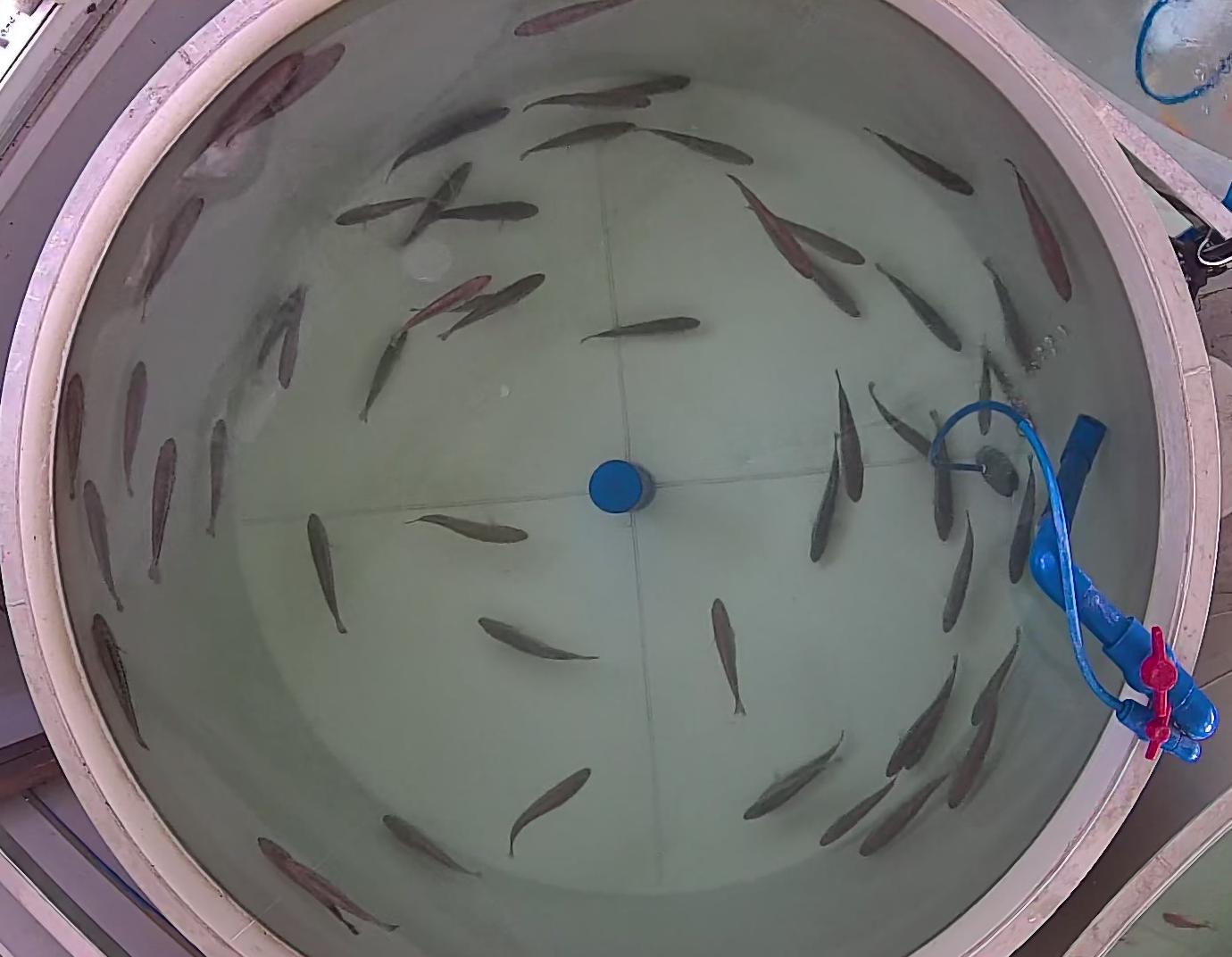

Supplement: S1 Dataset — (ZIP) [file pone.0283671.s001.zip › datasets/00081.jpg]

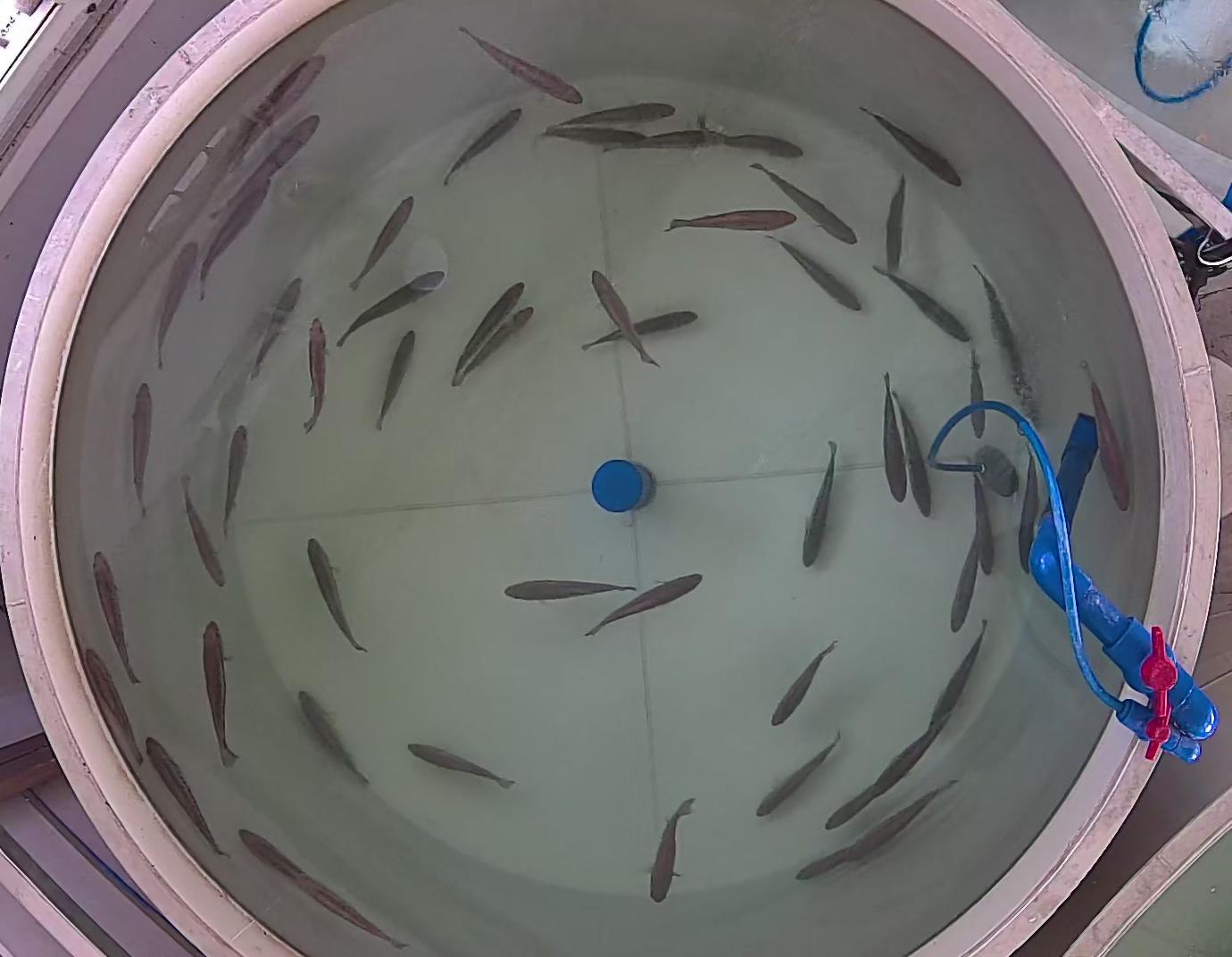

Supplement: S1 Dataset — (ZIP) [file pone.0283671.s001.zip › datasets/00082.jpg]

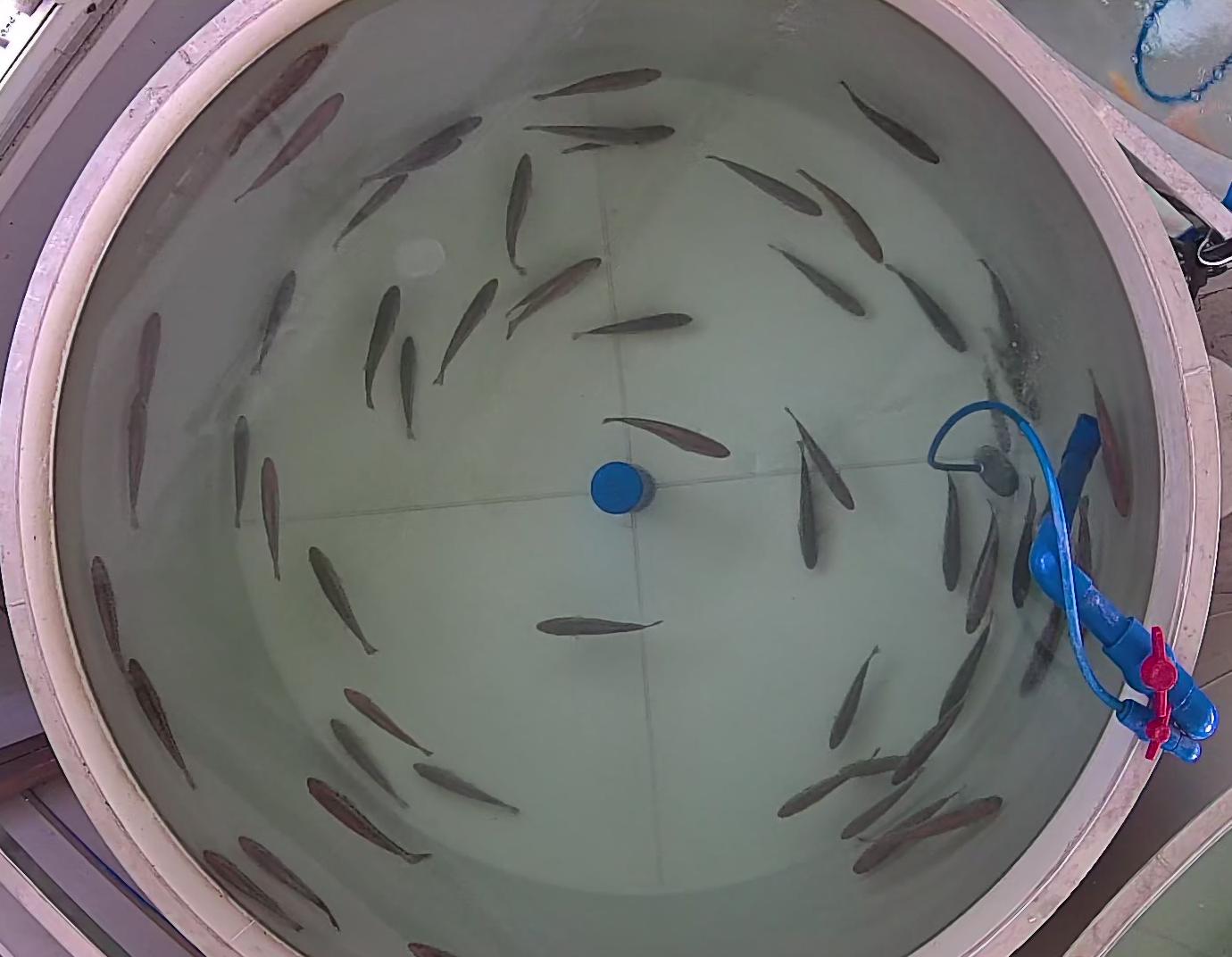

Supplement: S1 Dataset — (ZIP) [file pone.0283671.s001.zip › datasets/00083.jpg]

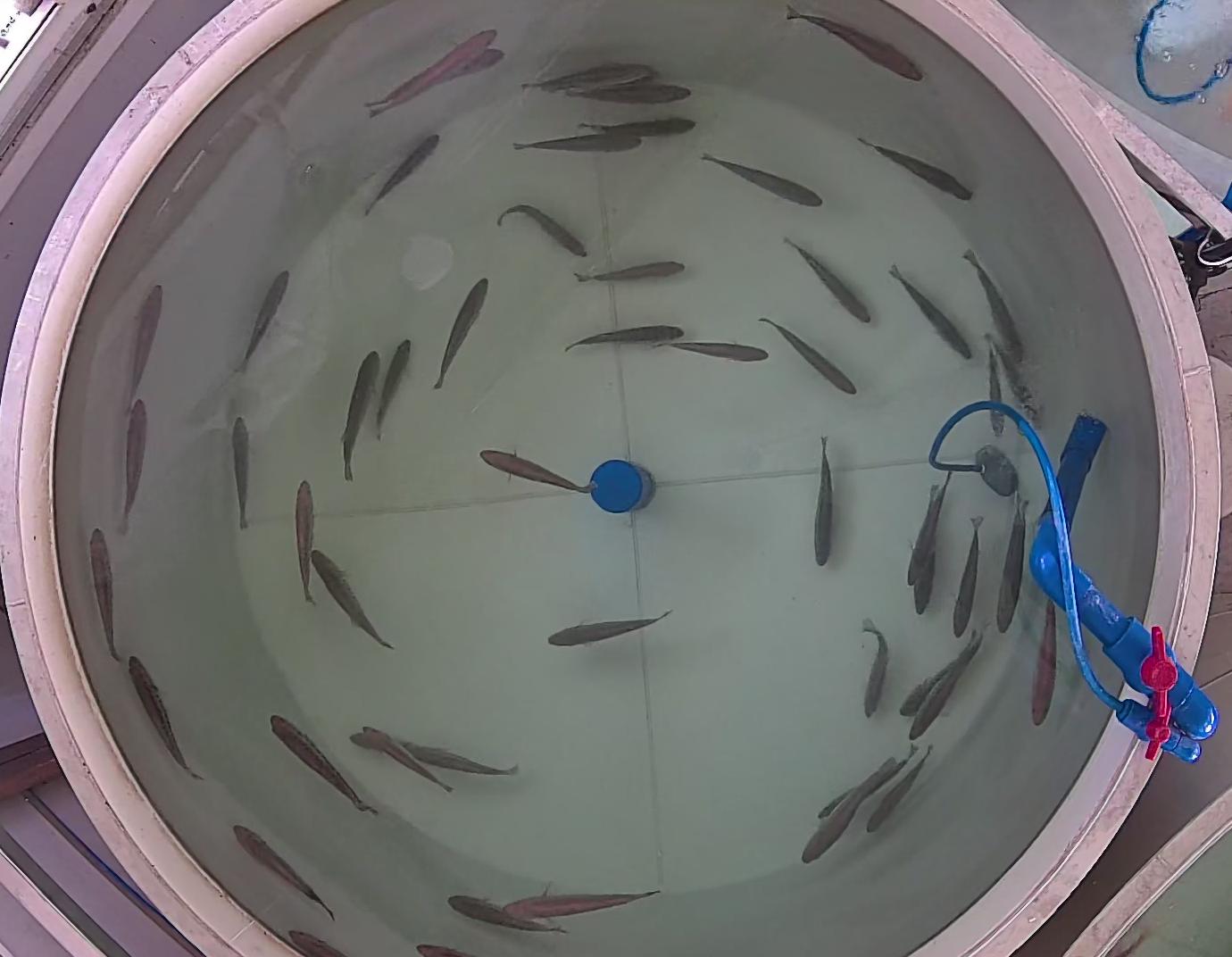

Supplement: S1 Dataset — (ZIP) [file pone.0283671.s001.zip › datasets/00084.jpg]

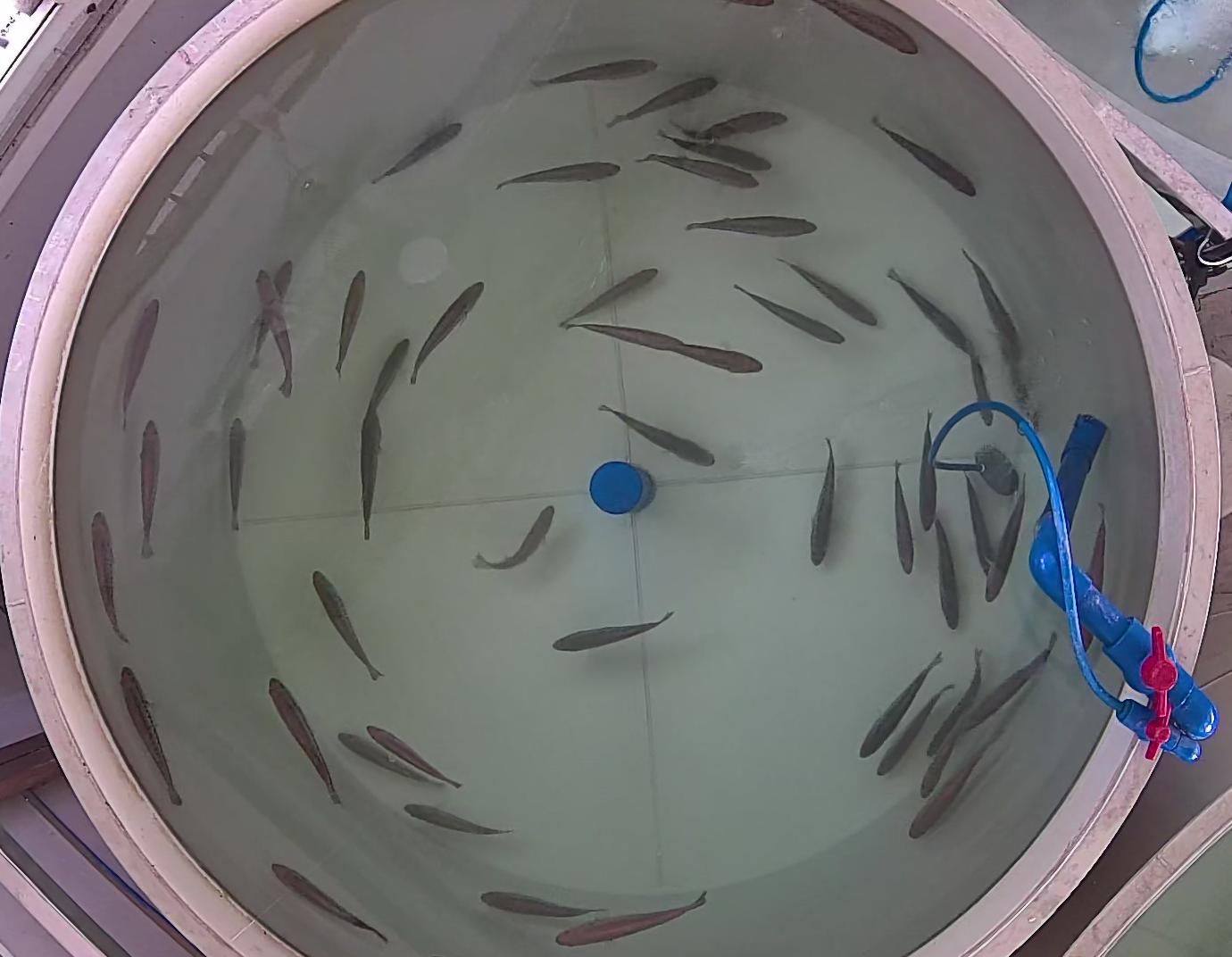

Supplement: S1 Dataset — (ZIP) [file pone.0283671.s001.zip › datasets/00085.jpg]

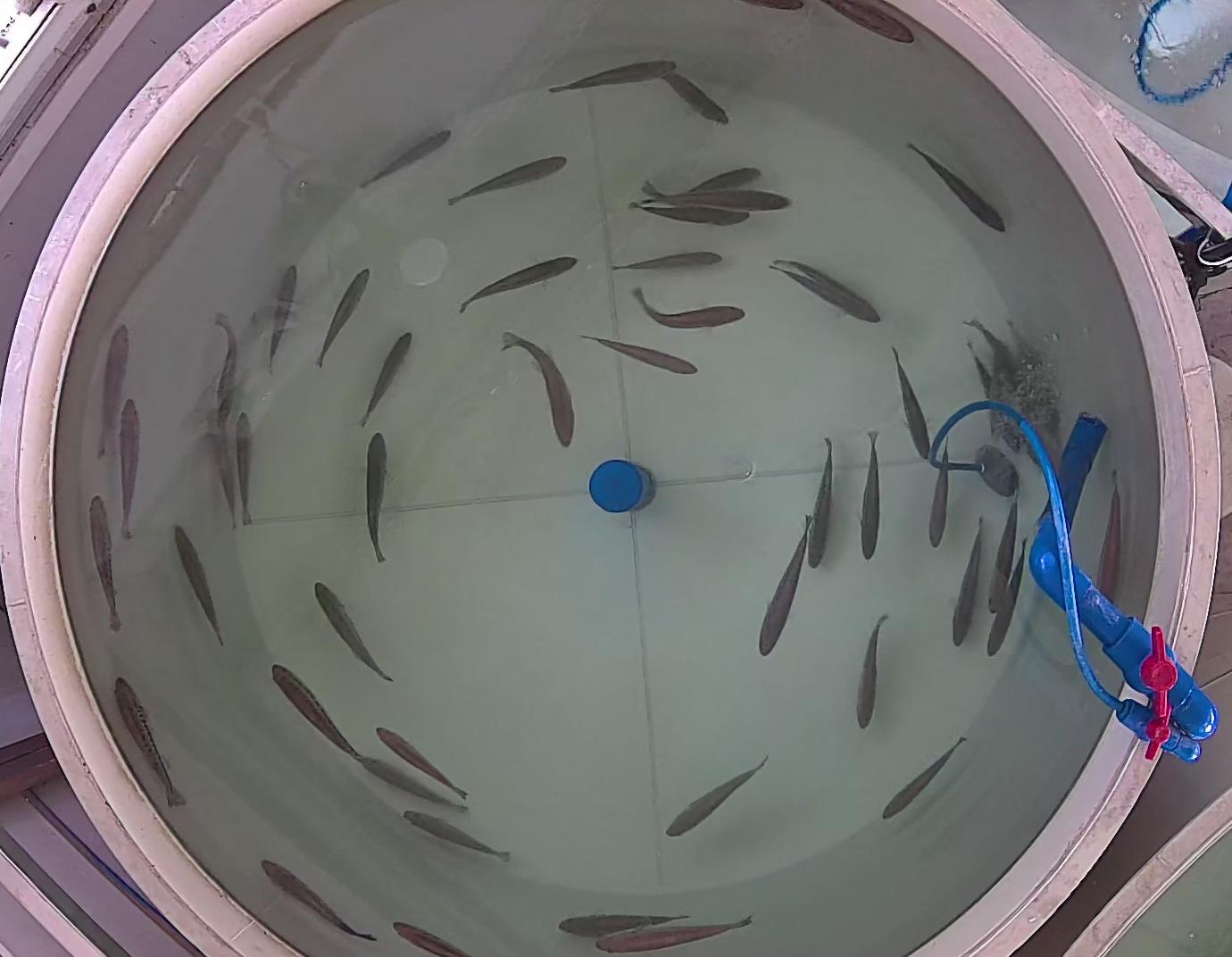

Supplement: S1 Dataset — (ZIP) [file pone.0283671.s001.zip › datasets/00086.jpg]

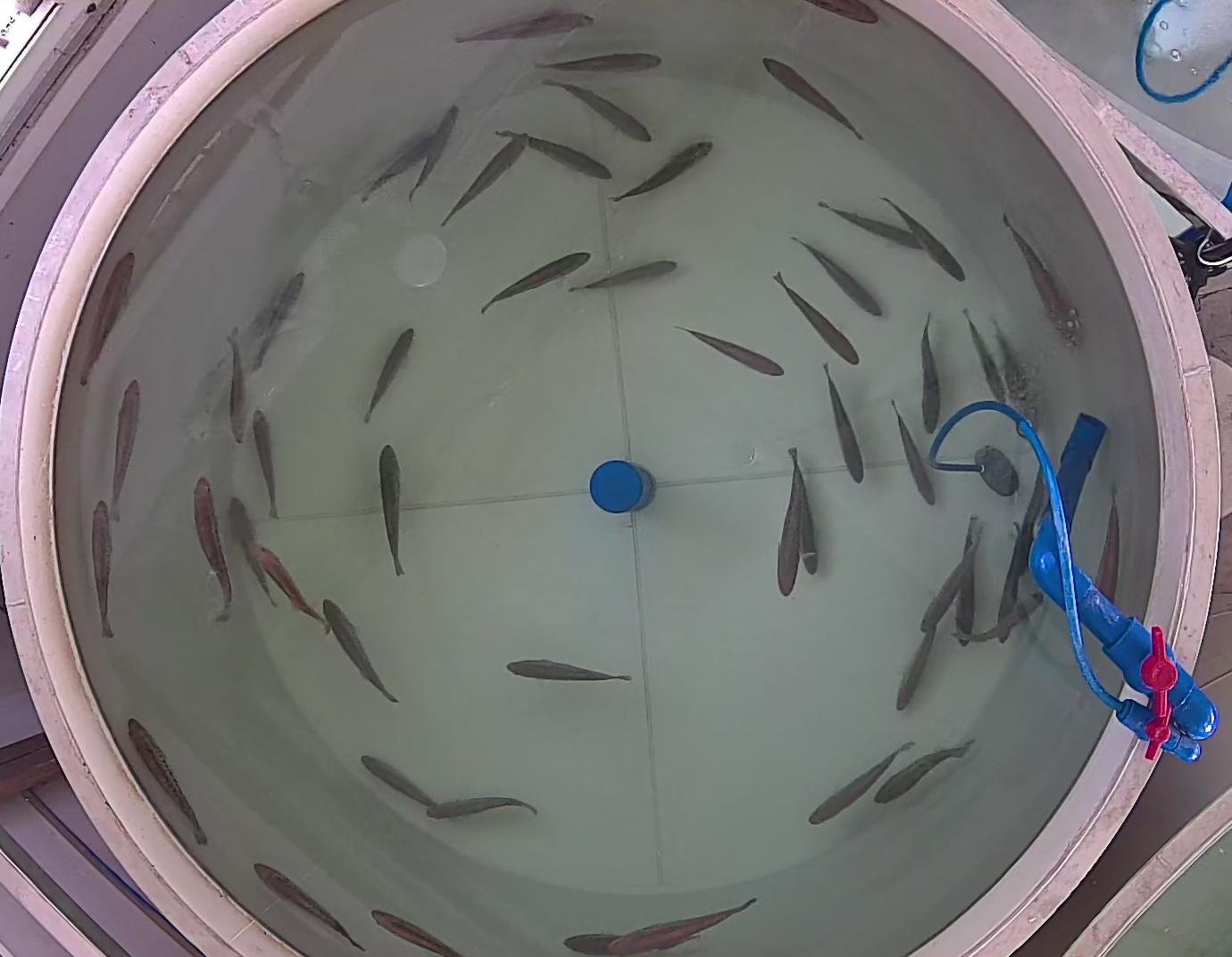

Supplement: S1 Dataset — (ZIP) [file pone.0283671.s001.zip › datasets/00087.jpg]

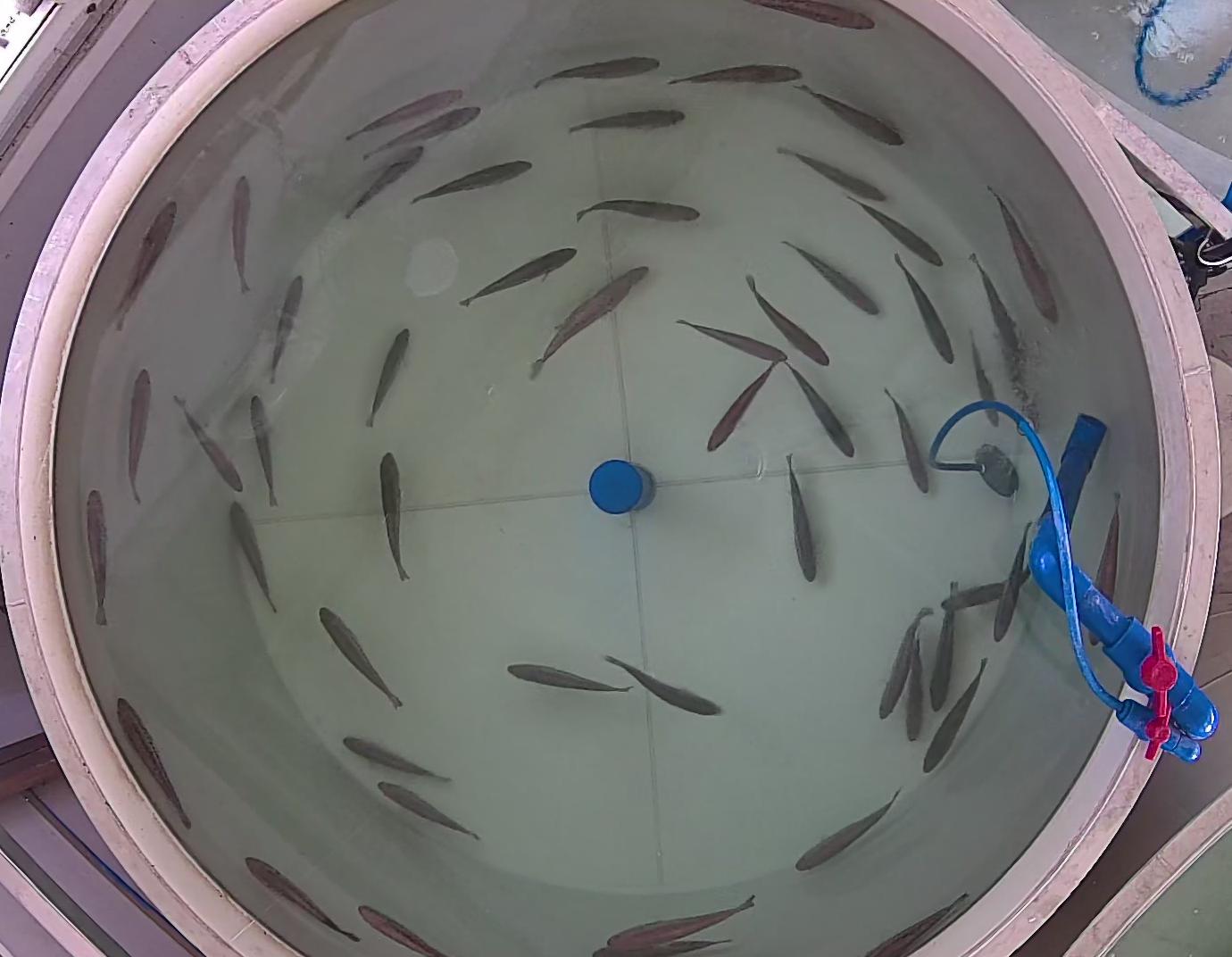

Supplement: S1 Dataset — (ZIP) [file pone.0283671.s001.zip › datasets/00088.jpg]

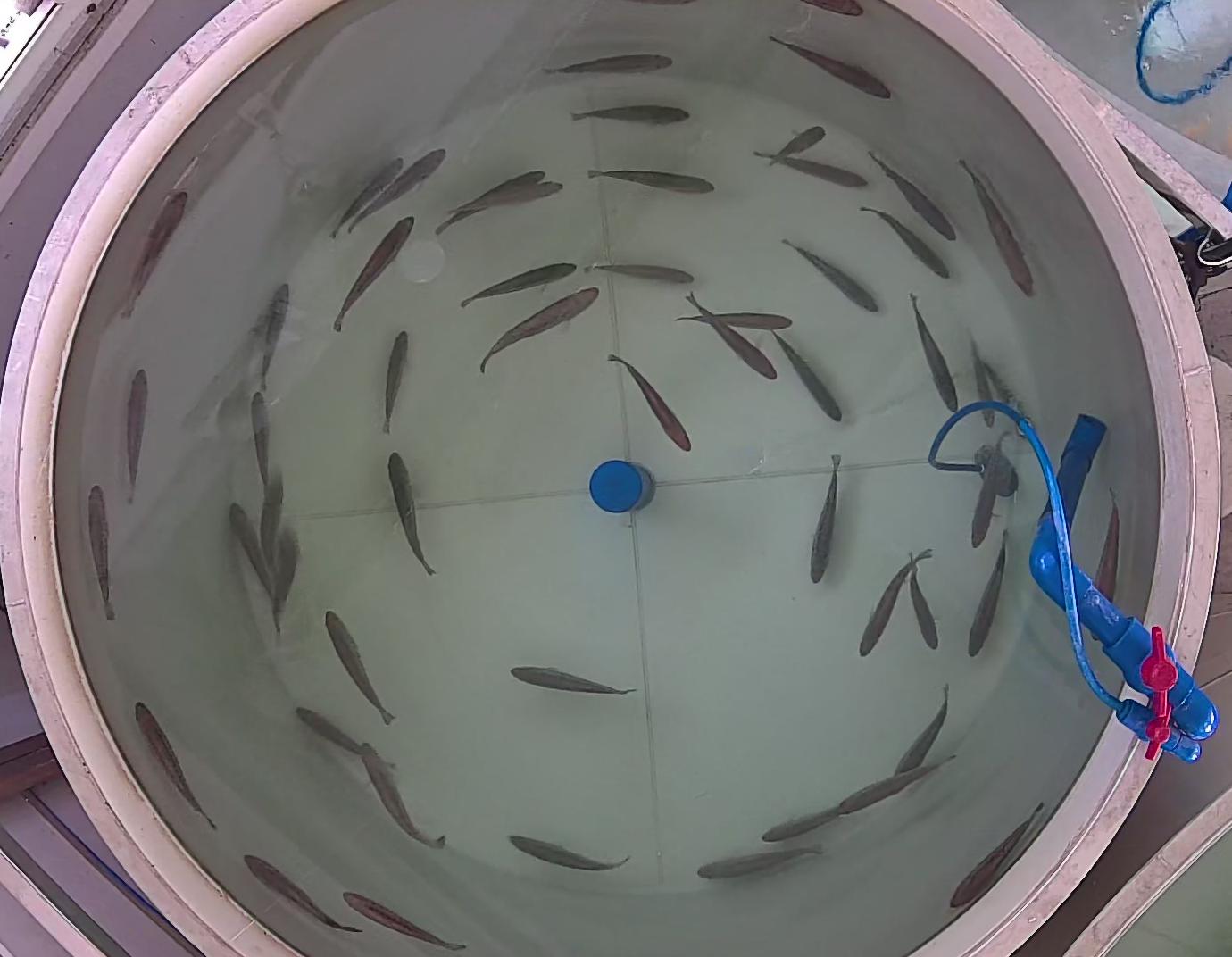

Supplement: S1 Dataset — (ZIP) [file pone.0283671.s001.zip › datasets/00089.jpg]

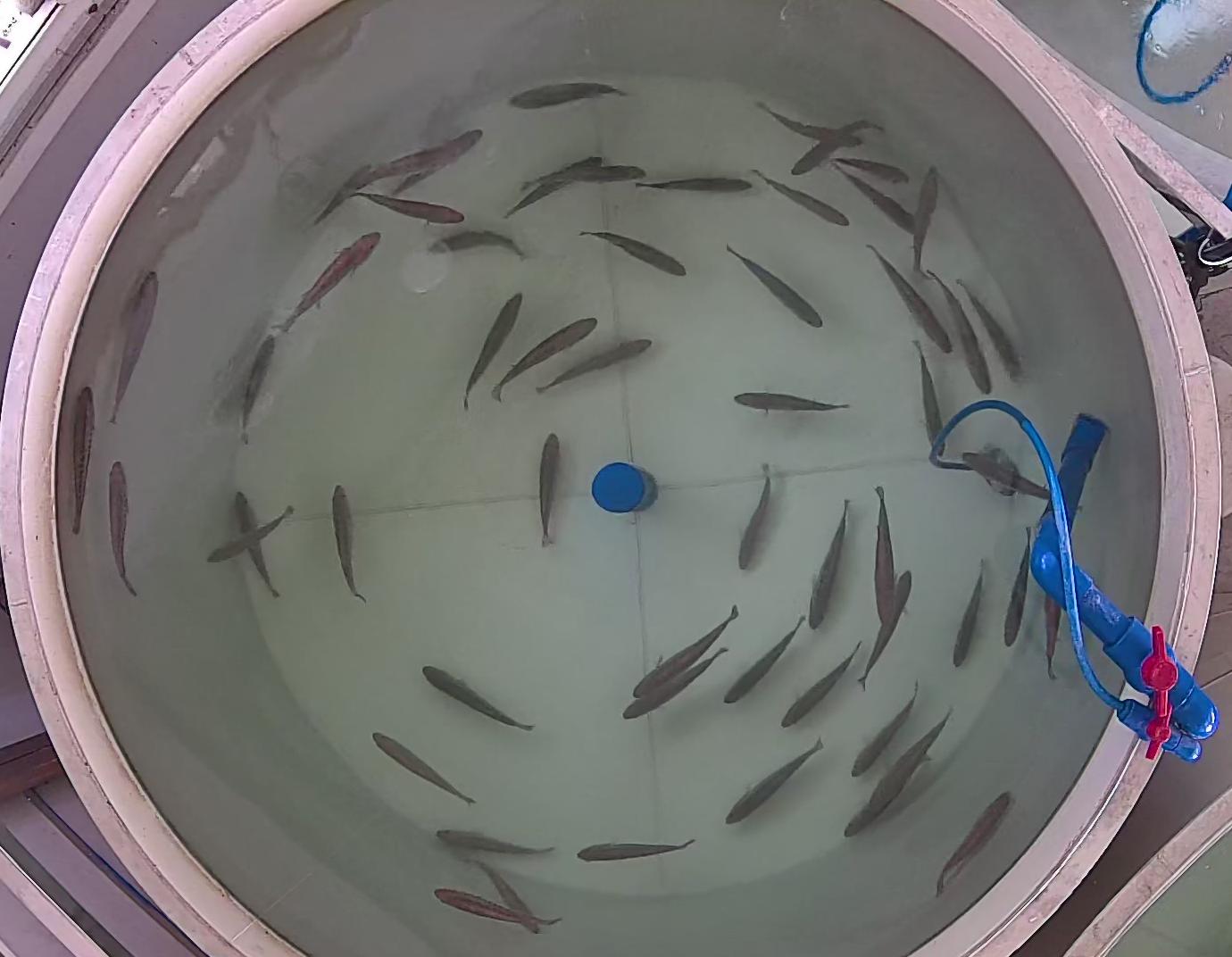

Supplement: S1 Dataset — (ZIP) [file pone.0283671.s001.zip › datasets/00090.jpg]

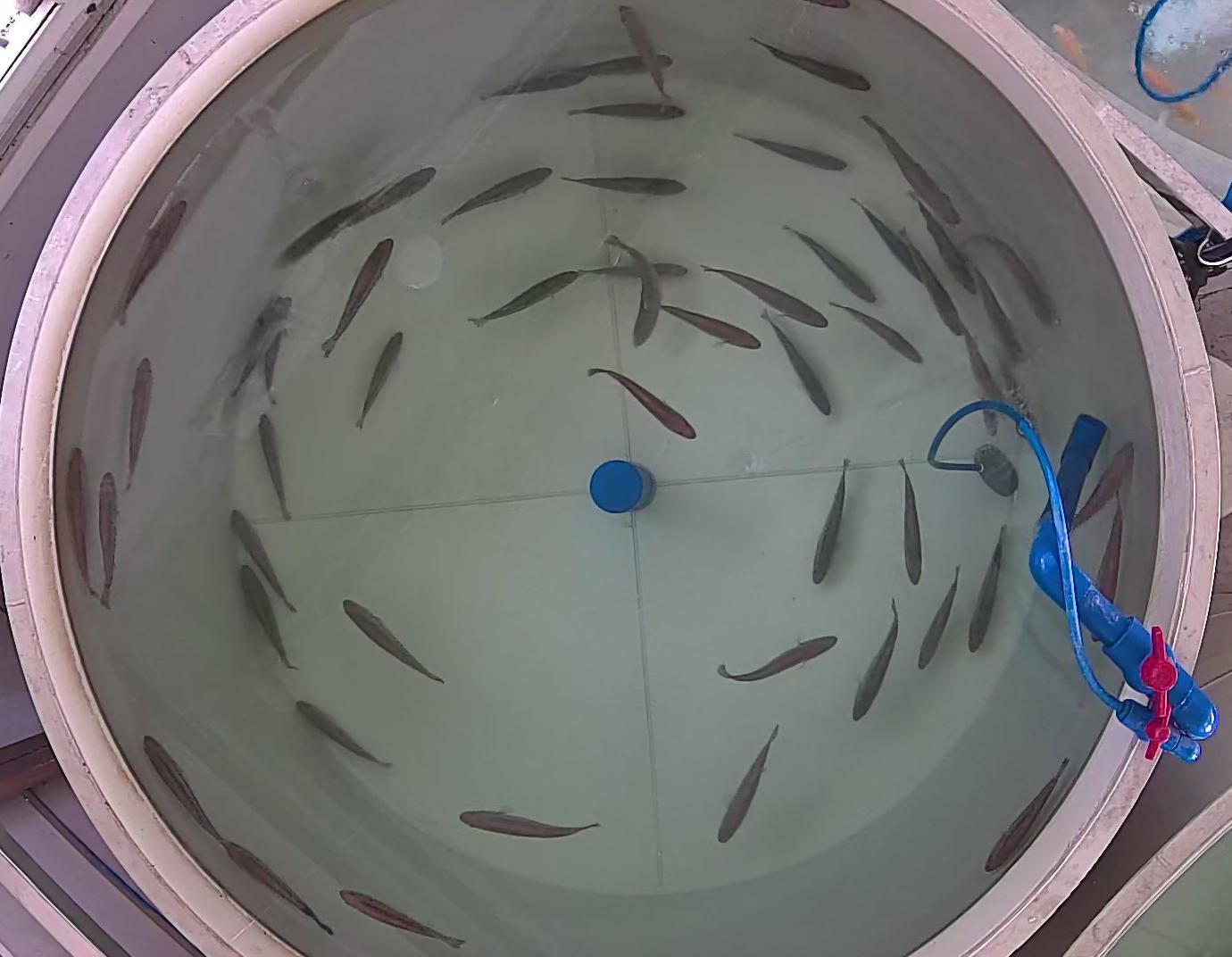

Supplement: S1 Dataset — (ZIP) [file pone.0283671.s001.zip › datasets/00091.jpg]

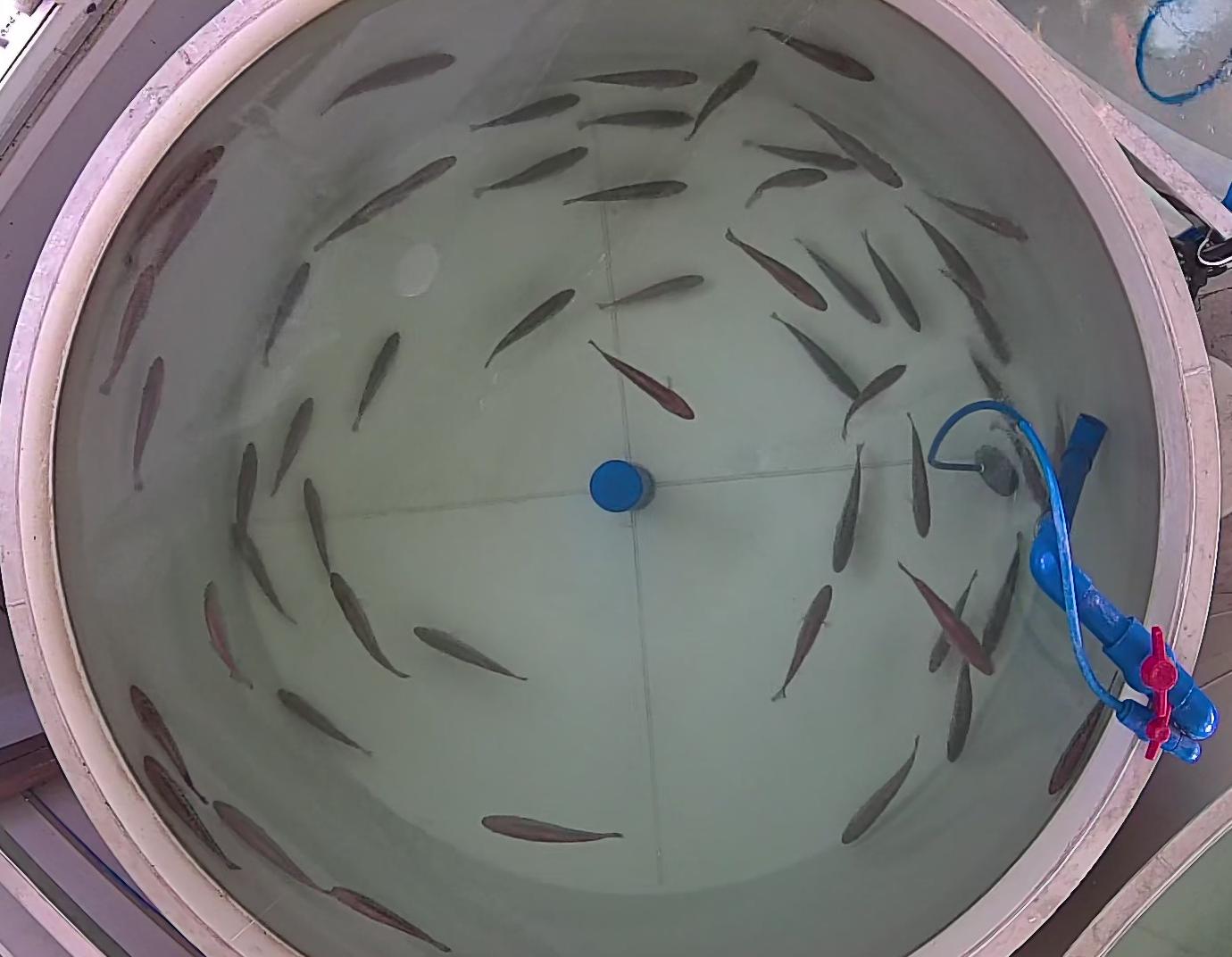

Supplement: S1 Dataset — (ZIP) [file pone.0283671.s001.zip › datasets/00092.jpg]

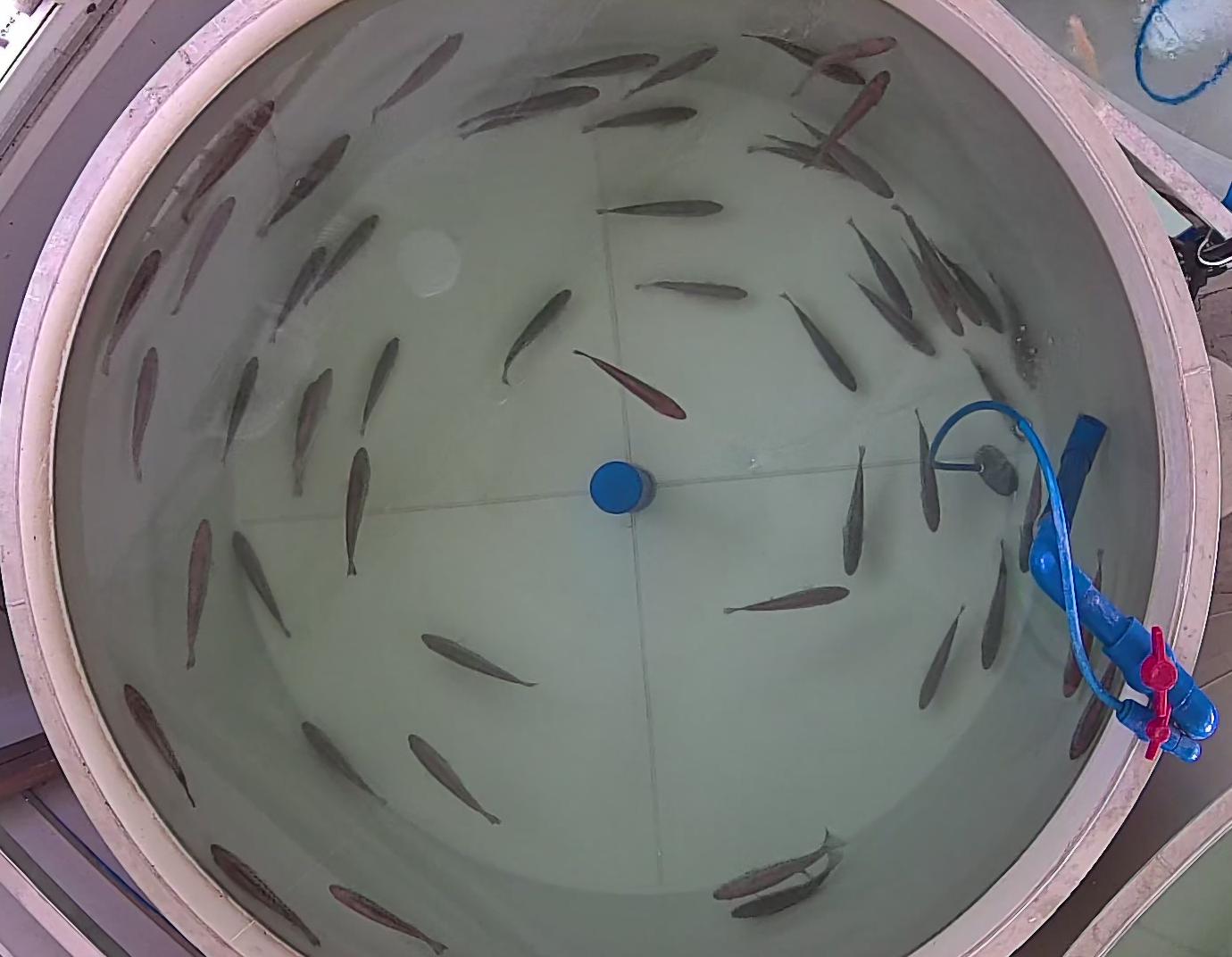

Supplement: S1 Dataset — (ZIP) [file pone.0283671.s001.zip › datasets/00093.jpg]

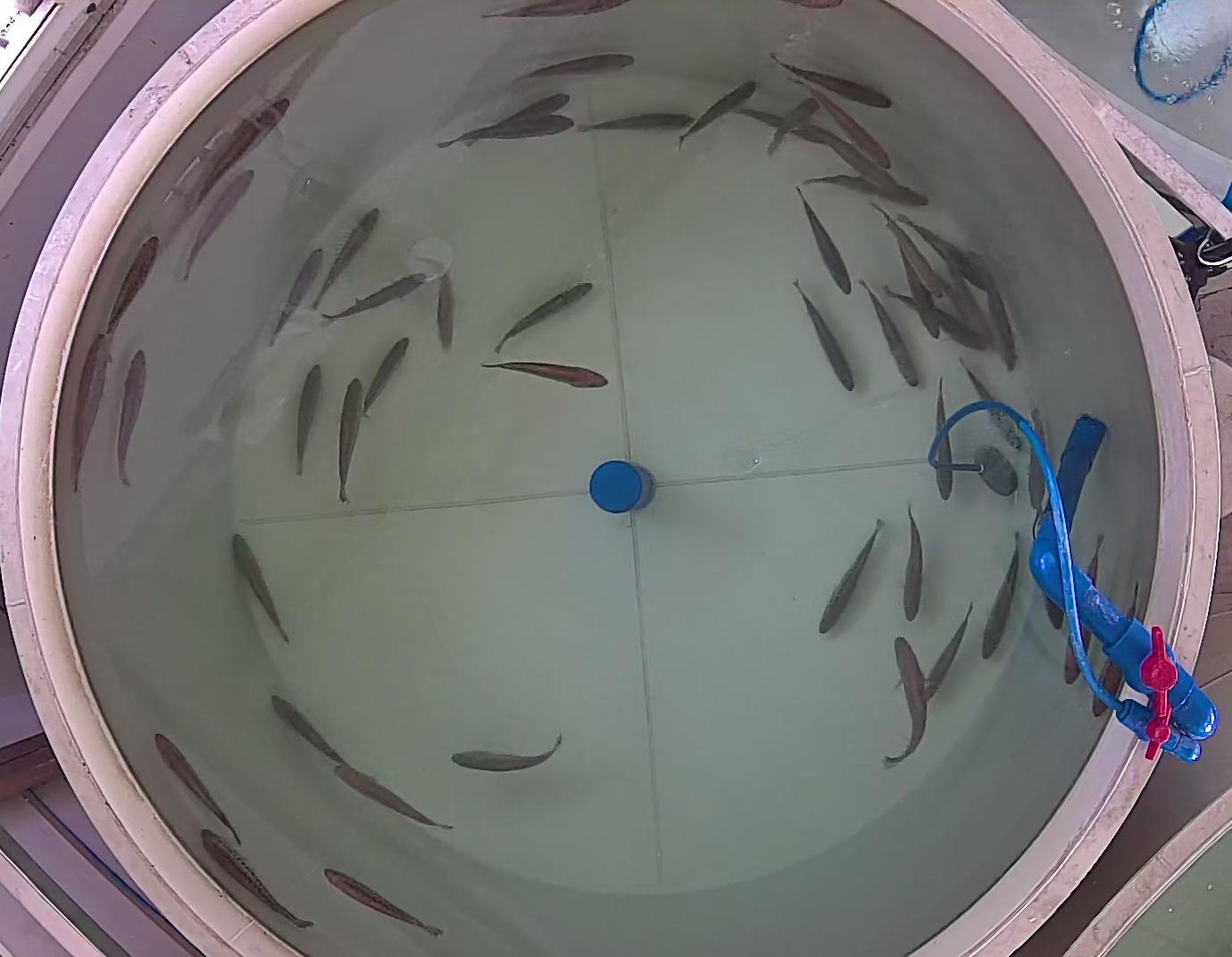

Supplement: S1 Dataset — (ZIP) [file pone.0283671.s001.zip › datasets/00094.jpg]

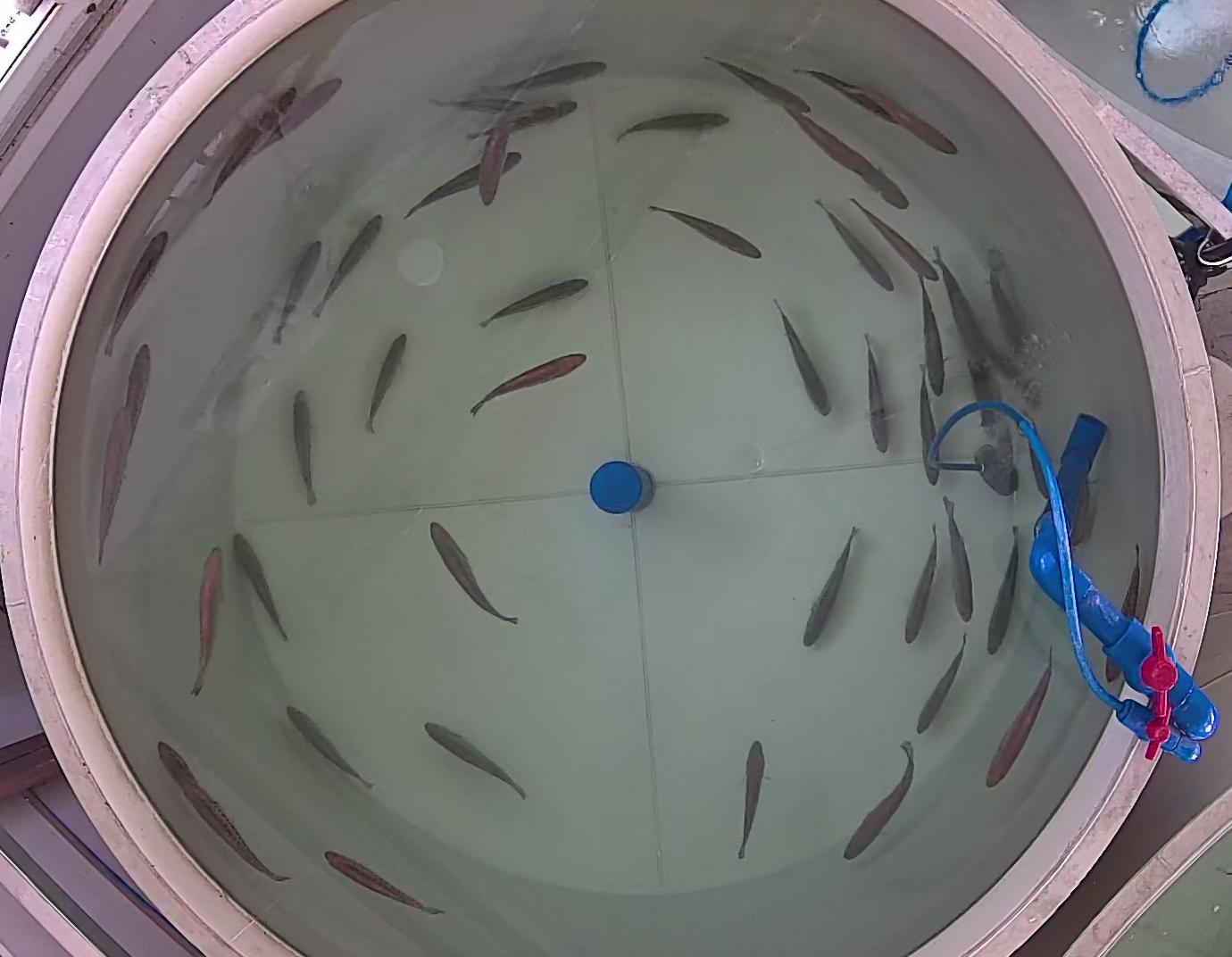

Supplement: S1 Dataset — (ZIP) [file pone.0283671.s001.zip › datasets/00095.jpg]

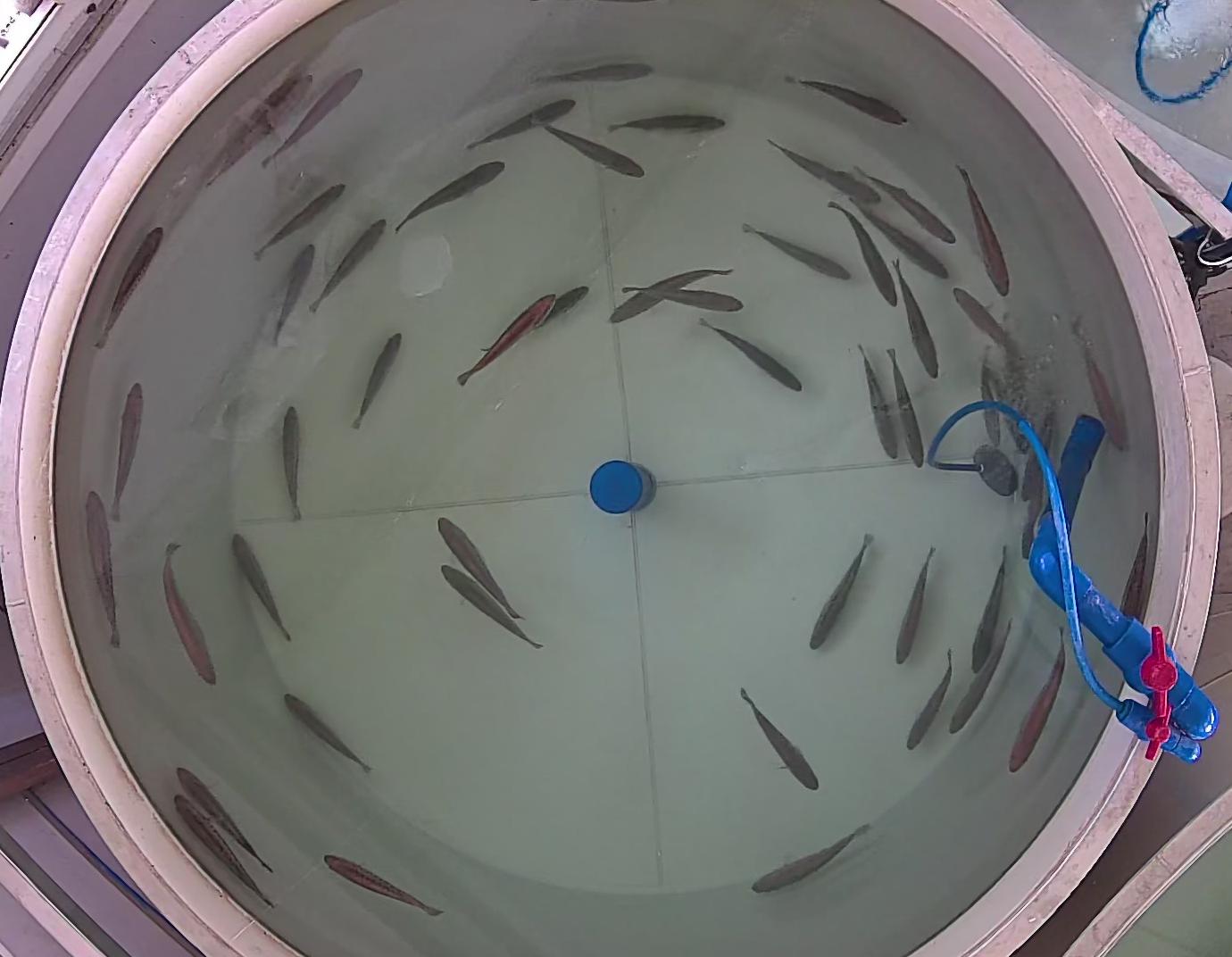

Supplement: S1 Dataset — (ZIP) [file pone.0283671.s001.zip › datasets/00096.jpg]

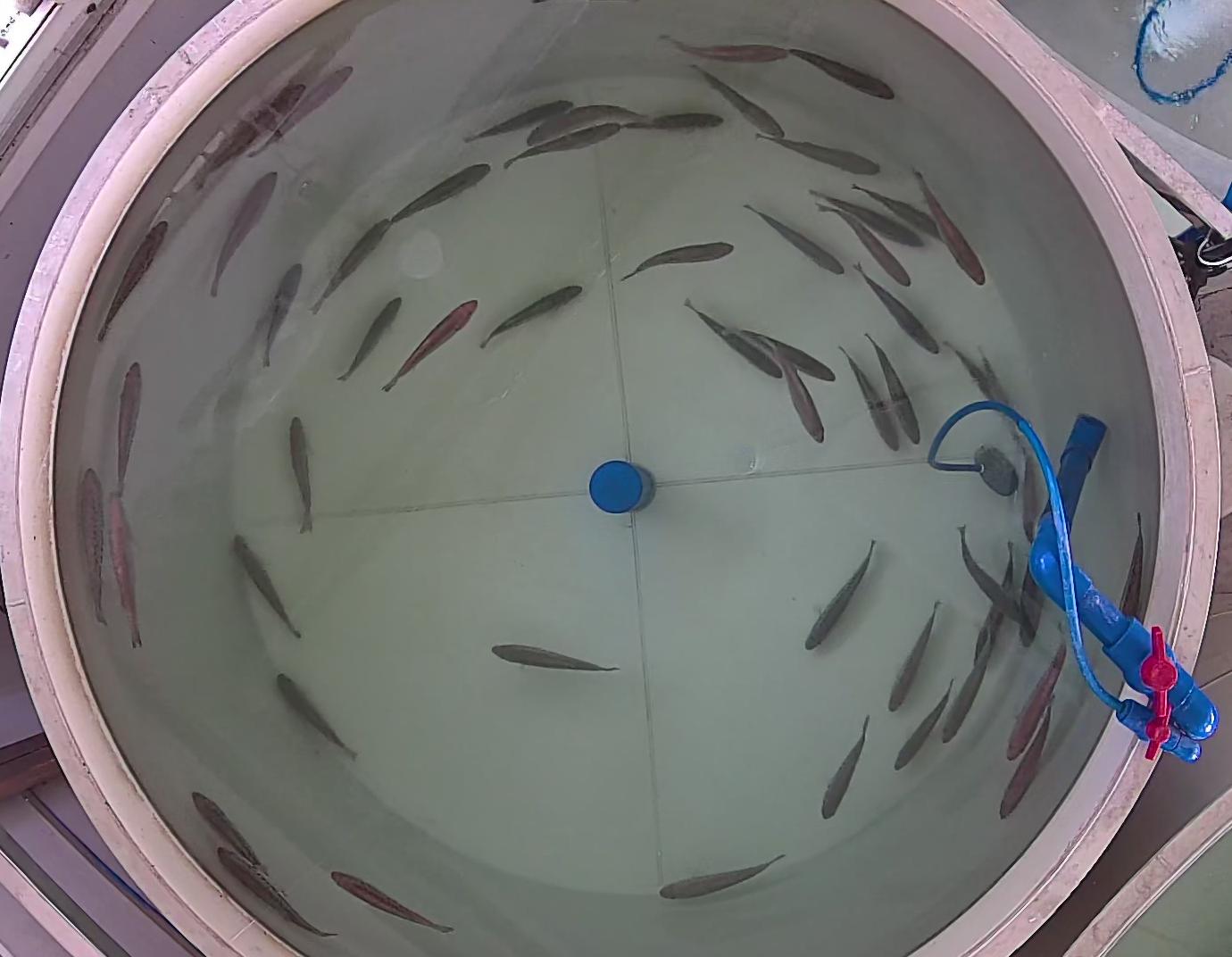

Supplement: S1 Dataset — (ZIP) [file pone.0283671.s001.zip › datasets/00097.jpg]

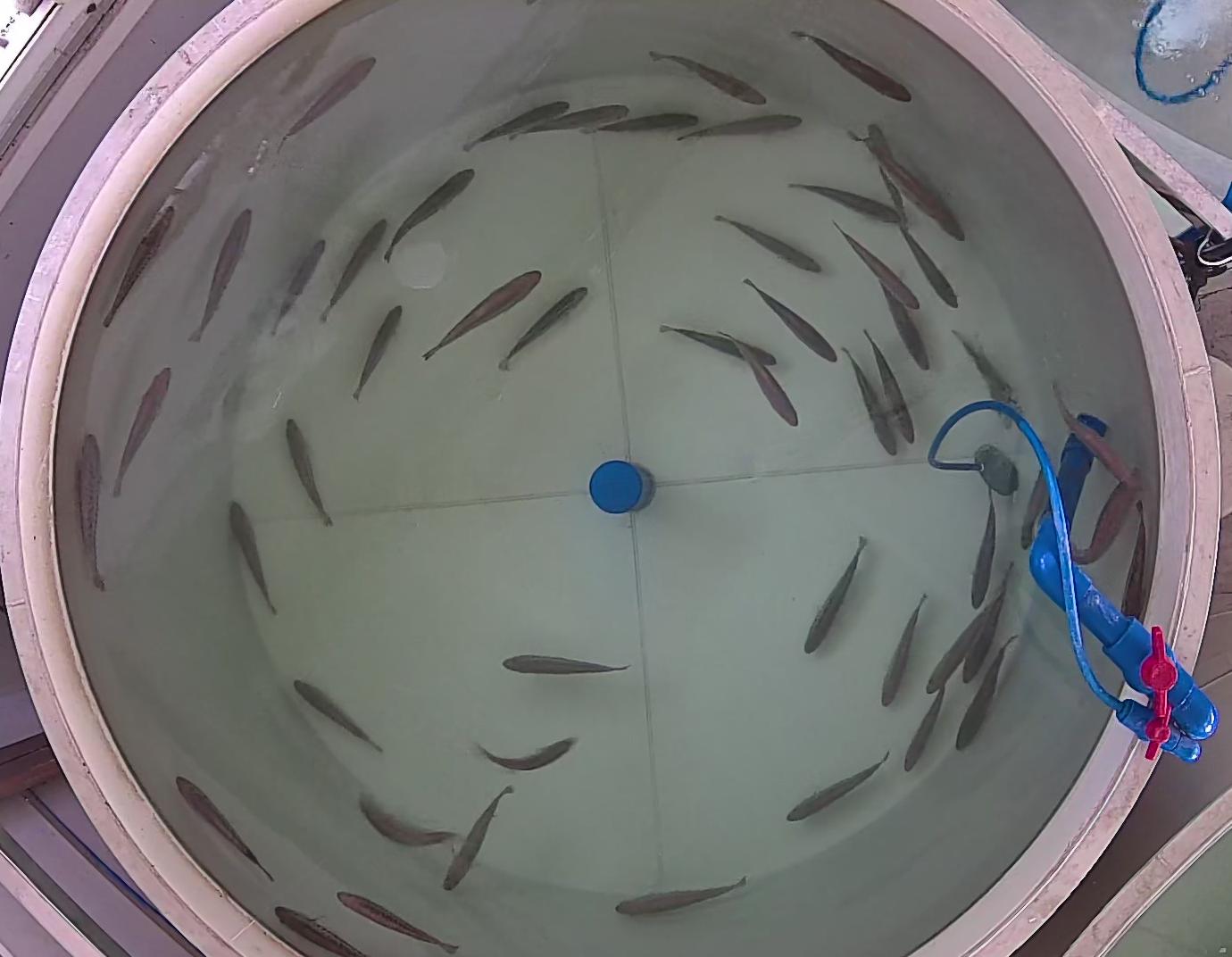

Supplement: S1 Dataset — (ZIP) [file pone.0283671.s001.zip › datasets/00098.jpg]

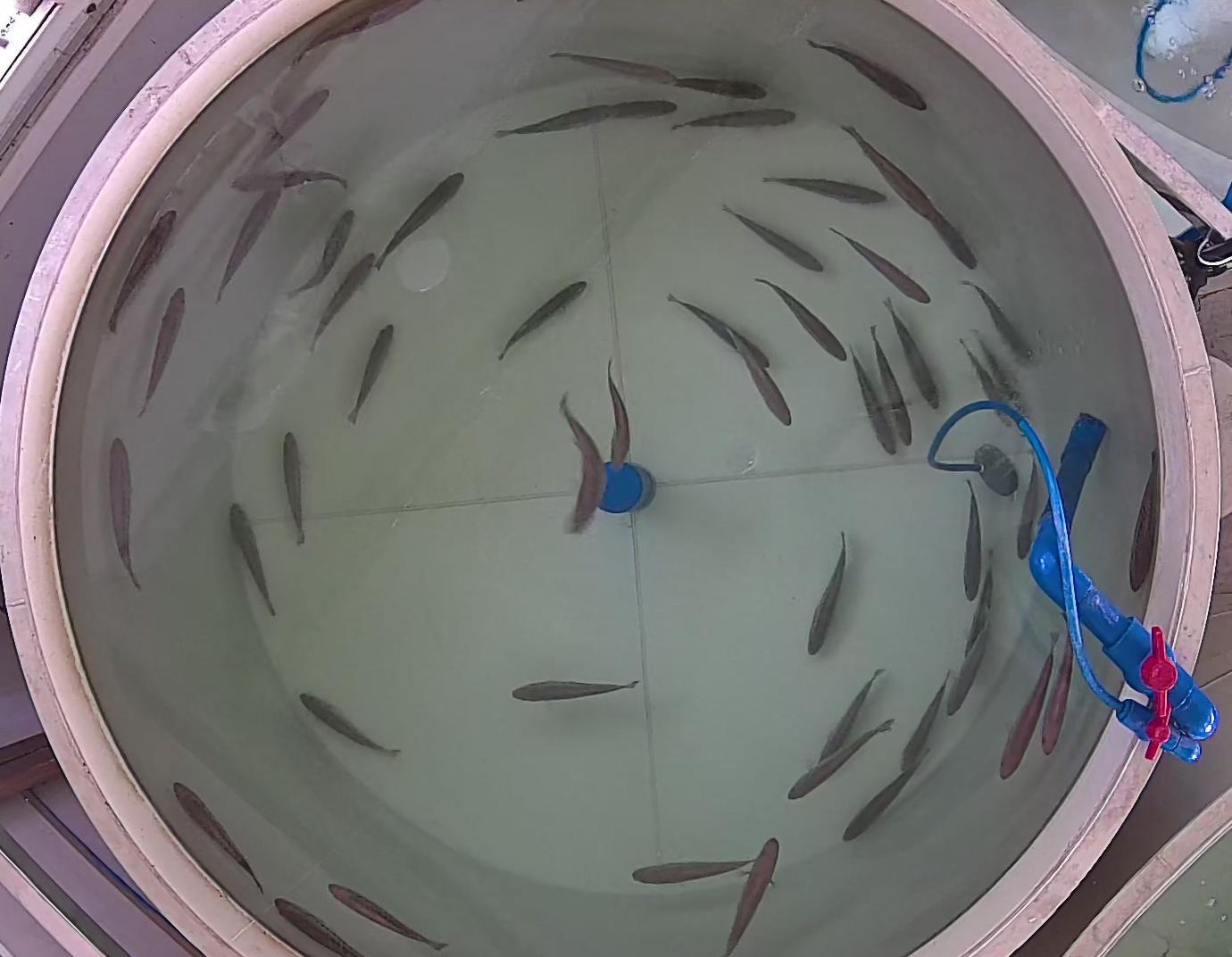

Supplement: S1 Dataset — (ZIP) [file pone.0283671.s001.zip › datasets/00099.jpg]

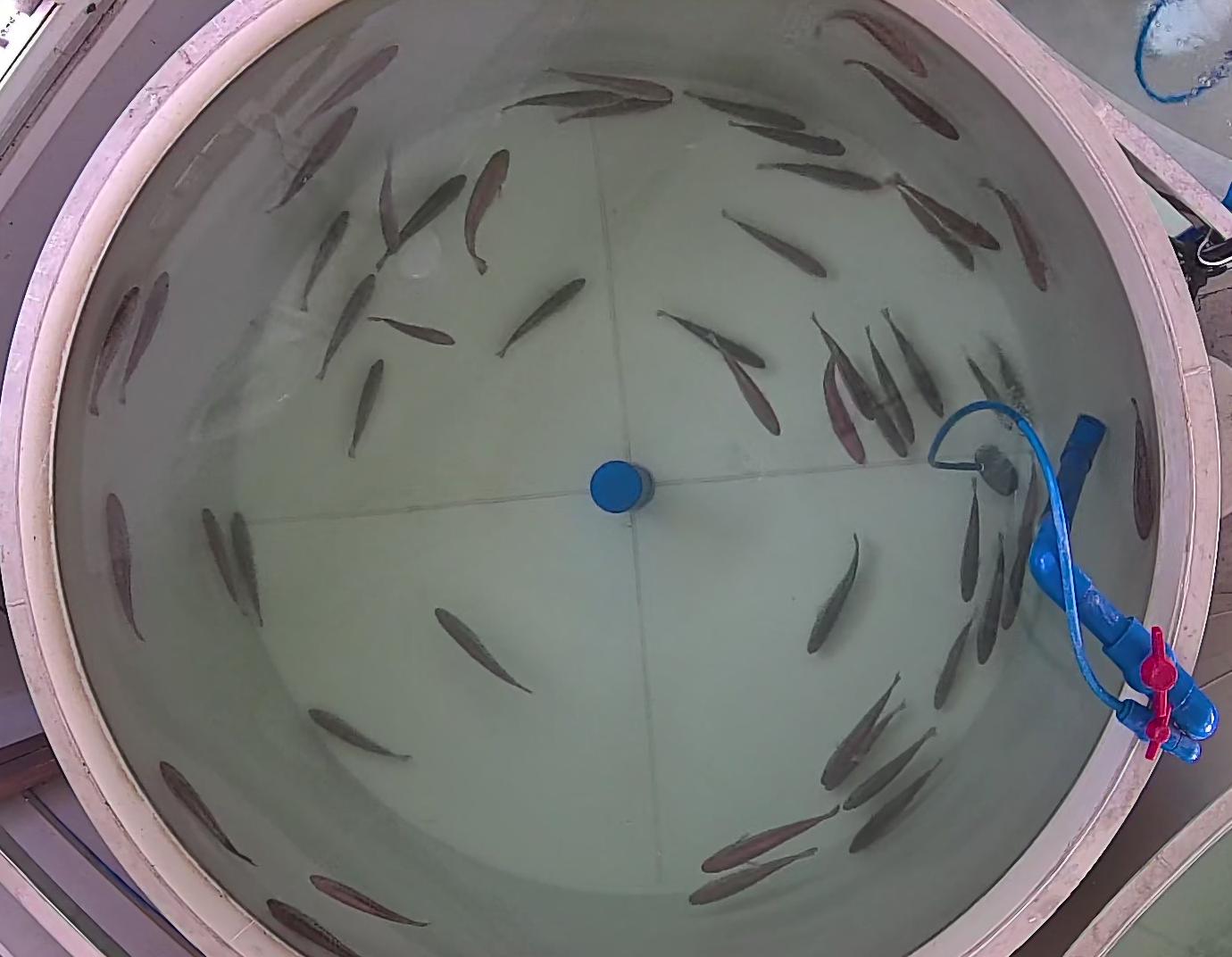

Supplement: S1 Dataset — (ZIP) [file pone.0283671.s001.zip › datasets/00100.jpg]
